# High-Throughput Synthesis and Screening of Cyclometalated Iridium Complexes for Antibacterial Applications

**DOI:** 10.1021/acs.inorgchem.6c03421

**Published:** 2026-10-28

**Authors:** David R. Husbands, Megan G. Dangerfield, Athi Welsh, Lukas Geciauskas, Çağrı Özsan, Kiera Robinson, Richard Gammons, Angelo Frei

**Affiliations:** † Department of Chemistry, 8748 University of York, York YO10 5DD, U. K.; ‡ Department of Chemistry, Biochemistry & Pharmaceutical Sciences, University of Bern, Freiestrasse 3, Bern 3012, Switzerland

## Abstract

The discovery of novel antibiotics to address the rise of antimicrobial resistance is an increasingly pressing issue for global health. In recent years, transition-metal complexes have gained attention as potential antibacterial agents. We herein describe a new methodology to generate novel cyclometalated iridium compounds with bidentate Schiff ligands in a high-throughput combinatorial approach, alongside the expansion of an existing approach using bidentate pyridine-triazole ligands, with the aim of finding new metalloantibiotics. A total of 552 new complexes were synthesized and evaluated. Variety in the libraries was introduced by including three cyclometalating phenylpyridine ligands, demonstrating that the synthesis can tolerate both electron-rich and electron-poor metal scaffolds while using mild and biologically compatible conditions. Screening of the crude reaction mixtures in a direct-to-biology approach revealed a number of highly active complexes against Gram-positive bacteria, which was confirmed by resynthesis and further testing of lead compounds. This approach widens the scope of metal complex combinatorial chemistry, enabling rapid exploration of the chemical space involving cyclometalated iridium complexes.

## Graphical abstract

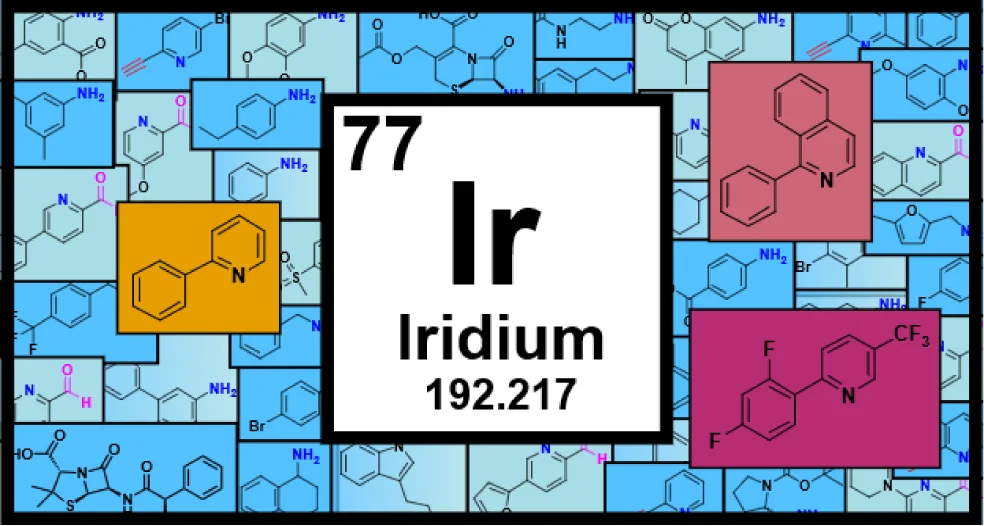

## Introduction

Antimicrobial resistance (AMR) to current antibiotics has been designated as one of the top threats to global health by the World Health Organization.^1,2^ Exacerbating the problem is a lack of new antibiotics in clinical trials, with only 19 antibacterial drugs being approved for use between 2013 and 2023.^3^ More problematically, only two of these represent a new chemical class, with significant reliance on modifying existing drugs with established modes of action. This highlights a growing need to discover new antimicrobial drugs, along with better stewardship of existing medicines to combat AMR.^4,5^

One underutilized source of new antimicrobial compounds is metal complexes, which in a screen by the Community for Open Antimicrobial Drug Discovery (CO-ADD) outperformed purely organic compounds as nontoxic, effective antimicrobials with a hit rate of 9.9% to 0.87%.^6^ As inherently 3-dimensional structures, they represent an underexplored chemical space with regard to antibacterial activity and exhibit modes of action distinct from those of many organic drugs.^6,7^ Additionally, metal complexes exhibit several advantages over organic small molecules, from easily tunable ligand design, variations in coordination geometries, and the ability to undergo ligand exchange. When coupled with the potential for redox-active centers and multiple modes of action such as photophysical reactive oxygen species (ROS) generation, protein binding, and DNA intercalation, metal complexes have significant potential as therapeutics.^8,9^ One other advantage over traditional organic drugs is that many metal complexes can be synthesized in one-step reactions. This characteristic can be harnessed using combinatorial chemistry, where suitable metal complex scaffolds are reacted with ligand building blocks in high conversions, allowing for rapid exploration of this chemical space.^10^

Combinatorial chemistry lends itself well to high-throughput screening, and if biologically compatible solvents are used (such as dimethyl sulfoxide or water), the resulting libraries of compounds can be screened for antibacterial activity in a “direct-to-biology” method. This approach has been successfully implemented as a way of efficiently identifying target compounds, utilizing high-throughput synthesis of compound libraries and testing reaction crudes without prior purification.^11–21^ At the same time, individual reaction components are tested to exclude potential false positives from biologically active building blocks. High-yielding reactions are favored as the majority of the species present will be the target metal complex.^22,23^ Screening is followed by hit validation, where promising compounds are resynthesized, purified, and then retested to confirm their biological activity. Overall, this technique enables rapid coverage of a large chemical space, improves synthesis efficiency by quickly excluding inactive compounds, and funnelling down to a small number of compounds with desirable properties.

Previously, our group and others have utilized combinatorial Schiff-base chemistry to create and test libraries of compounds, with Ru­(II)­(*p*-cymene), Mn­(I) tricarbonyl, and Re­(I) tricarbonyl constituting suitable metal scaffolds.^22–25^ Upon combination with pyridine-2-carbaldehydes and primary amines, the resulting bidentate Schiff-base ligand coordinates the metal center in efficient one-pot reactions. Similarly, for Fe­(III), 2-hydroxybenzaldehydes can be combined with 2-aminophenols and the metal scaffold in the same manner.^12^ More recently, our attention has turned to compounds of iridium,^26,27^ which has been studied extensively for biological activity, mainly with anticancer applications.^28^ Commonly, cyclopentadienyl-Ir­(III) piano stool (referred to as IrCp) and bis-cyclometalated 2-phenylpyridine Ir­(III) (referred to as IrCN) cores are used, often with a varying bidentate ligand (Figure 1).^29^ In the case of IrCN-based molecules, the resulting complexes can be highly phosphorescent,^30,31^ raising the possibility of photodynamic therapeutics.

**Figure 1:**
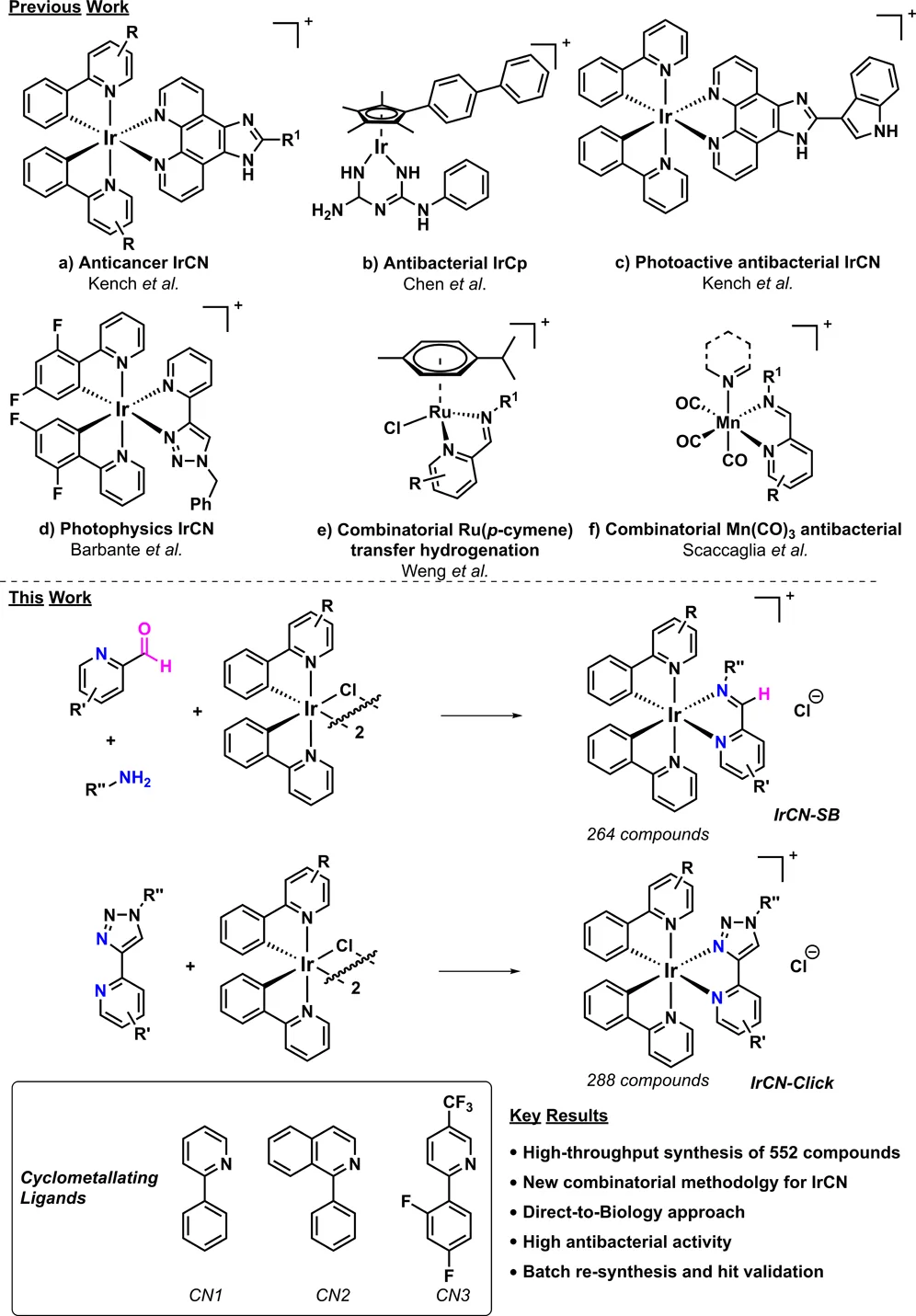
(Top) Previous work on biologically active iridium complexes for anticancer, antibacterial, and photophysical applications, with examples of metal scaffolds suitable for combinatorial chemistry: a) IrCN complexes for anticancer applications,^35^ b) antibacterial IrCp complex,^37^ c) photoactive antibacterial IrCN complexes,^36^ d) IrCN complex used in a photophysical study,^30^ reproduced with permission from ref 30, copyright 2014 John Wiley and Sons; e) structure of Ru­(*p*-cymene) Schiff-base complexes made using combinatorial chemistry for transfer hydrogenation in cells,^24^ reproduced with permission from ref. 24, Copyright 2020 John Wiley and Sons; f) structure of Mn­(CO)_3_ Schiff-base complexes made using combinatorial chemistry for antimicrobial activity;^23^ available under a CC BY-NC 4.0 license, copyright Scaccaglia et al.; (Bottom) a summary of this work with the key findings.

Cyclometalated IrCN complexes have been the target of several combinatorial studies in previous work, notably solid-phase synthesis of an IrCN library to screen for red phosphorescent emitters for use in applications in OLEDs,^32^ the room-temperature synthesis of 90 IrCN complexes with varying ancillary ligands in dichloromethane to explore and tune molecular color,^33^ and a large-scale synthesis and study of 1440 IrCN complexes for exploring photophysical properties.^34^ In the last example, the synthetic methodology involved forcing conditions of heating in DMSO at 150 °C overnight. In the medicinal chemistry field, the most prominent combinatorial work with IrCN has been undertaken by Kench et al., taking advantage of the photophysical properties of these complexes for irradiation-induced toxicity for anticancer and antimicrobial applications.^35,36^ Other notable antimicrobial applications have been determined using individual IrCN and IrCp compounds, demonstrating this scaffold’s potential for high activity against bacteria.^37–40^ In the studies involving cyclometalated IrCN complexes, there is often very good activity against Gram-positive bacteria, and almost no activity against Gram-negative strains, possibly due to the more complex outer membrane preventing permeation by this class of metallodrug.^41^ The modes of action vary between complexes but tend to be either membrane targeting (membrane depolarization, disruption, pore formation) or generation of ROS, especially upon photoirradiation, or a combination of both.^42^ Alongside effective biofilm disruption that these complexes can demonstrate, there is the possibility of photodynamic therapeutics targeting localized infections. Despite the advancements of combinatorial chemistry for IrCN complexes over the last 20 years, current direct-to-biology studies with cyclometalated IrCN compounds are somewhat limited by synthetic challenges. Although the cyclometalated iridium dimer precursor can be prepared in a simple microwave reaction of a (2-phenyl)­pyridine type ligand with IrCl_3_,^43^ the cleavage of this dimer and coordination by other ligands often requires forcing conditions or toxic solvents.^34,35,40^ The forcing conditions required to coordinate ligands in biologically compatible DMSO (150 °C over several hours) are energy intensive and not necessarily compatible with widely used heating systems (typically a 100 °C ceiling) for high-throughput chemistry. The alternative of chlorinated solvents under mild conditions is both environmentally damaging and incompatible with biological testing, requiring efficient removal of toxic solvents across hundreds of reaction wells.

Our previous research with pyridine–triazole compounds demonstrated that cyclometalated IrCN is a viable scaffold for these ligands in a high-throughput manner, utilizing DMSO and water as a solvent under mild conditions.^44^ This prompted us to expand the chemical space by varying the cyclometalating ligand to investigate its influence on biological activity while also exploring whether established Schiff-base combinatorial chemistry could be applied to this metal scaffold. Successful reactions would represent a significant increase in the available chemical space for exploration, as the pyridine-triazole ligands are limited in scope by the availability of 2-alkyne pyridine building blocks. In contrast, 2-aldehyde pyridines are much more common, and Schiff-base chemistry requires fewer steps than the pyridine–triazole Click ligand synthesis. Libraries of cyclometalated IrCN complexes incorporating Schiff bases and our recently introduced pyridine-triazole ligands, synthesized under mild and biologically compatible conditions, could represent a significant advance for studying the biological activity, photophysical properties, and photocatalytic potential of this class of compounds. Using our existing workflow, we therefore sought to develop and optimize a high-throughput synthetic methodology for these IrCN libraries, characterize the resulting compounds by automated liquid-chromatography–mass spectrometry (LC-MS), and screen them for antibacterial activity using a Direct-to-Biology approach. Promising candidates would be subsequently synthesized on a larger scale under mild conditions, purified, fully characterized, and subjected to further biological evaluation.

## Results and Discussion

### Synthesis and Optimization

Following on from our previously reported results where bidentate pyridine-triazole ligands (referred to as the **Tz-4-P** library) were coordinated to a range of metal scaffolds, we decided to widen the scope of coordination to cyclometalated iridium complexes in a combinatorial manner. The cyclometalated Ir­(III) complexes had the most promising antibacterial activity compared to toxicity to mammalian cells (therapeutic index, TI) in that study. Expansion of the chemical space was executed by varying the phenylpyridine cyclometalating ligand (**IrCN1**) to 1-phenylisoquinoline (**IrCN2**) and 2-(2,4-difluorophenyl)-5-(trifluoromethyl)­pyridine (**IrCN3**). These groups represent a shift in electron-withdrawing/electron-donating effects of the cyclometalating ligands, which manifests as significant changes in UV–vis/phosphorescence spectra relative to **IrCN1** (see Supporting Information Figures S1-S4).^31,34^ Isoquinoline-derived **IrCN2** complexes exhibit a significant red-shift in phosphorescent emission, while electron-deficient **IrCN3** complexes demonstrate blue-shifted emission. **IrCN3** structures have been used as photocatalysts in a number of photoredox transformations in organic chemistry and can be used to promote radical coupling reactions.^45,46^

Utilizing an Opentrons OT-2 liquid-handling robot, we synthesized the previously established pyridine-triazole **Tz-4-P** ligand library from azides generated *in situ* from primary amines (Figure 2), followed by a copper-catalyzed azide–alkyne cycloaddition (CuAAc) “Click” reaction.^47^ This freshly prepared library of 96 compounds (mean conversion by LC-MS peak area % of 78 ± 17%, *c.f.* previously reported conversion of 75 ± 16%, showing high reproducibility in library synthesis)^44^ was successfully coordinated simultaneously to **IrCN1–3**, generating 288 complexes. Both **IrCN2** and **IrCN3** required a higher DMSO:H_2_O ratio compared to **IrCN1** (2:1 compared to 1:1) to achieve good conversions. This was primarily due to the poor solubility of the **IrCN2–3** dimers with the higher water content. This collection of libraries is hereafter referred to as **IrCN-Click**.

**Figure 2:**
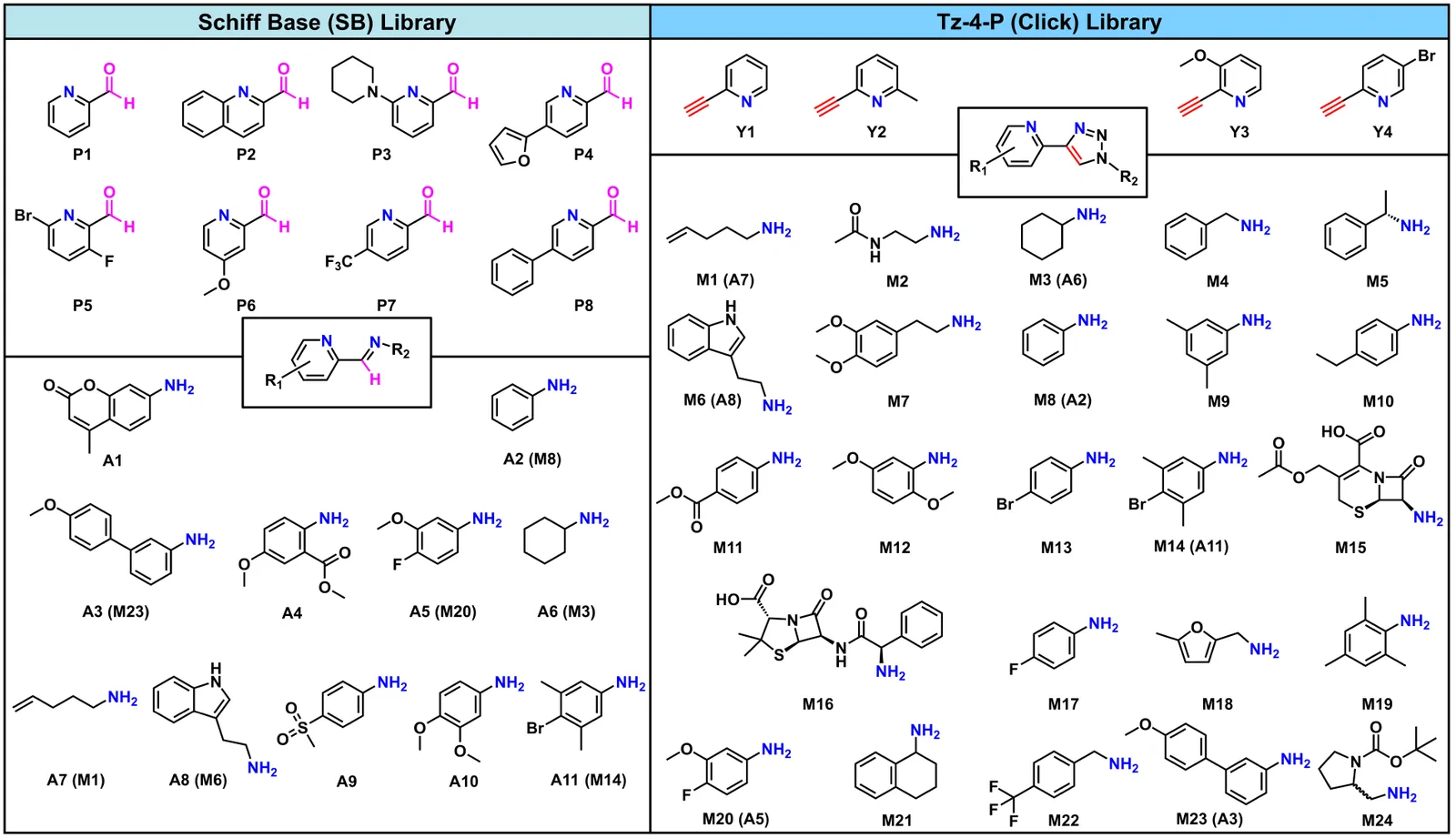
(Left) Building blocks for the **IrCN-SB** libraries; (Right) building blocks for the **Tz-4-P** library corresponding to the **IrCN-Click** libraries.

With these new libraries in hand, we turned our attention to Schiff-base chemistry to provide variation in the bidentate ligand coordinating the cyclometalated IrCN core. There have been reports of Schiff-base ligands comprising 2-pyridinecarbaldehyde derivatives and primary amines coordinating IrCN scaffolds, but they are limited to a small number of compounds with simple building blocks.^48–50^ As stated earlier, complexations of bidentate ligands to cyclometalated IrCN cores require either forcing conditions or nonbiologically compatible solvents such as dichloromethane/methanol. Although amenable to batch synthesis, these protocols are not generally well suited to high-throughput chemistry or biological screening. This led us to explore whether lower-temperature complexations of Schiff-base ligands to IrCN scaffolds could be facilitated by a similar approach to the **IrCN-Click** libraries, by introducing water as a cosolvent to DMSO. This would allow us to side-step forcing conditions or solvent evaporation, giving nontoxic, mild, high-throughput conditions for a direct-to-biology approach.

Initial coordination of building blocks **P1** (2-pyridinecarbaldehyde) and **A2** (aniline) to **IrCN1–3** in a one-pot reaction was optimized on a 100 μL scale in Eppendorf tubes, using LC-MS-determined conversion rates to obtain the best conditions. As with the **IrCN-Click** libraries, early results indicated that the ratio of DMSO:H_2_O solvent was critical for good conversions. Additionally, while reactions did occur at room temperature, higher conversions were achieved at 60 °C. The water in the reaction appears to play a contradictory role—it is required for efficient cleavage of the IrCN dimers, and increasing the quantity of water did substantially increase conversions to product. However, if the ratio of water to DMSO was too high, Schiff-base formation via condensation reaction was suppressed and any product precipitated out. This was primarily an issue for **IrCN3**, where precipitation was observed even at low levels of water. The optimized conditions were determined to be 60 °C in 3:2 DMSO:H_2_O for 18 h for **IrCN1–3**. It should be noted that these conditions are mild and use biologically compatible solvents, making the synthesis of large combinatorial libraries suitable for direct-to-biology screening possible.

With these conditions optimized, a series of building blocks for combinatorial libraries, hereafter referred to as **IrCN-SB**, were selected (Figure 2). The eight pyridine aldehydes included a wide range of functionalities, with electron-withdrawing and electron-donating moieties, a mixture of biaryls, fused rings, and halogens. The 11 amines consisted of both aliphatic and aromatic compounds, with electron-withdrawing and electron-donating functionalities. Of the amines, seven are represented in both **IrCN-Click** and **IrCN-SB** libraries. When combined with the three IrCN building blocks, this represents a substantial library of 264 complexes, covering a wide chemical space. These reactions were set up automatically using an Opentrons OT-2 liquid-handling system, with stock solutions of aldehydes and amines (DMSO) being combined in 700 μL glass vials in a Paradox plate with the IrCN dimers (DMSO), followed by the final addition of water. After heating overnight, the plates were analyzed by LC-MS to obtain conversion rates. Using our custom Python scripts, the 264 LC-MS traces were automatically processed using target complex masses, with the final conversion rates calculated from peak percentage area (254 nm). A similar approach towards the synthesis and analysis was taken with the **IrCN-Click** libraries. The annotated spectra of all crude compounds (552 IrCN compounds and the 96 Tz-4-P ligands) with extracted ion chromatograms, retention times, and integrated peak area percents are provided as Supporting Information to this paper. It should be noted that there are limitations with using the LC-MS absorbance to estimate conversion, namely that the extinction coefficient varies between molecules. As such, certain species may be over- or under-represented by peak area%, and so the following values should be treated as estimates, and not the quantitative conversions. Processed LC-MS spectra for each crude reaction mixture with the extracted ion chromatogram of the target complex can be found as Supporting Information to this article (IrCN_Combined_LCMS_data.pdf).

After approximately 12 months, a selection of four samples from each library was retested by LC-MS, and, for the **IrCN1/2-SB** libraries, the complexes remained intact, demonstrating that hydrolysis of the metalated Schiff base is not an issue in these systems. For **IrCN3-SB**, partial degradation (between −14 and −39% change in conversion) was observed, which points to the electron-withdrawing cyclometalating ligand facilitating hydrolysis over a long period of time (see SI section 5.1). Reasonable stability (between +13 and −22% change in conversion) was observed for the **IrCN1-3-Click** libraries, which are not prone to the same hydrolysis.

### Conversion Rate Analysis

The individual conversion rates based on LC-MS for each target complex are given in Figure 3 (**IrCN-SB**) and Figure 4 (**IrCN-Click**), along with histograms of conversion rates and the mean conversion rate. Generally, in the **IrCN-SB** scope both **IrCN1** and **IrCN2** libraries had good mean conversion rates (61.2 ± 29.5% and 60.9 ± 28.1%, respectively), albeit with high variance and large standard deviations (Figure 5, left). These large errors are due to conversion rates being clustered around very high or very low values, with a relatively small number of reactions having middling values. In comparison, **IrCN3** performed relatively poorly, with an average conversion rate of 41.3 ± 22.2%, and significantly less clustering around the extremes, leading to a narrower distribution. This is probably due to the strong electron-withdrawing and hydrophobic properties of the cyclometallating ligand, which likely leads to poor dimer cleavage by water, disfavoring product formation. Additionally, the poor solubility of both the **IrCN3** dimer and products in the solvent mixture may have affected the observed conversion rates, although samples were aspirated three times before aliquoting to ensure homogeneity. For the **IrCN-Click** library (Figure 5, right), similar trends in conversion rate were observed, although the average conversion rates were consistently higher (**IrCN1**= 68.0 ± 18.2%, **IrCN2** = 64.8 ± 15.1%, **IrCN3** = 53.8 ± 13.0%) and with significantly narrower distributions than the **IrCN-SB** libraries. Again, conversion rates with the **IrCN3** scaffold were notably poorer than the others. The outliers on the lower end on the **IrCN-Click** plots are **M11Y4** (*p*-methyl ester, *m*-bromine) for **IrCN1** and **IrCN3**, and the **M15**-containing ligands (7-aminocephalosporanic acid) for **IrCN2** and **IrCN3**. For **M11Y4** in particular, conversion rates are predetermined by the very low yield of the ligand synthesis, whereas **M5**-containing ligands are universally the poorest at coordinating.

**Figure 3:**
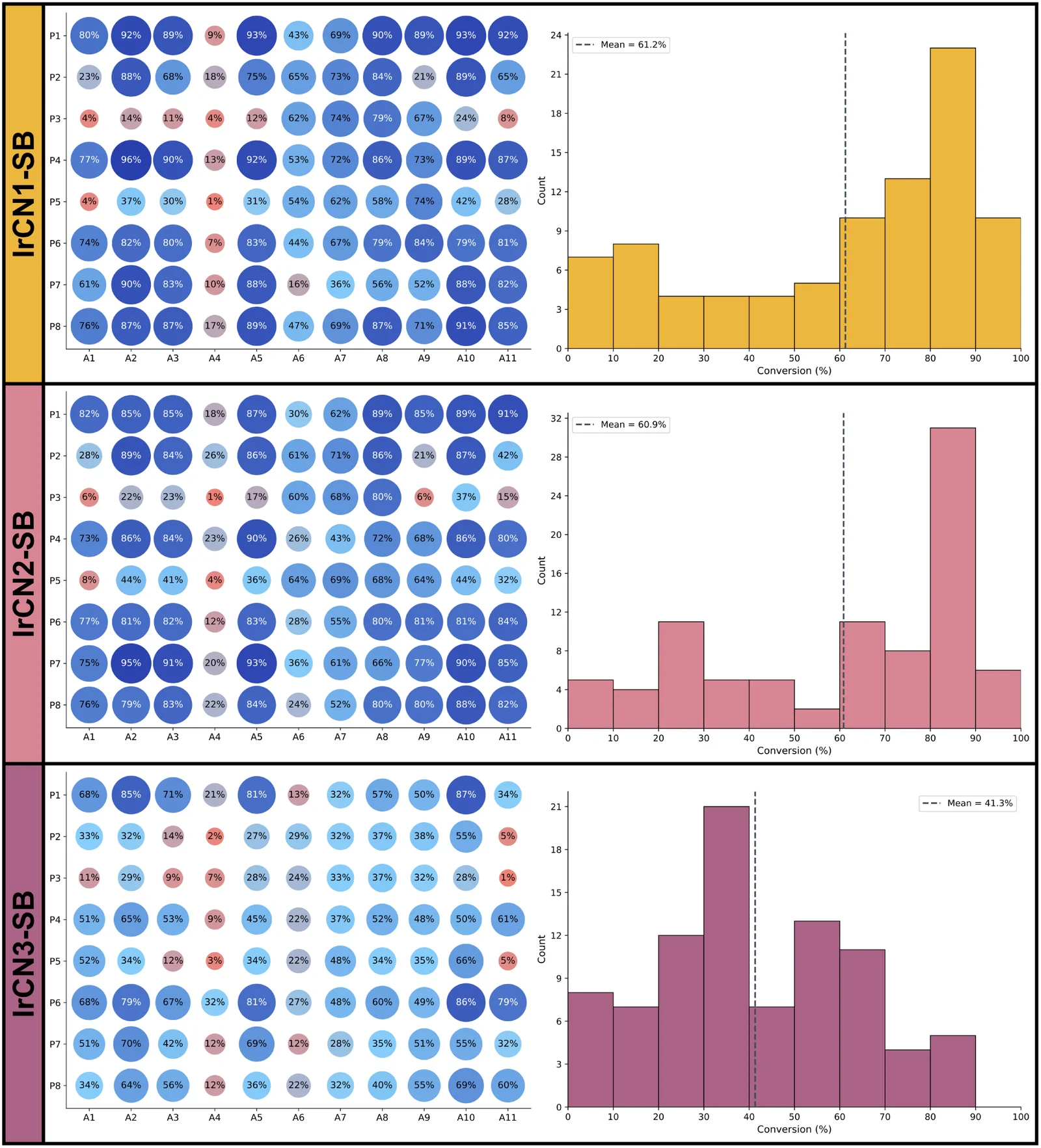
(Left) Conversion rates determined by LC-MS peak area% (254 nm) of the target complex for the **IrCN-SB** libraries; (Right) histograms showing the distribution of conversion rates for each library, with the mean conversion rate.

**Figure 4:**
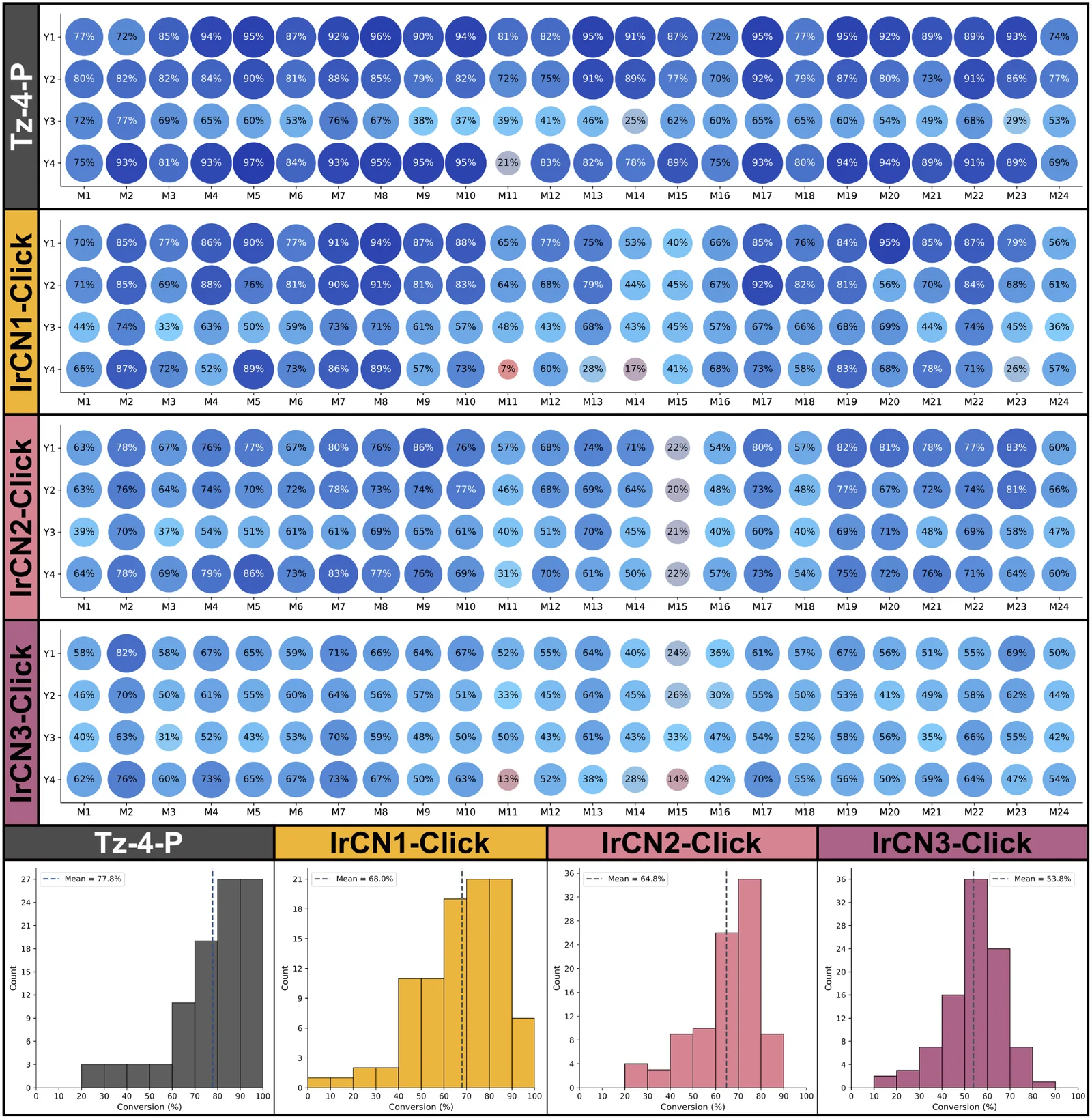
(Top) Conversion rates determined by LC-MS peak area% (254 nm) of the target complex for the **Tz-4-P** library and corresponding **IrCN-Click** libraries; (Bottom) histograms showing the distribution of conversion rates for each library, with the mean conversion rates.

**Figure 5:**
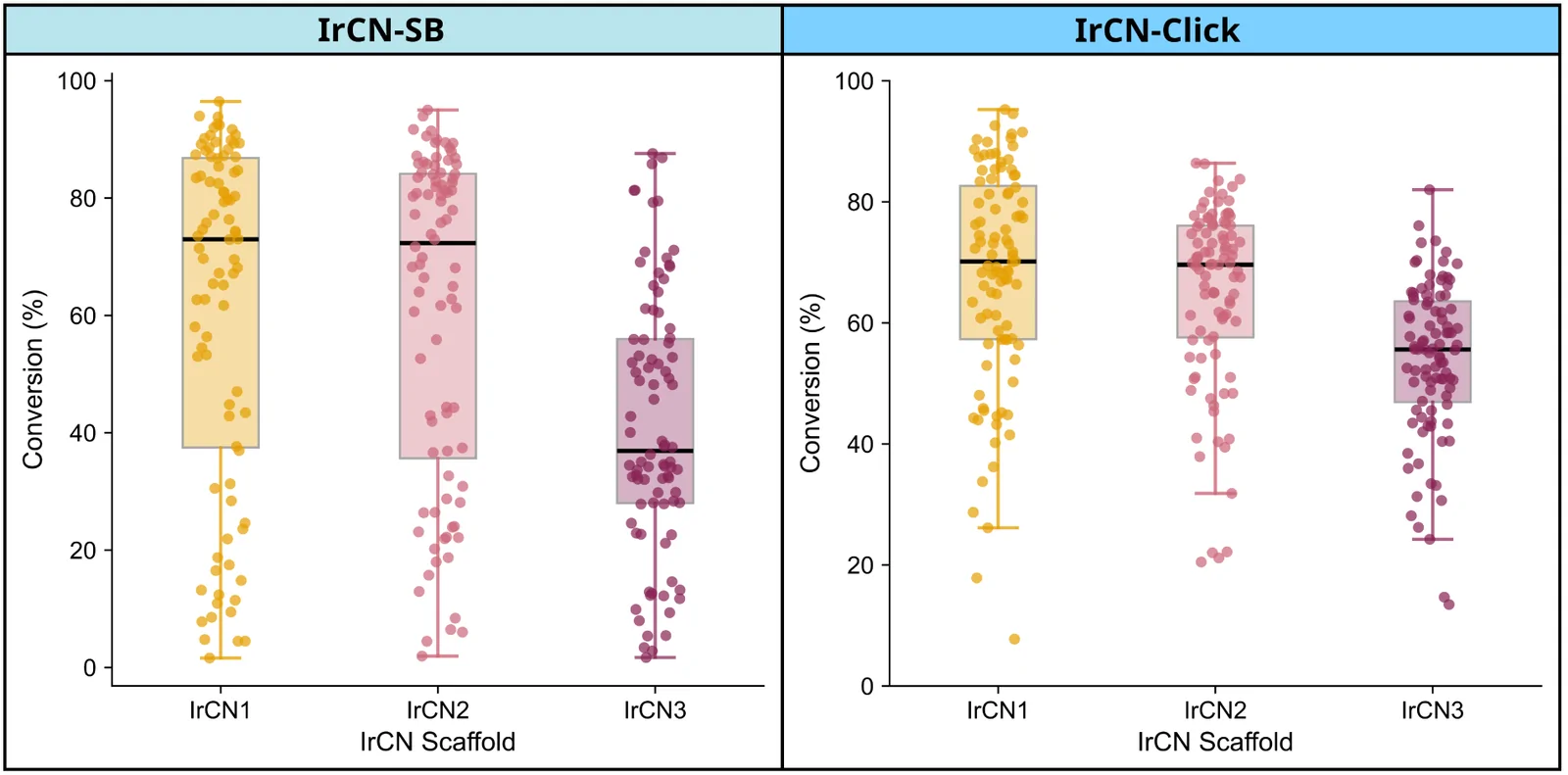
Box and whisker plots for conversion of **IrCN-SB** (Left) and **IrCN-Click** (Right) libraries, categorized by IrCN scaffold.

With regard to the building blocks of the **IrCN-SB** libraries (Figure 6, top), some immediately apparent low conversion rates across all libraries involve amine **A4** (*o*-methyl ester, *p*-methanol) and to a lesser extent **A6** (cyclohexane) and aldehydes **P3** (*o*-piperidine) and **P5** (*o*-Br, *m*-F). **A4** contains a strong electron-withdrawing ester group *ortho* to the amine, which is likely the cause of low conversion rates, reducing the nucleophilicity of the aromatic amine, and providing considerable steric hindrance. For the aldehydes, steric bulk blocking of pyridine coordination from the *ortho* piperidine ring on **P3** and *ortho*-bromine on **P5** is likely responsible for lower conversion rates. However, it should be noted that there is a high level of variance in all of these building blocks, and with the exception of A4, which is a clear outlier, all components have combinations giving either high or low conversions. This means that conversion is a function of the combined structural and electronic effects of the building blocks and not of the individual components. It also means that there are few obvious trends between the conversion and the properties of the building blocks.

**Figure 6:**
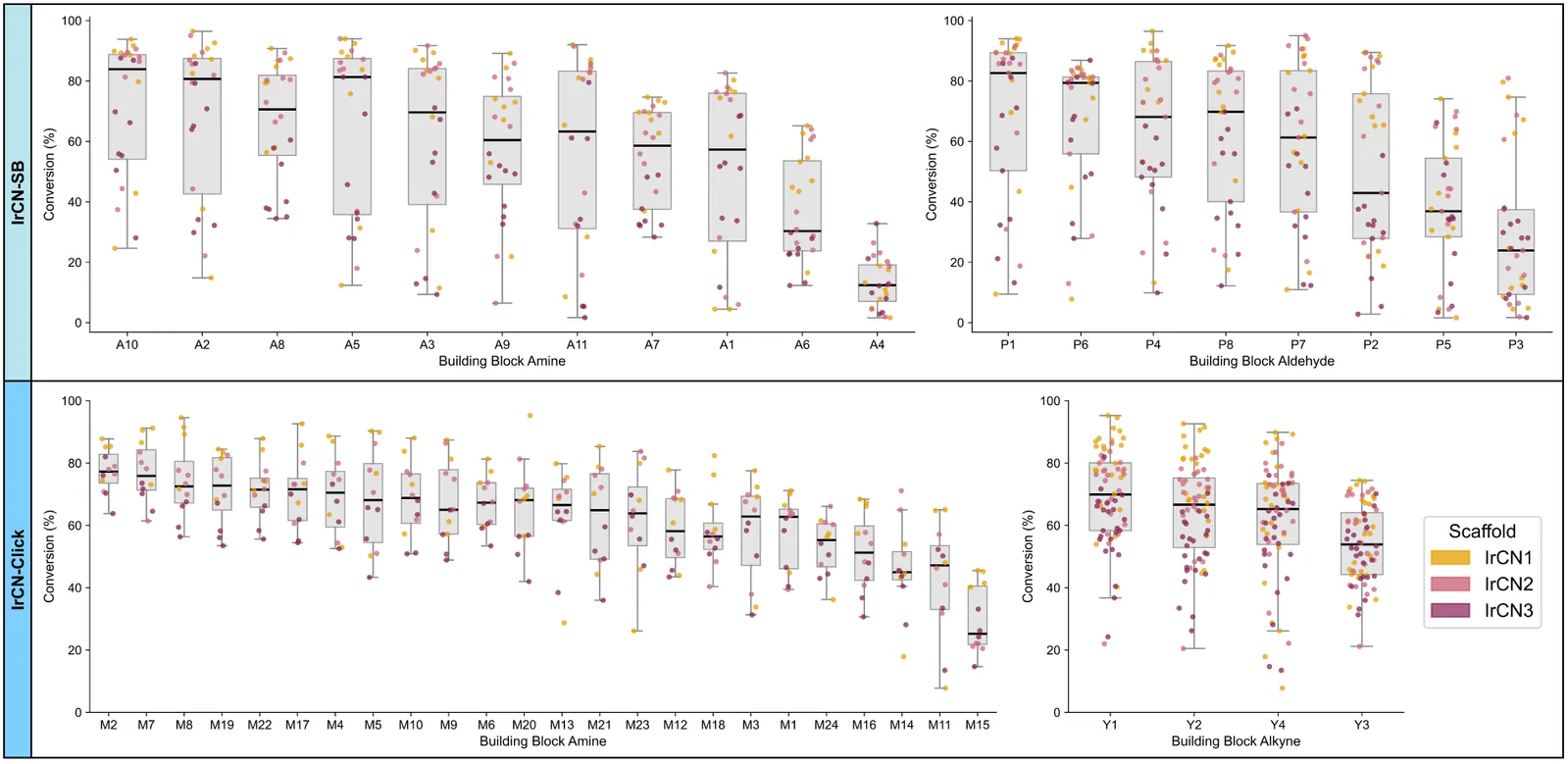
Box and whisker plots for conversion of **IrCN-SB** (Top) and **IrCN-Click** (Bottom) libraries, categorized by building blocks. The building blocks are ordered according to mean conversion rate in descending order.

For the **IrCN-Click** library building blocks (Figure 6, bottom), the only significant outlier for amines in terms of conversion rates across all libraries was the beta-lactam **M15** (7-aminocephalosporanic acid) (29.7 ± 10.8%), with only this and electron-poor **M11** (*p*-methyl ester) having a mean conversion rate below 50%. For the alkynes, **Y3** (*m*-methoxy, 54.1 ± 12.4%) was consistently lower than the other building blocks (**Y1** = 69.2 ± 15.4%, **Y2** = 64.3 ± 15.8%, **Y4** = 61.2 ± 19.1). The same trend of decreasing conversion from **IrCN1–3** was observed for all alkynes, except for **Y4** (*m*-Br), where **IrCN2** had the highest average conversion.

As the **Tz-4-P** library was synthesized prior to complexation with IrCNs, the conversion rates reflect both the ligand and complex formation combined. The highest conversion rates (over 70% for **M2**, **M7**, **M8**, **M17**, **M19**, and **M22**) are all from amines that are either aliphatic or aromatic with only one substituent. This reflects the fact that aliphatic and electron-rich amines are more amenable to the preliminary azide formation step in the SuFEx-click reaction to generate the triazole moiety.^47^ Additionally, a more electron-rich triazole system should coordinate better to the IrCN scaffold.

### Biological Testing

The workflow and selection process for the biological evaluation of compounds in this work was as follows: 1) reasonable purity by LC-MS, 2) structural diversity; 3) high Gram-positive antibacterial activity; 4) compound resynthesis, purification, and confirmation of activity; 5) evaluation of toxicity and other properties. Based on our group’s previous experience with cyclometalated IrCN compounds and other reports, we anticipated that a significant proportion of the synthesized compounds would be highly active against Gram-positive bacteria.^36,44^ As our previous screens of similar IrCN complexes showed no activity against Gram-negative strains, it was decided to limit initial assessment of antibacterial activity to a Gram-positive strain. Rather than testing all libraries in a single-dose response assay at fixed concentrations, we opted for a more detailed screen on subsets of the libraries. For the **IrCN-SB** libraries, we selected 60 compounds with the highest conversion rates (51 higher than 50%) by LC-MS while at the same time taking into account ligand diversity across the three metal scaffolds (**IrCN1–3**, 20 compounds of each), which included some lower conversion compounds. These were run in a minimum inhibitory concentration (MIC—the lowest concentration at which bacterial growth is prevented) assay against *Staphylococcus aureus* (CCUG 62707) as crude mixtures in a “direct-to-biology” approach (see Introduction). Of these 60, half had an MIC ≤6.25 μM against *S. aureus*, demonstrating generally high activity (Figure 7). It is apparent from these data that relatively small changes in chemical structure can dramatically alter the efficacy of metallodrug molecules. The most active ligand combinations were **P1**, **P4**, **A2,** and **A5**, with **P1** and **A2** representing simple phenyl rings. In terms of the scaffolds, **IrCN1** is the most active, with the majority of MICs below 3.13 μM. **IrCN2** and **IrCN3** have similar, but overall lower antibacterial activity, but the broadness of distribution across all scaffolds indicates that the cyclometallating ligand has a reduced effect on activity compared to the Schiff-base ligands (see Supporting Information, Figure S51). Overall, it would appear that less structurally elaborate ligand scaffolds (**IrCN1**, **P1**, **A2**) can result in very high activity against Gram-positive bacteria. One other interesting aspect is that in cases where the Schiff ligand was kept constant, but the IrCN scaffold was varied (Figure 7, **P1A3**, **P1A9**, **P6A9**), significant changes in MIC values were observed. This implies that the combination of building blocks is responsible for antimicrobial activity, but the level of activity is determined by the cyclometallating ligand.

**Figure 7:**
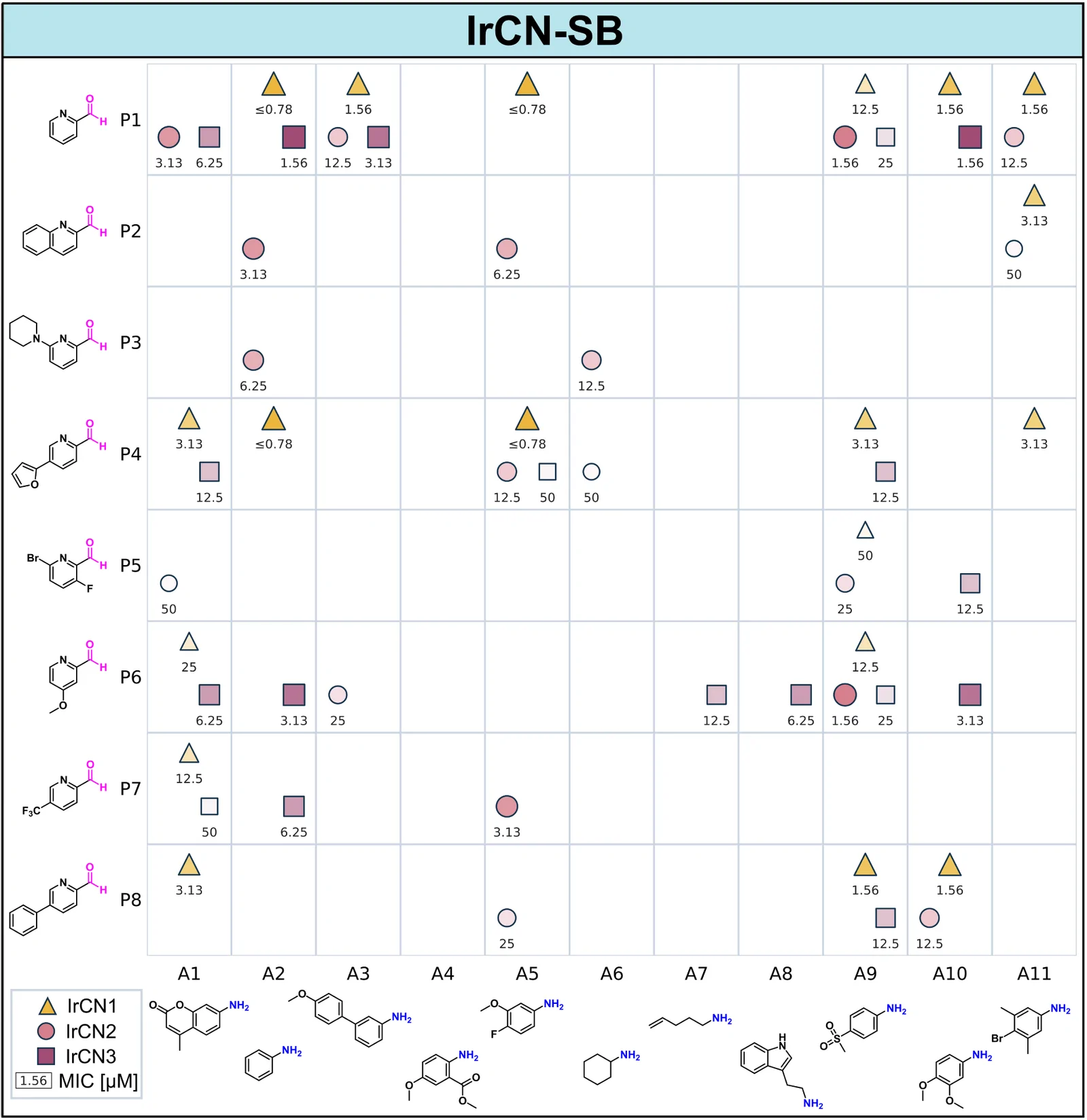
Minimum inhibitory concentrations (MIC) of subsets of crude **IrCN-SB** libraries against *S. aureus*.

For the **IrCN-Click** libraries, a similar approach was taken with 60 crudes, of which 48 displayed a higher than 50% conversion rate (Figure 8). A comparable number of highly active compounds to that in the **IrCN-SB** library was observed (33 with MIC ≤6.25 μM), but there were more compounds with MICs in the nanomolar range (10 **IrCN-Click** vs 4 **IrCN-SB**), with MICs as low as 0.39 μM. Again, **IrCN1** proved to be by far the most active of the scaffolds, while **IrCN3** had a middling distribution but a significant number of MIC values at 50 μM and was generally less active (see Supporting Information, Figure S52). **IrCN2** was more complicated, with a similar number of highly active compounds as **IrCN3**, but more in the mid-to-low-activity region. Similar to the **IrCN-SB** library, the most active ligand combination (0.39 μM) included amines with few substituents **M8** and **M17** in combination with **Y1** and **Y2**, but interestingly **Y4** was present in half of the compounds with high activity (≤0.78 μM). This could suggest that a bromine functionality is key for high activity in this class of compounds.

**Figure 8:**
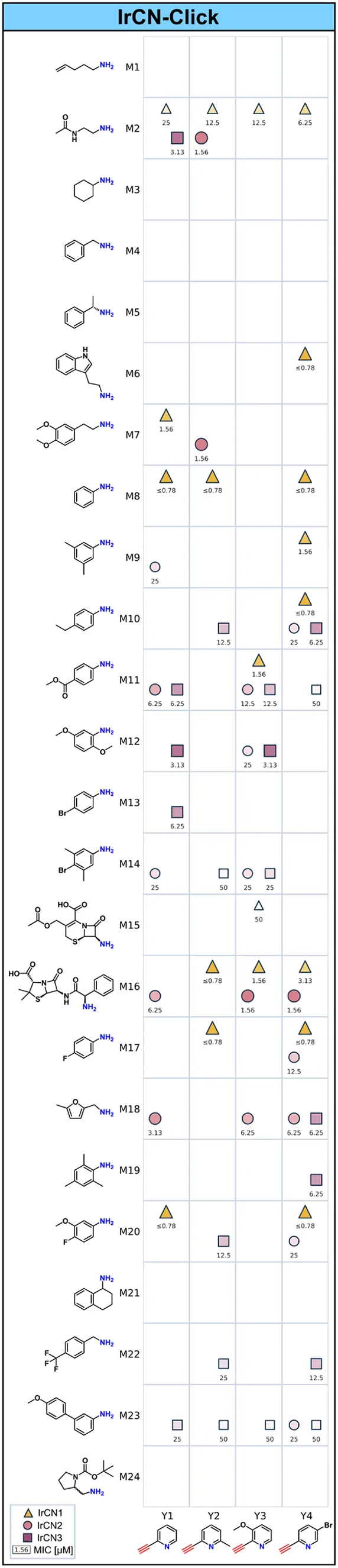
Minimum inhibitory concentrations (MIC) of subsets of crude **IrCN-Click** libraries against *S. aureus*.

Control experiments measuring the MICs of individual reaction components determined that both **IrCN1** and **IrCN2** dimers had an MIC of 100 μM, and the **IrCN3** dimer had an MIC of >100 μM against *S. aureus*. No amine or aldehyde building block in the **IrCN-SB** library showed activity under 100 μM. For the **Tz-4-P** library, previous tests have shown that only **M7Y1** **and M23Y3** (50 μM), and two ampicillin (**M16**)-containing ligands (3.13–12.5 μM) were active on their own.^44^ Coupled with high conversion rates in the combinatorial synthesis, these results allow us to assign the observed activity in biological assays to the putative metal complex with high confidence.

### Hit Validation

Of the tested active compounds, five **IrCN-SB** and three **IrCN-Click** complexes were selected for resynthesis, purification, biological validation, and further exploration (Figure 9). These compounds were picked to represent a variety of different ligands over all three IrCN scaffolds, and all had high conversions and activities (≤6.25 μM MIC), except for **IrCN2­(P3A2)**, which was chosen to account for potential false positives in the initial screening due to low conversion.

**Figure 9:**
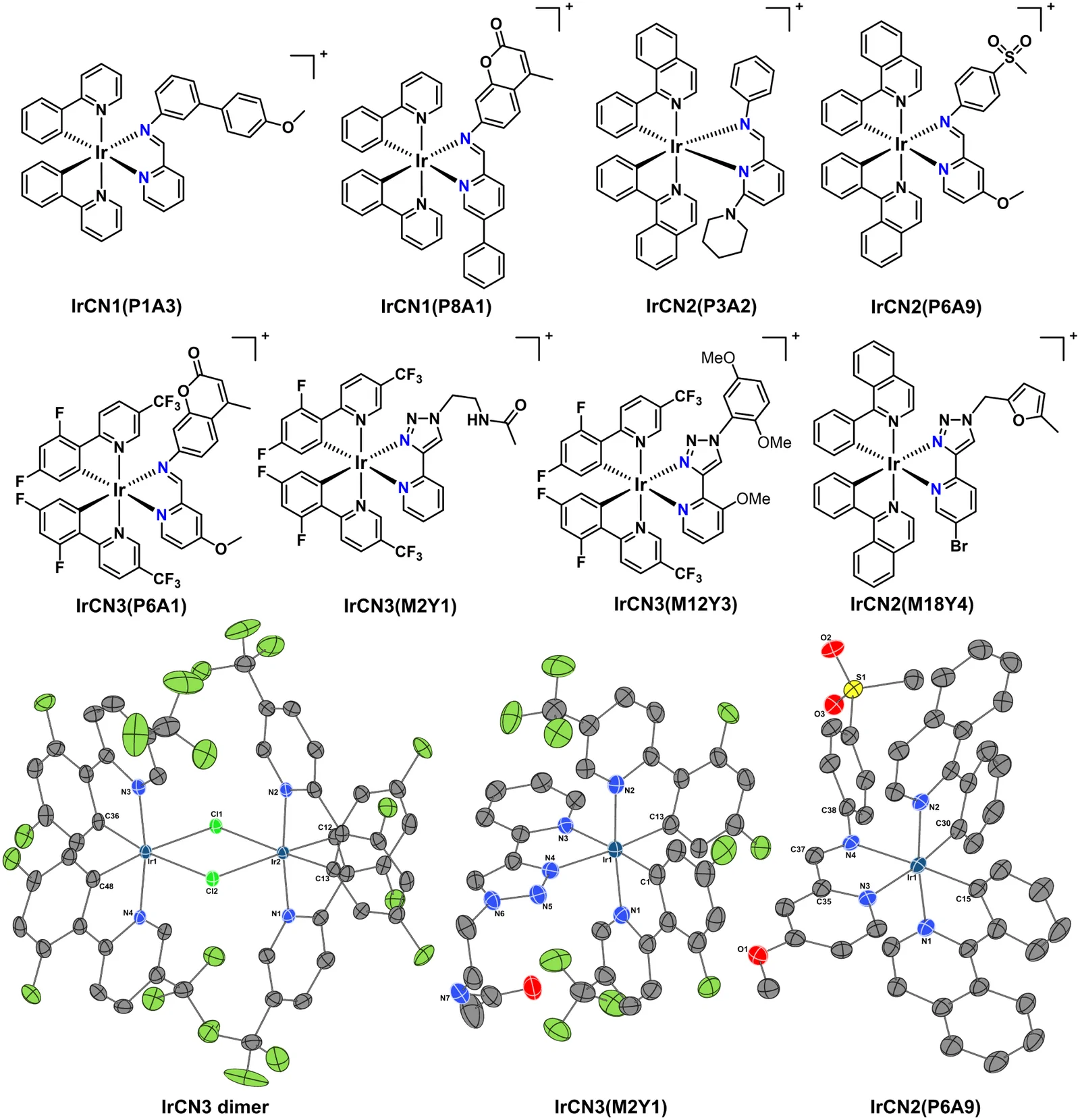
Structures of resynthesized compounds and single-crystal X-ray diffraction structures of **IrCN3** dimer, **IrCN3­(M2Y1)**, and **IrCN2­(P6A9)** (thermal ellipsoids set at 50% probability and hydrogens are not represented for clarity. **IrCN3** dimer pictured without lattice dichloromethane. The counterions of **IrCN3­(M2Y1)** (Cl^–^) and **IrCN2­(P6A9)** (PF_6_^–^) are not shown for clarity.

Synthesis of the pure **IrCN-SB** compounds was achieved under mild conditions by stirring the cyclometalated dimer with 2 equivalents of amine and aldehyde in a 1:1 mixture of DCM:MeOH, similar to the established methods.^35,38,40,50^ This chemistry can be done under air and at room temperature, giving the target complex in average-to-good yields (40–92%). A similar synthetic approach was taken with the **IrCN-Click** complexes, using 2 equivalents of the presynthesized ligand in DCM:MeOH (3:1) at 40 °C. The complexes were purified by column chromatography utilizing DCM with an increasing percentage of methanol. Single crystals suitable for X-ray diffraction were obtained for the **IrCN3** dimer by slow evaporation of DCM, with **IrCN3­(M2Y1)** and the hexafluorophosphate salt of **IrCN2­(P6A9)** crystallizing by slow vapour diffusion of diethyl ether into DCM (Figure 9).

Routine stability studies (UV–Vis and corresponding LC-MS stability in DMSO and DMSO/PBS mixtures, see Supporting Information, Section 4) of the compounds showed that **IrCN2­(P3A2)** degraded in water and water-DMSO combinations, reforming the **IrCN2** dimer. Moreover, **IrCN2­(M18Y4)** proved to be unstable on silica during purification and contained unidentifiable impurities by LC-MS. An alternative method of preparation involving purification by precipitation with KPF_6_ gave a purer complex, but this was shown to be highly unstable in solution (in both coordinating DMSO and non-coordinating DCM) and demonstrated acid sensitivity (1% formic acid). As such, **IrCN2­(M18Y4)** was discounted for biological studies.

All resynthesized compounds were tested in MIC assays against a panel of Gram-positive bacteria, including the original *S. aureus* strain. For the majority of compounds, there was either no change or an increase in activity compared to the crudes, validating the direct-to-biology approach (Table 1). Generally, the measured MICs were in the low micromolar range across the panel of bacteria, with **IrCN2­(P6A9)** and **IrCN3­(M12Y3)** in particular displaying consistently high activities comparable to those of the standard-of-care antibiotic vancomycin. Conversely, **IrCN2­(P3A2)** was not tested further due to the issues with stability, even though the compound seemed to be more active than its respective crude (which had a conversion of only 22% by LC-MS). All compounds showed no activity against Gram-negative strains of *E. coli* or *A. baumannii*, and MIC against *S. aureus* with photoirradiation at 405 nm provided no significant increase in activity, in line with previously reported results for cyclometalated Ir­(III) complexes.^36^

**Table 1:** Minimum Inhibitory Concentration (MIC) Values for the Pure Compounds against a Panel of Gram-Positive Bacteria Strains^a^

	MIC [μM]					Toxicity [μM]	
	*S. aureus* CCUG 62707		*Enterococcus faecium* CCUG 19434	*Enterococcus faecalis* CCUG 19916T	*Bacillus subtilis* 168CA	HEK293T	
**Compound**	**Crude**	**Pure**				**CC**_**50**_	**TI**
**IrCN1(P1A3)**	1.56	1.56	3.13	1.56	1.56	3.30 ± 0.39	2.1
**IrCN1(P8A1)**	3.13	3.13	6.25	3.13–6.25	3.13	3.61 ± 0.36	1.2
**IrCN2(P6A9)**	1.56	1.56	3.13	1.56	3.13	7.75 ± 0.69	5.0
**IrCN3(P6A1)**	6.25	3.13	6.25	3.13	3.13	13.81 ± 1.35	4.4
**IrCN3(M2Y1)**	3.13	3.13	25	12.5	3.13	13.83 ± 1.15	4.4
**IrCN3(M12Y3)**	3.13	3.13	3.13	3.13	3.13	2.27 ± 0.32	0.7
*Vancomycin*	0.86	1.73	0.86	0.43	0.43	n.d.	n.d.

a CC_50_ (HEK293T cells): the concentration at which 50% of the cells remain viable; the error recorded is the standard deviation. TI: therapeutic index , determined by dividing the CC_50_ value by the MIC value for each compound against *S. aureus*; MIC was determined with N = 3 technical replicates across two biological replicates . The median value for MIC is used. N.D. = not determined .

The remaining six compounds were tested for cytotoxicity against HEK293T cells in a dose-dependent MTT assay to obtain CC_50_ values (the concentration at which 50% of cells remain viable).^51^ These values can be used to determine the therapeutic index (TI), a simple measure of the selectivity, and hence, the potential of a given compound as an antibacterial agent. This can be calculated by dividing the CC_50_ value by the MIC value of the complex, meaning that toxicity can be offset by high activity against bacteria. Compound **IrCN2­(P6A9)** had a TI of up to 5 across multiple bacteria strains, and **IrCN3­(P6A1)** displayed a TI of 4.4 against *S. aureus*, alongside having high activity against other strains. Generally, the **IrCN1** compounds tended to be more toxic than **IrCN2** and **IrCN3**, although toxicity (and indeed activity) can be greatly affected by small changes in the coordinating ligands. Hence, both **IrCN2­(P6A9)** and **IrCN3­(P6A1)** show promise as starting points for further derivatization on these scaffolds for more effective antibiotics.

## Conclusions

In summary, we have presented a new methodology for the facile combinatorial synthesis of large libraries of cyclometalated iridium compounds utilizing bidentate Schiff-base and pyridine-triazole ligands. The coordination of these ligands has been optimized to proceed under mild conditions with high conversions in biologically compatible solvents and applied to the synthesis of 552 metal complexes in high throughput. We have shown that varying the cyclometalating ligand can affect both reaction conversion and biological efficacy, and small chemical changes in building blocks can have a significant impact on antimicrobial activity. We have demonstrated that the biologically relevant synthetic conditions for these libraries can be exploited in a direct-to-biology approach to screen rapidly for highly active compounds. Additionally, batch synthesis under ambient mild conditions of a number of compounds of interest has been reported, and the biological assessment of eight resynthesized compounds has validated direct-to-biology screening. The identification of two compounds that could be either investigated further or used as a starting point for further derivatization and screening has demonstrated the application of this approach and associated ligand chemistry.

## Experimental Section

Reagents were purchased from Merck, Fluorochem, Alfa Aesar, and Fisher and used without further purification. The purity of chemicals was confirmed by LC-MS before use. The synthesis of **IrCN1–3** dimers was carried out according to a method from Kench et al.^35^ The preparation of the **Tz-4-P** ligand library was done according to our previously reported method.^44^ NMR assignments of ^1^H, ^13^C, and ^19^F spectra have been provided in the associated Supporting Information.

Synthesis of the **IrCN-SB** libraries was undertaken using an Opentrons OT2 robotic platform with a 300 μL pipette. Paradox metal plates containing 96 × 700 μL glass vials were used, with each as a designated well. To each designated well, the amine (90 μL, 20 mM in DMSO), aldehyde (90 μL, 20 mM in DMSO), and IrCN dimer (90 μL, 10 mM in DMSO) were added, followed by 180 μL of H_2_O. The plate was sealed and heated for 18 h using an Eppendorf Thermomixer (60 °C, 600 rpm). The conversion of each well was measured by LC-MS (254 nm). Each well contained 450 μL of 4 mM stock in a 3:2 DMSO:H_2_O mixture (assuming 100% conversion to product).

Synthesis of the **IrCN-Click** libraries was undertaken using an Opentrons OT2 robotic platform with a 300 μL pipet. Paradox metal plates containing 96 × 700 μL glass vials were used, with each as a designated well. To each designated well, the triazole ligand (90 μL, 20 mM in DMSO) and IrCN dimer (90 μL, 10 mM in DMSO) were added, followed by 180 μL of H_2_O. The plate was sealed and heated for 18 h using an Eppendorf Thermomixer (60 °C, 600 rpm). For IrCN1, each well contained 360 μL of 5 mM stock in a 1:1 DMSO:H_2_O mixture (assuming 100% conversion to product). For **IrCN2**, the reaction was not complete by LC-MS, so a further 90 μL of DMSO was added to each well, the plate was sealed and heated for a further 18 h at 60 °C (600 rpm). An additional 90 μL of DMSO was then added to each well, so that each well contained 540 μL of 3.3 mM stock in a 2:1 DMSO:H_2_O mixture (assuming 100% conversion to product). For **IrCN3**, the reaction was not complete by LC-MS, so a further 180 μL of DMSO was added to each well, the plate was sealed and heated for a further 18 h at 65 °C (600 rpm). Each well contained 540 μL of 3.3 mM stock in a 2:1 DMSO:H_2_O mixture (assuming 100% conversion to product).

### General Procedure for Batch Synthesis of Click Ligands

FSO_2_N_3_ was synthesized^47^ and diluted with DMF to approximately 200 mM in 1:1 MTBE:DMF. The desired amine (0.56 mmol, 1.0 equiv.) was charged into a 50 mL polypropylene Falcon tube, followed by DMF (7 mL). Sat. KHCO_3_ solution (0.8 mL, approximately 2.4 mmol, 4 equiv. in water) was added, followed by FSO_2_N_3_ solution (1.05 equiv.). The resulting mixture was stirred at r.t. for 18 h. Sodium ascorbate solution (5.6 mL, 50 mM, 0.5 equiv. in water) was added, and the mixture was stirred for a further 10 min. Then, 2 mL of DMF was added, followed by alkyne (0.56 mmol, 1.0 equiv), then (BimH)_3_ solution (0.7 mL, 20 mM, 2.5 mol % in DMF) and CuSO_4_ solution (0.7 mL, 20 mM, 2.5 mol % in water). The resulting mixture was stirred for a further 18 h at r.t. The crude reaction mixture was analyzed by LC-MS, then 100 mL of deionized water was added. The mixture was extracted with EtOAc (5 × 20 mL) (and for M2Y1, additional 3:1 CHCl_3_:isopropyl alcohol (3 × 20 mL). The combined organic layers were washed with brine (5 × 25 mL), dried (MgSO_4_), filtered, and the solvent evaporated. Toluene (2 × 25 mL) was added to the residue, and the solvent removed in vacuo to remove residual DMF. Purification was achieved by flash column chromatography (Combiflash, 12g SiO_2_ cartridges. All compounds were loaded “wet” by dissolution in the minimum volume of EtOAc or DCM).

**M2Y1** Column chromatography in MeOH/DCM (0%–10% gradient), eluting at 7% MeOH. Cream solid, 63 mg (49% yield), R_f_ = 0.39 (10% MeOH in DCM); ^1^H NMR (400 MHz, C*D*Cl_3_) δ 8.54 (ddd, *J* = 4.9, 1.8, 0.9 Hz, 1H), 8.12 (s, 1H), 8.06 (dt, *J* = 8.0, 1.1 Hz, 1H), 7.75 (td, *J* = 7.7, 1.8 Hz, 1H), 7.22 (ddd, *J* = 7.6, 4.9, 1.2 Hz, 1H), 6.38 (s, 1H), 4.58–4.50 (m, 2H), 3.86–3.76 (m, 2H), 1.98 (s, 3H); ^13^C NMR (101 MHz, C*D*Cl_3_) δ 170.9, 150.0, 149.5, 148.5, 137.1, 123.1, 123.0, 120.3, 50.0, 39.6, 23.3; HRMS (ESI+) (C_11_H_13_N_5_NaO)^+^ *m*/*z* (calculated) 254.1012, (found) 254.1015, mass difference 1.1 ppm; LC-MS retention time (ESI+, 7 min method, H_2_O:MeCN 0.1% formic acid): 0.67 min, *m*/*z* 232.09; 0.98 min, *m*/*z* 232.05.

**M12Y3** Column chromatography in MeOH/DCM (0%–5% gradient), eluting at 3% MeOH. Brown oil (solvato-chromic compound), 84 mg (48% yield), R_f_ = 0.30 (100% EtOAc); ^1^H NMR (400 MHz, C*D*Cl_3_) δ 8.67 (s, 1H), 8.39 (d, *J* = 4.5 Hz, 1H), 7.41 (d, *J* = 3.0 Hz, 1H), 7.30 (d, *J* = 8.3 Hz, 1H), 7.25 (d, *J* = 4.6 Hz, 1H), 7.00 (d, *J* = 9.1 Hz, 1H), 6.94 (dd, *J* = 9.2, 3.0 Hz, 1H), 3.95 (s, 3H), 3.81 (s, 3H), 3.80 (s, 3H); ^13^C NMR (101 MHz, C*D*Cl_3_) δ 154.0, 152.7, 145.4, 143.6, 141.8, 139.4, 126.8, 123.2, 118.3, 115.8, 113.9, 110.9, 56.8, 56.1, 55.7; HRMS (ESI+) (C_16_H_16_N_4_NaO_3_)^+^ *m*/*z* (calculated) 335.1115, (found) 335.1117, mass difference 0.6 ppm; LC-MS retention time (ESI+, 7 min method, H_2_O:MeCN 0.1% formic acid): 2.57 min, *m*/*z* 313.11.

**M18Y4** Column chromatography in EtOAc/Hexane (0%–40% gradient), eluting at 23% EtOAc. White solid, 143 mg (80% yield), R_f_ = 0.53 (40% EtOAc); ^1^H NMR (400 MHz, C*D*Cl_3_) δ 8.60 (dd, *J* = 2.3, 0.6 Hz, 1H), 8.12 (s, 1H), 8.07 (dt, *J* = 8.5, 0.6 Hz, 1H), 7.88 (dd, *J* = 8.5, 2.4 Hz, 1H), 6.37 (d, *J* = 3.1 Hz, 1H), 5.96 (dd, *J* = 3.2, 1.2 Hz, 1H), 5.51 (s, 2H), 2.27 (s, 3H); ^13^C NMR (101 MHz, C*D*Cl_3_) δ 154.0, 150.5, 148.9, 147.7, 145.1, 139.7, 122.1, 121.6, 119.6, 111.8, 107.0, 47.2, 13.7; HRMS (ESI+) (C_13_H_11_BrN_4_NaO)^+^ *m*/*z* (calculated) 341.0008, (found) 341.0009, mass difference 0.2 ppm; LC-MS retention time (ESI+, 7 min method, H_2_O:MeCN 0.1% formic acid): 3.73 min, *m*/*z* 318.98;

### General Procedure for Batch Synthesis of IrCN-Schiff Complexes

To a 7 mL glass vial, IrCN dimer (1 equiv), aldehyde (2 equiv) and amine (2 equiv) were added. DCM (1.5 mL) and MeOH (1.5 mL) were added, and the resulting solution was stirred at room temperature overnight. LC-MS of the reaction mixture was taken to confirm product formation, then the solvent was removed *in vacuo*. The residue was purified by flash column chromatography (Combiflash, 12 g SiO_2_ cartridge, 0–15% MeOH in DCM over 25 min). The sample was loaded “wet” in DCM (approximately 2 mL) to obtain the pure compound.

**IrCN1­(P1A3)** Red solid, 45 mg (73% yield); ^1^H NMR (700 MHz, CD_2_Cl_2_) δ 10.58 (s, 1H), 9.30 (d, *J* = 7.6 Hz, 1H), 8.40 (d, *J* = 6.3 Hz, 1H), 8.14 (t, *J* = 7.3 Hz, 1H), 7.95 (dt, *J* = 8.2, 1.0 Hz, 1H), 7.87 (dd, *J* = 5.4, 1.4 Hz, 1H), 7.83 (tt, *J* = 7.2, 1.0 Hz, 2H), 7.81–7.77 (m, 1H), 7.71 (dd, *J* = 7.9, 1.3 Hz, 1H), 7.53 (dd, *J* = 7.9, 1.3 Hz, 1H), 7.51–7.49 (m, 1H), 7.45 (dd, *J* = 7.7, 5.4 Hz, 1H), 7.36 (dt, *J* = 6.5, 1.8 Hz, 1H), 7.23–7.17 (m, 5H), 7.16 (ddd, *J* = 7.4, 5.8, 1.4 Hz, 1H), 7.09–7.03 (m, 2H), 6.93–6.85 (m, 4H), 6.78 (td, *J* = 7.4, 1.3 Hz, 1H), 6.22 (ddd, *J* = 7.6, 2.7, 1.2 Hz, 2H), 3.82 (s, 3H); ^13^C NMR (176 MHz, CD_2_Cl_2_) δ 170.4, 168.2, 167.6, 160.0, 157.0, 151.1, 150.9, 150.5, 149.3, 148.8, 148.6, 144.0, 143.7, 142.0, 139.8, 138.6, 138.6, 133.2, 132.3, 132.0, 131.6, 131.0, 130.7, 129.8, 129.7, 128.7, 127.4, 125.1, 124.9, 124.2, 123.6, 123.3, 122.4, 122.2, 121.3, 120.2, 119.9, 114.4, 46.1; HRMS (ESI+) (C_41_H_32_IrN_4_O)^+^ *m*/*z* (calculated) 789.2200, (found) 789.2239, mass difference 4.7 ppm; LC-MS retention time (ESI+, 7 min method, H_2_O:MeCN 0.1% formic acid): 3.25 min, *m*/*z* 789.37.

**IrCN1­(P8A1)** Dark red solid, 60 mg (92% yield); ^1^H NMR (700 MHz, CD_2_Cl_2_) δ 10.77 (s, 1H), 9.37 (d, *J* = 8.1 Hz, 1H), 8.46 (d, *J* = 5.9 Hz, 1H), 8.33 (dd, *J* = 8.1, 2.1 Hz, 1H), 8.11 (d, *J* = 2.1 Hz, 1H), 7.98 (d, *J* = 8.2 Hz, 1H), 7.83 (dddd, *J* = 19.7, 15.3, 8.0, 1.5 Hz, 3H), 7.75 (dd, *J* = 7.9, 1.4 Hz, 1H), 7.59 (d, *J* = 5.7 Hz, 1H), 7.50 (d, *J* = 7.8 Hz, 1H), 7.46–7.39 (m, 4H), 7.35 (ddd, *J* = 7.0, 5.1, 2.8 Hz, 3H), 7.22 (ddd, *J* = 7.3, 5.8, 1.4 Hz, 1H), 7.12–7.06 (m, 2H), 6.99 (d, *J* = 2.1 Hz, 1H), 6.94 (td, *J* = 7.5, 1.3 Hz, 1H), 6.90 (td, *J* = 7.5, 1.3 Hz, 1H), 6.85 (td, *J* = 7.4, 1.4 Hz, 1H), 6.27 (ddd, *J* = 7.2, 5.7, 1.2 Hz, 2H), 6.16 (q, *J* = 1.4 Hz, 1H), 2.32 (d, *J* = 1.4 Hz, 3H); ^13^C NMR (176 MHz, CD_2_Cl_2_) δ 171.6, 168.1, 167.5, 160.3, 155.2, 153.6, 152.2, 151.3, 150.4, 150.3, 149.4, 148.7, 148.5, 144.0, 143.6, 142.6, 138.7, 138.7, 137.0, 135.0, 133.7, 132.0, 131.7, 131.1, 130.8, 130.4, 129.9, 127.5, 125.8, 125.2, 124.9, 124.4, 123.7, 123.4, 122.8, 120.3, 120.3, 119.9, 119.3, 115.7, 112.6, 18.8; HRMS (ESI+) (C_44_H_32_IrN_4_O_2_)^+^ *m*/*z* (calculated) 841.2149, (found) 841.2172, mass difference 2.4 ppm; LC-MS retention time (ESI+, 7 min method, H_2_O:MeCN 0.1% formic acid): 3.20 min, *m*/*z* 841.38.

**IrCN2­(P3A2)** Red solid, 20 mg (40% yield); ^1^H NMR (700 MHz, CD_2_Cl_2_) δ 9.80 (s, 1H), 8.97–8.91 (m, 1H), 8.70 (d, *J* = 8.6 Hz, 1H), 8.22 (dd, *J* = 11.1, 7.2 Hz, 2H), 8.14 (d, *J* = 6.3 Hz, 1H), 8.03–7.97 (m, 2H), 7.96–7.91 (m, 1H), 7.84–7.77 (m, 4H), 7.74 (ddd, *J* = 8.5, 7.0, 1.4 Hz, 1H), 7.68 (dd, *J* = 8.7, 7.1 Hz, 1H), 7.56 (d, *J* = 6.3 Hz, 1H), 7.40 (d, *J* = 6.3 Hz, 1H), 7.02 (ddd, *J* = 8.1, 7.0, 1.3 Hz, 1H), 6.97–6.91 (m, 1H), 6.87 (ddd, *J* = 8.2, 7.1, 1.3 Hz, 1H), 6.79 (t, *J* = 7.9 Hz, 2H), 6.75 (d, *J* = 8.7 Hz, 1H), 6.72 (td, *J* = 7.4, 1.3 Hz, 1H), 6.67 (td, *J* = 7.4, 1.3 Hz, 1H), 6.52–6.48 (m, 2H), 6.40 (dd, *J* = 7.7, 1.3 Hz, 1H), 6.36 (dd, *J* = 7.8, 1.3 Hz, 1H), 2.93 (ddd, *J* = 13.3, 7.6, 3.5 Hz, 2H), 2.55 (ddd, *J* = 13.3, 7.5, 3.5 Hz, 2H), 1.32 (p, *J* = 5.8 Hz, 2H), 1.24–1.17 (m, 2H), 1.14 (ddp, *J* = 10.9, 7.5, 3.6 Hz, 2H); ^13^C NMR (176 MHz, CD_2_Cl_2_) δ 170.9, 169.7, 169.0, 164.2, 155.6, 153.4, 149.2, 147.4, 146.0, 144.8, 142.4, 141.1, 139.6, 137.4, 137.3, 134.2, 132.4, 132.2, 132.1, 130.6, 130.3, 130.0, 129.2, 129.0, 129.0, 128.6, 127.9, 127.8, 127.8, 127.2, 126.8, 126.3, 126.2, 123.6, 122.1, 122.0, 121.9, 121.6, 121.4, 119.0, 51.9, 24.6, 24.3; HRMS (ESI+) (C_47_H_39_IrN_5_)^+^ *m*/*z* (calculated) 866.2829, (found) 866.2832, mass difference 0.0 ppm; LC-MS retention time (ESI+, 7 min method, H_2_O:MeCN 0.1% formic acid): 3.92 min, *m*/*z* 866.38.

**IrCN2­(P6A9)** Red solid, 29 mg (57% yield); ^1^H NMR (700 MHz, CD_2_Cl_2_) δ 10.70 (s, 1H), 9.36–9.30 (m, 1H), 8.97 (dq, *J* = 7.9, 0.9 Hz, 1H), 8.70 (d, *J* = 8.6 Hz, 1H), 8.33–8.26 (m, 2H), 8.02–7.93 (m, 3H), 7.86–7.78 (m, 3H), 7.75 (ddd, *J* = 8.4, 6.9, 1.4 Hz, 1H), 7.57 (d, *J* = 6.2 Hz, 1H), 7.50–7.44 (m, 5H), 7.12 (ddd, *J* = 8.2, 7.2, 1.3 Hz, 1H), 7.10–7.04 (m, 2H), 6.96–6.89 (m, 2H), 6.87 (td, *J* = 7.5, 1.3 Hz, 1H), 6.71 (td, *J* = 7.4, 1.3 Hz, 1H), 6.26 (dd, *J* = 7.7, 1.3 Hz, 1H), 6.12 (dd, *J* = 7.7, 1.3 Hz, 1H), 4.09 (s, 3H), 2.89 (s, 3H). ^13^C NMR (176 MHz, CD_2_Cl_2_) δ 172.4, 169.5, 168.9, 168.5, 158.2, 153.5, 152.0, 151.7, 151.0, 145.8, 145.6, 142.2, 140.8, 140.1, 137.5, 137.4, 132.5, 132.3, 132.3, 132.2, 131.1, 131.0, 130.8, 130.7, 129.2, 129.2, 128.3, 127.9, 127.8, 127.2, 126.8, 126.5, 124.0, 122.8, 122.5, 122.3, 122.2, 119.9, 116.7, 57.8, 44.8. HRMS (ESI+) (C_44_H_34_IrN_4_O_3_S)^+^ *m*/*z* (calculated) 891.1975, (found) 891.2011, mass difference 3.9 ppm; LC-MS retention time (ESI+, 7 min method, H_2_O:MeCN 0.1% formic acid): 3.24 min, *m*/*z* 891.34.

**IrCN3­(P6A1)** Orange solid, 35 mg (63% yield); ^1^H NMR (700 MHz, CD_2_Cl_2_) δ 10.83 (s, 1H), 9.39 (d, *J* = 2.9 Hz, 1H), 8.61 (s, 1H), 8.49 (dd, *J* = 8.8, 2.8 Hz, 1H), 8.40 (dd, *J* = 8.8, 2.8 Hz, 1H), 8.13 (ddd, *J* = 8.4, 5.9, 2.1 Hz, 2H), 7.70 (s, 1H), 7.62 (d, *J* = 6.3 Hz, 1H), 7.52 (d, *J* = 8.5 Hz, 1H), 7.29 (dd, *J* = 8.5, 2.2 Hz, 1H), 7.06 (dd, *J* = 6.3, 2.8 Hz, 1H), 6.80 (d, *J* = 2.2 Hz, 1H), 6.66 (ddd, *J* = 12.4, 8.9, 2.3 Hz, 1H), 6.50 (ddd, *J* = 12.5, 9.0, 2.3 Hz, 1H), 6.22 (q, *J* = 1.3 Hz, 1H), 5.62 (dd, *J* = 8.1, 2.3 Hz, 1H), 5.58 (dd, *J* = 8.2, 2.3 Hz, 1H), 4.14 (s, 3H), 2.36 (d, *J* = 1.4 Hz, 3H); ^19^F NMR (659 MHz, CD_2_Cl_2_) δ −62.46 (s, 3F), −62.89 (s, 3F), −102.58 to −102.70 (m, 1F), −102.87 to −102.98 (m, 1F), −106.46 (dd, *J* = 12.6, 3.5 Hz, 1F), −106.82 to −106.96 (m, 1F); ^13^C NMR (176 MHz, CD_2_Cl_2_) δ 168.3 (d, *J* = 6.6 Hz), 167.8 (d, *J* = 6.5 Hz), 165.7 (d, *J* = 12.7 Hz), 164.2 (d, *J* = 12.6 Hz), 163.5 (d, *J* = 13.1 Hz), 162.1–161.9 (m), 159.9, 158.2, 155.1 (d, *J* = 7.0 Hz), 153.8, 152.5 (d, *J* = 7.2 Hz), 150.8, 149.6, 147.8 (t, *J* = 4.5 Hz), 146.1 (d, *J* = 4.7 Hz), 137.3, 137.1 (d, *J* = 3.4 Hz), 127.0, 126.8, 126.7, 126.4, 126.3, 124.2, 124.1, 124.0, 123.9, 123.0 (d, *J* = 14.0 Hz), 121.5 (d, *J* = 13.8 Hz), 121.0, 120.8, 118.8, 117.5, 116.3, 114.5 (d, *J* = 17.4 Hz), 114.3 (d, *J* = 19.4 Hz), 112.0, 100.3 (t, *J* = 26.7 Hz), 99.7 (t, *J* = 26.7 Hz), 58.1, 18.8; HRMS (ESI+) (C_41_H_24_F_10_IrN_4_O_3_)^+^ *m*/*z* (calculated) 1003.1312, (found) 1003.1335, mass difference 2.0 ppm; LC-MS retention time (ESI+, 7 min method, H_2_O:MeCN 0.1% formic acid): 3.38 min, *m*/*z* 1003.30.

### Batch Synthesis of IrCN-Click Complexes

**IrCN3­(M2Y1)** To a 20 mL glass vial, IrCN3 dimer (48 mg, 0.032 mmol, 1 equiv) and M2Y1 (15 mg, 0.065 mmol, 2 equiv) were added. DCM (3 mL) and MeOH (1 mL) were added, and the resulting solution was stirred rapidly at 40 °C for 18 h. The solvent was removed *in vacuo*, and the residue was purified by flash column chromatography (Combiflash, 4 g SiO_2_ cartridge, 0–10% MeOH in DCM, with compound eluting at 7% MeOH as a broad peak). Sample loaded “wet” in DCM (approximately 2 mL)) to obtain the pure compound. Bright yellow solid, 55 mg (87% yield); ^1^H NMR (600 MHz, CDCl_3_) δ 10.86 (s, 1H), 8.87 (d, *J* = 7.9 Hz, 1H), 8.65 (s, 1H), 8.47 (td, *J* = 8.5, 3.0 Hz, 2H), 8.14 (td, *J* = 7.8, 1.6 Hz, 1H), 8.05 (td, *J* = 8.9, 2.1 Hz, 2H), 7.88 (dd, *J* = 2.1, 1.1 Hz, 1H), 7.78 (dd, *J* = 5.6, 1.5 Hz, 1H), 7.51 (dd, *J* = 2.2, 1.1 Hz, 1H), 7.42 (ddd, *J* = 7.7, 5.5, 1.2 Hz, 1H), 6.65 (ddd, *J* = 12.4, 8.9, 2.3 Hz, 1H), 6.59 (ddd, *J* = 12.5, 8.9, 2.3 Hz, 1H), 5.69 (dd, *J* = 8.0, 2.3 Hz, 1H), 5.60 (dd, *J* = 8.2, 2.3 Hz, 1H), 4.74 (ddd, *J* = 9.9, 6.7, 3.6 Hz, 2H), 3.85–3.67 (m, 2H), 1.97 (s, 3H); ^19^F NMR (565 MHz, CDCl_3_) δ −62.53 (s, 3F), −62.84 (s, 3F), −101.61 (dt, *J* = 12.6, 8.5 Hz, 1F), −102.52 (dt, *J* = 12.2, 8.6 Hz, 1F), −106.16 (td, *J* = 12.4, 3.4 Hz, 1F), −106.92 (td, *J* = 12.4, 3.6 Hz, 1F); ^13^C NMR (151 MHz, CDCl_3_) δ 172.0, 168.4, 167.7, 165.8, 165.2 (d, *J* = 12.8 Hz), 164.0 (d, *J* = 12.5 Hz), 163.4, 163.1, 161.7, 161.7, 154.4 (d, *J* = 6.8 Hz), 150.0, 149.9, 149.7, 147.9, 146.4, 144.8 (d, *J* = 4.7 Hz), 141.3, 136.6, 129.5, 126.9, 126.7 (d, *J* = 4.4 Hz), 126.5, 126.1, 125.9, 125.1, 123.7 (d, *J* = 21.0 Hz), 123.5 (d, *J* = 21.2 Hz), 122.6 (d, *J* = 14.5 Hz), 120.8 (d, *J* = 14.9 Hz), 114.5, 114.4, 100.4–100.1 (m), 100.1–99.7 (m), 52.0, 38.4, 23.0; HRMS (ESI+) (C_35_H_23_F_10_IrN_7_O)^+^ *m*/*z* (calculated) 940.1428, (found) 940.1443, mass difference 1.4 ppm; LC-MS retention time (ESI+, 7 min method, H_2_O:MeCN 0.1% formic acid): 3.17 min, *m*/*z* 940.30.

**IrCN3­(M12Y3)** To a 20 mL glass vial, IrCN3 dimer (43 mg, 0.029 mmol, 1 equiv) and M12Y3 (18 mg, 0.058 mmol, 2 equiv) were added. DCM (3 mL) and MeOH (1 mL) were added, and the resulting solution was stirred rapidly at 40 °C for 18 h. The solvent was removed *in vacuo*, and the residue was purified by flash column chromatography (Combiflash, 4 g SiO_2_ cartridge, 0–10% MeOH in DCM, with compound eluting at 7.4% MeOH as a broad peak). Sample loaded “wet” in DCM (approximately 2 mL) to obtain the pure compound. Yellow solid, 33 mg (54% yield); ^1^H NMR (600 MHz, CDCl_3_) δ 9.23 (s, 1H), 8.48 (dt, *J* = 8.1, 3.8 Hz, 2H), 8.40 (dd, *J* = 8.9, 1.0 Hz, 1H), 8.08 (dd, *J* = 8.8, 2.1 Hz, 2H), 7.82 (dd, *J* = 8.8, 5.3 Hz, 1H), 7.68 (d, *J* = 1.0 Hz, 1H), 7.46 (dd, *J* = 5.3, 1.0 Hz, 1H), 7.11–7.02 (m, 3H), 6.65 (ddd, *J* = 12.3, 8.9, 2.3 Hz, 1H), 6.55 (ddd, *J* = 12.4, 8.9, 2.3 Hz, 1H), 5.64 (ddd, *J* = 10.5, 8.1, 2.3 Hz, 2H), 4.40 (s, 3H), 3.86 (s, 3H), 3.79 (s, 3H); ^19^F NMR (565 MHz, CDCl_3_) δ −62.69 (s, 3F), −62.72 (s, 3F), −101.34 to −101.40 (m, 1F), −102.57 to −102.64 (m, 1F), −105.72 to −105.77 (m, 1F), −106.92 to −106.97 (m, 1F); ^13^C NMR (151 MHz, CDCl_3_) δ 168.6, 167.9, 165.8, 165.2 (d, *J* = 12.6 Hz), 164.0 (d, *J* = 12.6 Hz), 163.5 (d, *J* = 12.7 Hz), 163.2 (d, *J* = 12.9 Hz), 161.7 (d, *J* = 12.8 Hz), 161.5 (d, *J* = 13.0 Hz), 155.2, 154.1 (d, *J* = 6.9 Hz), 153.8, 150.7 (d, *J* = 7.4 Hz), 146.4, 146.1, 145.5, 145.2, 141.8, 138.0, 136.7, 136.6 (d, *J* = 3.2 Hz), 130.4, 129.9, 126.6 (d, *J* = 2.8 Hz), 126.4, 126.2, 126.1, 125.8, 124.8, 124.7, 124.5, 123.7 (t, *J* = 20.3 Hz), 122.7 (d, *J* = 7.5 Hz), 120.9 (d, *J* = 7.3 Hz), 117.6, 114.6–114.3 (m), 114.2 (d, *J* = 18.2 Hz), 114.0, 112.3, 100.4 (t, *J* = 26.5 Hz), 99.8 (t, *J* = 26.6 Hz), 58.5, 56.9, 56.3; HRMS (ESI+) (C_40_H_26_F_10_IrN_6_O_3_)^+^ *m*/*z* (calculated) 1021.1530, (found) 1021.1580, mass difference 4.6 ppm; LC-MS retention time (ESI+, 7 min method, H_2_O:MeCN 0.1% formic acid): 3.70 min, *m*/*z* 1021.31.

### Stability of Pure Compounds

Purified compounds were dissolved in DMSO to make solutions at 10 mM concentration. These were diluted down to 50 μM for stability tests with the following media: DMSO, DMSO:H_2_O (1:1), DMSO:PBS 1X (1:1), H_2_O, PBS (1X). UV–vis spectra (280–600 nm, 10 flashes) were collected over 21 h to monitor the stability of these complexes at 37 °C. For complimenting LC-MS stability studies, complexes were dissolved in DMSO (5 mM) then added to either DMSO (24 μL in 1200 μL) or 1:1 DMSO:PBS (1X) (24 μL in 1200 μL) in 2 mL HPLC vials fitted with a PTFE septum, giving a concentration of 100 μM. The samples were incubated at 37 °C inside the LC-MS sample chamber and analyzed by LC-MS over the course of 18 h.

### Bacterial Testing

Bacterial stock solutions were prepared as follows. Single bacterial colonies were grown on Tryptic Soy Broth (TSB) or Miller’s LB Broth (LB, *B. subtilis*) agar. A single colony was removed and placed in the relevant growth medium (5 mL TSB in a 15 mL falcon tube or 2 mL LB in a 50 mL Falcon tube) and incubated overnight at 37 °C (200 rpm). 100 μL of this overnight culture was added to 5 mL of the same growth medium, and the day culture was incubated at 37 °C (200 rpm) until the OD600 was 0.4–0.9 (approximately 4 h). A sample of this culture was diluted with Mueller-Hinton (MH) broth to OD600 = 0.022, at which point the bacterial cultures were added to plates for testing. For all testing, samples were incubated with bacteria for 16–20 h at 37 °C, after which the absorbance at 600 nm was measured using a plate reader. The antibiotic control was vancomycin (stock solution at 1 mg/mL in water, MIC beginning at 20 μg/mL) for all bacteria. Unless stated otherwise, 2 biological repeats per sample were recorded, with each having at least 2 technical repeats.

### Minimum Inhibitory Concentration (MIC) Assay

Stock solutions of samples were diluted with DMSO so that 6 μL of stock solution could be added to 300 μL of MH growth media to give an initial concentration of 100 μM. These solutions were serially diluted (150 μL into 150 μL) 7 times (crude compounds) or 11 times (pure compounds) to give solutions of 150 μL. To these, 5 μL of the relevant bacterial strain was added in MH growth media. The plates were incubated for 16–20 h at 37 °C, after which the absorbance at 600 nm was measured using a plate reader.

### Determination of the Cytotoxic Concentration 50% (CC_50_)–MTT Assay

HEK-293T cells were seeded at 10000 cells per well and incubated overnight to allow adhesion. Next, 5 mM stock solutions of pure compounds were prepared in DMSO. In a 1.5 mL 96-well dilution plate, starting concentrations of 150 μM and 200 μM of each compound were made in growth media (1 mL). 500 μL of these were serially diluted into 500 μL of media 11 times. 500 μL of media was then added to each well to make 1 mL of each concentration (starting at 75 μM and 100 μM). 100 μL of these solutions were added into wells containing the precounted cells and incubated for 24 h. To quantify cell viability, the cells were treated with the 3-(4,5-dimethylthiazol-2-yl)-2,5-diphenyltetrazolium bromide (MTT) salt (Apollo Scientific, UK) according to the procedure described by Mosmann.^51^ The absorbance at 595 nm was measured using a TECAN well plate reader instrument M1000. For each compound, 4 biological repeats in triplicate were done for this study (12 data points) from which the CC_50_ was determined.

## Supplementary Material







## Data Availability

The Python code used for automatic LC–MS data processing is available in the Frei Lab GitHub repository https://github.com/TheFreiLab/FreiLab-LCMSProcessing.

## Notes

Numerical LC–MS data, including retention times and conversion percentages, as well as biological data, including minimum inhibitory concentrations (MICs) of the crude libraries are provided (XLSX)
This manuscript was originally published as a preprint on ChemRxiv prior to submission.^52^ All experimental data supporting the findings of this study and experimental methods (PDF)
The compiled LC-MS spectra for all combinatorial libraries (PDF)
