# Supplementary material for: High-Throughput Synthesis and Screening of Cyclometalated Iridium Complexes for Antibacterial Applications
Source: Inorg Chem. 2026 Oct 28;65(44):25793–807. doi: 10.1021/acs.inorgchem.6c03421 (PMC13753727; doi:10.1021/acs.inorgchem.6c03421)

IrCN1-Schiff library

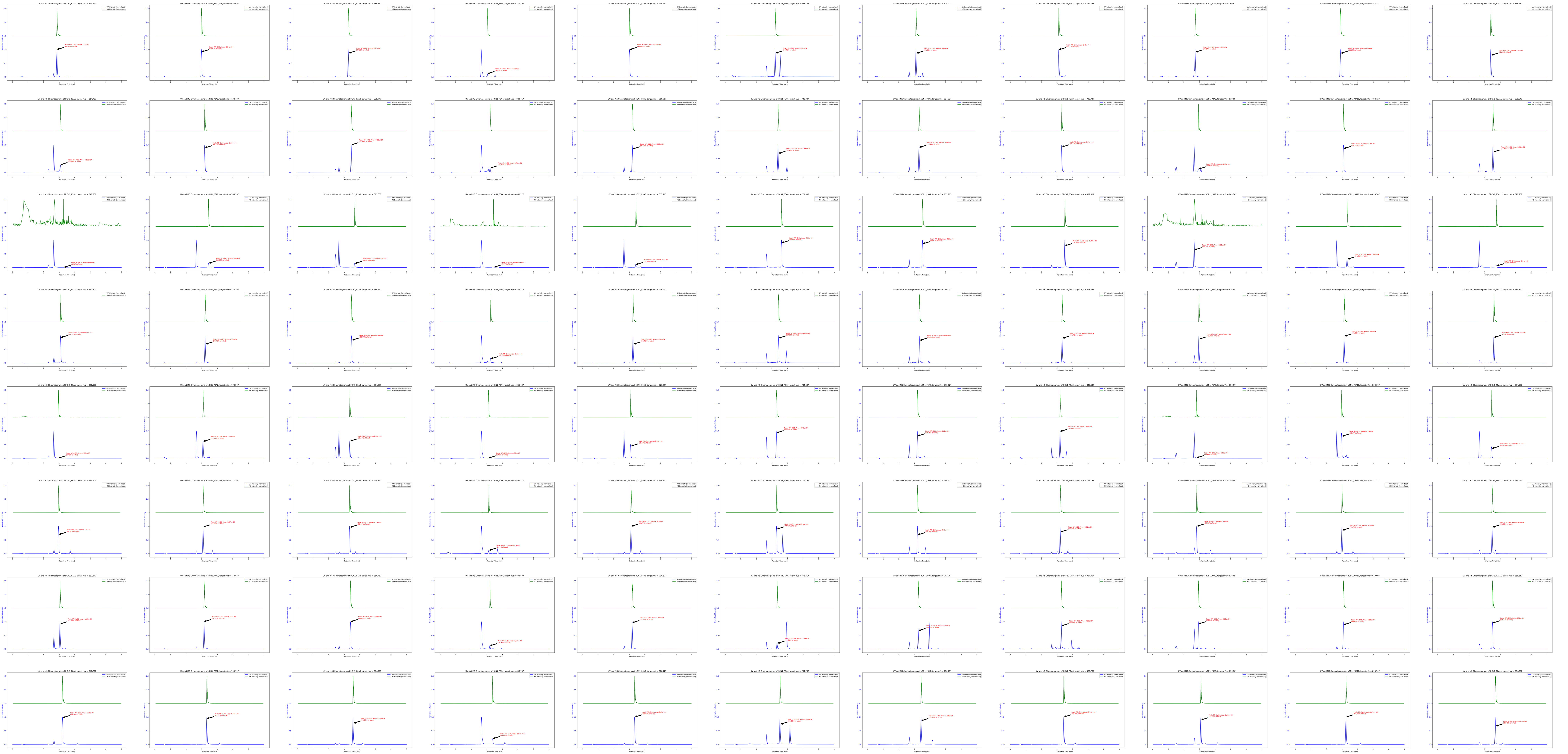

hCA2-Schiff library

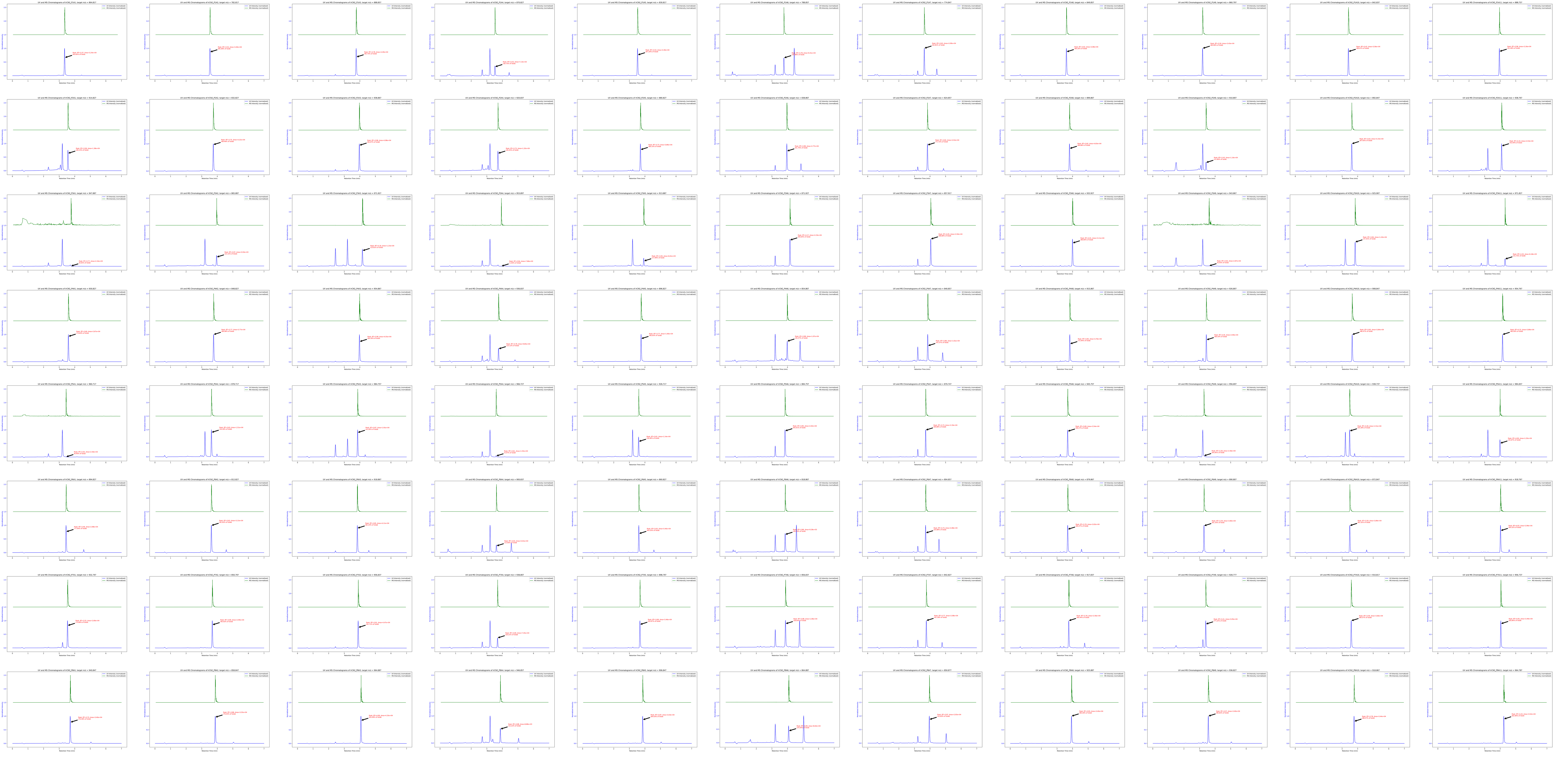

IrCN3-Schiff library

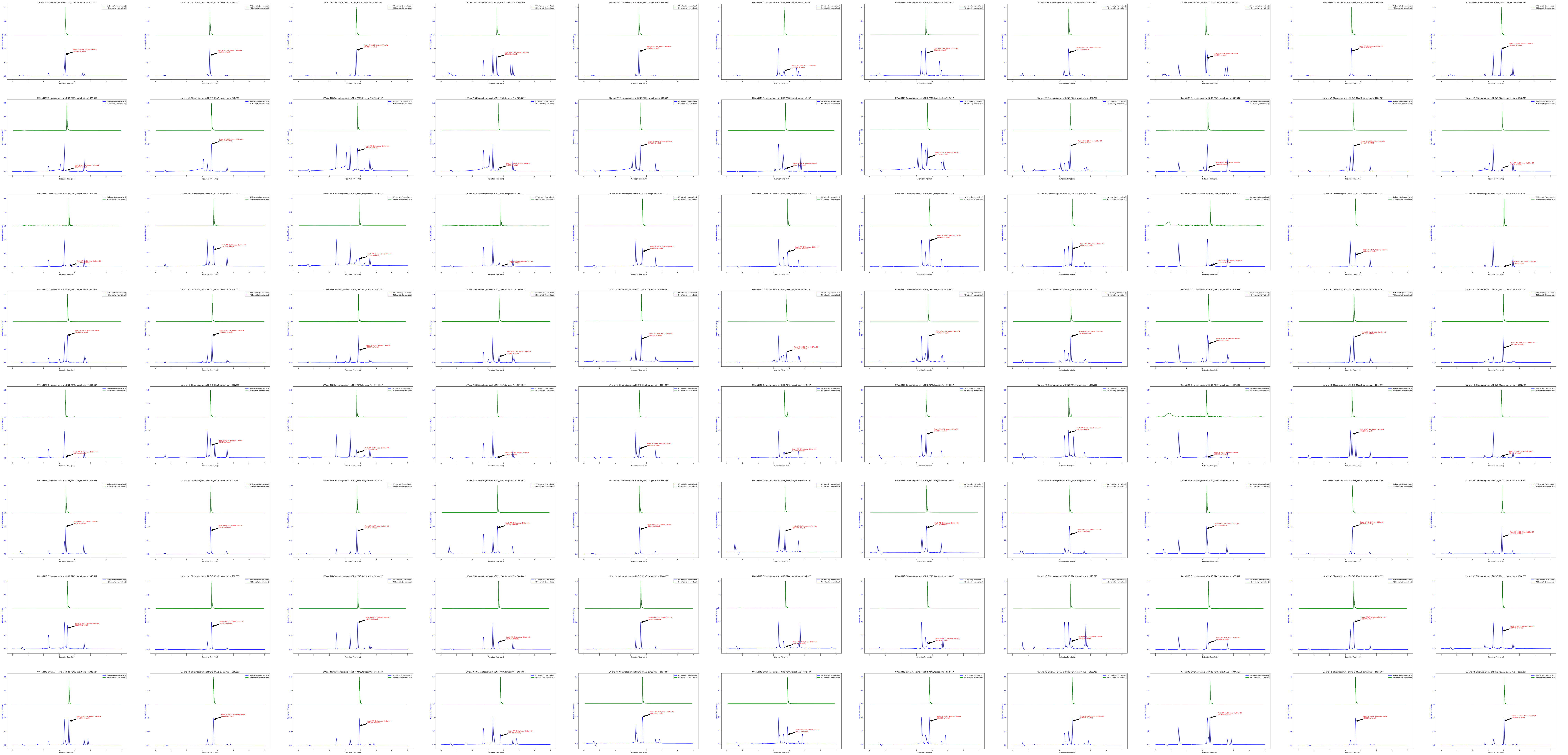

Click ligand library remade

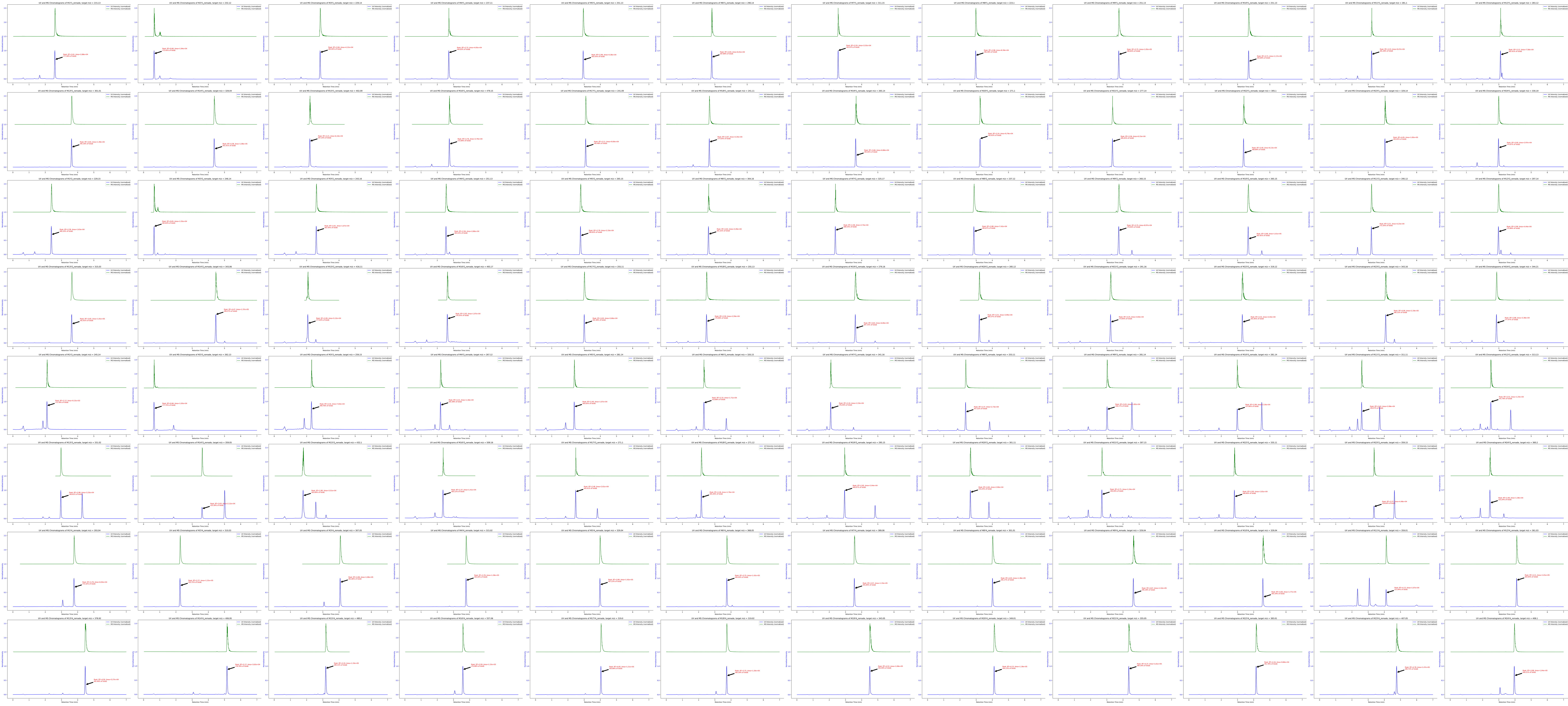

IrCN1 Click library remade

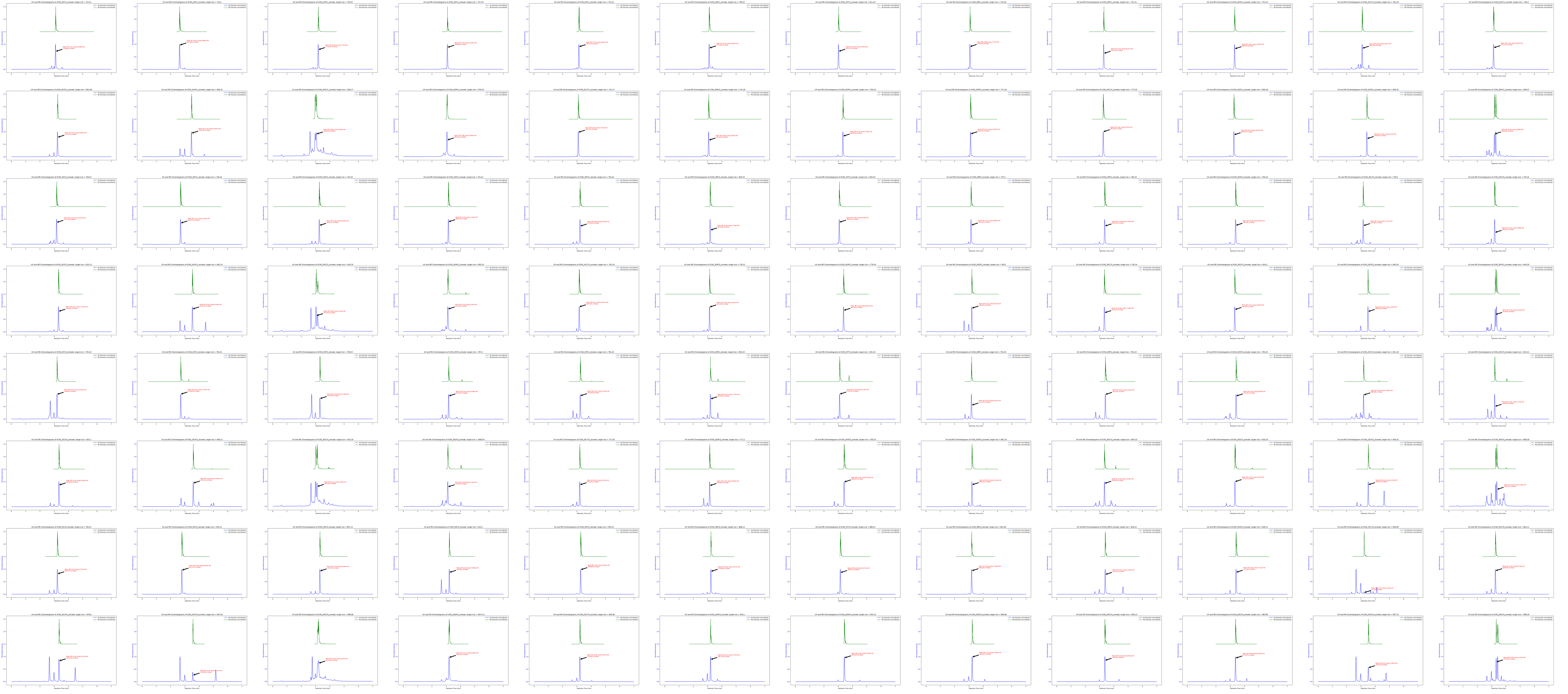

IrCN2 Click library rerun

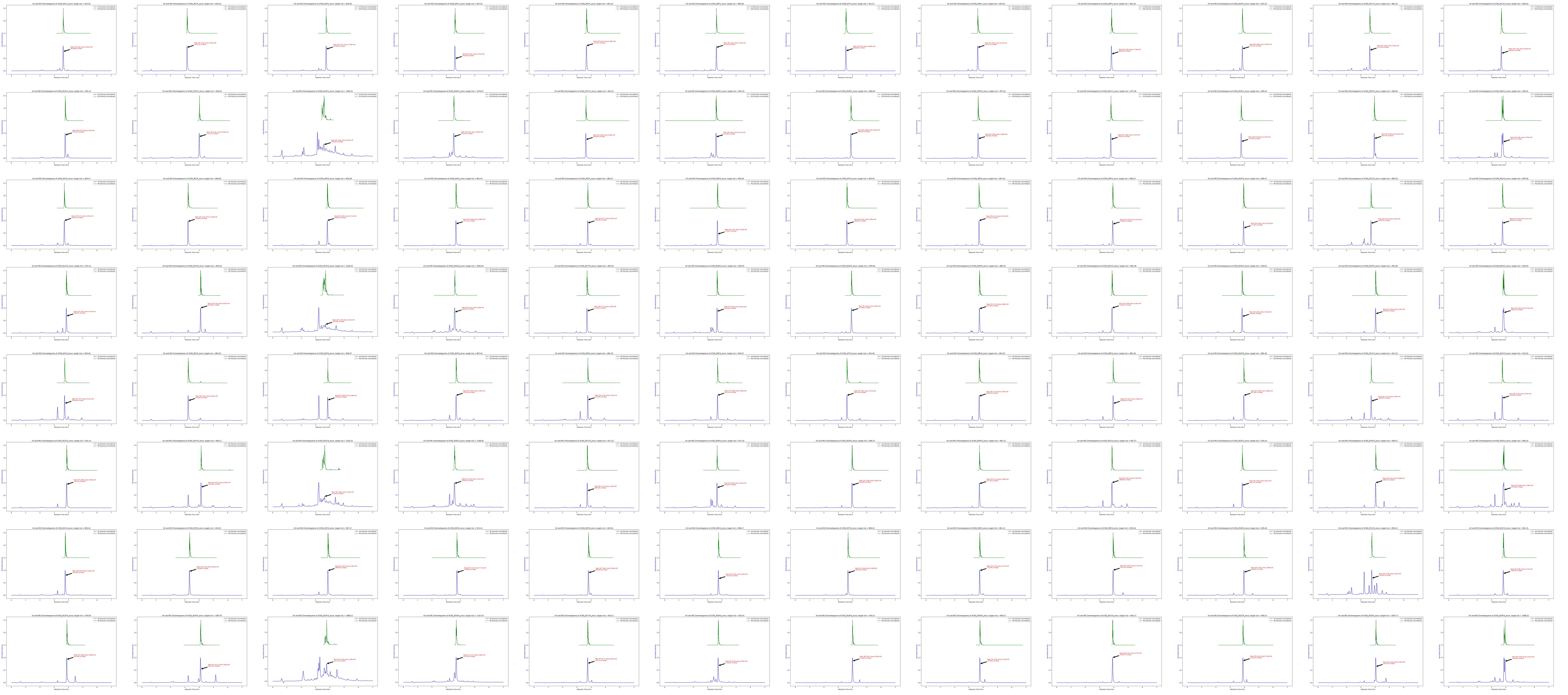

IrCN2 Click library rerun

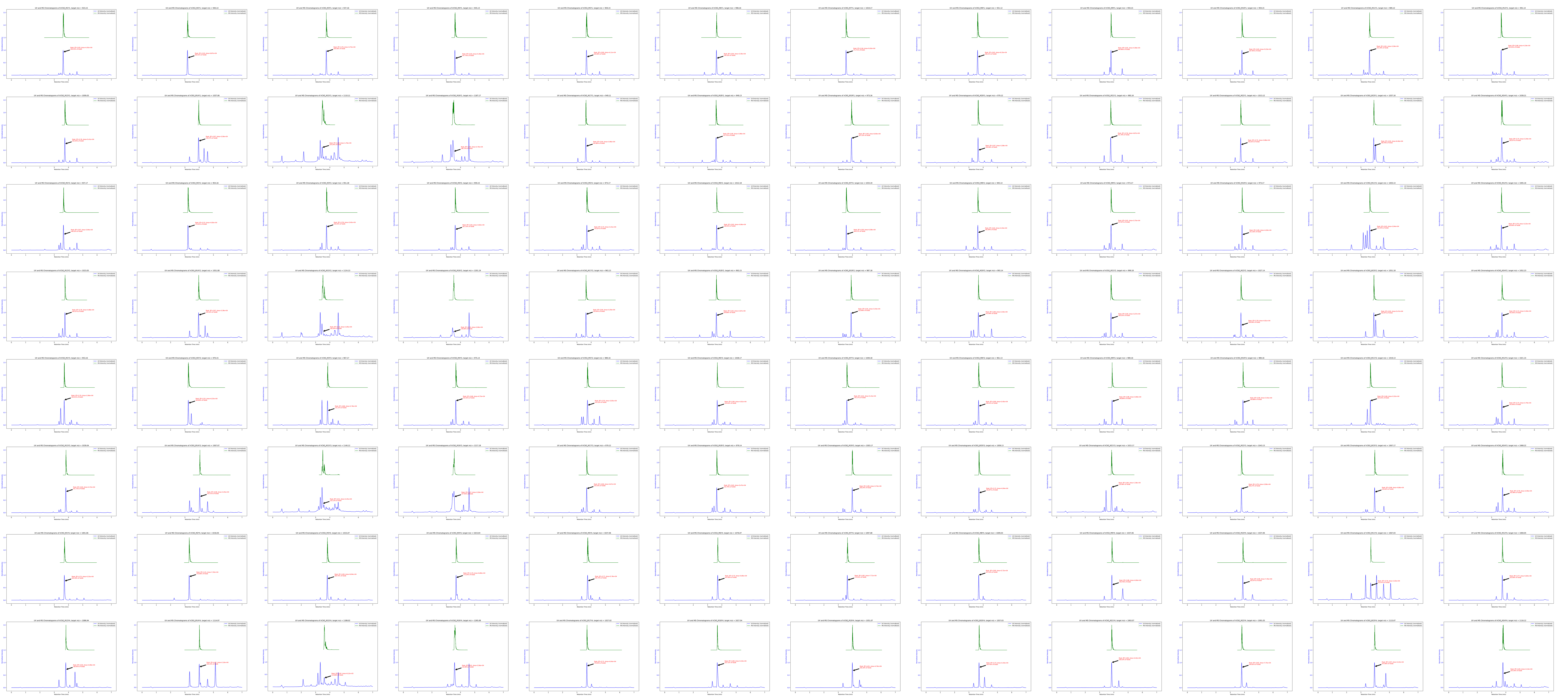

IrCN Stability after 1 year

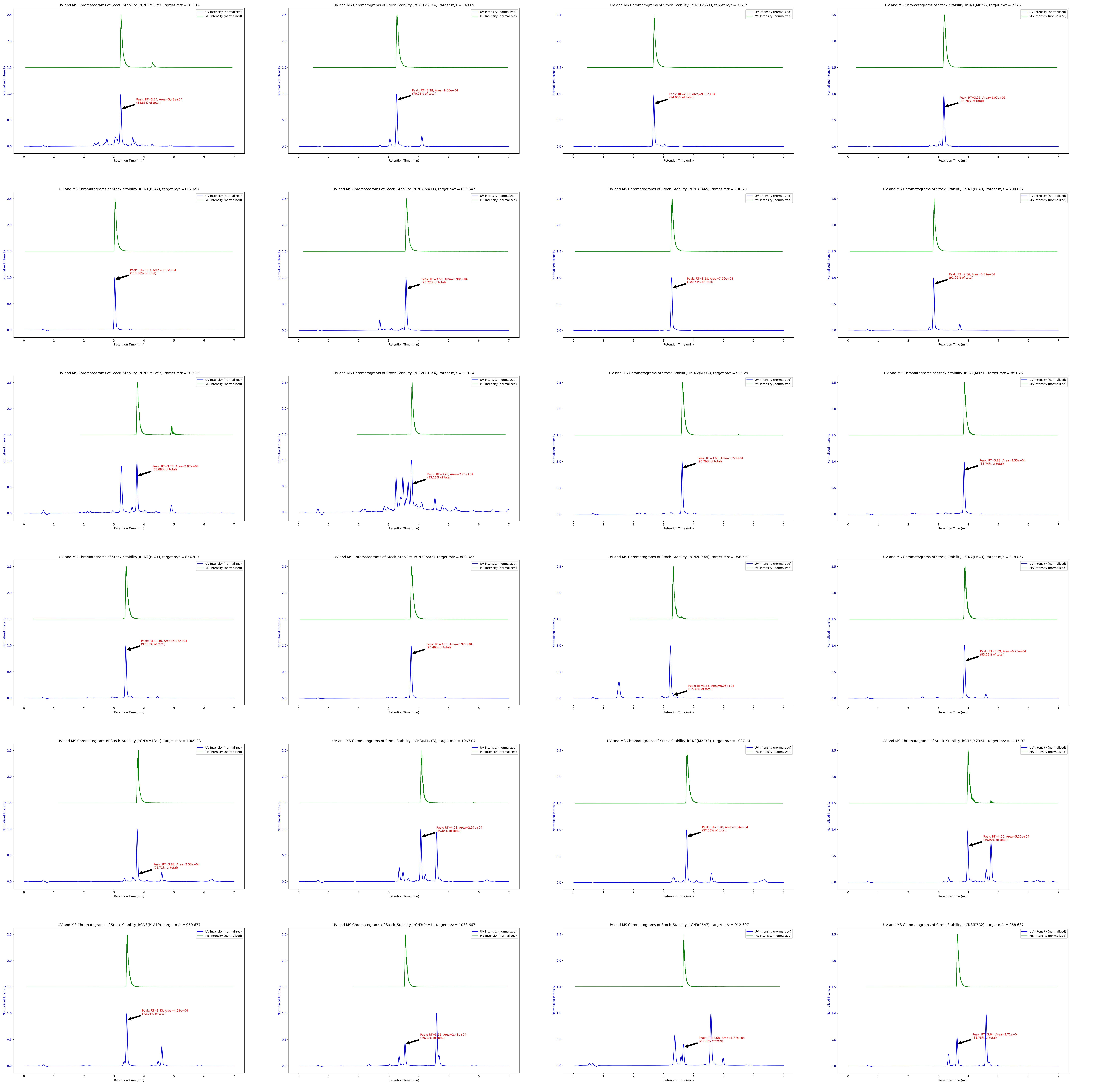

Supplement: Supplementary file 1 [file ic6c03421_si_002.pdf]
