# Supplementary material for: High-Throughput Synthesis and Screening of Cyclometalated Iridium Complexes for Antibacterial Applications
Source: Inorg Chem. 2026 Oct 28;65(44):25793–807. doi: 10.1021/acs.inorgchem.6c03421 (PMC13753727; doi:10.1021/acs.inorgchem.6c03421)
Supplement: Supplementary file 2 [file ic6c03421_si_003.pdf]

Supporting Information for

High-throughput synthesis and screening of cyclometallated iridium complexes for antibacterial applications

*David R. Husbands†[1], Megan G. Dangerfield†[1], Athi Welsh[1], Lukas Geciauskas[1], Çağrı Özsan[1,2], Kiera Robinson[1], Richard Gammons[1], Angelo Frei[1,2]**

†These authors contributed equally to this work

[1] Department of Chemistry, University of York, York YO10 5DD, United Kingdom

[2] Department of Chemistry, Biochemistry & Pharmaceutical Sciences, University of Bern, Freiestrasse 3, 3012 Bern, Switzerland

*Corresponding author email: angelo.frei@york.ac.uk

Contents

1. General Information 3

1.1 Compound Preparative Techniques....3

1.2 Instrument Details and Methods for Compound Characterization 3

2. Experimental Details....4

2.1 Preparation of IrCN scaffolds....4

2.2 Preparation of IrCN-Schiff combinatorial libraries....6

2.3 Preparation of the Click ligand library 7

2.4 Preparation of the IrCN-Click combinatorial libraries....8

2.5 Batch synthesis of Click ligands....9

2.6 Batch synthesis of IrCN-Schiff complexes 11

2.7 Batch synthesis of IrCN-Click complexes 17

3. Photophysical Properties....21

4. Stability of pure compounds 24

5. LCMS Processing....50

5.1 LCMS Stability of Crude Reaction Mixtures over 1 year....53

6.Biological testing....56

6.1 Minimum Inhibitory Concentration (MIC)....56

6.2 Toxicity Testing 64

7. HRMS data of pure compounds 66

8. LCMS data of pure compounds 72

9. X-Ray Crystallography 78

10. NMR Spectral data for Organic and Inorganic Compounds 86

11. References 115

1. General Information

1.1 Compound Preparative Techniques

Reagents were purchased from Merck, Fluorochem, Alfa Aesar, and Fisher and used without further purification. The purity of chemicals was confirmed by LCMS before use.

1.2 Instrument Details and Methods for Compound Characterization

NMR spectra were obtained in the solvent indicated in the text below, using a Bruker Avance Neo 700 instrument fitted with a liquid N_2 cooled triple resonance cryoprobe (700 MHz [^{1}H], 659 MHz [^{19}F], 176 MHz [^{13}C]) Bruker AVIIIHD 600 Widebore instrument (600 MHz [^{1}H], 565 MHz [^{19}F], 151 MHz [^{13}C]) or JEOL ECX400 or JEOL ECS400 spectrometer (400 MHz [^{1}H], 101 MHz [^{13}C] and 377 MHz [^{19}F]). Chemical shifts (δ) are reported in parts per million (ppm) and were referenced to the residual non-deuterated solvent of the deuterated solvent used; $CDCl_3$: δ ^{1}H = 7.26 (C*H*Cl_3) and ^{13}C = 77.16 ($CDCl_3$); $(CD_3)_2SO$: ^{1}H = 2.50 ((CD_3)(CD_2*H*)SO) and ^{13}C = 39.52 ((*C*D_3)$_2$SO); CD_2Cl_2 : δ ^{1}H = 5.32 (C*H*DCl_2) and ^{13}C = 53.89 (CD_2Cl_2);. Spectral data were typically collected at 298 K (25 °C), unless stated otherwise. All ^{1}H NMR signals are reported as they appear in the spectrum. All ^{19}F spectra were referenced using the spectrometers internal referencing.

All ^{13}C NMR spectra were obtained with ^{1}H decoupling. All NMR spectra were processed using MestReNova (MNova) software (v. 14).

High-resolution mass spectrometry (HRMS) ESI-MS spectra were measured using a Bruker Daltronics micrOTOF MS, Agilent series 1200LC with electrospray ionization (ESI) or on a Thermo LCQ using electrospray ionization, with <5 ppm error recorded for all HRMS samples. High resolution mass spectra (HRMS) are reported with <5 ppm error (ESI). For clarity, data are reported for the most abundant natural isotope of metals, which is part of 'exact mass' values. ESI ions are reported as the $[M+H]^+$ cation, unless a Na or K is present in the molecular formula, in which case the $[M+Na]^+$ or $[M+K]^+$ ion is being measured.

LCMS data was collected using a Dr. Maisch ReproSil-Pur 120 ODS-3, 2.4 μm, 50 mm x 3 mm column, on a ThermoScientific Vanquish instrument, coupled to a ThermoScientific HCT ultra ETD II ion trap. Mass spectrometry (ESI) was run in the positive mode. The solvent system consisted of H_2O / MeCN, both containing 0.1% formic acid. The gradient used for the "7 minute method" that was commonly used is described in Table S1. The UV channels correspond to UV1: 200 nm, UV2: 220 nm, UV3: 254 nm. Characterization data and assessments of purity on the generated libraries were done using 254 nm absorbances.

Table S1: Flow gradient for LCMS "7 min method"

Time / min	Flow / mL min^{-1}	% MeCN

0.0	0.500	10.0
4.0	0.500	90.0
6.3	0.500	90.0
6.4	0.500	10.0
7.0	0.500	10.0

UV-Vis absorbance and fluorescence data was collected using a Tecan Infinite 200 Pro plate reader, using the “Greiner 96 Flat Bottom Transparent Polystyrene” option.

All reaction temperatures were measured independently from the heating block using a TENMA 72-7715 electronic thermometer and thermocouple to ensure accurate reaction temperature regulation. 96-well reactions were shaken and heated using an Eppendorf ThermoMixer with “plates” attachment. Irradiation at 405 nm was achieved using a 96 LED array (250 mW/LED) (Atlas Photonics Lumos Bio).

2. Experimental Details

2.1 Preparation of IrCN scaffolds

General method

Adapted from Kench *et al.*[1]

136 mg of $IrCl_3.xH_2O$ (0.37 mmol Ir) and ligand (2.2 equiv., 0.82 mmol) were added to a microwave vial. 2-methoxyethanol (6 mL) and DI water (2 mL) were added, and the vial was sealed. The mixture was heated at 150 °C for 10 mins in a microwave reactor, and after cooling, water (20 mL) was added to precipitate the complex. The precipitate was isolated by filtration, washed with water (2 x 20 mL), methanol (2 x 2 mL) and Et_2O (3 x 20 mL). The precipitate was dried under a flow of air, yielding the pure product. Formation of the complex was confirmed by agreement of ^{1}H NMR spectra to the literature.

IrCN1 dimer

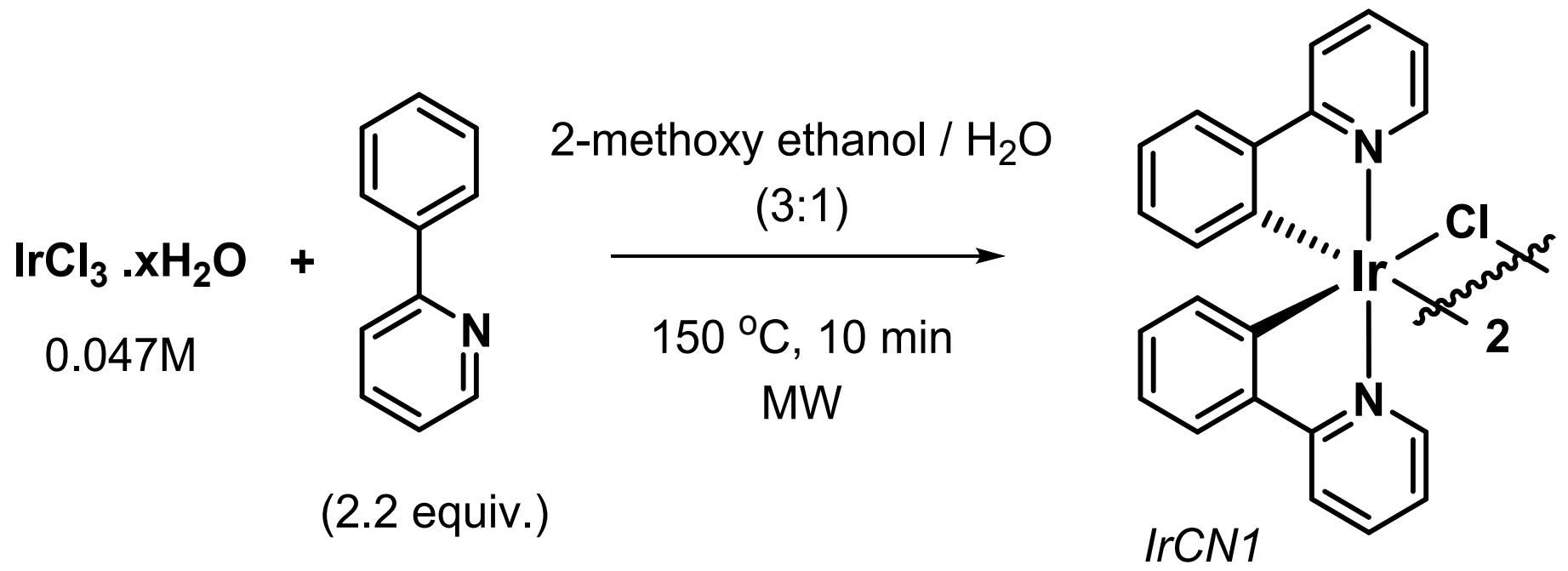

Yellow solid, 130 mg (65% yield);

^{1}H NMR (400 MHz, DMSO-*D*$_6$) δ 9.84 – 9.78 (m, 1H), 9.53 (dd, *J* = 5.9, 0.9 Hz, 1H), 8.30 – 8.24 (m, 1H), 8.18 (dd, *J* = 8.2, 1.3 Hz, 1H), 8.09 (ddd, *J* = 8.1, 7.4, 1.6 Hz, 1H), 8.04 – 7.98 (m, 1H), 7.79 (dd, *J* = 7.7, 1.4 Hz, 1H), 7.73 (dd, *J* = 7.7, 1.4 Hz, 1H), 7.57 (ddd, *J* = 7.3, 5.8, 1.4 Hz, 1H), 7.45 (ddd, *J* = 7.4, 5.8, 1.5 Hz, 1H), 6.89 (td, *J* = 7.5, 1.2 Hz, 1H), 6.84 (td, *J* = 7.5, 1.2 Hz, 1H), 6.76 (td, *J* = 7.4, 1.4 Hz, 1H), 6.69 (td, *J* = 7.4, 1.3 Hz, 1H), 6.25 (dd, *J* = 7.7, 1.1 Hz, 1H), 5.66 (dd, *J* = 7.8, 1.1 Hz, 1H).

IrCN2 dimer

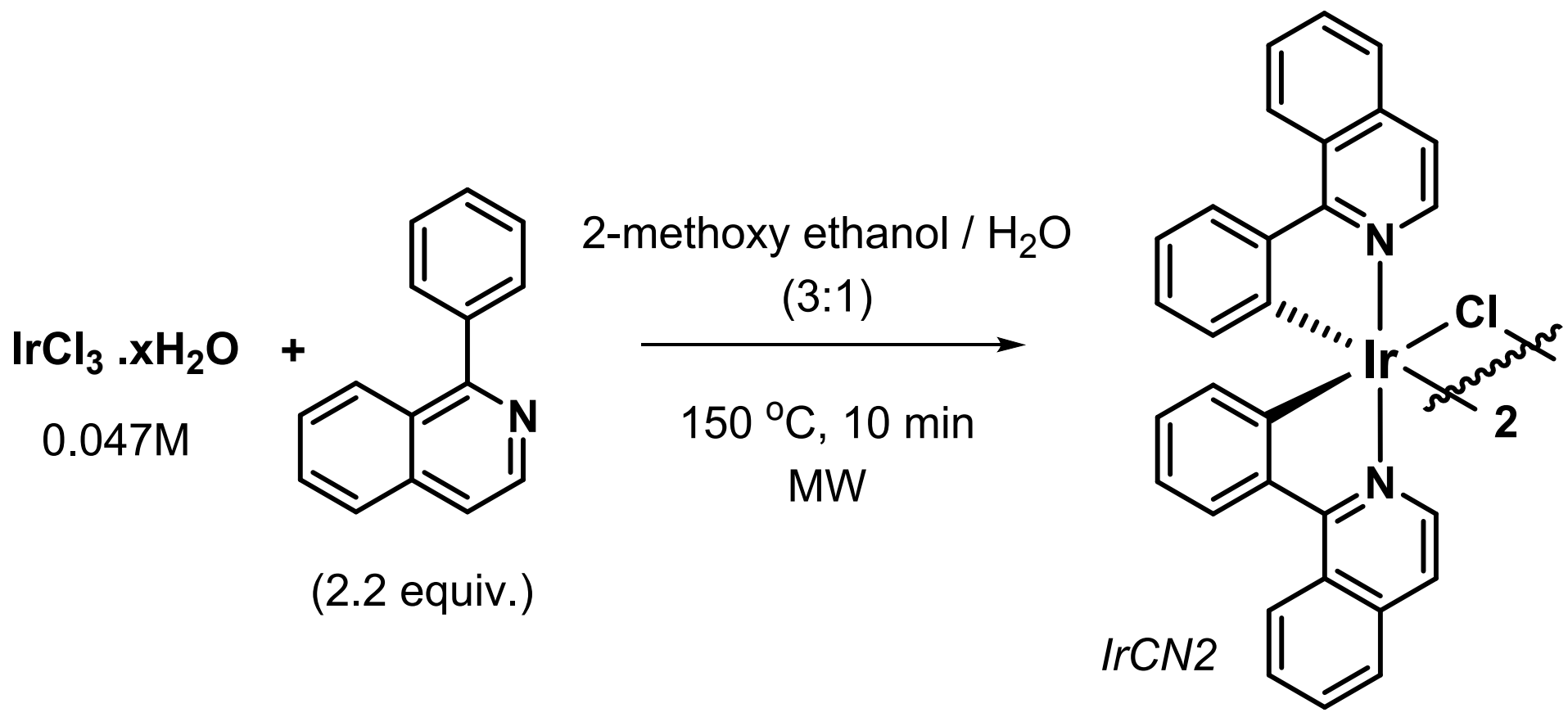

Red-brown solid, 179 mg (59% yield);

^{1}H NMR (400 MHz, DMSO-*d*$_6$) δ 9.75 (d, *J* = 6.6 Hz, 1H), 9.58 (d, *J* = 6.4 Hz, 1H), 8.90 (dd, *J* = 22.9, 8.8 Hz, 2H), 8.28 – 8.16 (m, 3H), 8.13 (d, *J* = 8.1 Hz, 1H), 8.00 (dd, *J* = 24.0, 6.9 Hz, 2H), 7.97 – 7.80 (m, 5H), 7.01 (t, *J* = 7.4 Hz, 1H), 6.92 (t, *J* = 7.6 Hz, 1H), 6.79 (t, *J* = 7.5 Hz, 1H), 6.64 (t, *J* = 7.5 Hz, 1H), 6.32 (d, *J* = 7.9 Hz, 1H), 5.56 (d, *J* = 7.7 Hz, 1H);

IrCN3 dimer

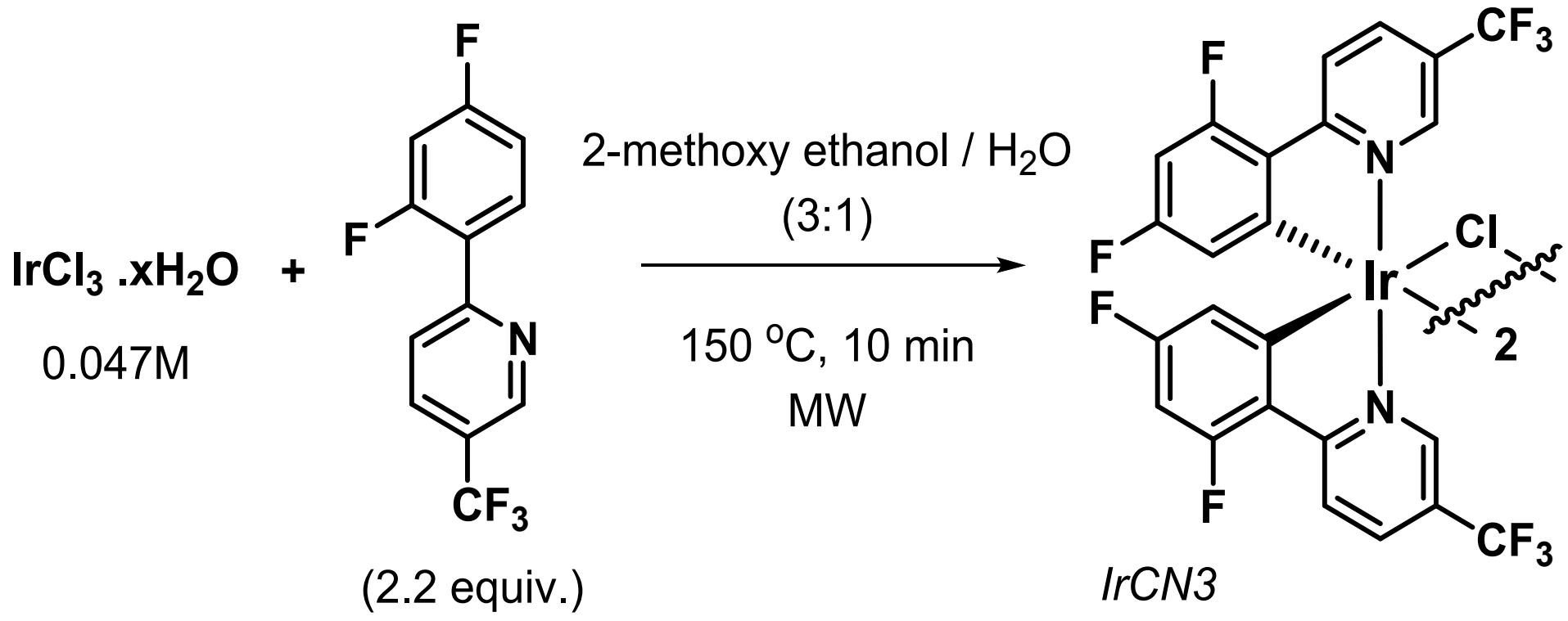

Yellow solid, 200 mg (72% yield);

^{1}H NMR (400 MHz, DMSO-d_6) δ 10.48 (s, 1H), 9.83 (s, 1H), 8.62 (dd, *J* = 8.8, 2.2 Hz, 1H), 8.57 – 8.41 (m, 3H), 7.05 – 6.88 (m, 2H), 5.94 (dd, *J* = 8.3, 2.3 Hz, 1H), 5.03 (dd, *J* = 8.7, 2.3 Hz, 1H);

^{19}F NMR (376 MHz, DMSO-d_6) δ -60.88 (s, 3F), -61.10 (s, 3F), -103.16 (q, *J* = 9.7 Hz, 1F), -104.63 (q, *J* = 9.5 Hz, 1F), -106.66 (t, *J* = 12.3 Hz, 1F), -107.76 (t, *J* = 12.1 Hz, 1F).

2.2 Preparation of IrCN-Schiff combinatorial libraries

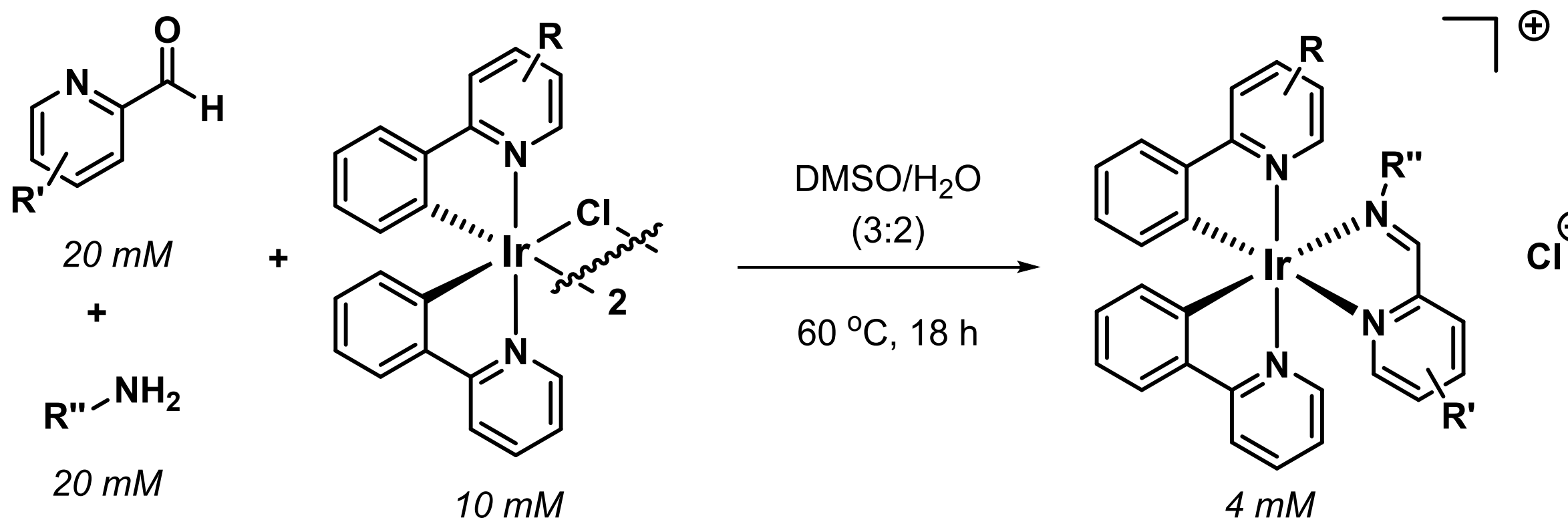

Synthesis of the IrCN Schiff libraries was undertaken using an Opentrons OT2 robotic platform with a 300 µL pipette. Paradox® metal plates containing 96 x 700 µL glass vials were used, with each as a designated well. To each designated well, the amine (90 µL, 20 mM in DMSO), aldehyde (90 µL, 20 mM in DMSO) and IrCN dimer (90 µL, 10 mM in DMSO) were added, followed by 180 µL of H_2O. The plate was sealed and heated for 18 h using an Eppendorf Thermomixer (60 °C, 600 rpm). The conversion of each well was measured by LCMS (254 nm). Each well contained 450 µL of 4 mM stock in a 3:2 DMSO:H_2O mixture (assuming 100% conversion to product).

2.3 Preparation of the Click ligand library

The synthesis of the Click library was reported in our previous paper.[2] For this work, the library was remade using the same procedure, which is replicated here for convenience.

The following procedure was carried out using an Opentrons liquid handling robot OT2, utilizing both 300 µL and 20 µL pipettes (Gen2). This procedure generates ligands at nominal concentration of 20 mM in 400 µL of solvent. It should be noted that the solubility of some ligands is an issue, with a number precipitating or crystallizing out of solution over time. This can be mitigated to a certain extent by further dilution, or by sonicating the individual reaction vials to pulverize the crystals. For accurate additions, these ligand solutions were aspirated 3 times to ensure efficient mixing before dispensing.

Preparation of the FSO_2N_3 reagent

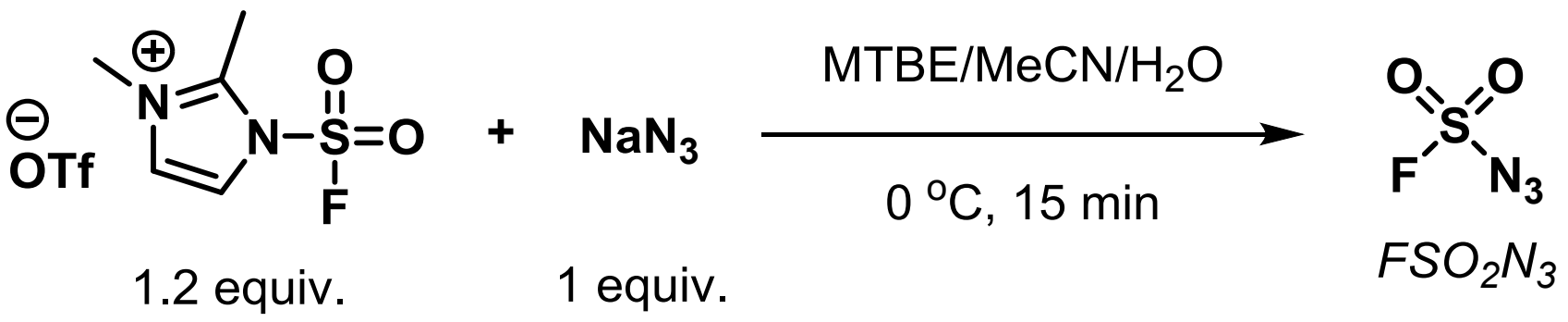

Method adapted from Meng *et al.*[3]

NaN_3 (99 mg, 1.524 mmol, 1 equiv.) was dissolved in deionized water (3.0 mL) in a 15 mL falcon tube. To this, 3.0 mL of MTBE was added and the mixture stirred vigorously at 0 °C. Separately, 1-(fluorosulfonyl)-2,3-dimethyl-1H-imidazol-3-ium trifluoromethanesulfonate (600 mg, 1.83 mmol, 1.2 equiv.) was dissolved in 150 µL of MeCN in a falcon tube and was added to the reaction mixture. A further 150 µL of MeCN was used to wash the falcon tube and ensure efficient transfer of material, with this also being added to the reaction mixture. The mixture was stirred vigorously at 0 °C with the lid loosely screwed on top for 15 min, then allowed to settle at r.t. for 30 min The upper organic layer was decanted and diluted with 3 mL DMSO, giving a solution of FSO_2N_3 of approximately 173 mM (calculated according to the method described by Meng *et al.*).[3]

The following procedure assumes a concentration of 173 mM for FSO_2N_3 and describes the addition to 1 well of a Paradox® 96 well plate (700 µL glass vial) using a Opentrons OT2 liquid handling robot (P300 Gen2 300 µL pipette and P20 Gen2 20 µL pipette). It is also adapted from Meng *et al.*[3]

Preparation of the ligand library

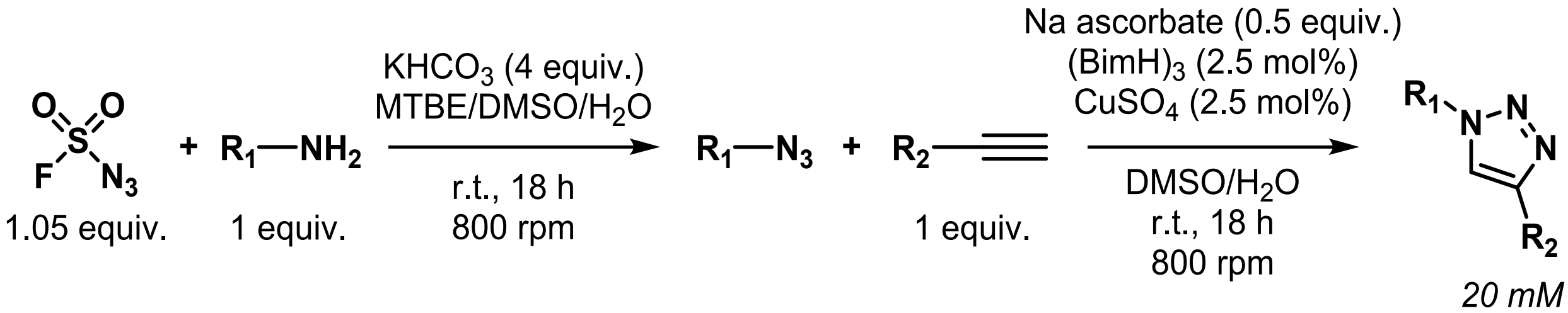

To the reaction well, sat. $KHCO_3$ solution (11 µL, 4 equiv., nominally 3 M), followed by DMSO (65 µL, Note: this is the volume required to make up the total volume to 200 µL, while additionally accounting for evaporation of MTBE, which is half the volume of the FSO_2N_3 stock solution) was added, then amine stock solution (100 µL, 80 mM, 1 equiv.). FSO_2N_3 stock solution was then added (48 µL, 173 mM 1.05 equiv., Note: the pipette was aspirated 3 times to equilibrate solvent vapor pressure. It is recommended to prepare a significant excess (25%) of the Sharpless reagent to account for evaporation during addition). The reaction vials were then sealed, and shaken at 800 rpm for 18 h at r.t.

Next, to each reaction well, sodium ascorbate solution (80 µL, 50 mM, 0.5 equiv.) was added and the reaction shaken for a further 15 min at r.t.

To each reaction well, alkyne stock solution (100 µL, 80 mM, 1 equiv.) was added, followed by $(BimH)_3$ solution in DMSO (10 µL, 20 mM, 2.5 mol%), then $CuSO_4$ solution in water (10 µL, 20 mM, 2.5 mol%). The reaction was sealed, and shaken at 800 rpm for 18 h at r.t. The reaction mixture was analysed by LCMS (5 µL in 200 µL MeCN / H_2O (60:40)) to confirm the presence and identity of the product ligand.

2.4 Preparation of the IrCN-Click combinatorial libraries

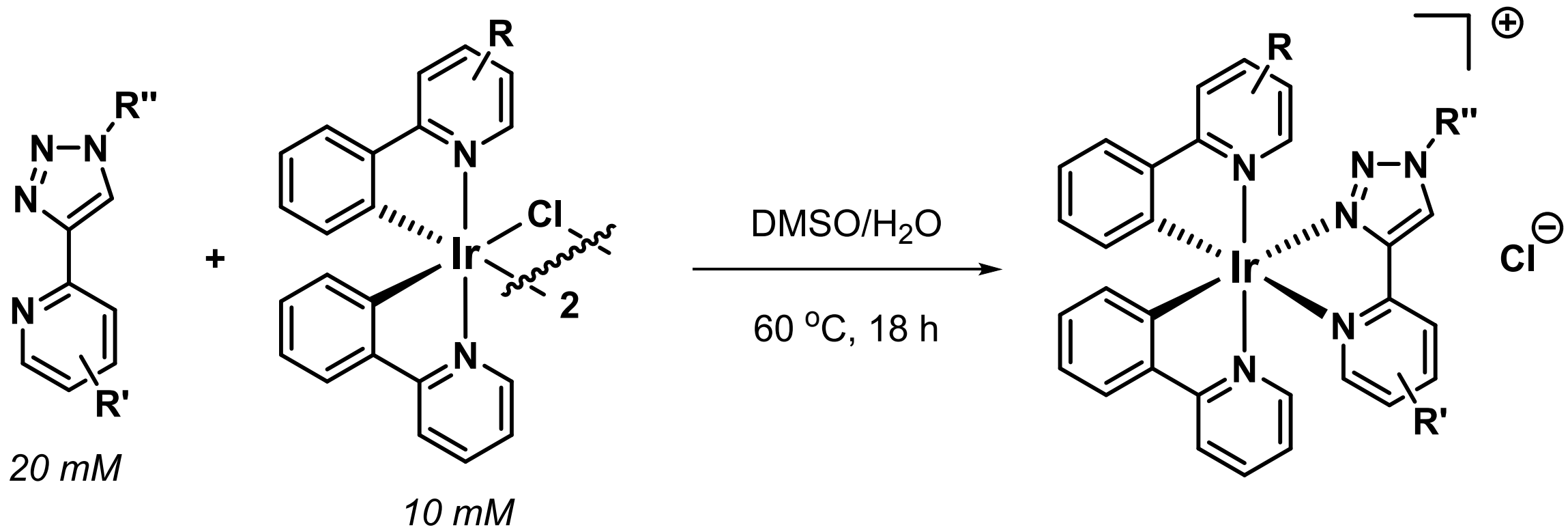

Synthesis of the IrCN-Click libraries was undertaken using an Opentrons OT2 robotic platform with a 300 µL pipette. Paradox® metal plates containing 96 x 700 µL glass vials were used, with each as a designated well. To each designated well, the triazole ligand (90 µL, 20 mM in DMSO) and IrCN dimer (90 µL, 10 mM in DMSO) were added, followed by 180 µL of H_2O. The plate was sealed and heated for 18 h using an Eppendorf Thermomixer (60 °C, 600 rpm). For IrCN1, each well contained 360 µL of 5 mM stock in a 1:1 DMSO:H_2O mixture (assuming 100% conversion to product).

For IrCN2, the reaction was not complete by LCMS, so a further 90 µL of DMSO was added to each well, the plate was sealed and heated for a further 18 h at 60 °C (600 rpm). An additional 90 µL of DMSO was then added to each well, so that each well contained 540 µL of 3.3 mM stock in a 2:1 DMSO:H_2O mixture (assuming 100% conversion to product).

For IrCN3, the reaction was not complete by LCMS, so a further 180 µL of DMSO was added to each well, the plate was sealed and heated for a further 18 h at 65 °C (600 rpm). Each well contained 540 µL of 3.3 mM stock in a 2:1 DMSO:H_2O mixture (assuming 100% conversion to product).

2.5 Batch synthesis of Click ligands

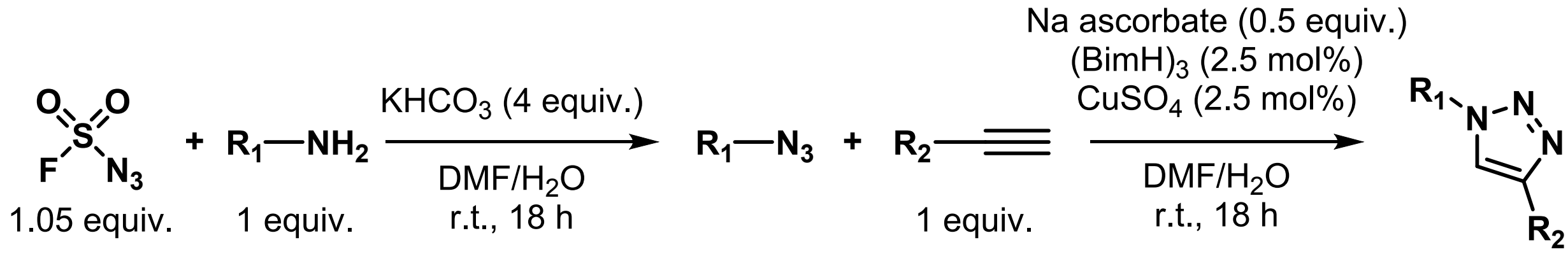

FSO_2N_3 was synthesized as before and diluted with DMF to approx. 200 mM in 1:1 MTBE:DMF. The desired amine (0.56 mmol, 1.0 equiv.) was charged into a 50 mL polypropylene Falcon tube, followed by DMF (7 mL). Sat. $KHCO_3$ solution (0.8 mL, approx. 2.4 mmol, 4 equiv. in water) was added, followed by FSO_2N_3 solution (1.05 equiv.). The resulting mixture was stirred at r.t. for 18 h. Sodium ascorbate solution (5.6 mL, 50 mM, 0.5 equiv. in water) was added, and the mixture was stirred for a further 10 mins. 2 mL of DMF was added, followed by alkyne (0.56 mmol, 1.0 equiv.), then $(BimH)_3$ solution (0.7 mL, 20 mM, 2.5 mol% in DMF) and $CuSO_4$ solution (0.7 mL, 20 mM, 2.5 mol% in water). The resulting mixture was stirred for a further 18 h at r.t.

The crude reaction mixture was analyzed by LCMS, then 100 mL of deionized water was added. The mixture was extracted with EtOAc (5 x 20 mL) (and for M2Y1, additional 3:1 $CHCl_3$: isopropyl alcohol (3 x 20 mL). The combined organic layers were washed with brine (5 x 25 mL), dried ($MgSO_4$), filtered, and the solvent evaporated. Toluene (2 x 25 mL) was added to the residue and the solvent removed in vacuo to remover residual DMF. Purification was achieved by flash column chromatography (Combiflash, 12g SiO_2 cartridges. All compounds were loaded 'wet' by dissolution in the minimum volume of EtOAc or DCM).

M2Y1

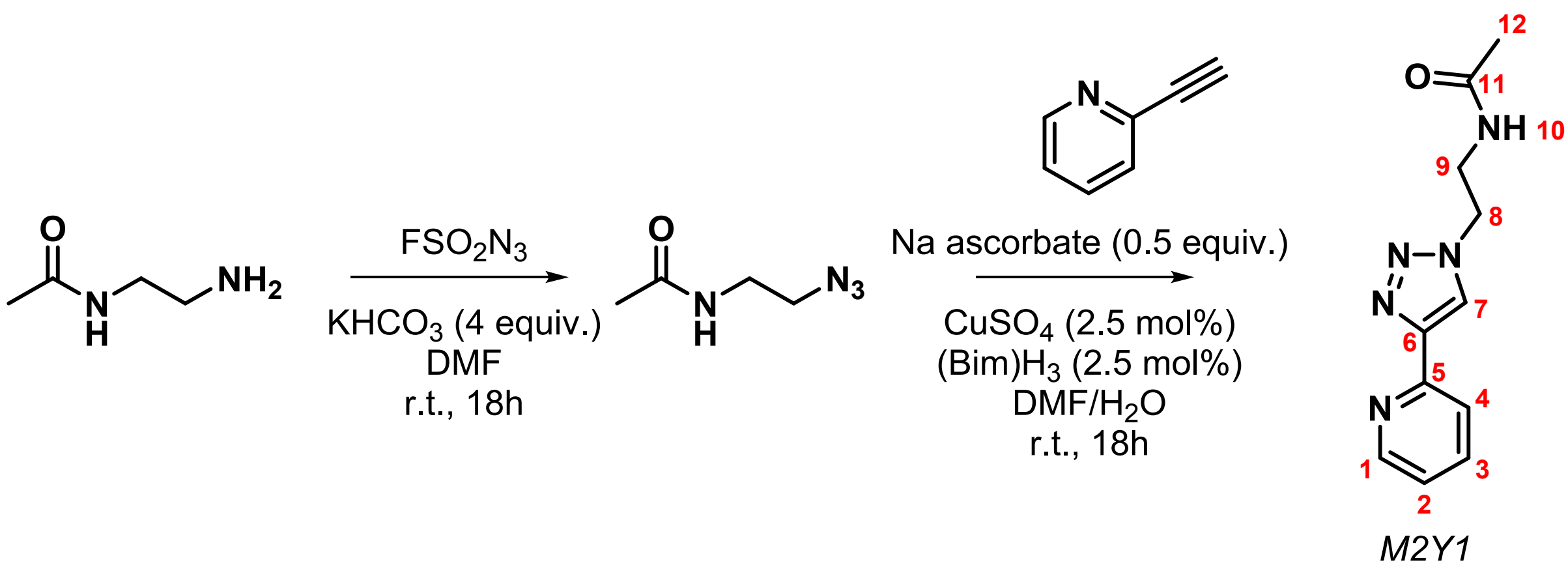

Column chromatography in MeOH/DCM (0% - 10% gradient), eluting at 7% MeOH.

Cream solid, 63 mg (49% yield), R_f = 0.39 (10% MeOH in DCM);

^{1}H NMR (400 MHz, $CDCl_3$) δ 8.54 (ddd, J = 4.9, 1.8, 0.9 Hz, 1H, H-1), 8.12 (s, 1H, H-7), 8.06 (dt, J = 8.0, 1.1 Hz, 1H, H-4), 7.75 (td, J = 7.7, 1.8 Hz, 1H, H-3), 7.22 (ddd, J = 7.6, 4.9, 1.2 Hz, 1H, H-2), 6.38 (s, 1H, H-10), 4.58 – 4.50 (m, 2H, H-8), 3.86 – 3.76 (m, 2H, H-9), 1.98 (s, 3H, H-12);

^{13}C NMR (101 MHz, $CDCl_3$) δ 170.9 (C-11), 150.0 (C-5), 149.5 (C-1), 148.5 (C-6), 137.1 (C-3), 123.1 (C-7), 123.0 (C-4), 120.3 (C-2), 50.0 (C-8), 39.6 (C-9), 23.3 (C-12);

HRMS (ESI+) $(C_{11}H_{13}N_5NaO)^+$ m/z (calculated) 254.1012, (found) 254.1015, mass difference 1.1 ppm;

LCMS retention time (ESI+, 7 min method, H_2O:MeCN 0.1% formic acid): 0.67 min, m/z 232.09; 0.98 min, m/z 232.05.

M12Y3

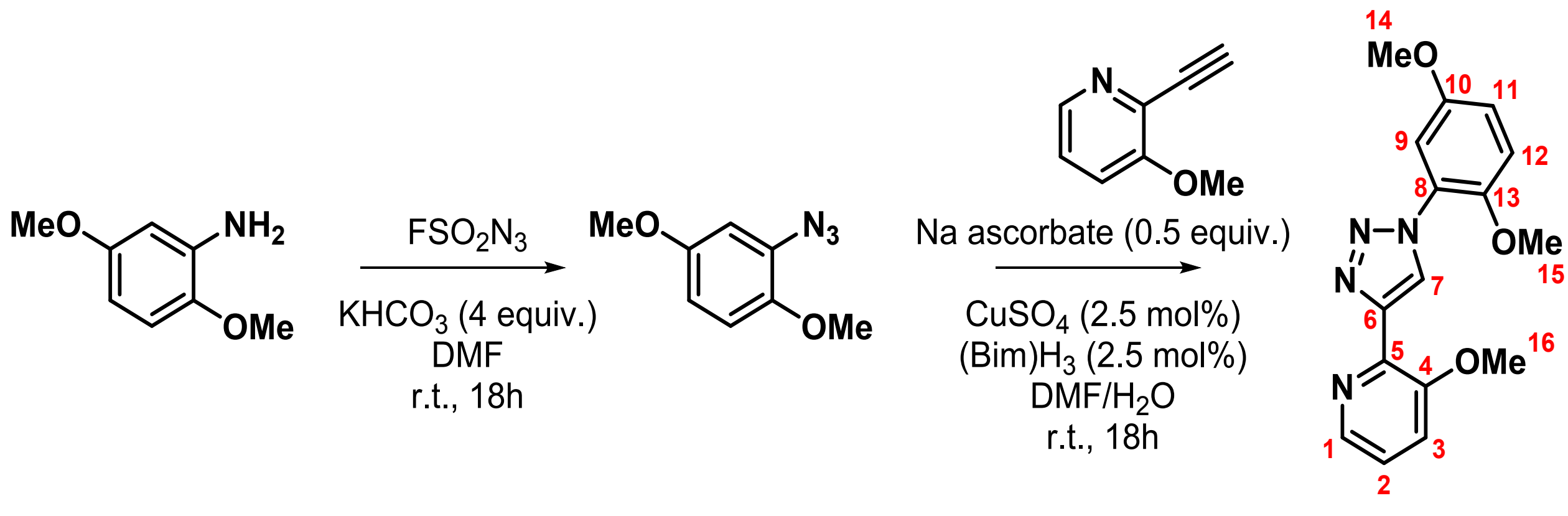

M12Y3

Column chromatography in MeOH/DCM (0% - 5% gradient), eluting at 3% MeOH.

Brown oil (solvato-chromic compound), 84 mg (48% yield), R_f = 0.30 (100% EtOAc);

^{1}H NMR (400 MHz, $CDCl_3$) δ 8.67 (s, 1H, H-7), 8.39 (d, J = 4.5 Hz, 1H, H-1), 7.41 (d, J = 3.0 Hz, 1H, H-9), 7.30 (d, J = 8.3 Hz, 1H, H-3), 7.25 (d, J = 4.6 Hz, 1H, H-2), 7.00 (d, J = 9.1 Hz, 1H, H-12), 6.94 (dd, J = 9.2, 3.0 Hz, 1H, H-11), 3.95 (s, 3H, H-16), 3.81 (s, 3H, H-14/15), 3.80 (s, 3H, H-14/15);

^{13}C NMR (101 MHz, $CDCl_3$) δ 154.0 (C-1), 152.7 (C-14), 145.4 (C-7), 143.6 (C-16), 141.8 (C-5), 139.4 (C-13), 126.8 (C-2), 123.2 (C-6), 118.3 (C-8), 115.8 (C-3), 113.9 (C11,12), 110.9 (C-9), 56.8 (OCH_3), 56.1 (OCH_3), 55.7 (OCH_3);

HRMS (ESI+) $(C_{16}H_{16}N_4NaO_3)^+$ m/z (calculated) 335.1115, (found) 335.1117, mass difference 0.6 ppm;

LCMS retention time (ESI+, 7 min method, H_2O:MeCN 0.1% formic acid): 2.57 min, m/z 313.11.

M18Y4

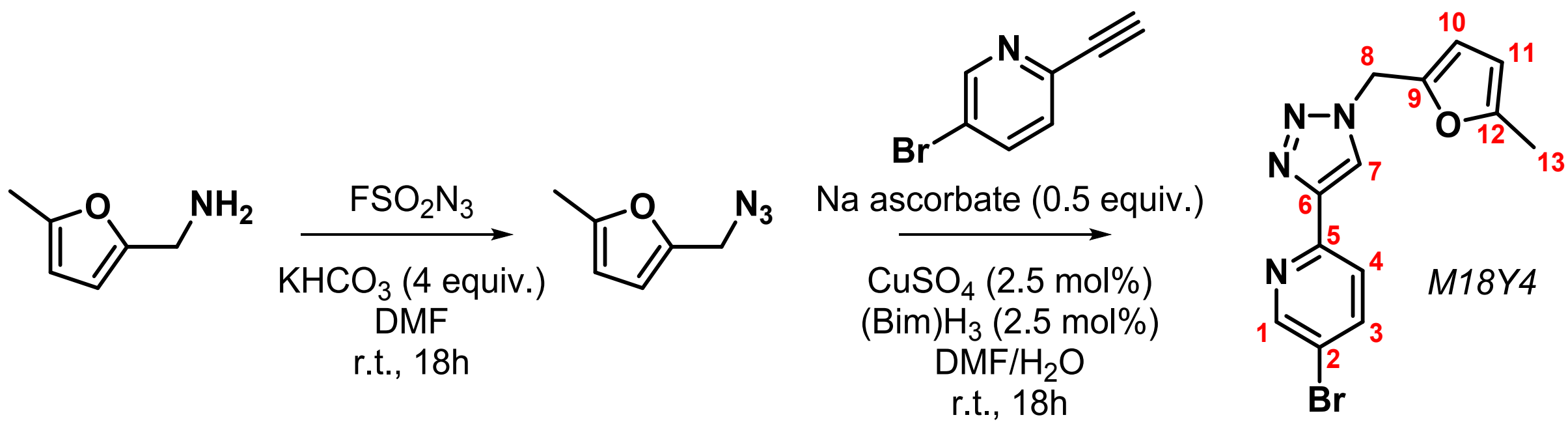

Column chromatography in EtOAc/Hexane (0% - 40% gradient), eluting at 23% EtOAc.

White solid, 143 mg (80% yield), R_f = 0.53 (40% EtOAc);

^{1}H NMR (400 MHz, C*D*Cl$_3$) δ 8.60 (dd, *J* = 2.3, 0.6 Hz, 1H, H-1), 8.12 (s, 1H, H-7), 8.07 (dt, *J* = 8.5, 0.6 Hz, 1H, H-3), 7.88 (dd, *J* = 8.5, 2.4 Hz, 1H, H-4), 6.37 (d, *J* = 3.1 Hz, 1H, H-10), 5.96 (dd, *J* = 3.2, 1.2 Hz, 1H, H-11), 5.51 (s, 2H, H-8), 2.27 (s, 3H, H-13);

^{13}C NMR (101 MHz, C*D*Cl$_3$) δ 154.0 (C-5), 150.5 (C-1), 148.9 (C-12), 147.7 (C-9), 145.1 (C-3), 139.7 (C-4), 122.1 (C-6), 121.6 (C-7), 119.6 (C-2), 111.8 (C-10), 107.0 (C-11), 47.2 (C-8), 13.7 (C-14);

HRMS (ESI+) $(C_{13}H_{11}BrN_4NaO)^+$ m/z (calculated) 341.0008, (found) 341.0009, mass difference 0.2 ppm;

LCMS retention time (ESI+, 7 min method, H_2O:MeCN 0.1% formic acid): 3.73 min, m/z 318.98.

2.6 Batch synthesis of IrCN-Schiff complexes

General procedure

To a 7 mL glass vial, IrCN dimer (1 equiv.), aldehyde (2 equiv.) and amine (2 equiv.) were added. DCM (1.5 mL) and MeOH (1.5 mL) was added, and the resulting solution was stirred at room temperature overnight. LCMS of the reaction mixture was taken to confirm product formation, then the solvent was removed *in vacuo*. The residue was purified by flash column chromatography (Combiflash, 12 g SiO_2 cartridge, 0 – 15% MeOH in DCM over 25 mins). Sample loaded 'wet' in DCM (approx. 2 mL) to obtain the pure compound.

IrCN1(P1A3)

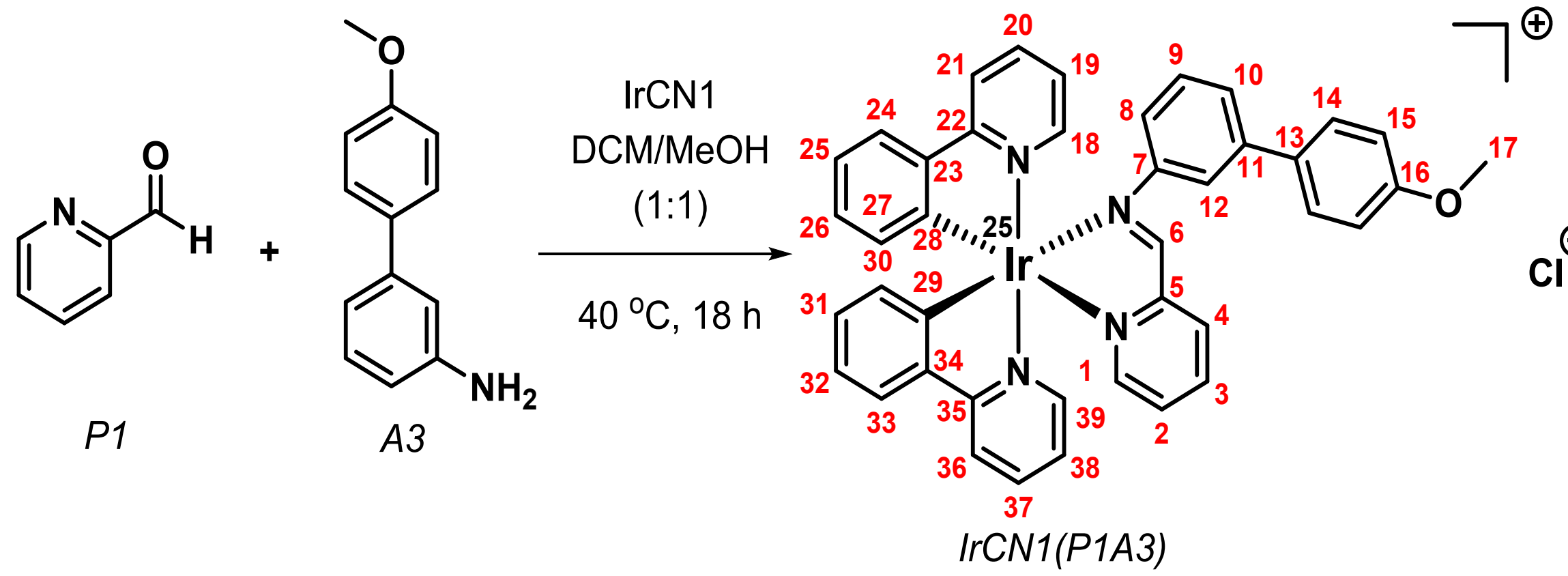

Red solid, 45 mg (73% yield);

^{1}H NMR (700 MHz, CD_2Cl_2) δ 10.58 (s, 1H, H-6), 9.30 (d, *J* = 7.6 Hz, 1H, H-27), 8.40 (d, *J* = 6.3 Hz, 1H, H-30), 8.14 (t, *J* = 7.3 Hz, 1H, H-26), 7.95 (dt, *J* = 8.2, 1.0 Hz, 1H, H-1), 7.87 (dd, *J* = 5.4, 1.4 Hz, 1H, H-24), 7.83 (m, 2H, H-33, 2), 7.81 – 7.77 (m, 1H, H-32), 7.71 (dd, *J* = 7.9, 1.3 Hz, 1H, H-18), 7.53 (dd, *J* = 7.9, 1.3 Hz, 1H, H-39), 7.51 – 7.49 (m, 1H, H-4), 7.45 (dd, *J* = 7.7, 5.4 Hz, 1H, H-25), 7.36 (dt, *J* = 6.5, 1.8 Hz, 1H, H-9), 7.23 – 7.17 (m, 5H, H-14, 8, 10, 12), 7.16 (ddd, *J* = 7.4, 5.8, 1.4 Hz, 1H, H-31), 7.09 – 7.03 (m, 2H, H-19, 3), 6.93 – 6.85 (m, 4H, H-20, 38, 15), 6.78 (td, *J* = 7.4, 1.3 Hz, 1H, H-37), 6.22 (ddd, *J* = 7.6, 2.7, 1.2 Hz, 2H, H-36, H21), 3.82 (s, 3H, H-17);

^{13}C NMR (176 MHz, CD_2Cl_2) δ 170.4 (C-6), 168.2 (C-28), 167.6 (C-29), 160.0 (C-23), 157.0 (C-34), 151.1 (C-30), 150.9 (C-22), 150.5 (C-24), 149.3 (C-4), 148.8 (C-35), 148.6 (C-5), 144.0 (C-7), 143.7 (C-11), 142.0 (C-13), 139.8 (C-26), 138.6 (C-32), 138.6 (C-33), 133.2 (C-27), 132.3 (C-16), 132.0 (C-36), 131.6 (C-21), 131.0 (C-20), 130.7 (C-37), 129.8 (C-10), 129.7 (C-25), 128.7 (C-14), 127.4 (C-9), 125.1 (C-18), 124.9 (C-39), 124.2 (C-31), 123.6 (C-19), 123.3 (C-3), 122.4 (C-38), 122.2 (C-8), 121.3 (C-12), 120.2 (C-1), 119.9 (C-2), 114.4 (C-15), 46.1 (C-17);

HRMS (ESI+) $(C_{41}H_{32}IrN_4O)^+$ m/z (calculated) 789.2200, (found) 789.2239, mass difference 4.7 ppm;

LCMS retention time (ESI+, 7 min method, H_2O:MeCN 0.1% formic acid): 3.25 min, m/z 789.37.

IrCN1(P8A1)

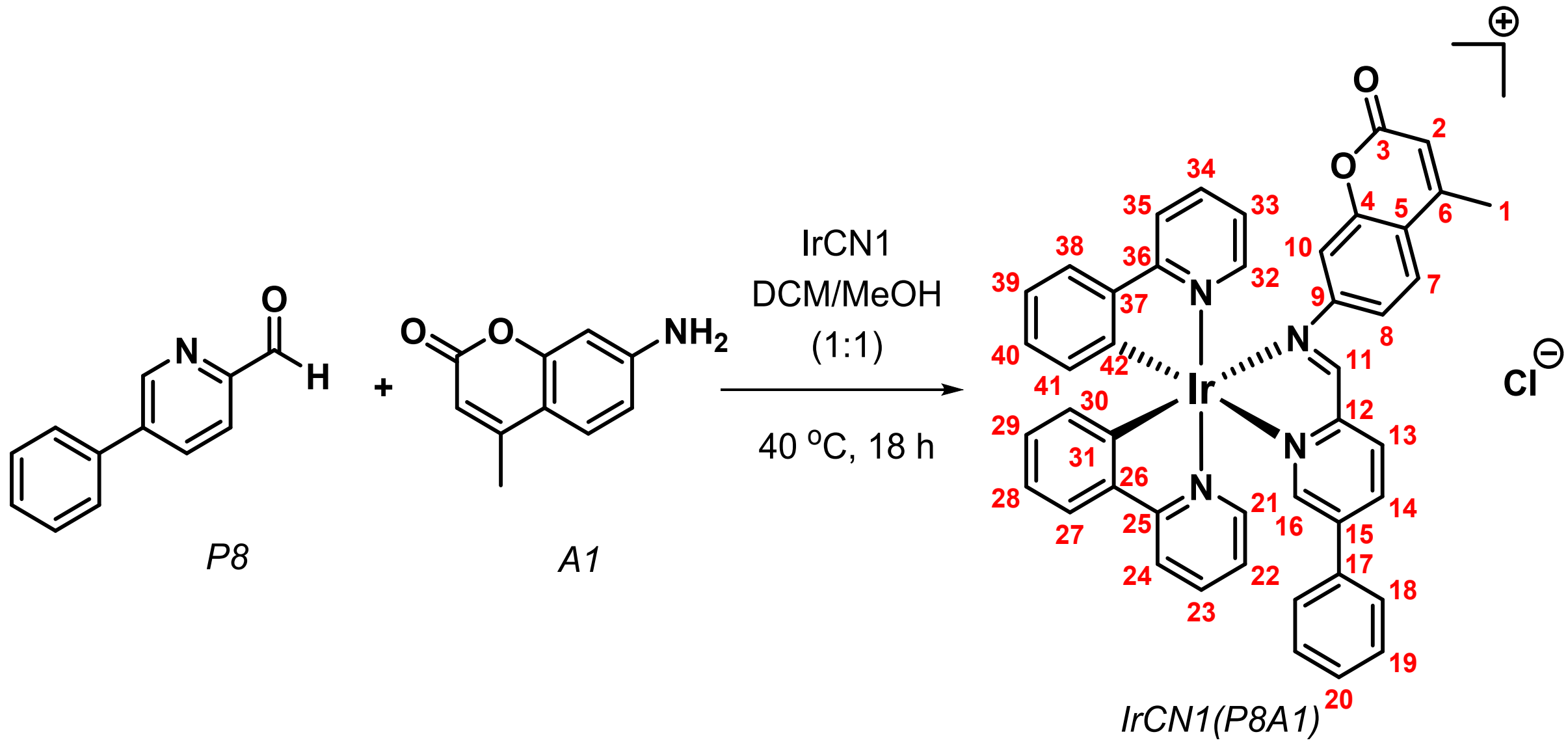

Dark red solid, 60 mg (92% yield);

^{1}H NMR (700 MHz, CD_2Cl_2) δ 10.77 (s, 1H, H-11), 9.37 (d, J = 8.1 Hz, 1H, H-7), 8.46 (d, J = 5.9 Hz, 1H, H-41), 8.33 (dd, J = 8.1, 2.1 Hz, 1H, H-8), 8.11 (d, J = 2.1 Hz, 1H, H-10), 7.98 (d, J = 8.2 Hz, 1H, H-38), 7.83 (m, 3H, H-39, 30, 28), 7.75 (dd, J = 7.9, 1.4 Hz, 1H, H-32), 7.59 (d, J = 5.7 Hz, 1H, H-27), 7.50 (d, J = 7.8 Hz, 1H, H-21), 7.46 – 7.39 (m, 4H, H-18, 14, 16), 7.35 (ddd, J = 7.0, 5.1, 2.8 Hz, 3H, H-19, 13), 7.22 (ddd, J = 7.3, 5.8, 1.4 Hz, 1H, H-40), 7.12 – 7.06 (m, 2H, H-33, 29), 6.99 (d, J = 2.1 Hz, 1H, H-20), 6.94 (td, J = 7.5, 1.3 Hz, 1H, H-34), 6.90 (td, J = 7.5, 1.3 Hz, 1H, H-22), 6.85 (td, J = 7.4, 1.4 Hz, 1H, H-23), 6.27 (ddd, J = 7.2, 5.7, 1.2 Hz, 2H, H-35, 24), 6.16 (q, J = 1.4 Hz, 1H, H-2), 2.32 (d, J = 1.4 Hz, 3H, H-1);

^{13}C NMR (176 MHz, CD_2Cl_2) δ 171.6 (C-11), 168.1 (C-42), 167.5 (C-31), 160.3 (C-3), 155.2 (C-4), 153.6 (C-5), 152.2 (C-6), 151.3 (C-41), 150.4 (C-37), 150.3 (C-26), 149.4 (C-27), 148.7 (C-10), 148.5 (C-36), 144.0 (C-25), 143.6 (C-9), 142.6 (C-12), 138.7 (C-39), 138.7 (C-30), 137.0 (C-8), 135.0 (C-15), 133.7 (C-7), 132.0 (C-35), 131.7 (C-24), 131.1 (C-34), 130.8 (C-23), 130.4 (C-16), 129.9 (C-18), 127.5 (C-19), 125.8 (C-14), 125.2 (C-32), 124.9 (C-21), 124.4 (C-40), 123.7 (C-33), 123.4 (C-29), 122.8 (C-22), 120.3 (C-38), 120.3 (C-7), 119.9 (C-28), 119.3 (C-13), 115.7 (C-2), 112.6 (C-20), 18.8 (C-1);

HRMS (ESI+) $(C_{44}H_{32}IrN_4O_2)^+$ m/z (calculated) 841.2149, (found) 841.2172, mass difference 2.4 ppm;

LCMS retention time (ESI+, 7 min method, H_2O:MeCN 0.1% formic acid): 3.20 min, m/z 841.38.

IrCN2(P3A2)

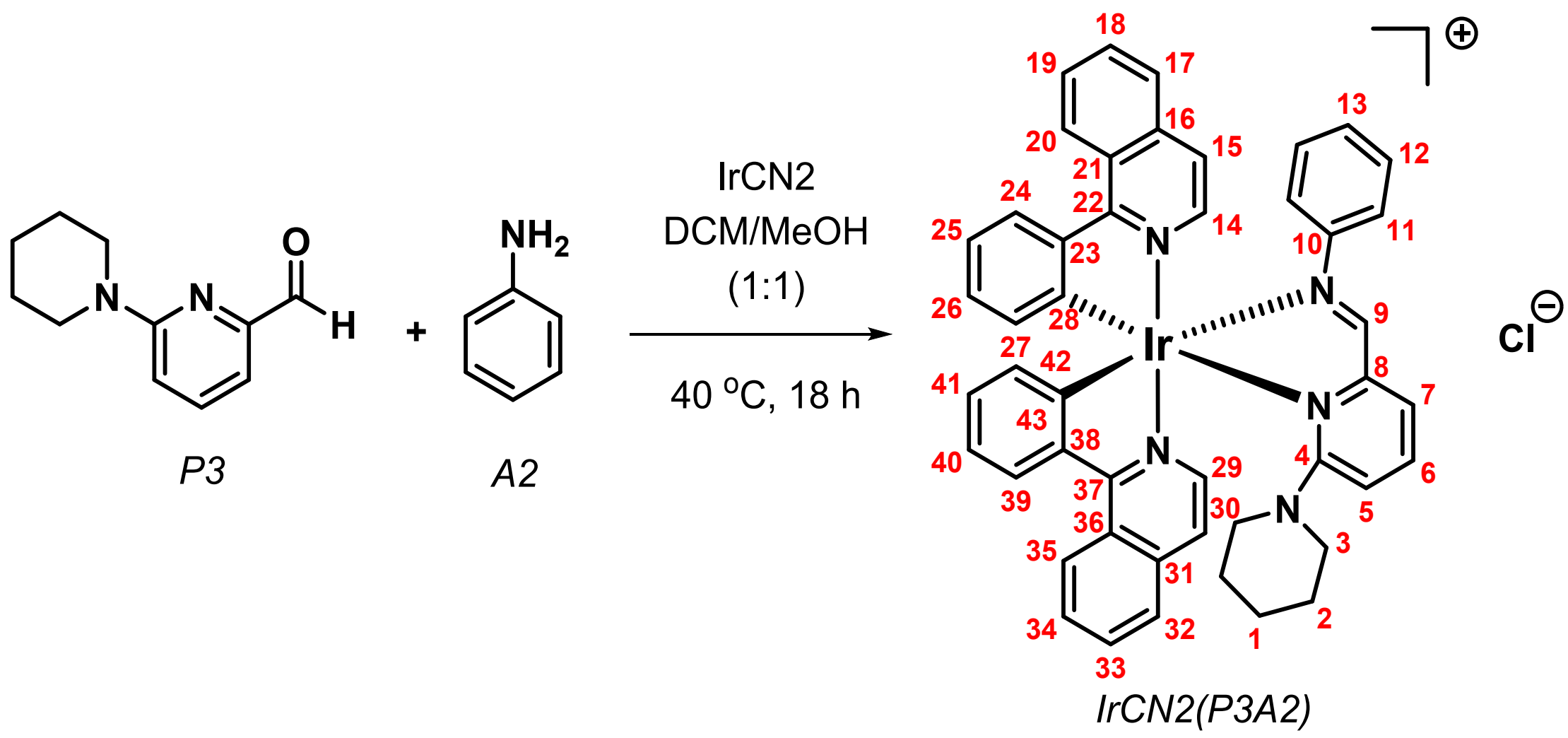

Red solid, 20 mg (40% yield);

^{1}H NMR (700 MHz, CD_2Cl_2) δ 9.80 (s, 1H, H-9), 8.97 – 8.91 (m, 1H, H-27), 8.70 (d, *J* = 8.6 Hz, 1H, H-42), 8.22 (dd, *J* = 11.1, 7.2 Hz, 2H, H-17, 14), 8.14 (d, *J* = 6.3 Hz, 1H, H-29), 8.03 – 7.97 (m, 2H, H-24, 39), 7.96 – 7.91 (m, 1H, H-25), 7.84 – 7.77 (m, 4H, H-32, 26, 40, 7), 7.74 (ddd, *J* = 8.5, 7.0, 1.4 Hz, 1H, H-41), 7.68 (dd, *J* = 8.7, 7.1 Hz, 1H, H-6), 7.56 (d, *J* = 6.3 Hz, 1H, H-15), 7.40 (d, *J* = 6.3 Hz, 1H, H-30), 7.02 (ddd, *J* = 8.1, 7.0, 1.3 Hz, 1H, H-18), 6.97 – 6.91 (m, 1H, H-13), 6.87 (ddd, *J* = 8.2, 7.1, 1.3 Hz, 1H, H-33), 6.79 (t, *J* = 7.9 Hz, 2H, H-12), 6.75 (d, *J* = 8.7 Hz, 1H, H-5), 6.72 (td, *J* = 7.4, 1.3 Hz, 1H, H-19), 6.67 (td, *J* = 7.4, 1.3 Hz, 1H, H-34), 6.52 – 6.48 (m, 2H, H-11), 6.40 (dd, *J* = 7.7, 1.3 Hz, 1H, H-20), 6.36 (dd, *J* = 7.8, 1.3 Hz, 1H, H-35), 2.93 (ddd, *J* = 13.3, 7.6, 3.5 Hz, 2H, H-3'), 2.55 (ddd, *J* = 13.3, 7.5, 3.5 Hz, 2H, H-3), 1.32 (p, *J* = 5.8 Hz, 2H, H-2'), 1.24 – 1.17 (m, 2H, H-2), 1.14 (ddp, *J* = 10.9, 7.5, 3.6 Hz, 2H, H-1);

^{13}C NMR (176 MHz, CD_2Cl_2) δ 170.9 (C-9), 169.7 (C-28), 169.0 (C-43), 164.2 (C-23), 155.6 (C-38), 153.4 (C-22), 149.2 (C-37), 147.4 (C-21), 146.0 (C-36), 144.8 (C-16), 142.4 (C-14), 141.1 (C-29), 139.6 (C-6), 137.4 (C-16), 137.3 (C-31), 134.2 (C-20), 132.4 (C-35), 132.2 (C-32), 132.1 (C-26), 130.6 (C-40), 130.3 (C-34), 130.0 (C-17), 129.2 (C-5), 129.0 (C-41), 129.0 (C-7), 128.6 (C-12), 127.9 (C-13), 127.8 (C-25), 127.8 (C-39), 127.2 (C-42), 126.8 (C-27), 126.3 (C-4), 126.2 (C-10), 123.6 (C-24), 122.1 (C-11), 122.0 (C-33), 121.9 (C-18), 121.6 (C-15), 121.4 (C-30), 119.0 (C-5), 51.9 (C-3,3'), 24.6 (C-2'), 24.3 (C-1,2);

HRMS (ESI+) $(C_{47}H_{39}IrN_5)^+$ m/z (calculated) 866.2829, (found) 866.2832, mass difference 0.0 ppm;

LCMS retention time (ESI+, 7 min method, H_2O:MeCN 0.1% formic acid): 3.92 min, m/z 866.38.

IrCN2(P6A9)

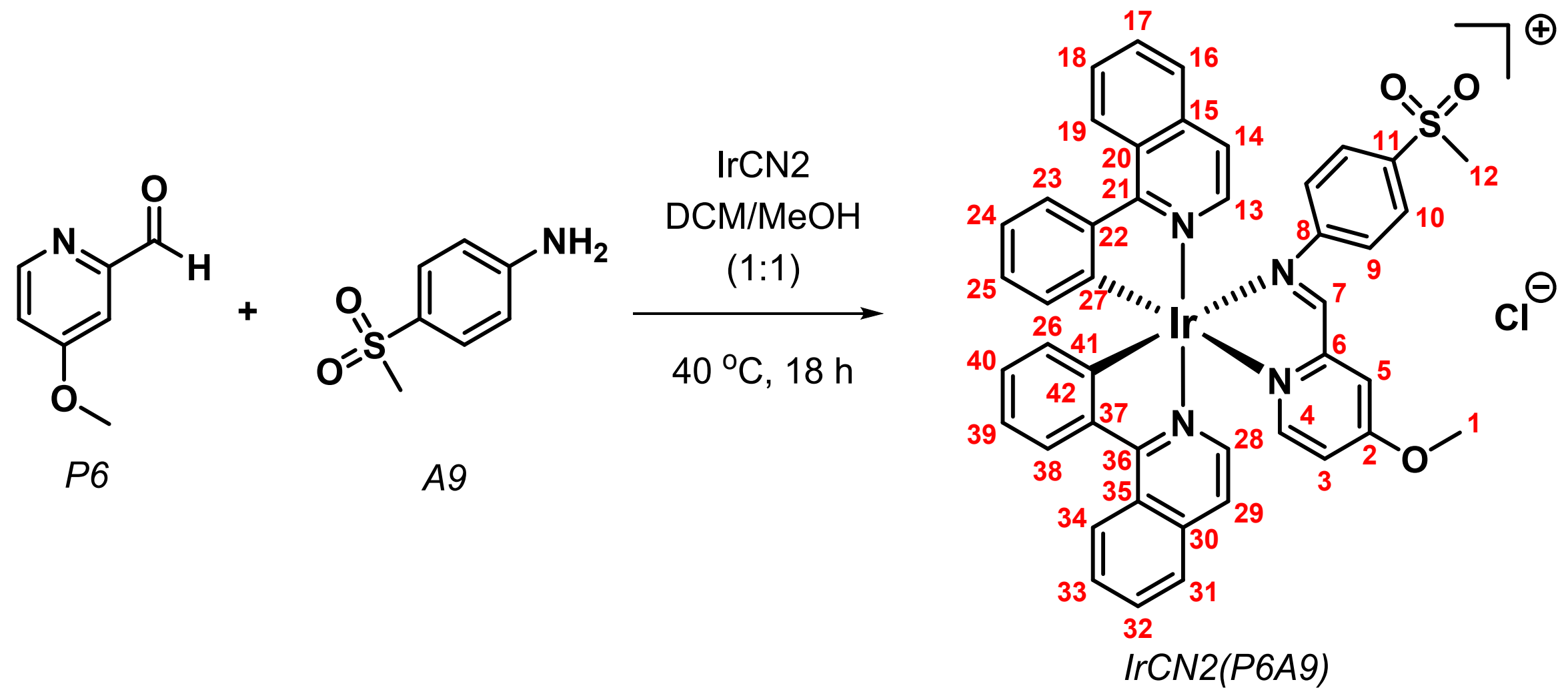

Red solid, 29 mg (57% yield);

^{1}H NMR (700 MHz, CD_2Cl_2) δ 10.70 (s, 1H, H-7), 9.36 – 9.30 (m, 1H, H-26), 8.97 (dq, *J* = 7.9, 0.9 Hz, 1H, H-41), 8.70 (d, *J* = 8.6 Hz, 1H, H-38), 8.33 – 8.26 (m, 2H, H-13, 16), 8.02 – 7.93 (m, 3H, H-23, 4, 5), 7.86 – 7.78 (m, 3H, H-40, 28, 29), 7.75 (ddd, *J* = 8.4, 6.9, 1.4 Hz, 1H, H-39), 7.57 (d, *J* = 6.2 Hz, 1H, H-14), 7.50 – 7.44 (m, 5H, H-24, 31, 10, 3), 7.12 (ddd, *J* = 8.2, 7.2, 1.3 Hz, 1H, H-17), 7.10 – 7.04 (m, 2H, H-9), 6.96 – 6.89 (m, 2H, H-25, H32), 6.87 (td, *J* = 7.5, 1.3 Hz, 1H, H-18), 6.71 (td, *J* = 7.4, 1.3 Hz, 1H, H-33), 6.26 (dd, *J* = 7.7, 1.3 Hz, 1H, H-19), 6.12 (dd, *J* = 7.7, 1.3 Hz, 1H, H-34), 4.09 (s, 3H, H-12), 2.89 (s, 3H, H-1);

^{13}C NMR (176 MHz, CD_2Cl_2) δ 172.4 (C-7), 169.5 (C-27), 168.9 (C-41), 168.5 (C-11), 158.2 (C-6, 153.5 (C-8), 152.0 (C-22), 151.7 (C-37), 151.0 (C-24), 145.8 (C-21), 145.6 (C-36), 142.2 (C-13), 140.8 (C-31), 140.1 (C-20), 137.5 (C-30), 137.4 (C-15), 132.5 (C-34), 132.3 (C-19), 132.3 (C-28), 132.2 (C-40), 131.1 (C-23), 131.0 (C-16), 130.8 (C-18), 130.7 (C-33), 129.2 (C-29), 129.2 (C-39), 128.3 (C-10), 127.9 (C-4), 127.8 (C-5), 127.2 (C-38, 41), 126.8 (C-35), 126.5 (C-2), 124.0 (C-9), 122.8 (C-17), 122.5 (C-14), 122.3 (C-25), 122.2 (C-3), 119.9 (C-26), 116.7 (C-32), 57.8 (C-12), 44.8 (C-1);

HRMS (ESI+) $(C_{44}H_{34}IrN_4O_3S)^+$ m/z (calculated) 891.1975, (found) 891.2011, mass difference 3.9 ppm;

LCMS retention time (ESI+, 7 min method, H_2O:MeCN 0.1% formic acid): 3.24 min, m/z 891.34.

IrCN3(P6A1)

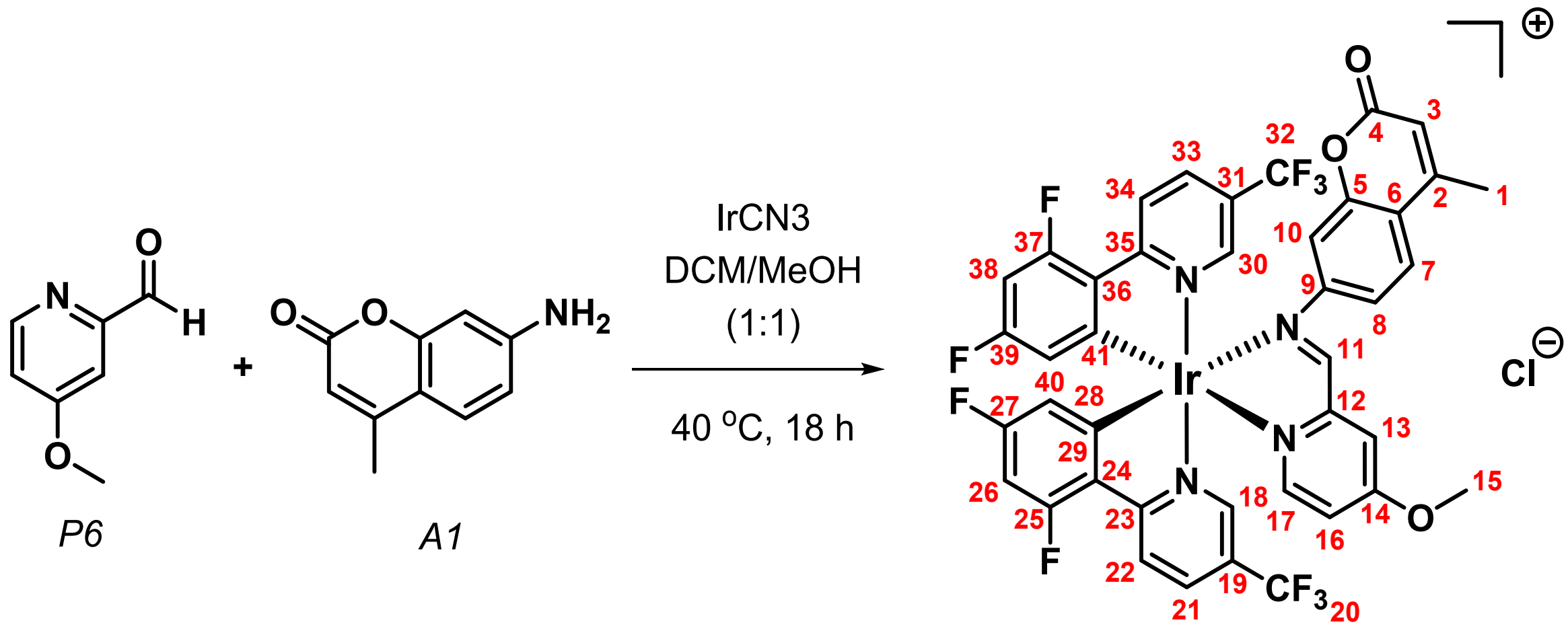

Orange solid, 35 mg (63% yield);

^{1}H NMR (700 MHz, CD_2Cl_2) δ 10.83 (s, 1H, H-11), 9.39 (d, *J* = 2.9 Hz, 1H, H-10), 8.61 (s, 1H, H-18), 8.49 (dd, *J* = 8.8, 2.8 Hz, 1H, H-22), 8.40 (dd, *J* = 8.8, 2.8 Hz, 1H, H-7), 8.13 (m, 2H, H-21,8), 7.70 (s, 1H, H-30), 7.62 (d, *J* = 6.3 Hz, 1H, H-34), 7.52 (d, *J* = 8.5 Hz, 1H, H-17), 7.29 (dd, *J* = 8.5, 2.2 Hz, 1H, H-16), 7.06 (dd, *J* = 6.3, 2.8 Hz, 1H, H-33), 6.80 (d, *J* = 2.2 Hz, 1H, H-13), 6.66 (ddd, *J* = 12.4, 8.9, 2.3 Hz, 1H, H-38), 6.50 (ddd, *J* = 12.5, 9.0, 2.3 Hz, 1H, H-26), 6.22 (q, *J* = 1.3 Hz, 1H, H-3), 5.62 (dd, *J* = 8.1, 2.3 Hz, 1H, H-40), 5.58 (dd, *J* = 8.2, 2.3 Hz, 1H, H-28), 4.14 (s, 3H, H-15), 2.36 (d, *J* = 1.4 Hz, 3H, H-1);

^{19}F NMR (659 MHz, CD_2Cl_2) δ -62.46 (s, 3F, CF_3-20), -62.89 (s, 3F, CF_3-32), -102.58 – -102.70 (m, 1F, F-37), -102.87 – -102.98 (m, 1F, F-25), -106.46 (dd, *J* = 12.6, 3.5 Hz, 1F, F-39), -106.82 – -106.96 (m, 1F, F-27);

Note: Due to the large and complex fluorine splitting pattern, it has not been possible to assign all of the carbon environments.

^{13}C NMR (176 MHz, CD_2Cl_2) δ 174.6 (C-11), 168.3 (d, *J* = 6.6 Hz, C-41), 167.8 (d, *J* = 6.5 Hz, C-29), 165.7 (d, *J* = 12.7 Hz), 164.2 (d, *J* = 12.6 Hz), 163.5 (d, *J* = 13.1 Hz), 162.1 – 161.9 (m), 159.9, 158.2, 155.1 (d, *J* = 7.0 Hz), 153.8, 152.5 (d, *J* = 7.2 Hz), 150.8 (C-34), 149.6, 147.8 (t, *J* = 4.5 Hz, C-18), 146.1 (d, *J* = 4.7 Hz, C-30), 137.3, 137.1 (d, *J* = 3.4 Hz, C-21), 127.0, 126.8, 126.7, 126.4 (C-17), 126.3, 124.2 (), 124.1, 124.0 (C-22), 123.9 (C-7), 123.0 (d, *J* = 14.0 Hz), 121.5 (d, *J* = 13.8 Hz), 121.0, 120.8 (C-10), 118.8 (C-16), 117.5 (C-33), 116.3 (C-3), 114.5 (d, *J* = 17.4 Hz, C-28), 114.3 (d, *J* = 19.4 Hz, C-40), 112.0, 100.3 (t, *J* = 26.7 Hz, C-38), 99.7 (t, *J* = 26.7 Hz, C-26), 58.1 (C-15), 18.8 (C-1);

HRMS (ESI+) $(C_{41}H_{24}F_{10}IrN_4O_3)^+$ m/z (calculated) 1003.1312, (found) 1003.1335, mass difference 2.0 ppm;

LCMS retention time (ESI+, 7 min method, H_2O:MeCN 0.1% formic acid): 3.38 min, m/z 1003.30.

2.7 Batch synthesis of IrCN-Click complexes

IrCN3(M2Y1)

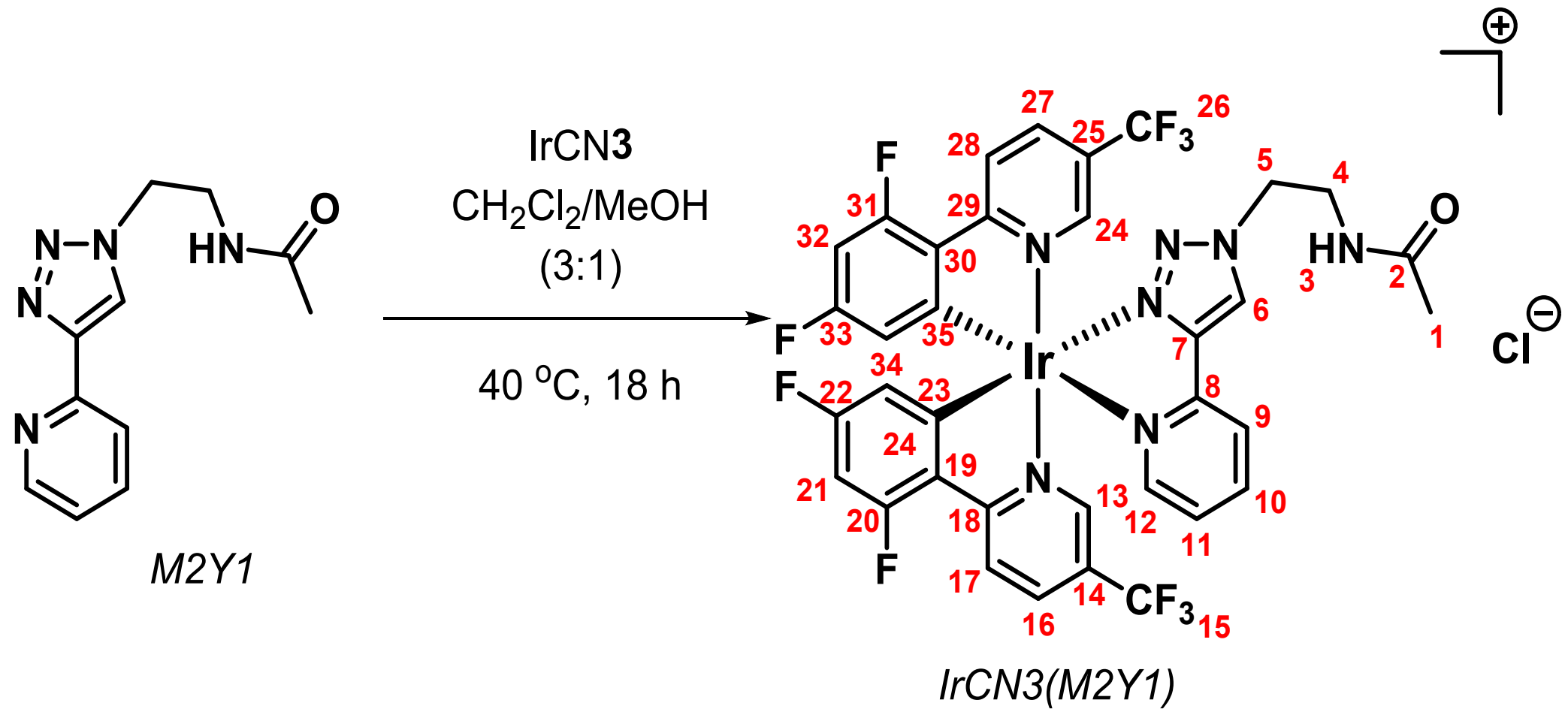

To a 20 mL glass vial, IrCN3 dimer (48 mg, 0.032 mmol, 1 equiv.) and M2Y1 (15 mg, 0.065 mmol, 2 equiv.) were added. DCM (3 mL) and MeOH (1 mL) were added, and the resulting solution was stirred rapidly at 40 °C for 18 h. The solvent was removed *in vacuo*, and the residue was purified by flash column chromatography (Combiflash, 4 g SiO_2 cartridge, 0 – 10% MeOH in DCM, with compound eluting at 7% MeOH as a broad peak). Sample loaded 'wet' in DCM (approx. 2 mL)) to obtain the pure compound.

Bright yellow solid, 55 mg, (87% yield);

^{1}H NMR (600 MHz, $CDCl_3$) δ 10.86 (s, 1H, H-6), 8.87 (d, *J* = 7.9 Hz, 1H, H-12), 8.65 (s, 1H, H-3), 8.47 (td, *J* = 8.5, 3.0 Hz, 2H, H-27, 16), 8.14 (td, *J* = 7.8, 1.6 Hz, 1H, H-11), 8.05 (td, *J* = 8.9, 2.1 Hz, 2H, H-28, 17), 7.88 (dd, *J* = 2.1, 1.1 Hz, 1H, H-24), 7.78 (dd, *J* = 5.6, 1.5 Hz, 1H, H-9), 7.51 (dd, *J* = 2.2, 1.1 Hz, 1H, H-13), 7.42 (ddd, *J* = 7.7, 5.5, 1.2 Hz, 1H, H-10), 6.65 (ddd, *J* = 12.4, 8.9, 2.3 Hz, 1H, H-32), 6.59 (ddd, *J* = 12.5, 8.9, 2.3 Hz, 1H, H-21), 5.69 (dd, *J* = 8.0, 2.3 Hz, 1H, H-34), 5.60 (dd, *J* = 8.2, 2.3 Hz, 1H, H-23), 4.74 (ddd, *J* = 9.9, 6.7, 3.6 Hz, 2H, H-5), 3.85 – 3.67 (m, 2H, H-4), 1.97 (s, 3H, H-1);

^{19}F NMR (565 MHz, $CDCl_3$) δ -62.53 (s, 3F, CF_3-26), -62.84 (s, 3F, CF_3-15), -101.61 (dt, *J* = 12.6, 8.5 Hz, 1F, F-33), -102.52 (dt, *J* = 12.2, 8.6 Hz, 1F, F-22), -106.16 (td, *J* = 12.4, 3.4 Hz, 1F, F-31), -106.92 (td, *J* = 12.4, 3.6 Hz, 1F, F-20);

Note: Due to a complex fluorine splitting pattern, it has not been possible to fully assign the ^{13}C NMR for this molecule.

^{13}C NMR (151 MHz, $CDCl_3$) δ 172.0 (C-2), 168.4, 167.7, 165.8, 165.2 (d, *J* = 12.8 Hz), 164.0 (d, *J* = 12.5 Hz), 163.4, 163.1, 161.7, 161.7, 154.4 (d, *J* = 6.8 Hz), 150.0 (C-9), 149.9, 149.7, 147.9 (C-24), 146.4, 144.8 (d, *J* = 4.7 Hz, C-13), 141.3 (C-11), 136.6 (C-28, 17), 129.5 (C-6), 126.9 (C-10), 126.7 (d, *J* = 4.4 Hz, C-12), 126.5 , 126.1, 125.9, 125.1, 123.7 (d, *J* = 21.0 Hz, C-27), 123.5 (d, *J* = 21.2 Hz, C-

16), 122.6 (d, *J* = 14.5 Hz), 120.8 (d, *J* = 14.9 Hz), 114.5 (C-34), 114.4 (C-23), 100.4 – 100.1 (m), 100.1 – 99.7 (m), 52.0 (C-5), 38.4 (C-4), 23.0 (C-1);

HRMS (ESI+) $(C_{35}H_{23}F_{10}IrN_7O)^+$ m/z (calculated) 940.1428, (found) 940.1443, mass difference 1.4 ppm;

LCMS retention time (ESI+, 7 min method, H_2O:MeCN 0.1% formic acid): 3.17 min, m/z 940.30.

IrCN3(M12Y3)

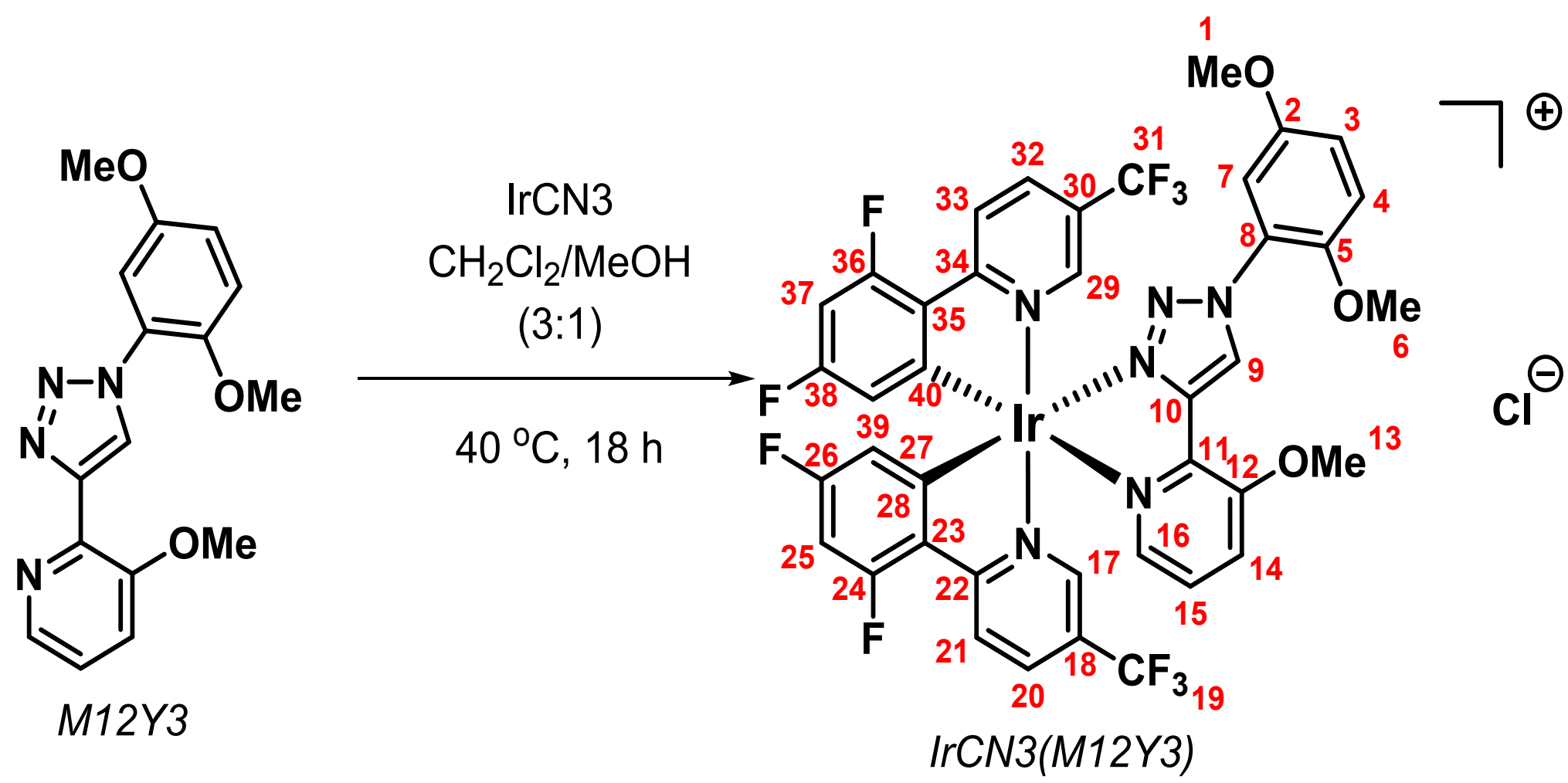

To a 20 mL glass vial, IrCN3 dimer (43 mg, 0.029 mmol, 1 equiv.) and M12Y3 (18 mg, 0.058 mmol, 2 equiv.) were added. DCM (3 mL) and MeOH (1 mL) were added, and the resulting solution was stirred rapidly at 40 °C for 18 h. The solvent was removed *in vacuo*, and the residue was purified by flash column chromatography (Combiflash, 4 g SiO_2 cartridge, 0 – 10% MeOH in DCM, with compound eluting at 7.4% MeOH as a broad peak). Sample loaded 'wet' in DCM (approx. 2 mL)) to obtain the pure compound.

Yellow solid, 33 mg, (54% yield);

^{1}H NMR (600 MHz, $CDCl_3$) δ 9.23 (s, 1H, H-9), 8.48 (dt, *J* = 8.1, 3.8 Hz, 2H, H-16, 15), 8.40 (dd, *J* = 8.9, 1.0 Hz, 1H, H-32), 8.08 (dd, *J* = 8.8, 2.1 Hz, 2H, H33, 21), 7.82 (dd, *J* = 8.8, 5.3 Hz, 1H, H-20), 7.68 (d, *J* = 1.0 Hz, 1H, H-29), 7.46 (dd, *J* = 5.3, 1.0 Hz, 1H, H-14), 7.11 – 7.02 (m, 3H, H-3,4,17), 6.65 (ddd, *J* = 12.3, 8.9, 2.3 Hz, 1H, H-37), 6.55 (ddd, *J* = 12.4, 8.9, 2.3 Hz, 1H, H-25), 5.64 (ddd, *J* = 10.5, 8.1, 2.3 Hz, 2H, H-39, 27), 4.40 (s, 3H, H-13), 3.86 (s, 3H, H-1/6), 3.79 (s, 3H, H1/6);

^{19}F NMR (565 MHz, $CDCl_3$) δ -62.69 (s, 3F, CF_3-31), -62.72 (s, 3F, CF_3-19), -101.34 – -101.40 (m, 1F, F-38), -102.57 – -102.64 (m, 1F, F-26), -105.72 – -105.77 (m, 1F, F-36), -106.92 – -106.97 (m, 1F, F-24);

Note: Due to lack of 2D NMR spectra and a complex fluorine splitting pattern, it has not been possible to assign the ^{13}C NMR for this molecule.

^{13}C NMR (151 MHz, $CDCl_3$) δ 168.6, 167.9, 165.8, 165.2 (d, *J* = 12.6 Hz), 164.0 (d, *J* = 12.6 Hz), 163.5 (d, *J* = 12.7 Hz), 163.2 (d, *J* = 12.9 Hz), 161.7 (d, *J* = 12.8 Hz), 161.5 (d, *J* = 13.0 Hz), 155.2, 154.1 (d, *J* = 6.9 Hz), 153.8, 150.7 (d, *J* = 7.4 Hz), 146.4, 146.1, 145.5, 145.2, 141.8, 138.0, 136.7, 136.6 (d, *J* = 3.2 Hz), 130.4, 129.9, 126.6 (d, *J* = 2.8 Hz), 126.4, 126.2, 126.1, 125.8, 124.8, 124.7, 124.5, 123.7 (t, *J* = 20.3 Hz), 122.7 (d, *J* = 7.5 Hz), 120.9 (d, *J* = 7.3 Hz), 117.6, 114.6 – 114.3 (m), 114.2 (d, *J* = 18.2 Hz), 114.0, 112.3, 100.4 (t, *J* = 26.5 Hz), 99.8 (t, *J* = 26.6 Hz), 58.5 (C-13), 56.9 (C-1/6), 56.3 (C-1/6);

HRMS (ESI+) $(C_{40}H_{26}F_{10}IrN_6O_3)^+$ m/z (calculated) 1021.1530, (found) 1021.1580, mass difference 4.6 ppm;

LCMS retention time (ESI+, 7 min method, H_2O:MeCN 0.1% formic acid): 3.70 min, m/z 1021.31.

IrCN2(M18Y4)-PF_6

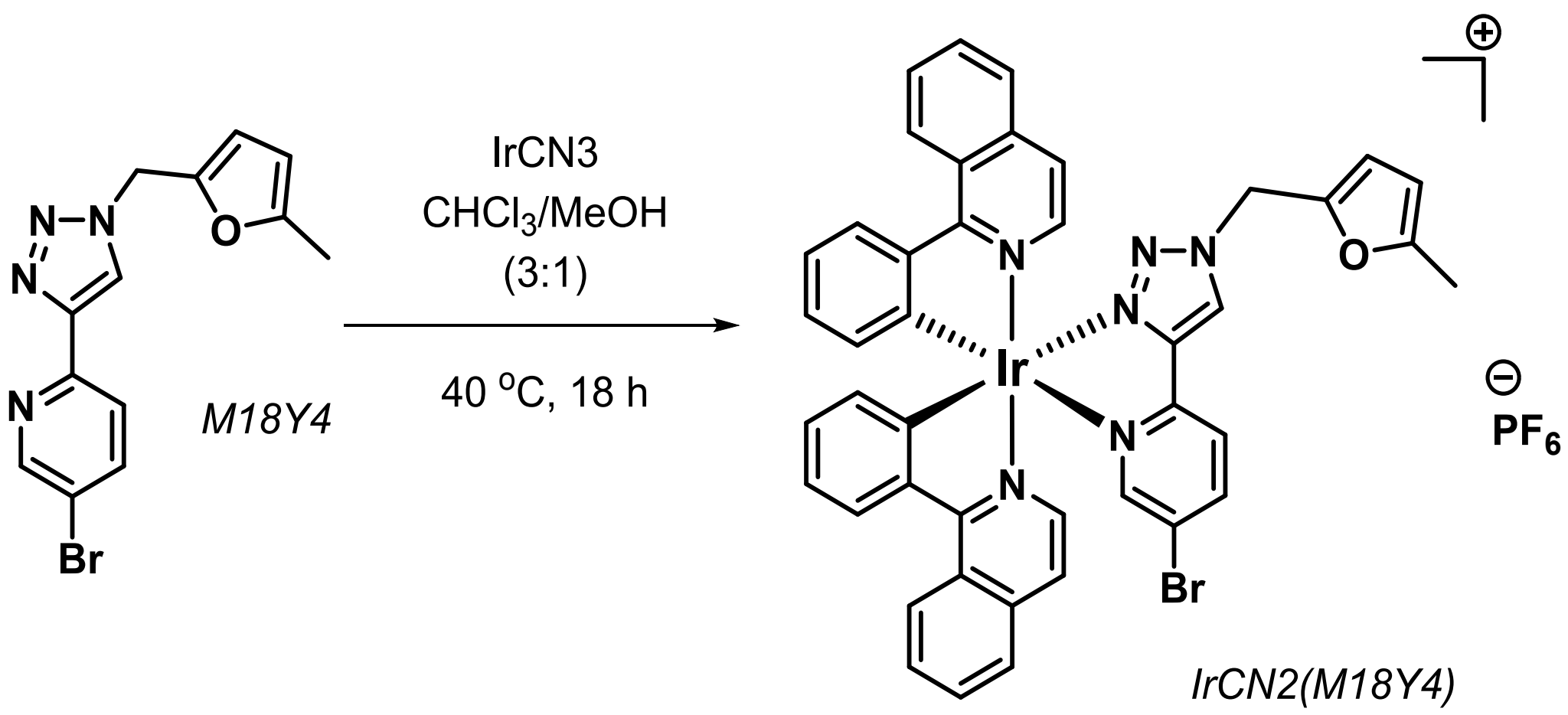

To a 20 mL glass vial, IrCN2 dimer (9 mg, 0.007 mmol, 1 equiv.) and M18Y4 (6.5 mg, 0.02 mmol, 3 equiv.) were added. DCM (3 mL) and MeOH (3 mL) were added, and the resulting solution was stirred rapidly at 40 °C overnight. The solvent was removed *in vacuo*, and the residue was redissolved in the minimum volume of MeOH and precipitated with an excess of sat. KPF_6 solution. The precipitate was collected by centrifugation, washed (5 x 2 mL H_2O, 3 x 2 mL Et_2O) and dried under a stream of nitrogen.

Red solid, 6.6 mg, (43% yield);

Note: This compound proved to be extremely unstable with respect to acid or acidic environments such as silica, or in solution in general. As such, although it was possible to obtain a ^1H and ^{13}C NMR, further 2D NMR analysis and assignment was not possible with this complex. Additionally, over time this compound degraded in the DCM-d_2 solvent.

^1H NMR (400 MHz, CD_2Cl_2) δ 8.99 (q, *J* = 5.3 Hz, 2H), 8.65 (s, 1H), 8.33 (t, *J* = 8.0 Hz, 2H), 8.13 (dd, *J* = 3.8, 1.7 Hz, 2H), 7.99 – 7.91 (m, 2H), 7.81 (dq, *J* = 9.8, 4.1 Hz, 4H), 7.63 (d, *J* = 1.4 Hz, 1H), 7.53 (d, *J* = 6.4 Hz, 1H), 7.46 – 7.40 (m, 2H), 7.36 (d, *J* = 6.5 Hz, 1H), 7.21 – 7.15 (m, 1H), 7.14 – 7.07 (m,

1H), 7.00 – 6.91 (m, 1H), 6.84 (td, *J* = 7.4, 1.3 Hz, 1H), 6.46 (d, *J* = 3.2 Hz, 1H), 6.41 (d, *J* = 7.5 Hz, 1H), 6.27 (dd, *J* = 7.7, 1.3 Hz, 1H), 5.97 (d, *J* = 3.2 Hz, 1H), 5.59 – 5.44 (m, 2H), 2.20 (s, 2H).

^{13}C NMR (176 MHz, CD_2Cl_2) δ 169.32, 168.94, 155.13, 152.30, 151.77, 150.85, 148.83, 148.41, 148.29, 146.09, 143.62, 142.77, 141.44, 140.76, 137.64, 137.51, 132.70, 132.57, 132.29, 132.19, 131.24, 131.22, 130.70, 130.32, 129.26, 129.12, 127.93, 127.79, 127.34, 127.24, 126.82, 126.76, 126.28, 124.28, 123.14, 123.08, 122.55, 122.13, 121.56, 113.56, 107.34, 49.23, 13.61.

HRMS (ESI+) $(C_{43}H_{31}BrIrN_6O)^+$ m/z (calculated) 919.1366, (found) 919.1391, mass difference 4.1 ppm.

LCMS retention time (ESI+, 7 min method, H_2O:MeCN 0.1% formic acid): 4.43 min, m/z 919.40.

3. Photophysical Properties

A stock solution of each pure compound in DMSO (5 mM) was diluted to a concentration of 31.25 µM with DMSO polystyrene 96 well plate (200 µL). The UV-Vis absorbance (270 – 700 nm, 10 flashes) and fluorescence (λ_{ex} = 380 nm, λ_{em} = 420 – 700 nm, 10 flashes) of these compounds were then measured using a plate reader. Spectra for IrCN1(M8Y4) is included for reference.[2]

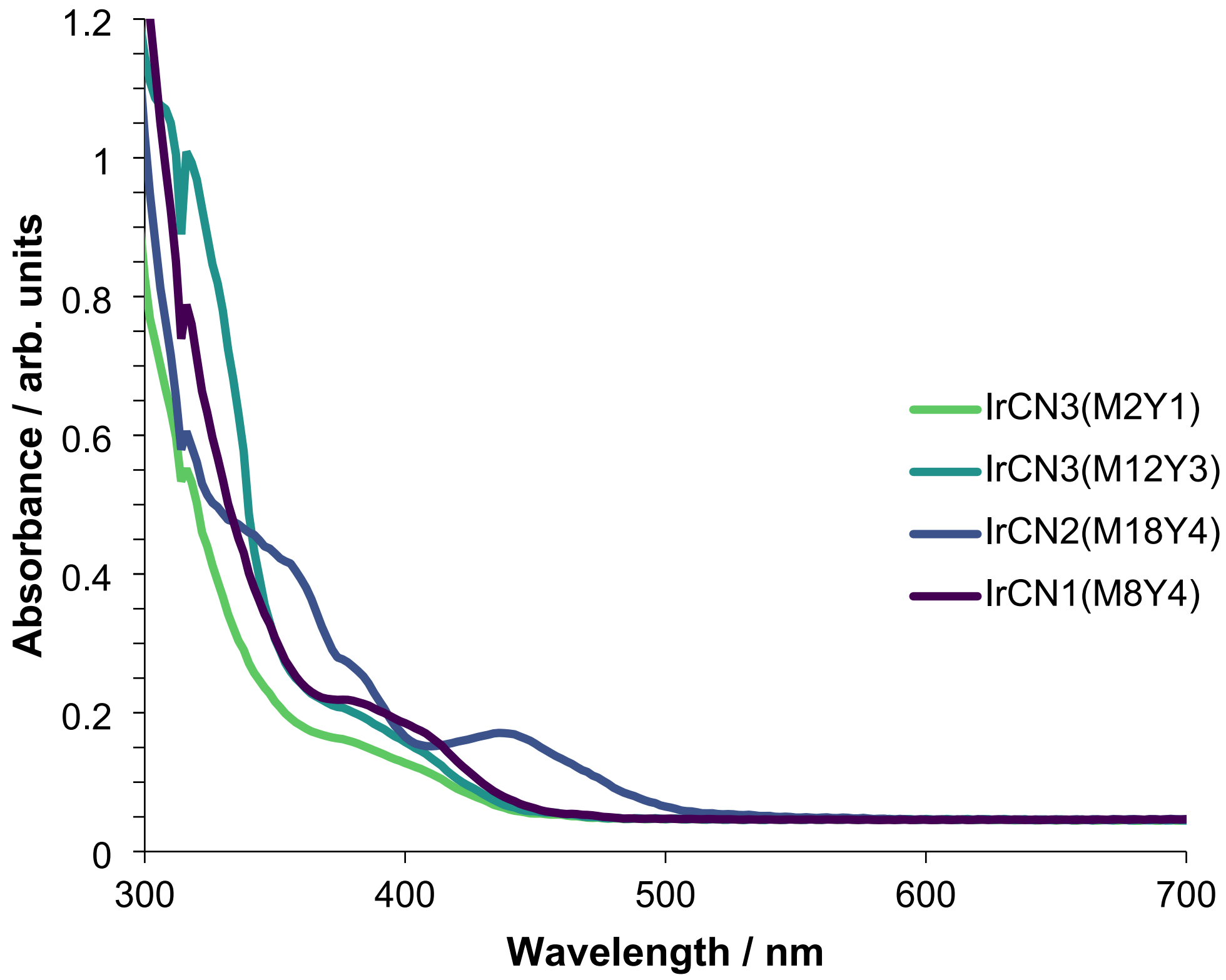

Figure S1: Absorbance spectra of pure IrCN Click compounds

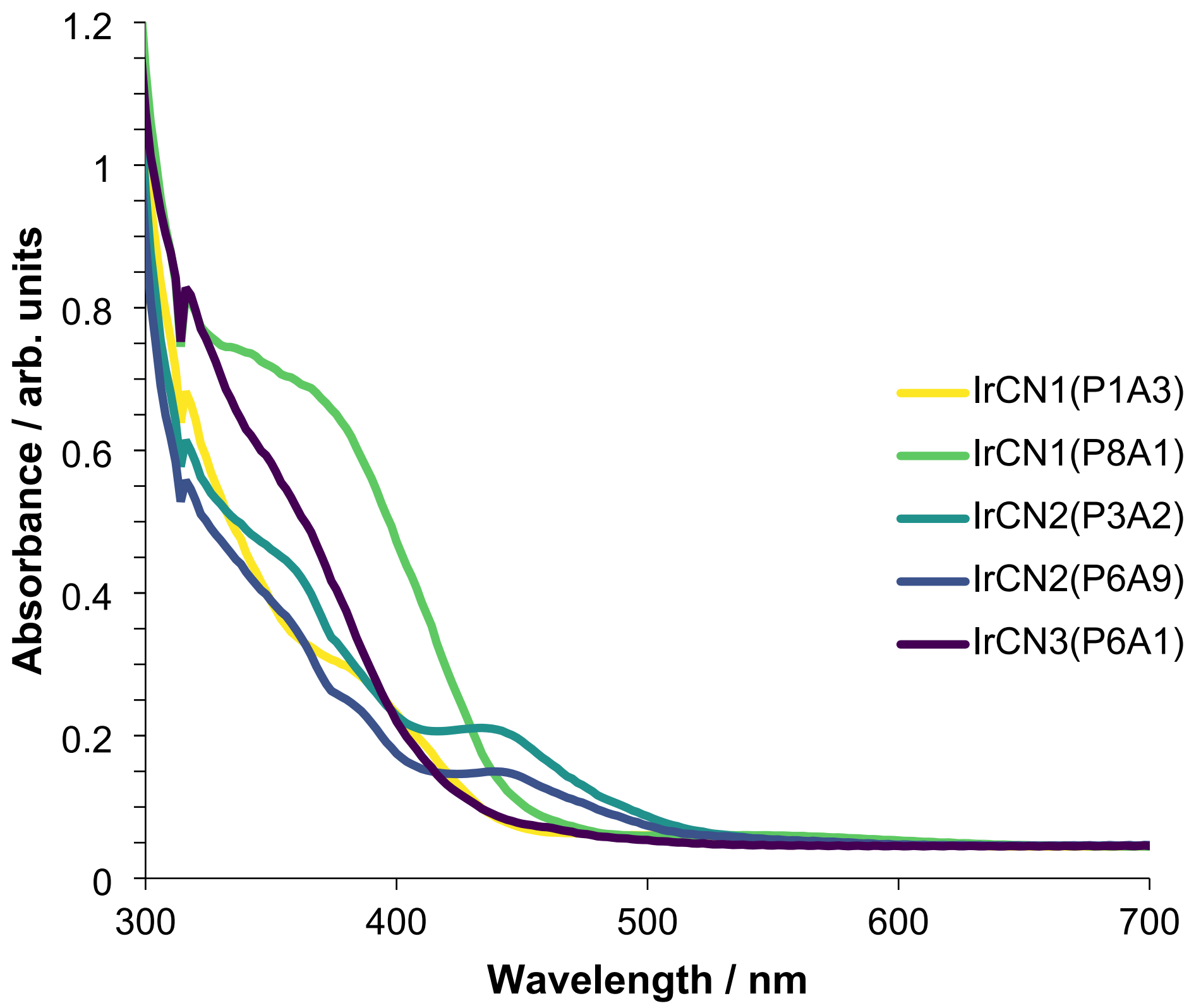

Figure S2: Absorbance spectra of pure IrCN Schiff compounds

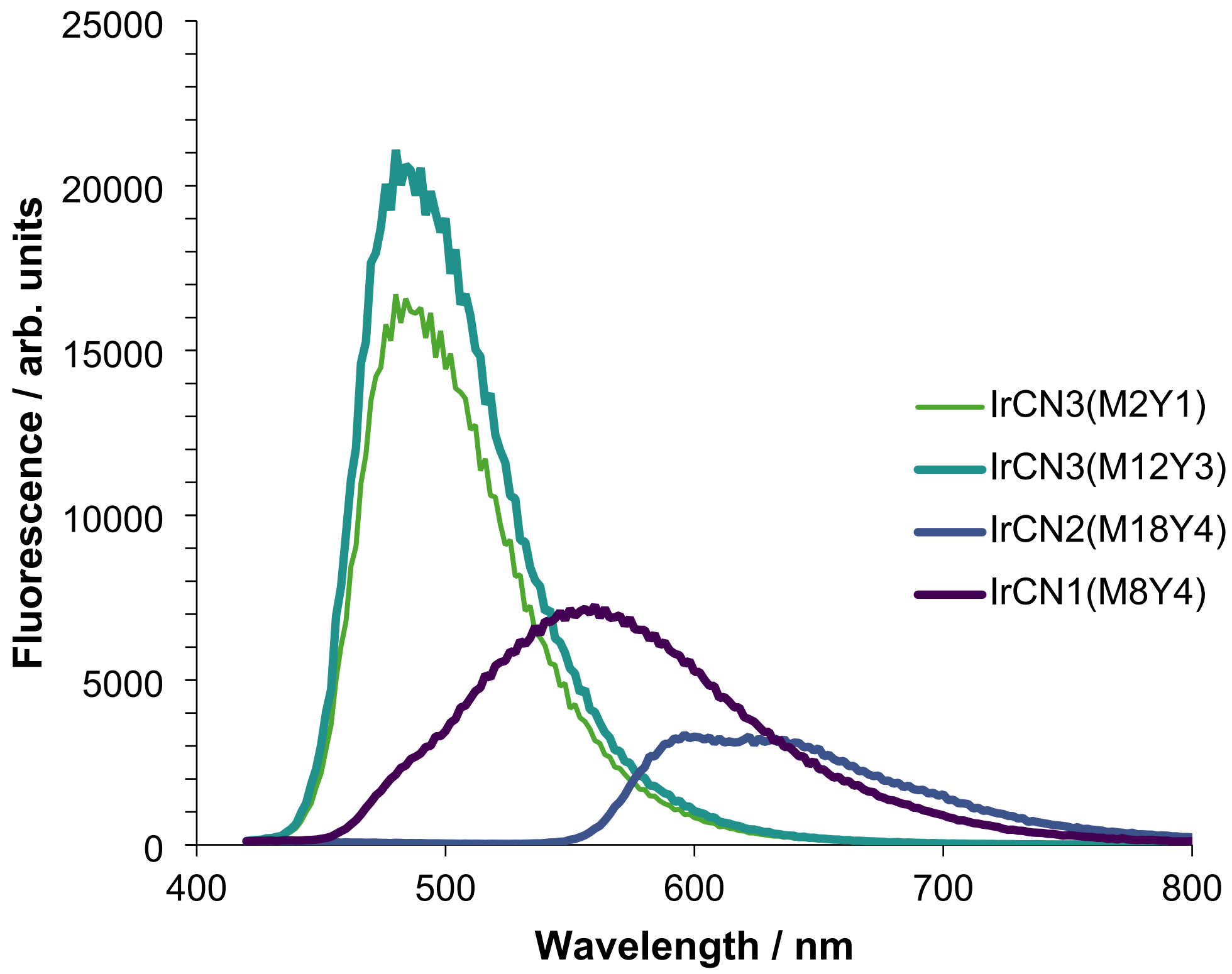

Figure S3: Fluorescence spectra (λ_{ex} = 380 nm) of pure IrCN Click compounds

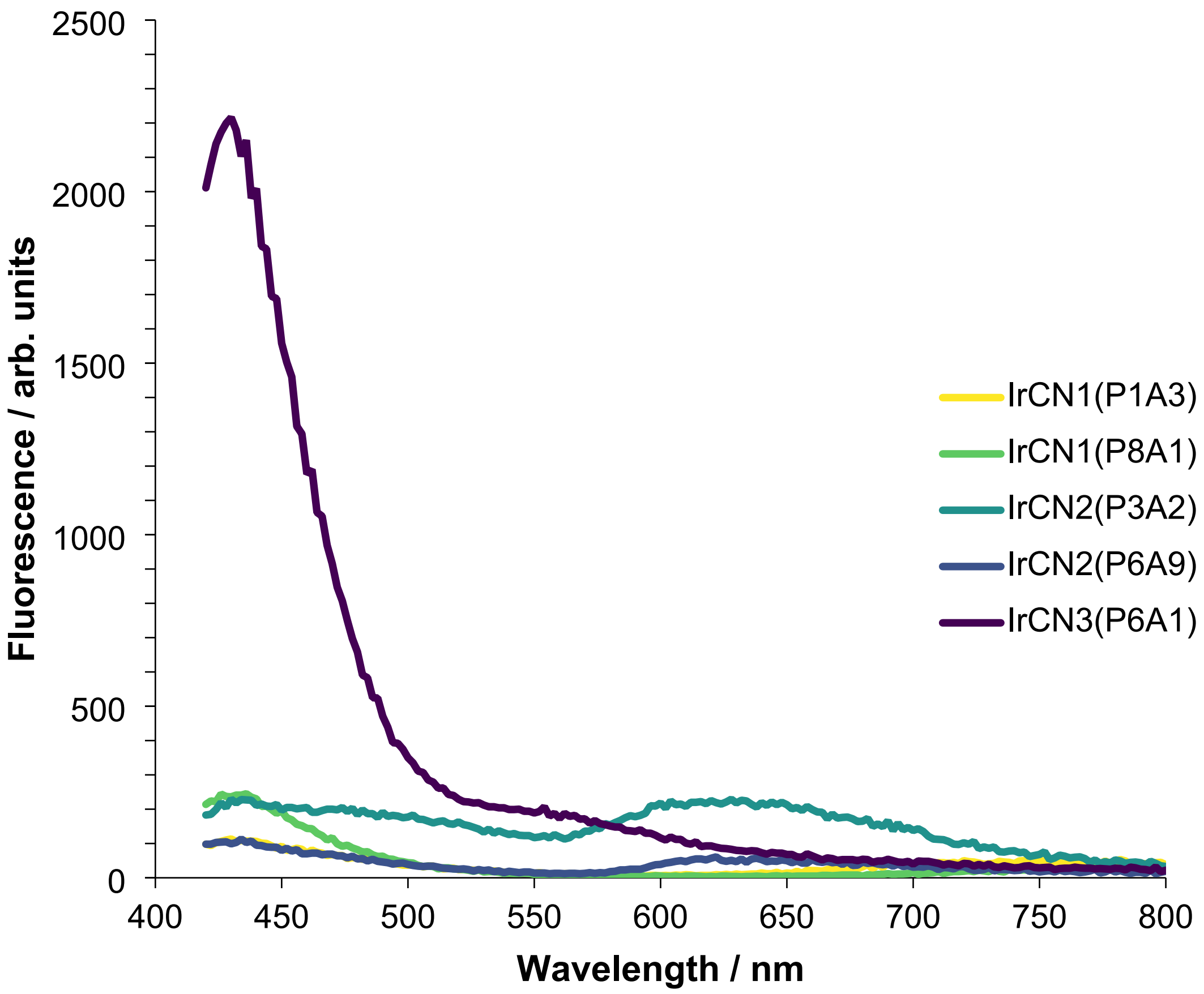

Figure S4: Fluorescence spectra (λ_{ex} = 380 nm) of pure IrCN Schiff compounds

4. Stability of pure compounds

Purified compounds were dissolved in DMSO to make solutions at 10 mM concentration. These were diluted down to 50 µM for stability tests with the following media: DMSO, DMSO:H_2O (1:1), DMSO:PBS 1X (1:1), H_2O, PBS (1X). UV-Vis spectra (280 – 600 nm, 10 flashes) were collected over 21 h to monitor the stability of these complexes at 37 °C.

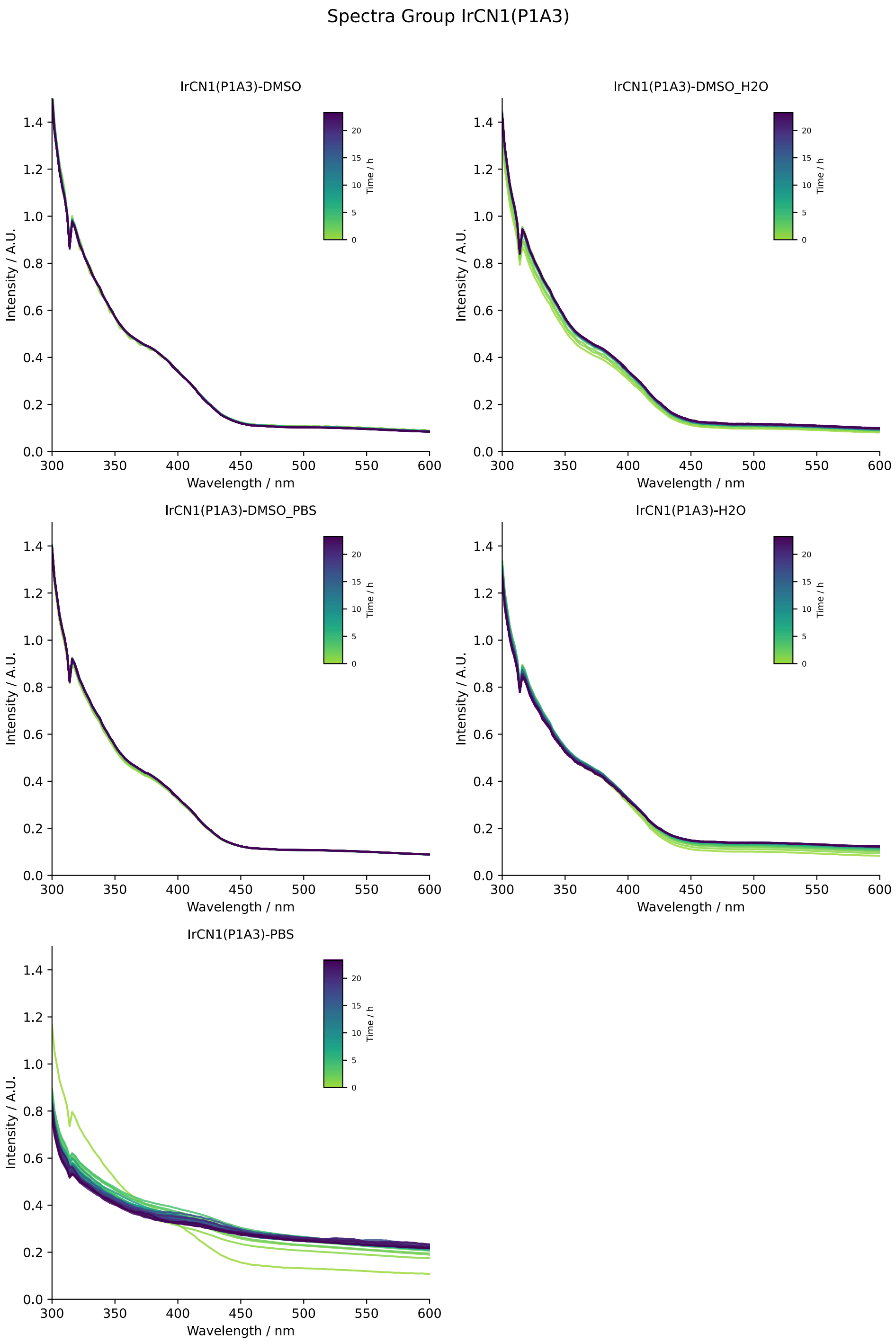

Figure S5: Absorbance spectra over 21 h at 37 °C in different solvents of IrCN1(P1A3)

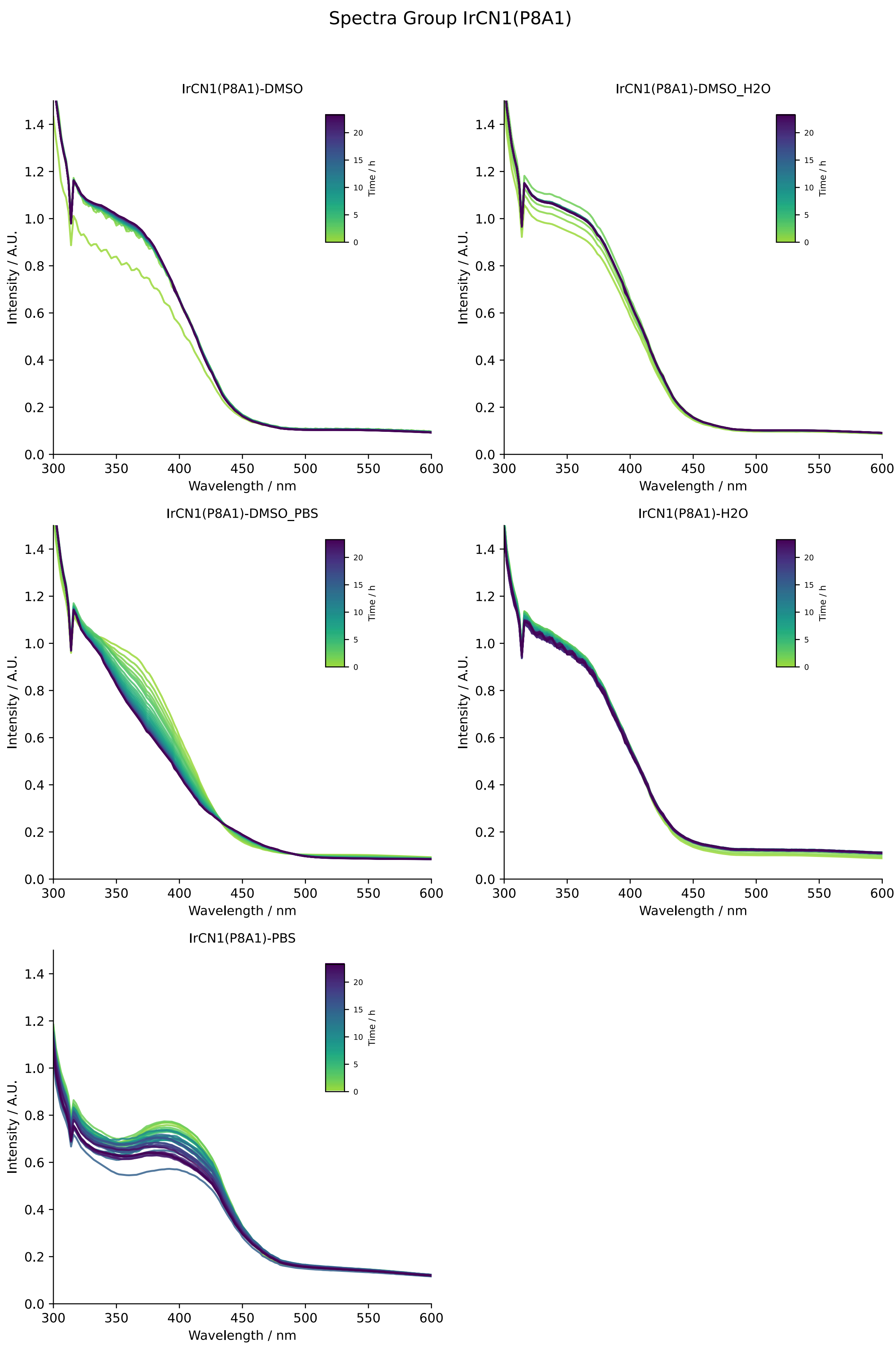

Figure S6: Absorbance spectra over 21 h at 37 °C in different solvents of IrCN1(P8A1)

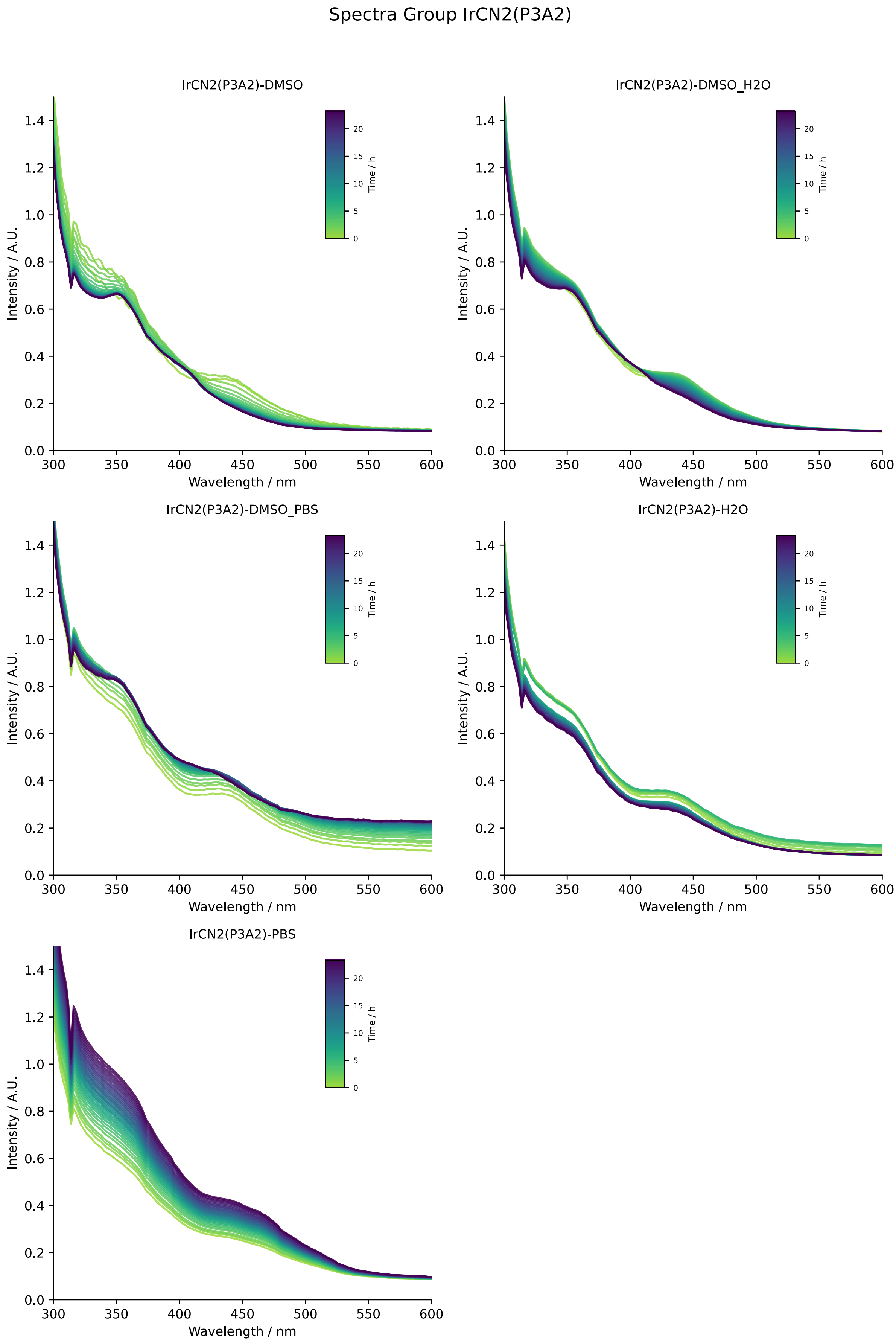

Figure S7: Absorbance spectra over 21 h at 37 °C in different solvents of IrCN2(P3A2)

Spectra Group IrCN2(P6A9)

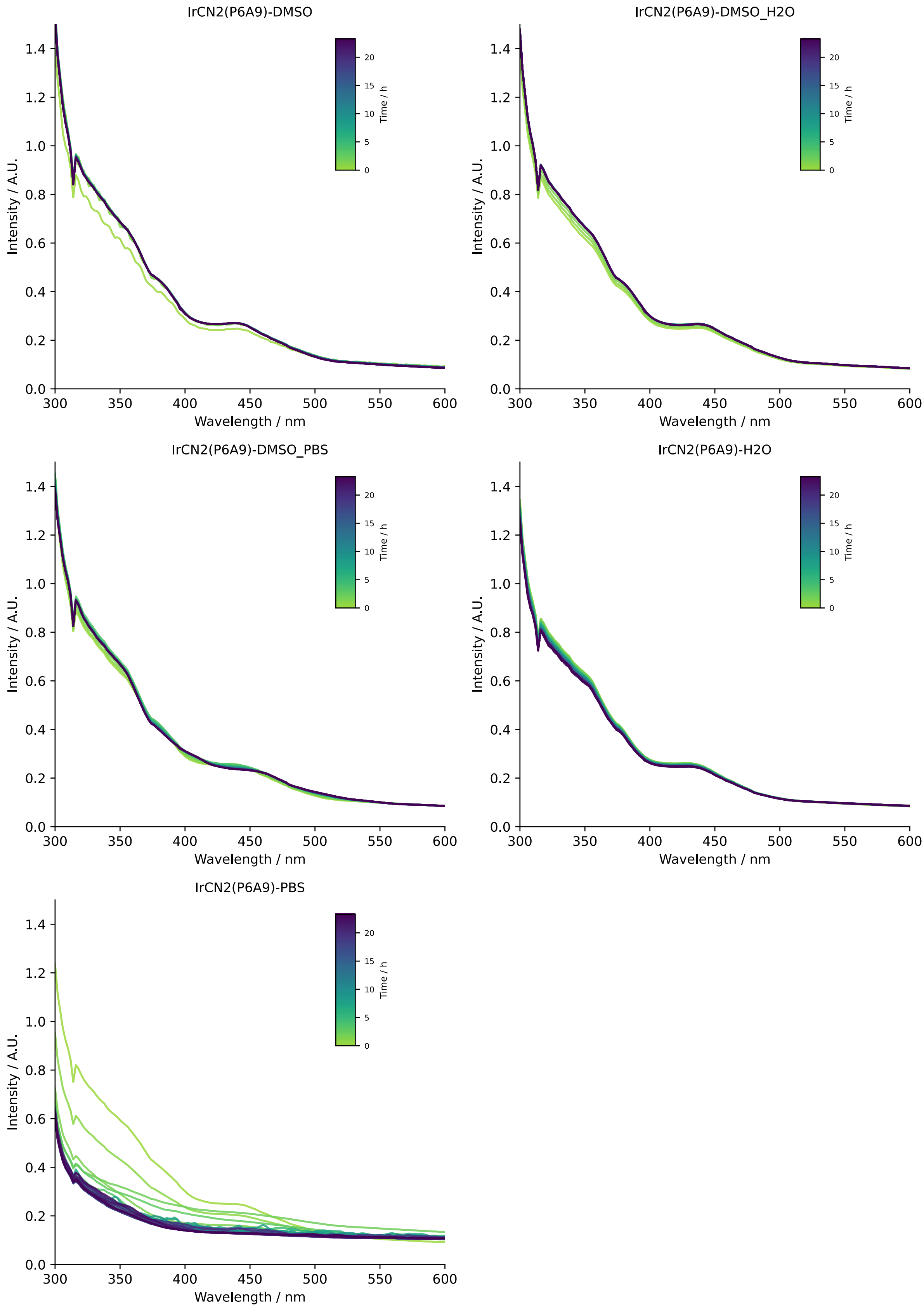

Figure S8: Absorbance spectra over 21 h at 37 °C in different solvents of IrCN2(P6A9)

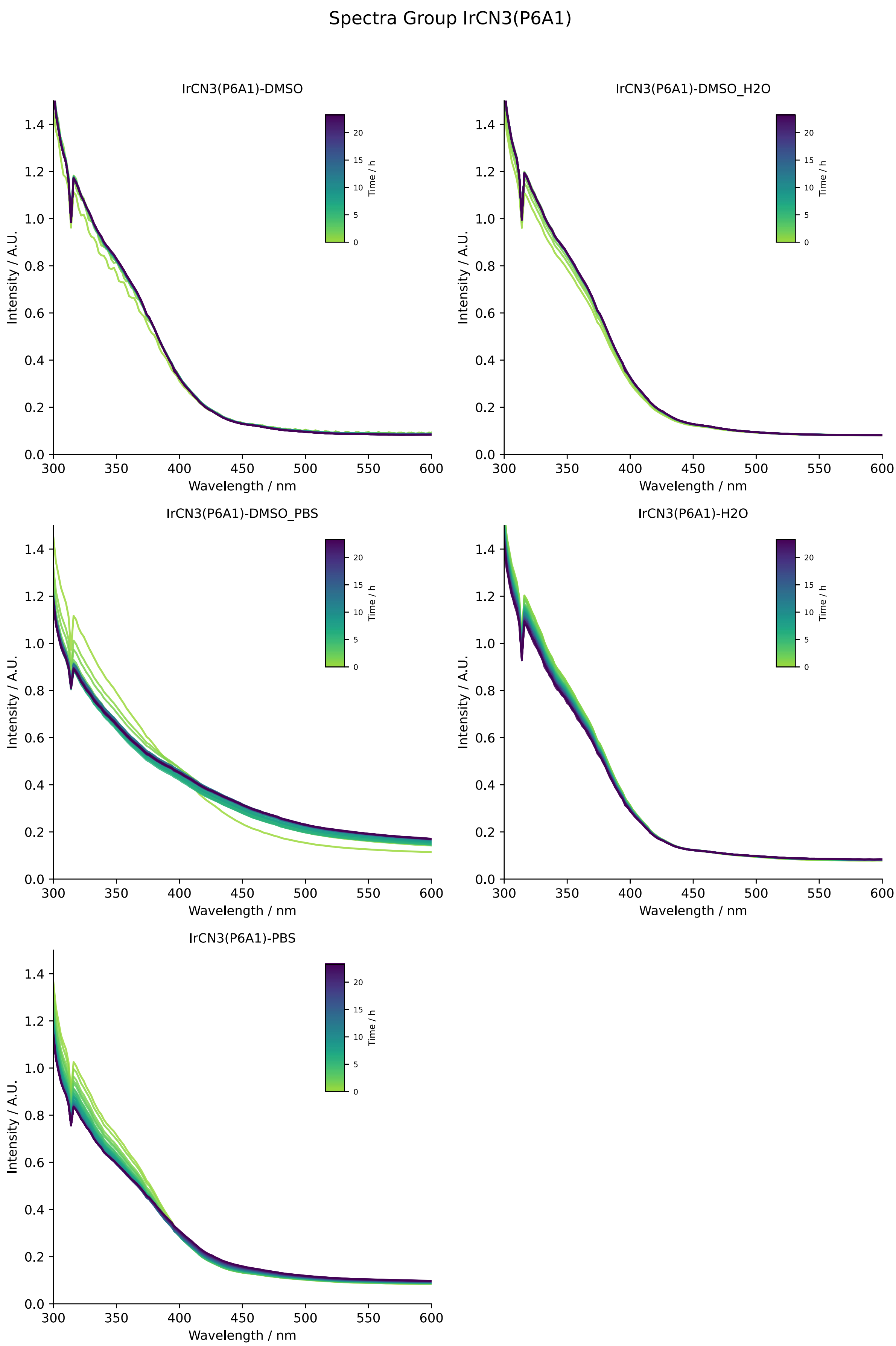

Figure S9: Absorbance spectra over 21 h at 37 °C in different solvents of IrCN3(P6A1)

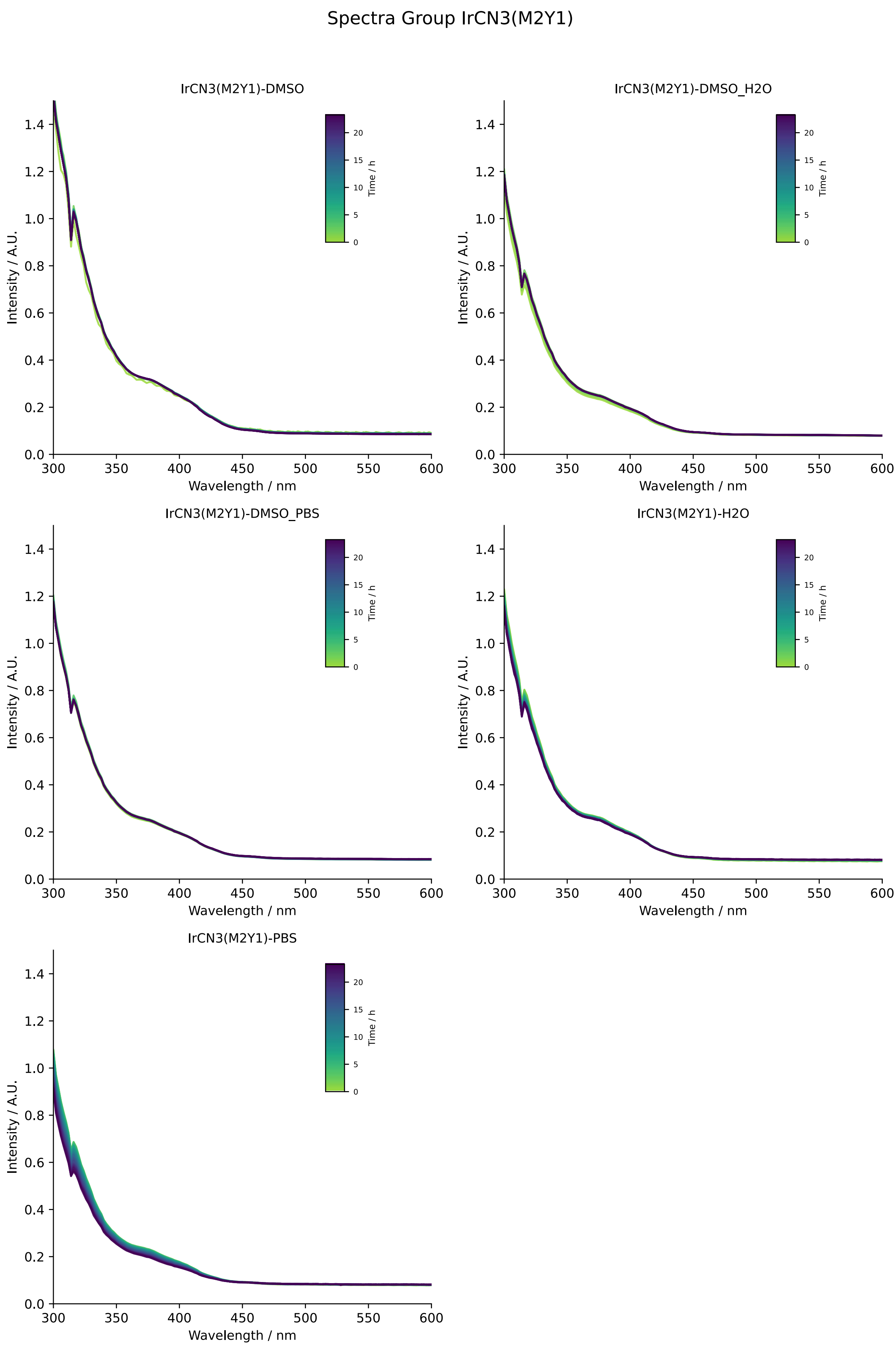

Figure S10: Absorbance spectra over 21 h at 37 °C in different solvents of IrCN3(M2Y1)

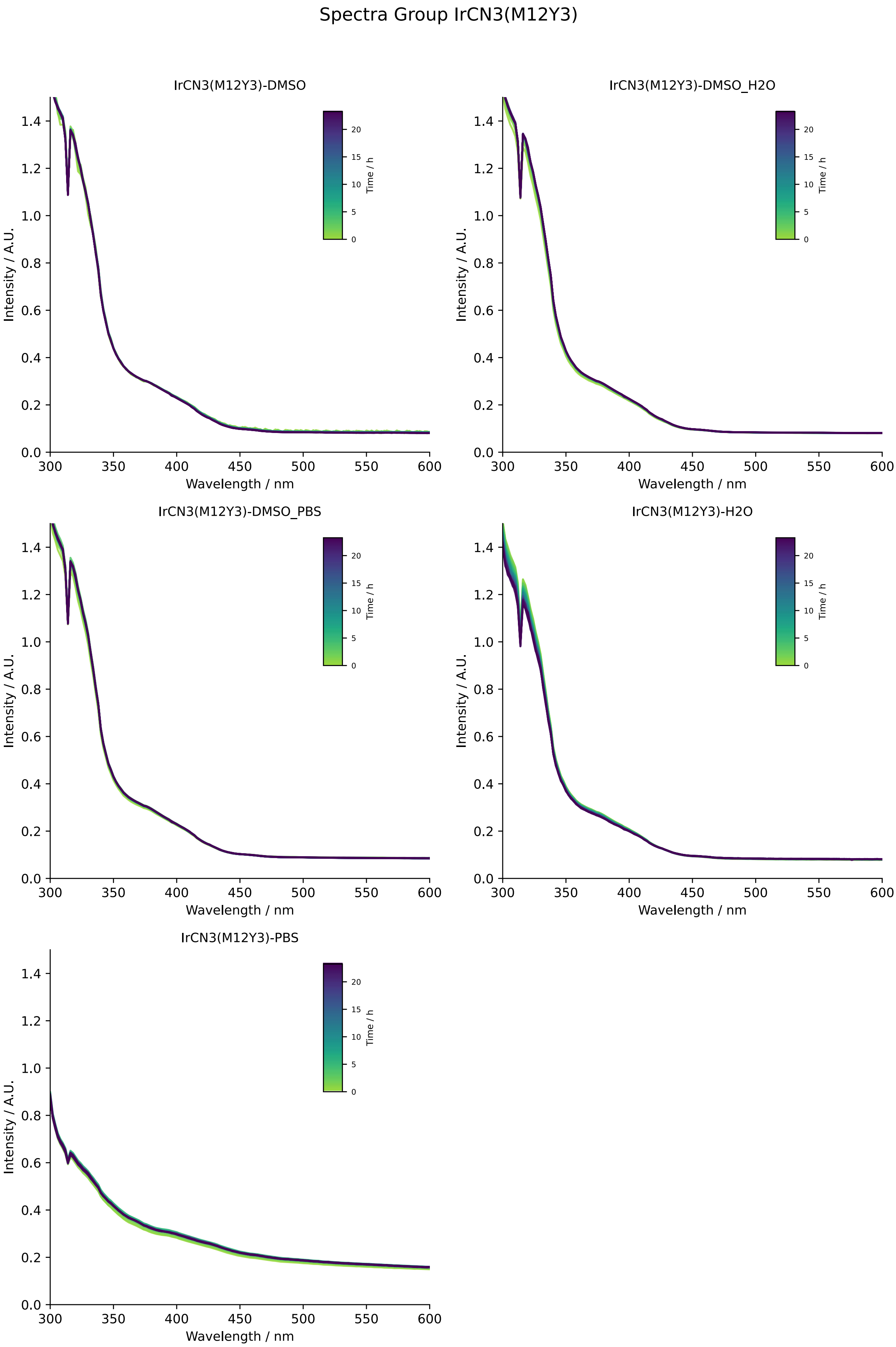

Figure S11: Absorbance spectra over 21 h at 37 °C in different solvents of IrCN3(M12Y3)

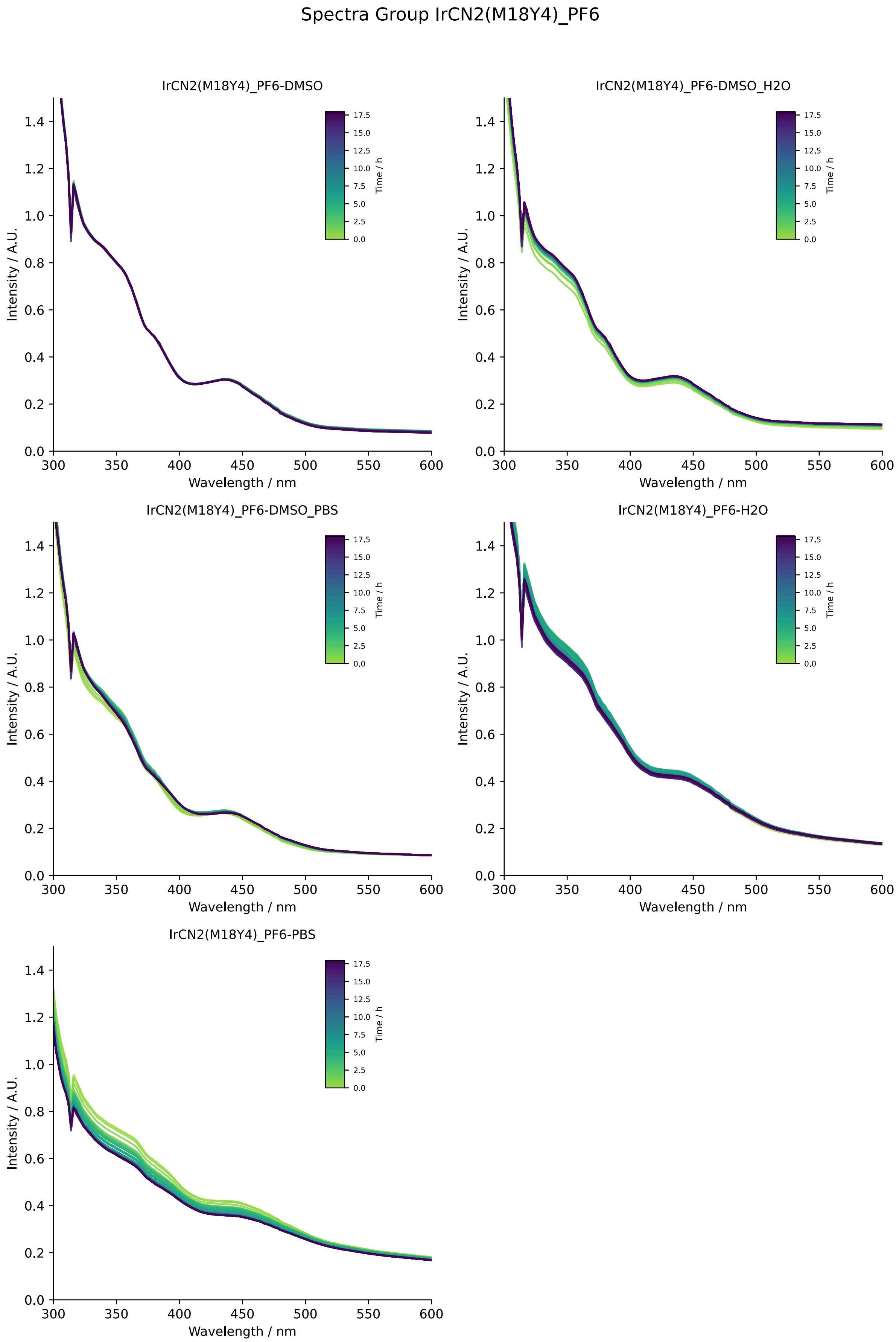

Figure S12: Absorbance spectra over 21 h at 37 °C in different solvents of IrCN2(M18Y4)-PF_6

Corresponding stability studies were also undertaken using LC-MS. Complexes were dissolved in DMSO (5 mM) then added to either DMSO (24 µL in 1200 µL) or 1:1 DMSO: PBS (1X) (24 µL in 1200 µL) in 2 mL HPLC vials fitted with a PTFE septum, giving a concentration of 100 µM. The samples were incubated at 37 °C inside the LC-MS sample chamber and analyzed by LC-MS over the course of 18 hours. The broad absorbances at the start of each trace are due to DMSO and PBS.

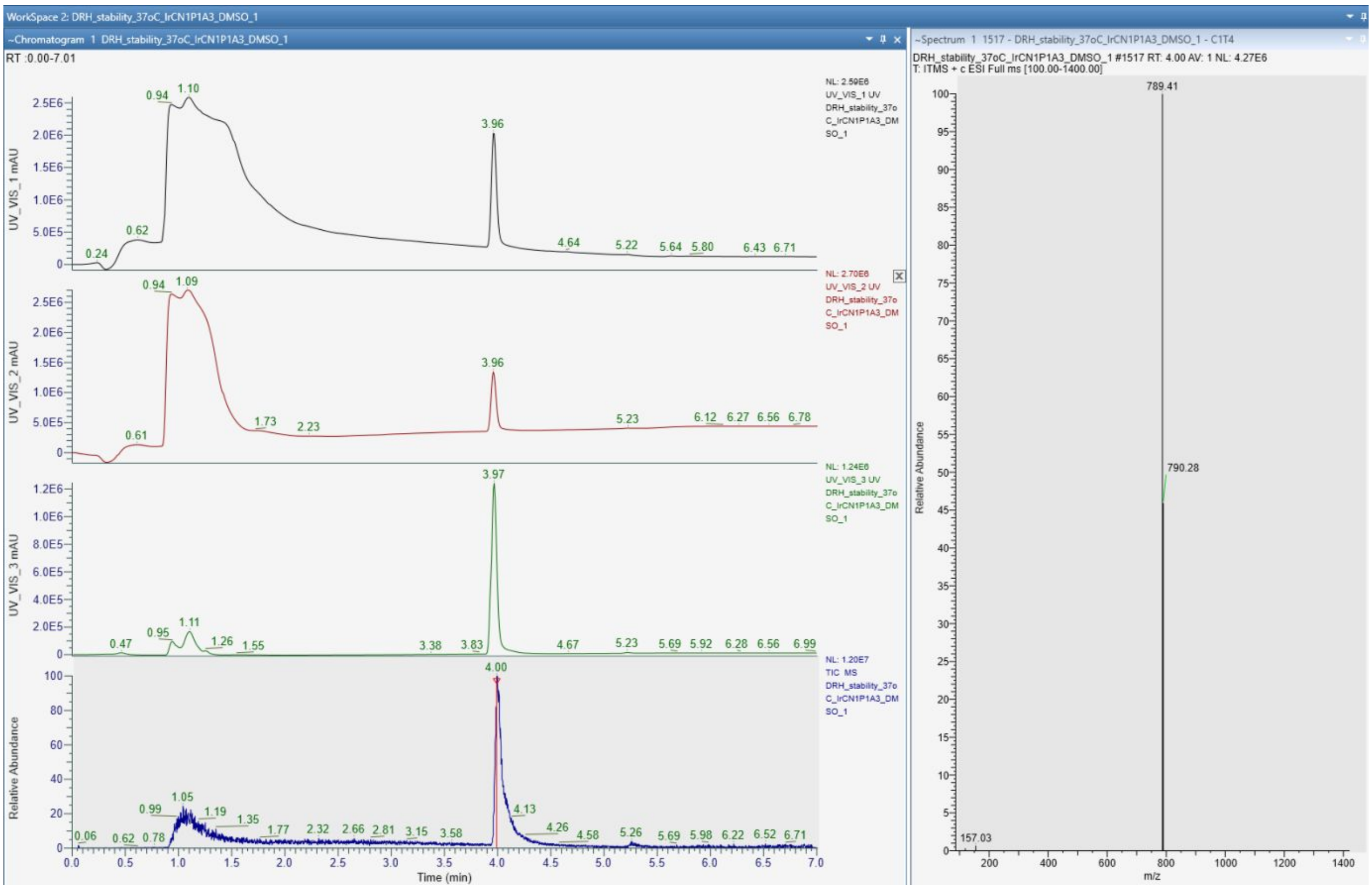

Figure S13: LC-MS trace (7 min method) of IrCN1(P1A3) in DMSO (100 µM) after 0 h at 37 °C

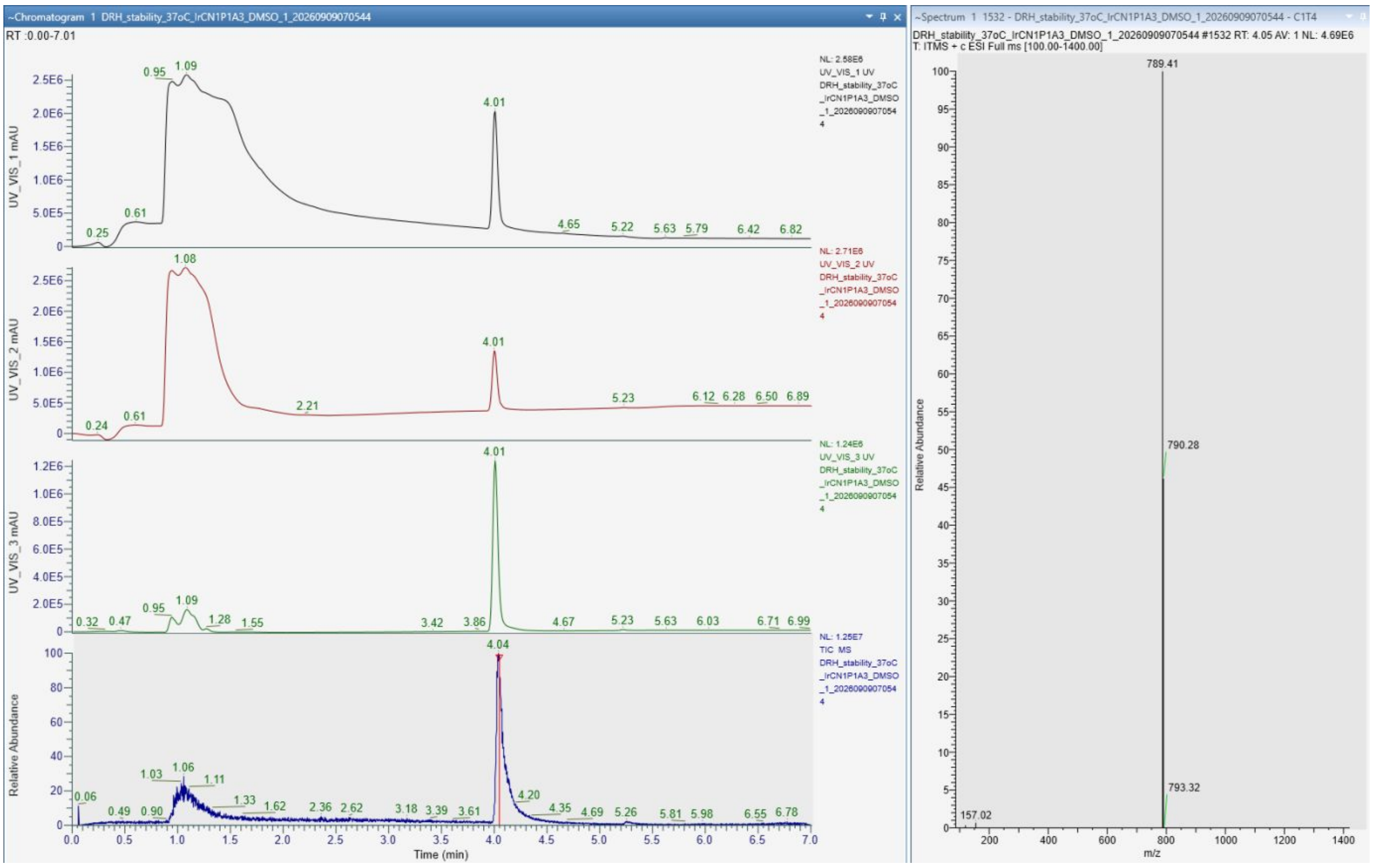

Figure S14: LC-MS trace (7 min method) of IrCN1(P1A3) in DMSO (100 µM) after 18 h at 37 °C

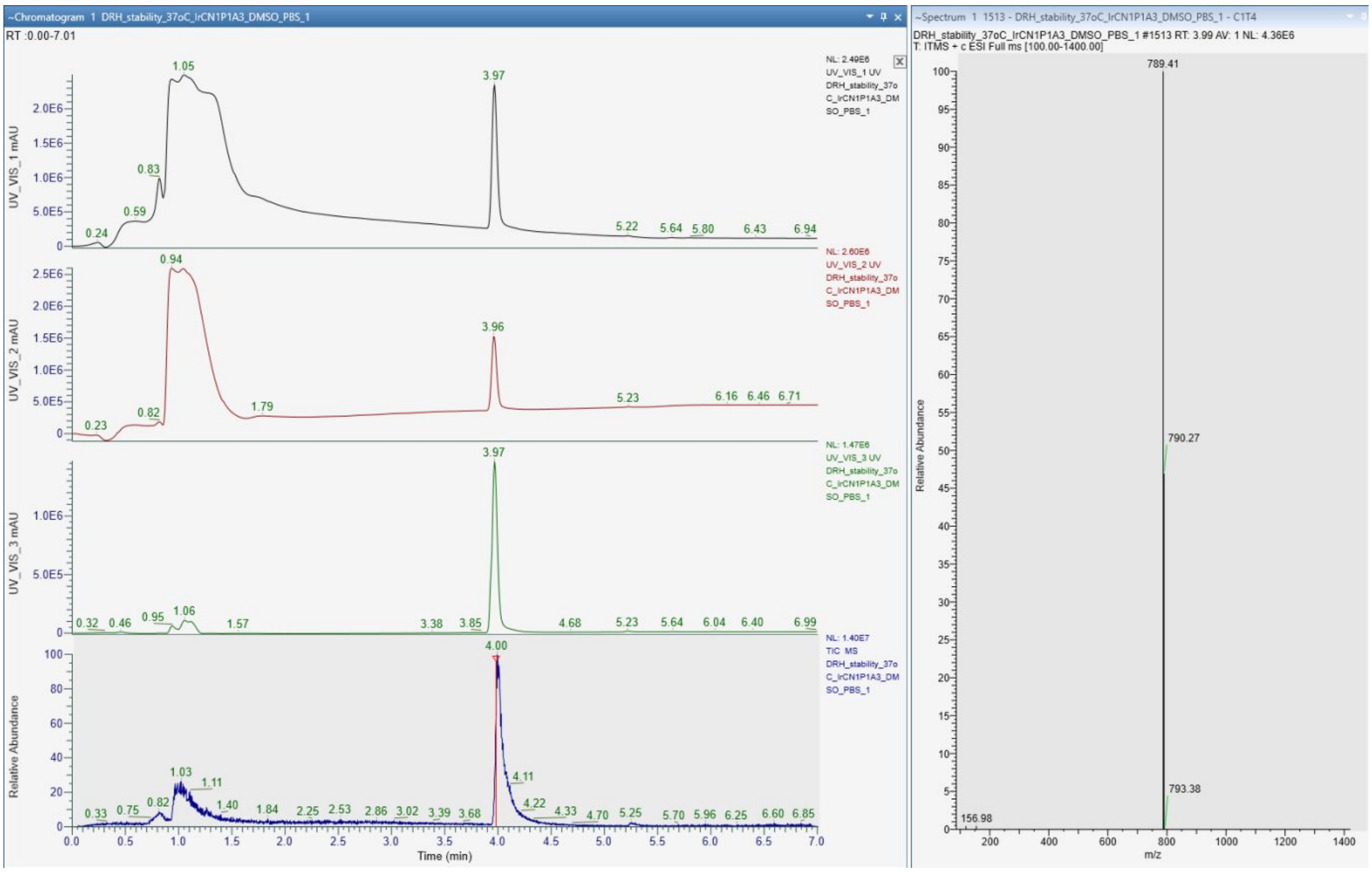

Figure S15: LC-MS trace (7 min method) of IrCN1(P1A3) in DMSO: PBS (1X) (100 µM) after 0 h at 37 °C

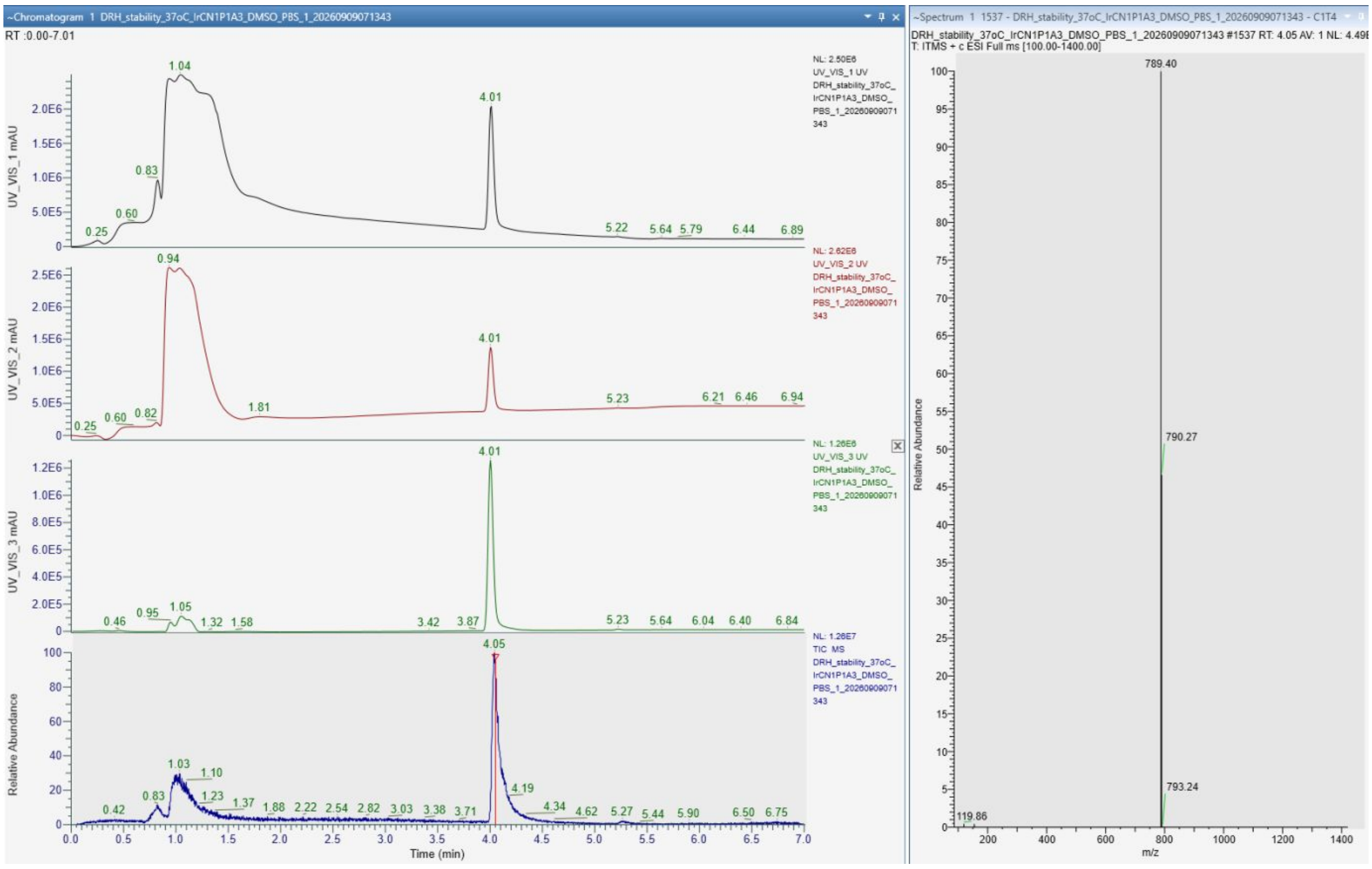

Figure S16: LC-MS trace (7 min method) of IrCN1(P1A3) in DMSO: PBS (1X) (100 μM) after 18 h at 37 °C

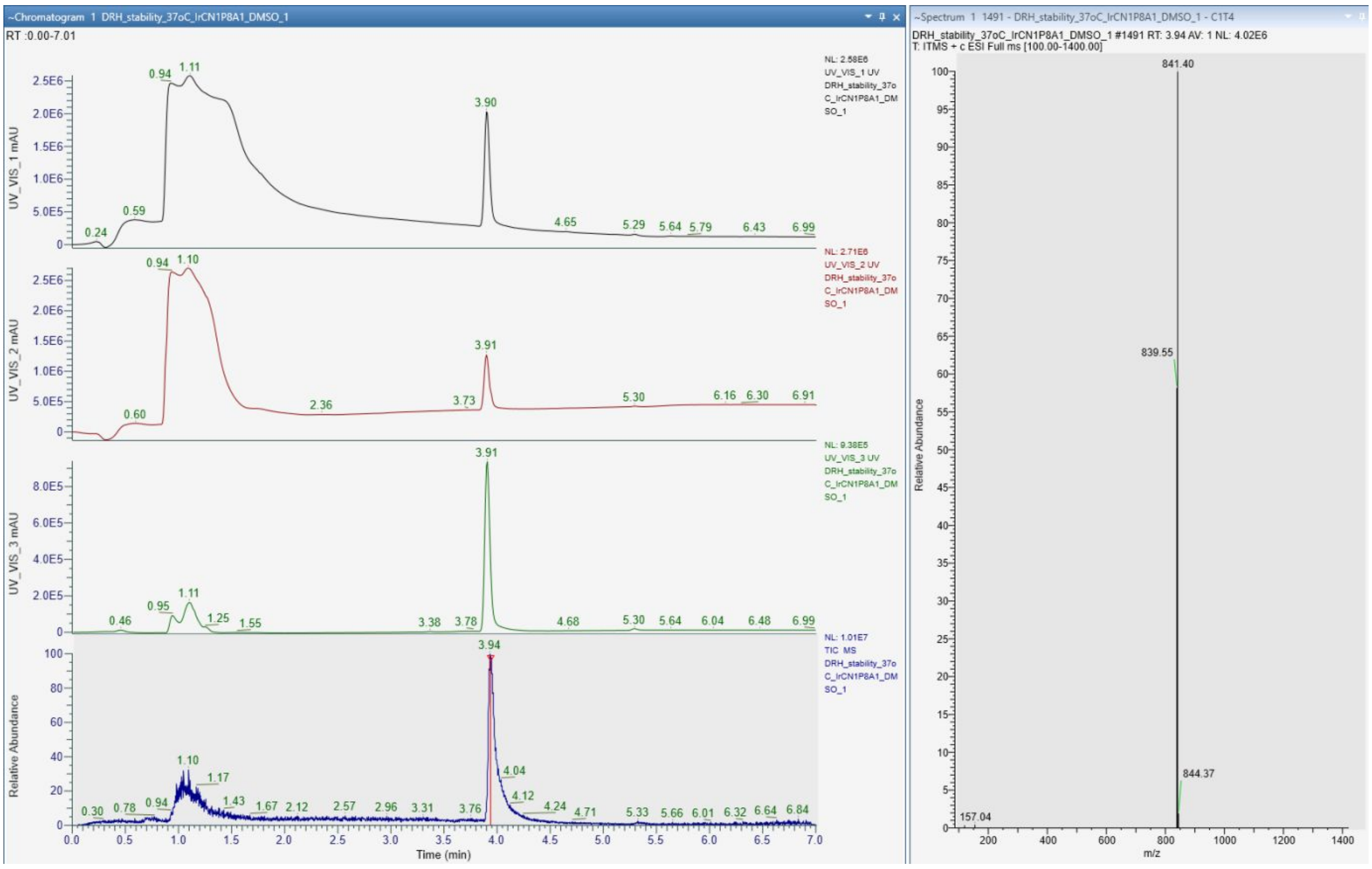

Figure S17: LC-MS trace (7 min method) of IrCN1(P8A1) in DMSO (100 μM) after 0 h at 37 °C

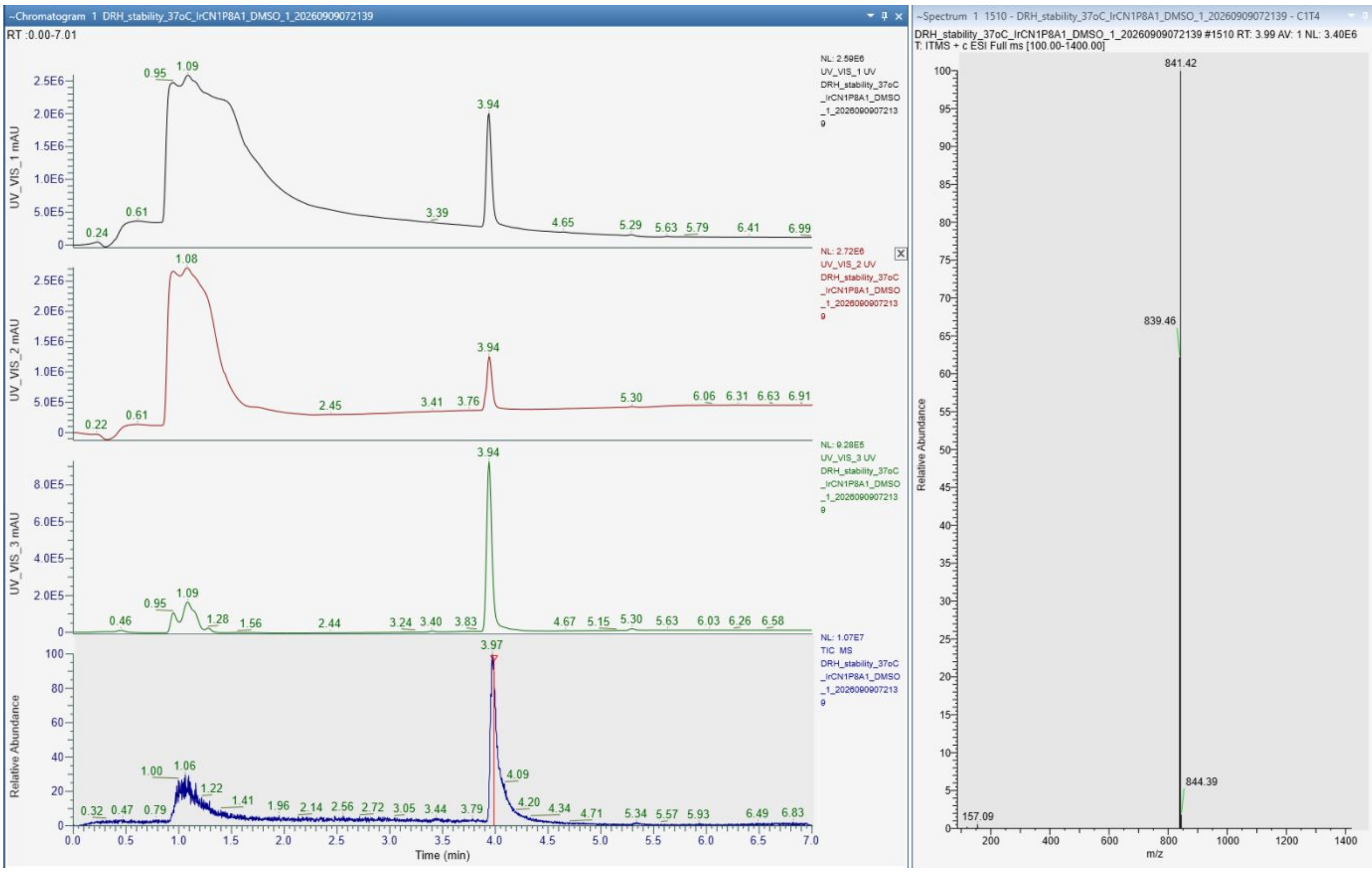

Figure S18: LC-MS trace (7 min method) of IrCN1(P8A1) in DMSO (100 μM) after 18 h at 37 °C

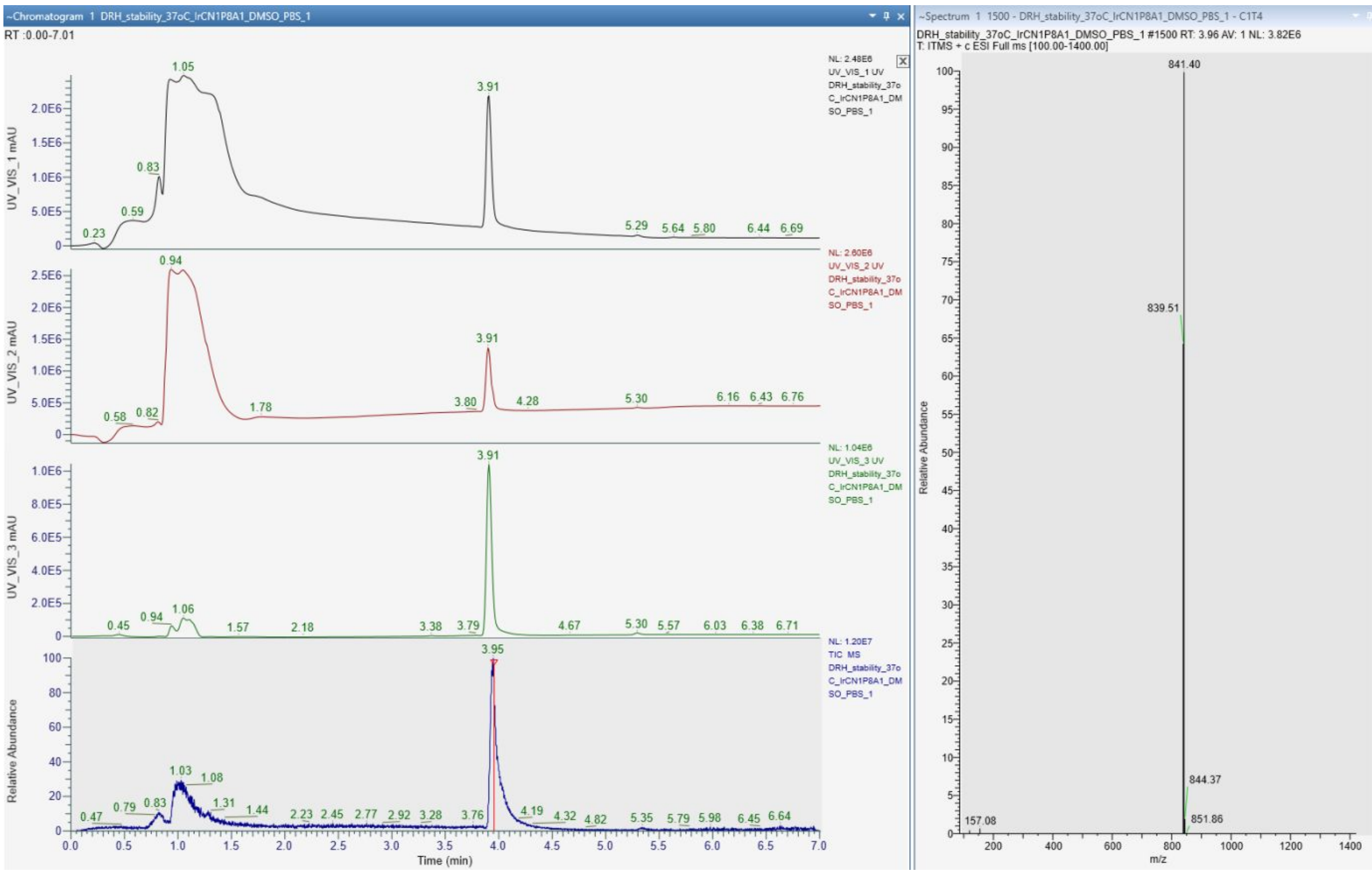

Figure S19: LC-MS trace (7 min method) of IrCN1(P8A1) in DMSO: PBS (1X) (100 μM) after 0 h at 37 °C

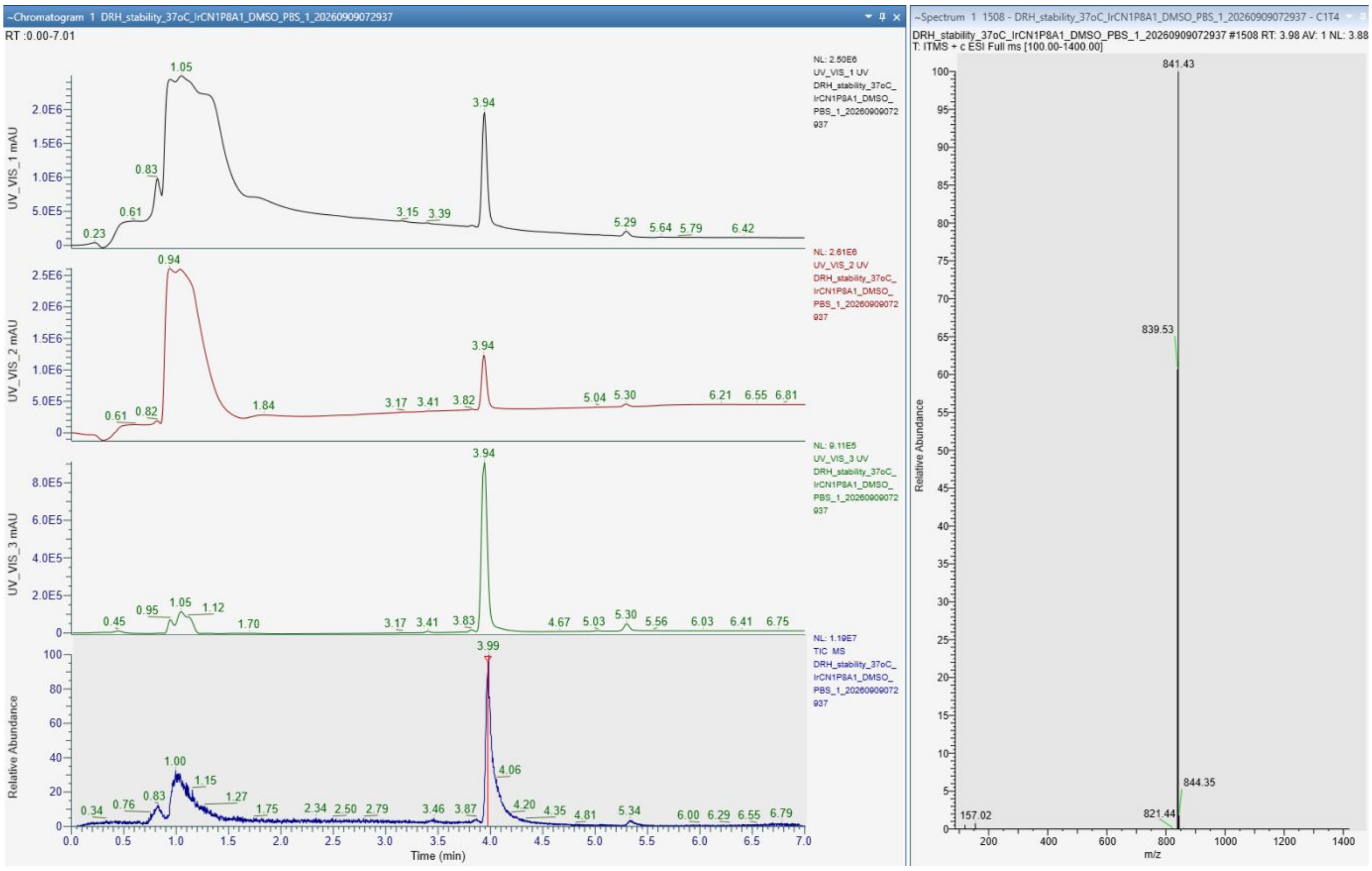

Figure S20: LC-MS trace (7 min method) of IrCN1(P8A1) in DMSO: PBS (1X) (100 µM) after 18 h at 37 °C

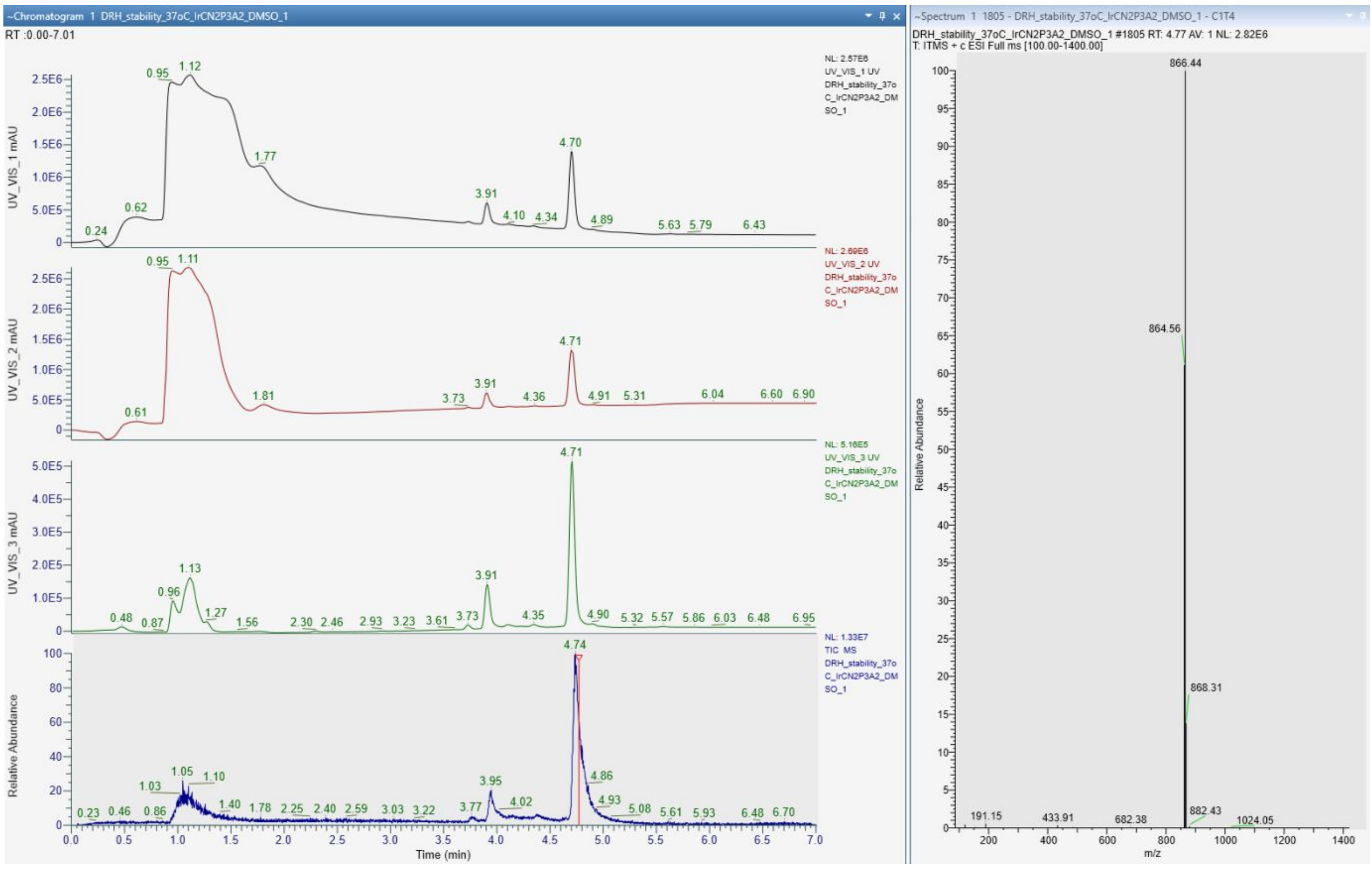

Figure S21: LC-MS trace (7 min method) of IrCN2(P3A2) in DMSO (100 µM) after 0 h at 37 °C. The peak at 3.91 min is the IrCN2(MeCN)$_2$ species.

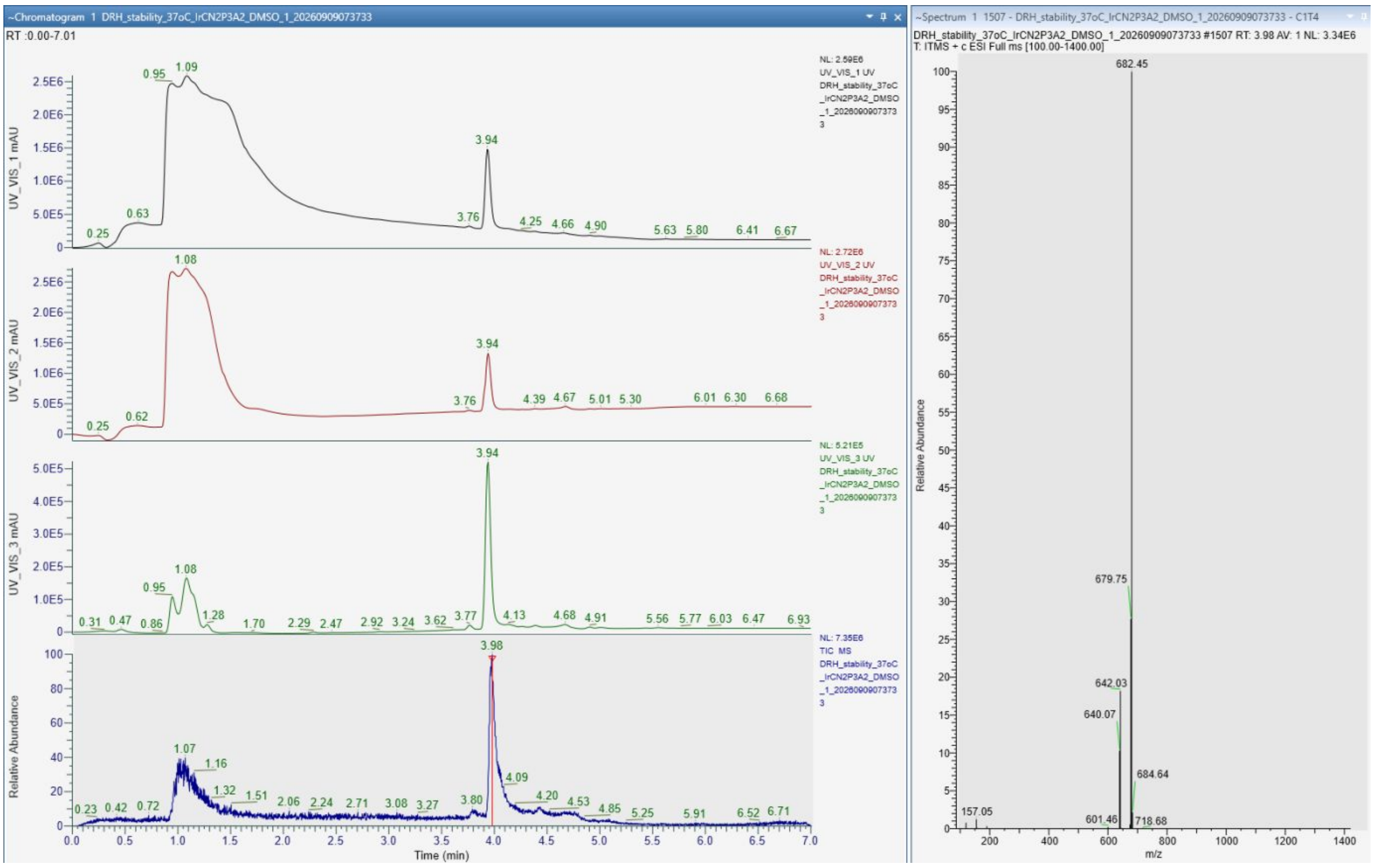

Figure S22: LC-MS trace (7 min method) of IrCN2(P3A2) in DMSO (100 µM) after 18 h at 37 °C. The peak at 3.94 min corresponds to an adduct of the IrCN2 dimer (confirmed by an authentic sample of IrCN2)

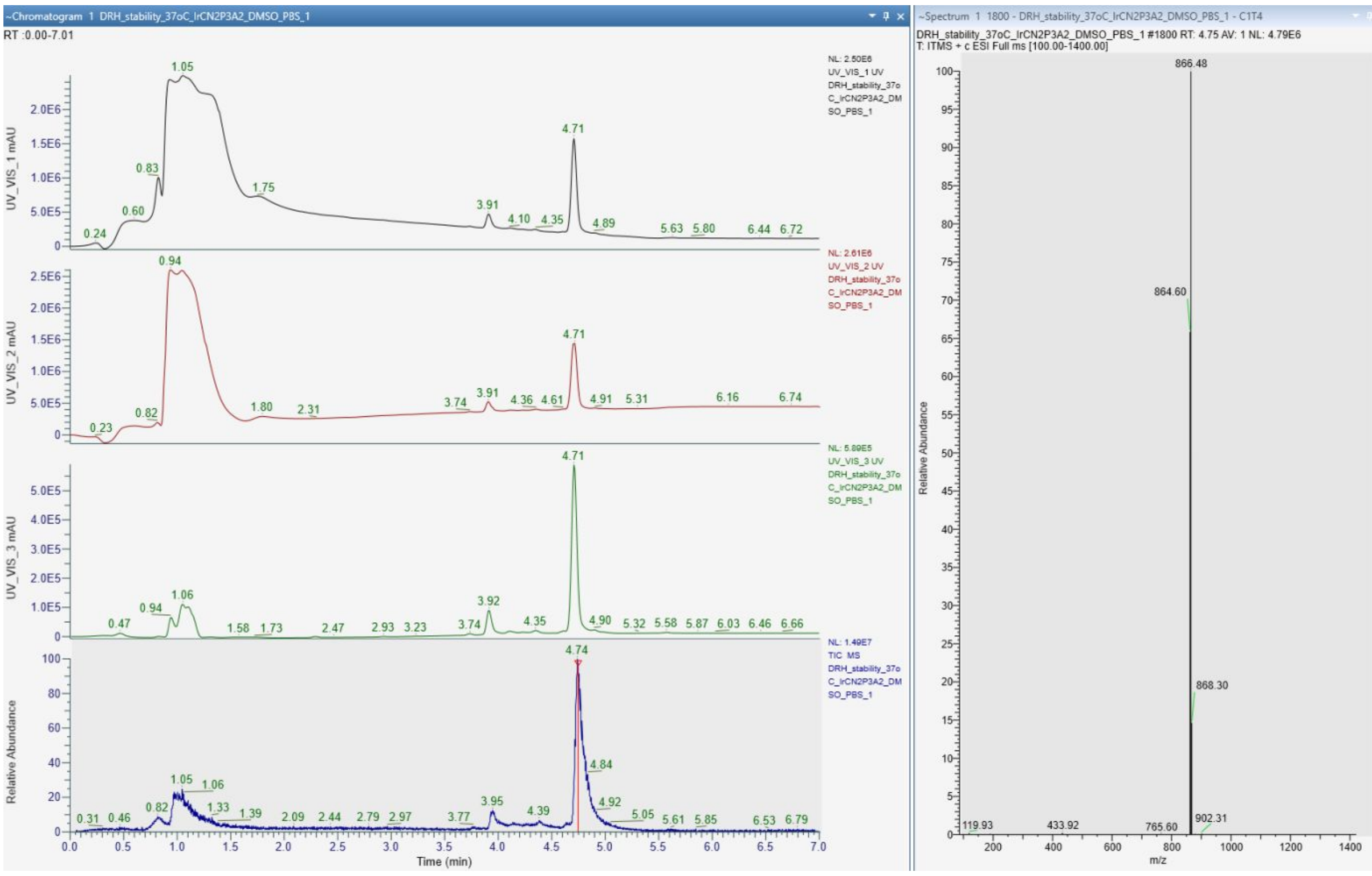

Figure S23: LC-MS trace (7 min method) of IrCN2(P3A2) in DMSO:PBS (1X) (100 µM) after 0 h at 37 °C

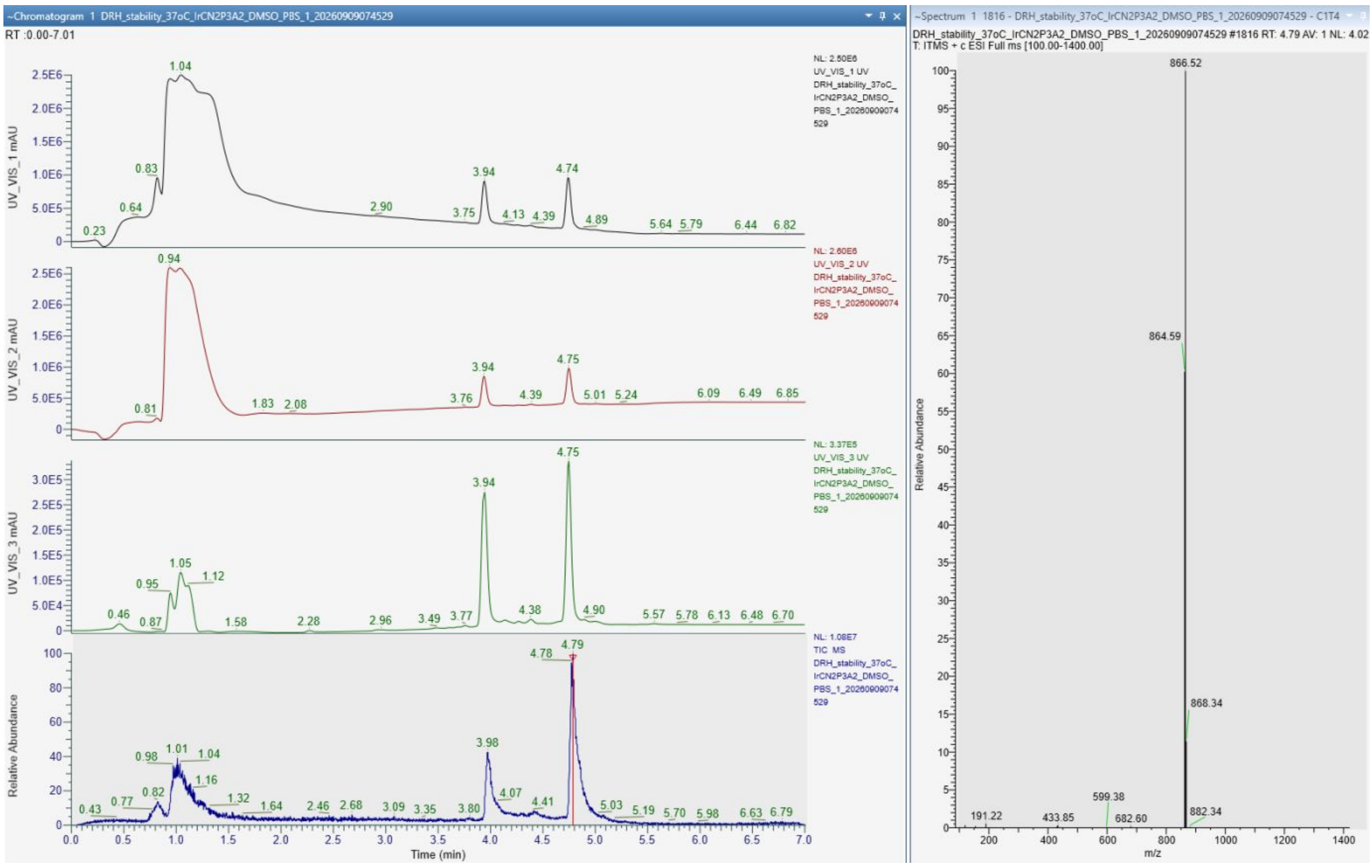

Figure S24: LC-MS trace (7 min method) of IrCN2(P3A2) in DMSO:PBS (1X) (100 µM) after 18 h at 37 °C

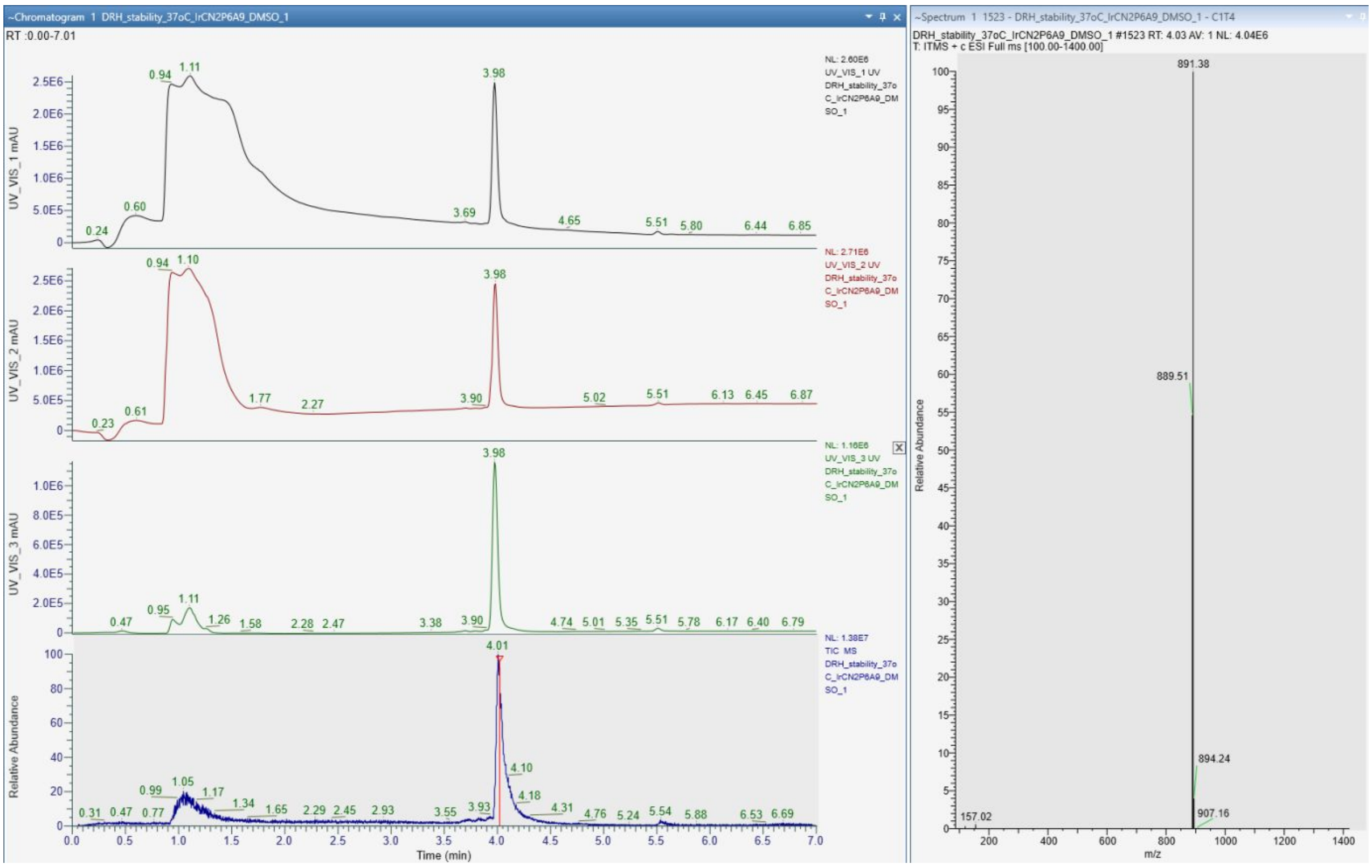

Figure S25: LC-MS trace (7 min method) of IrCN2(P6A9) in DMSO (100 µM) after 0 h at 37 °C

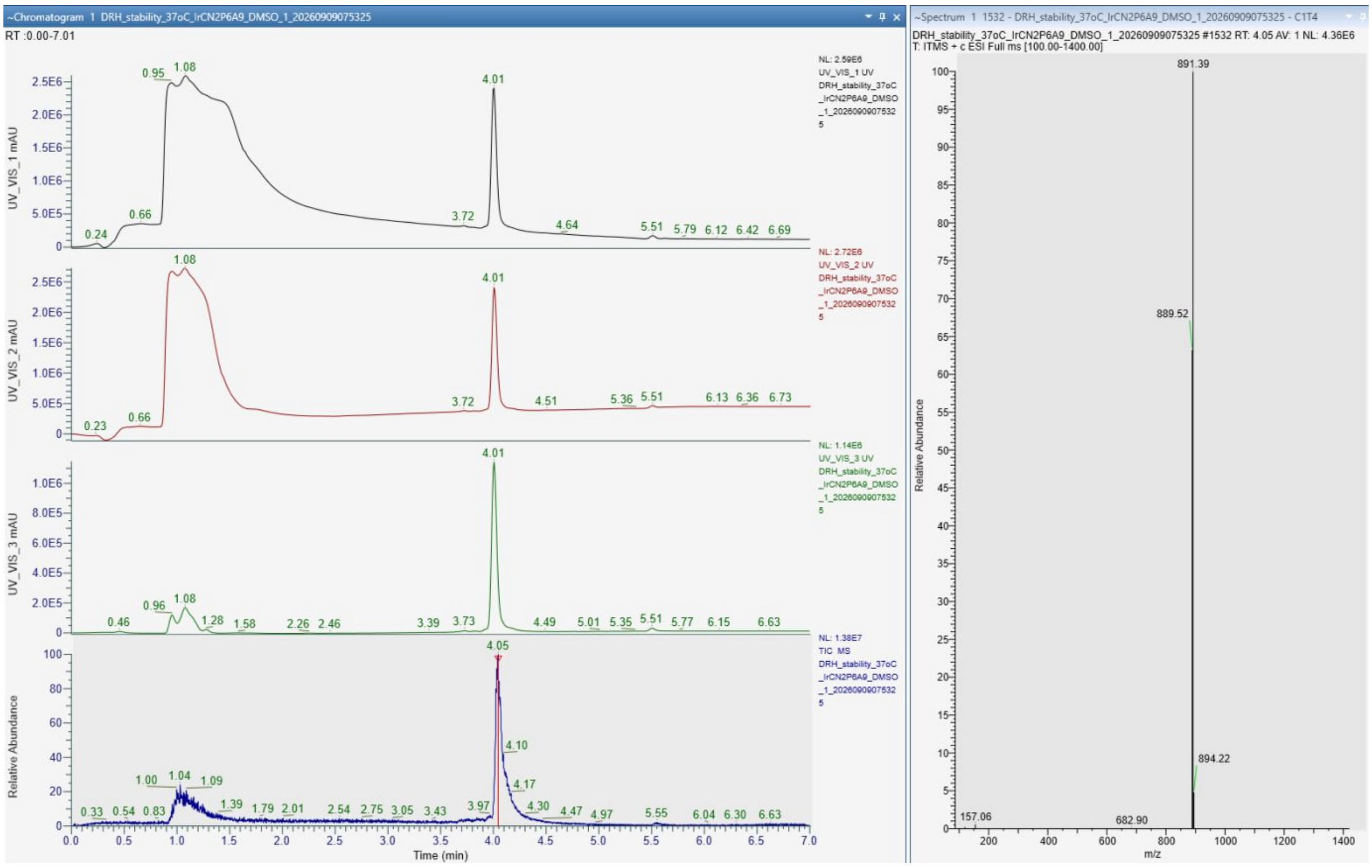

Figure S26: LC-MS trace (7 min method) of IrCN2(P6A9) in DMSO (100 μM) after 18 h at 37 °C

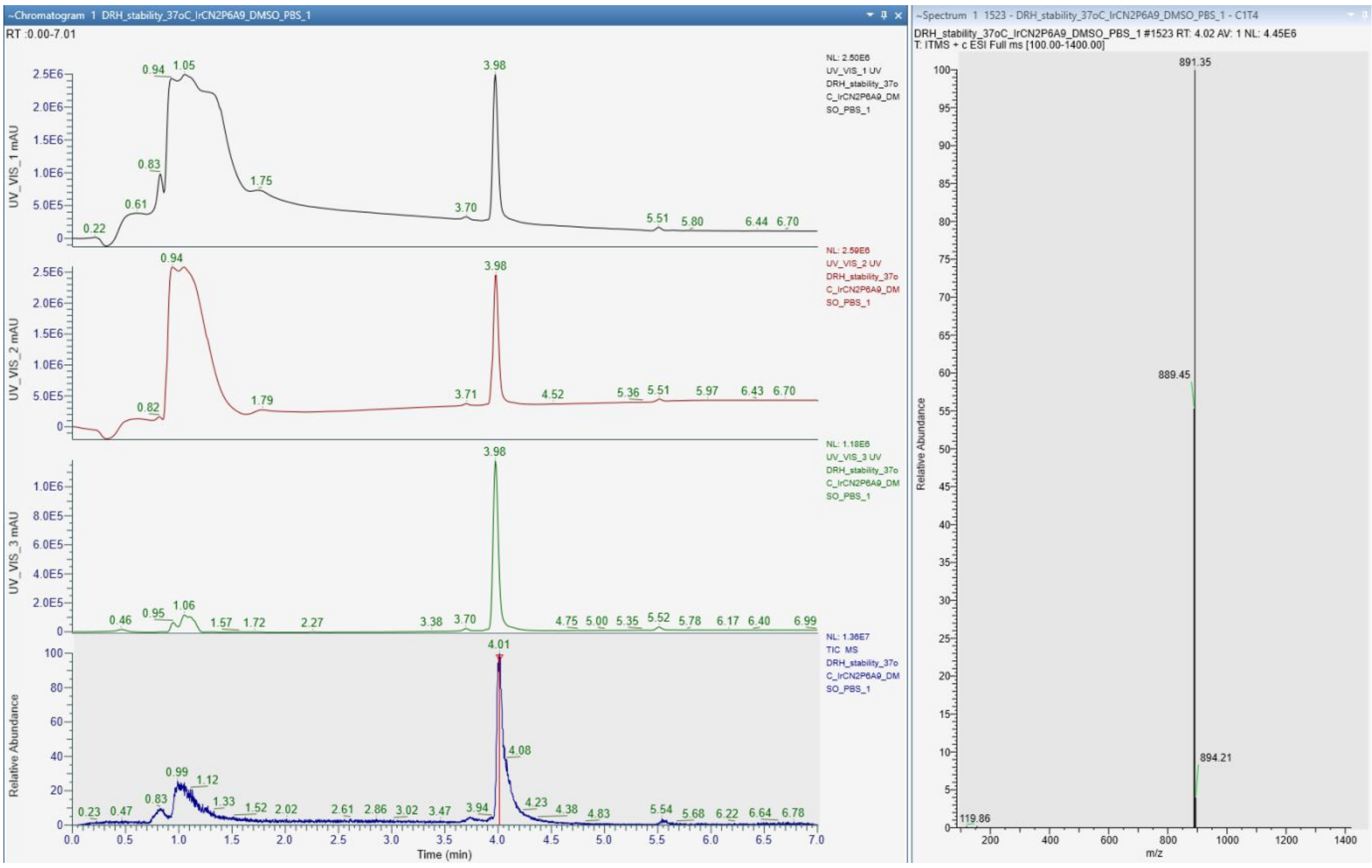

Figure S27: LC-MS trace (7 min method) of IrCN2(P6A9) in DMSO: PBS (1X) (100 μM) after 0 h at 37 °C

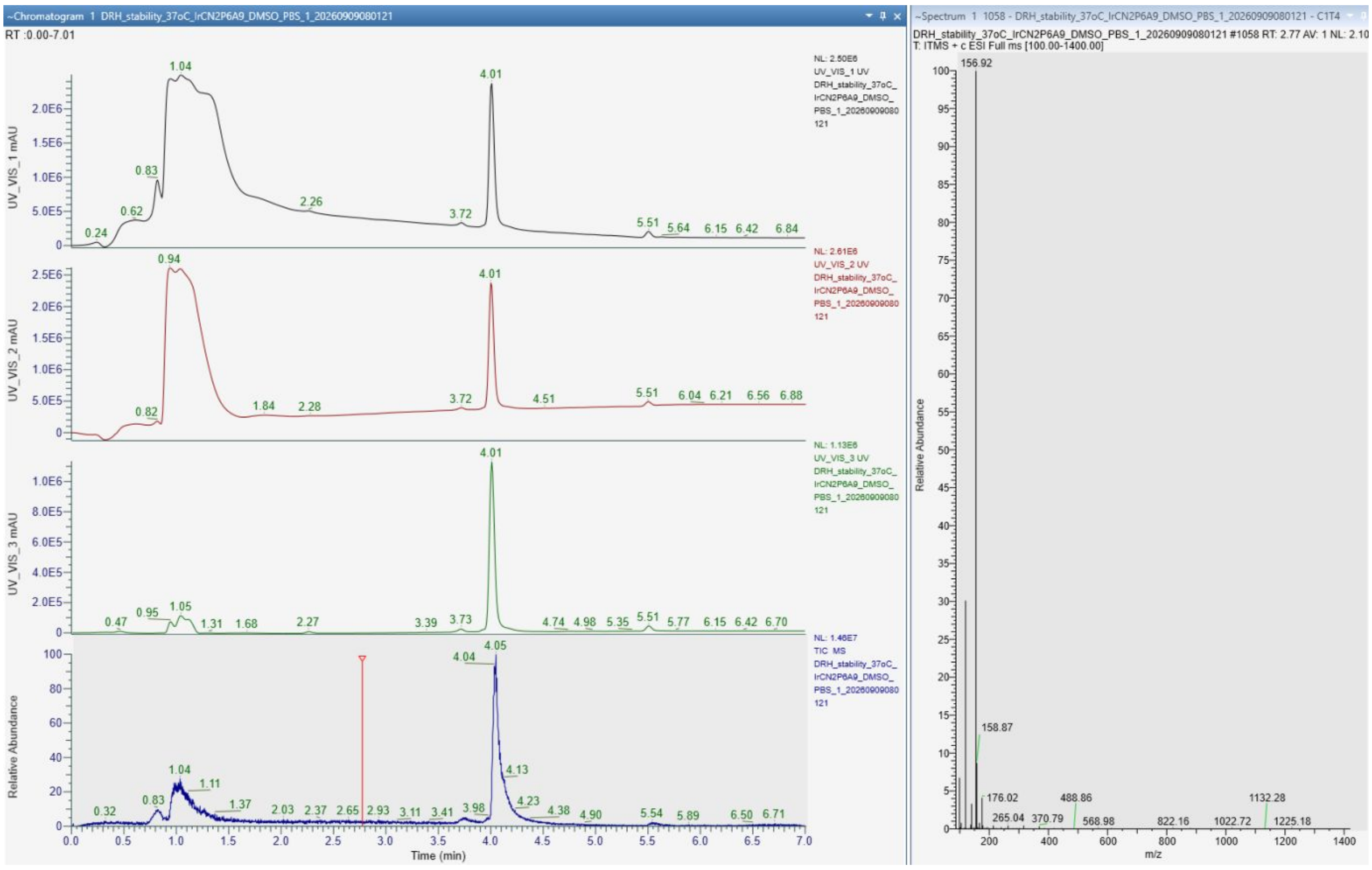

Figure S28: LC-MS trace (7 min method) of IrCN2(P6A9) in DMSO:PBS (1X) (100 µM) after 18 h at 37 °C

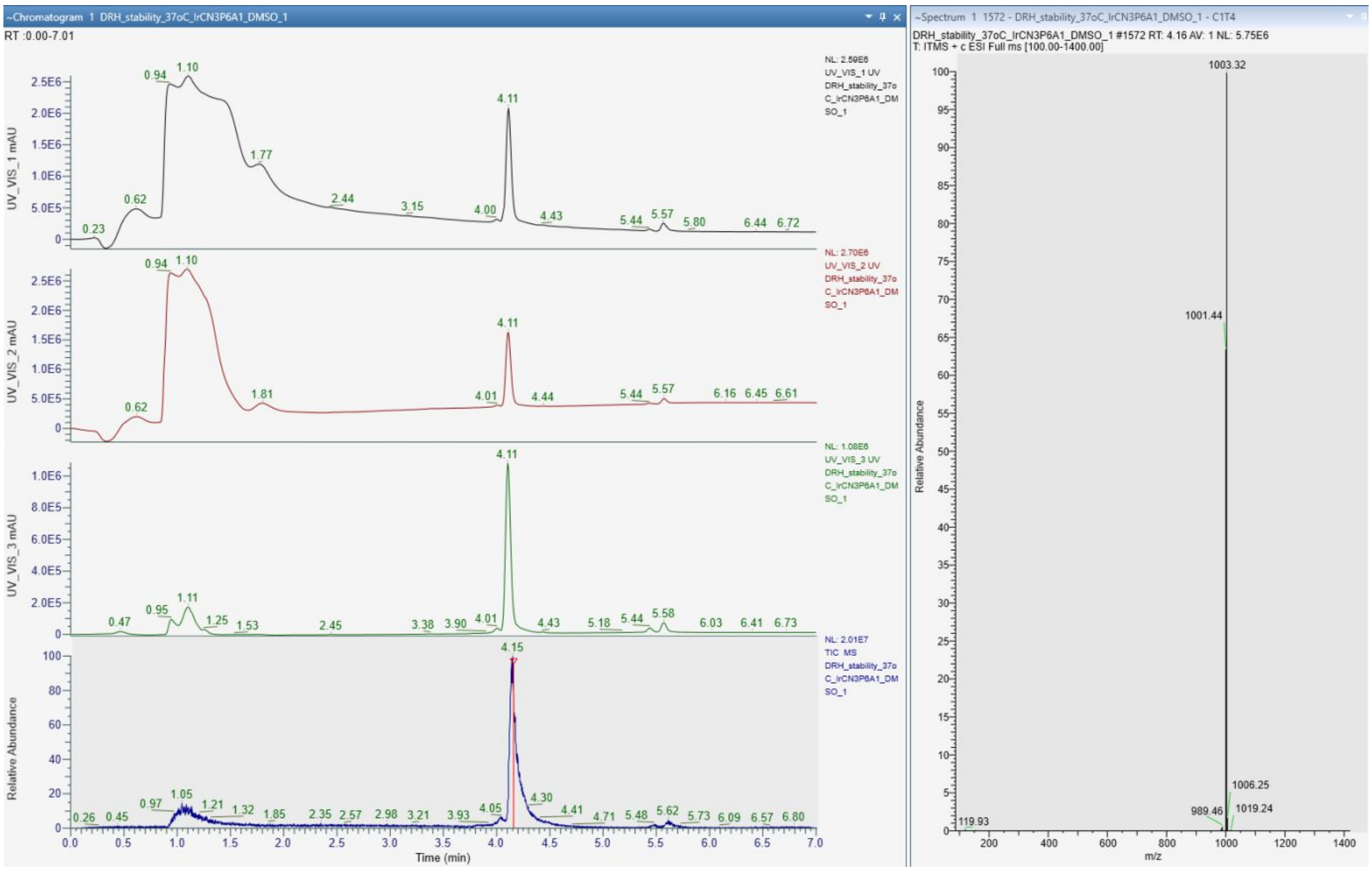

Figure S29: LC-MS trace (7 min method) of IrCN3(P6A1) in DMSO (100 µM) after 0 h at 37 °C

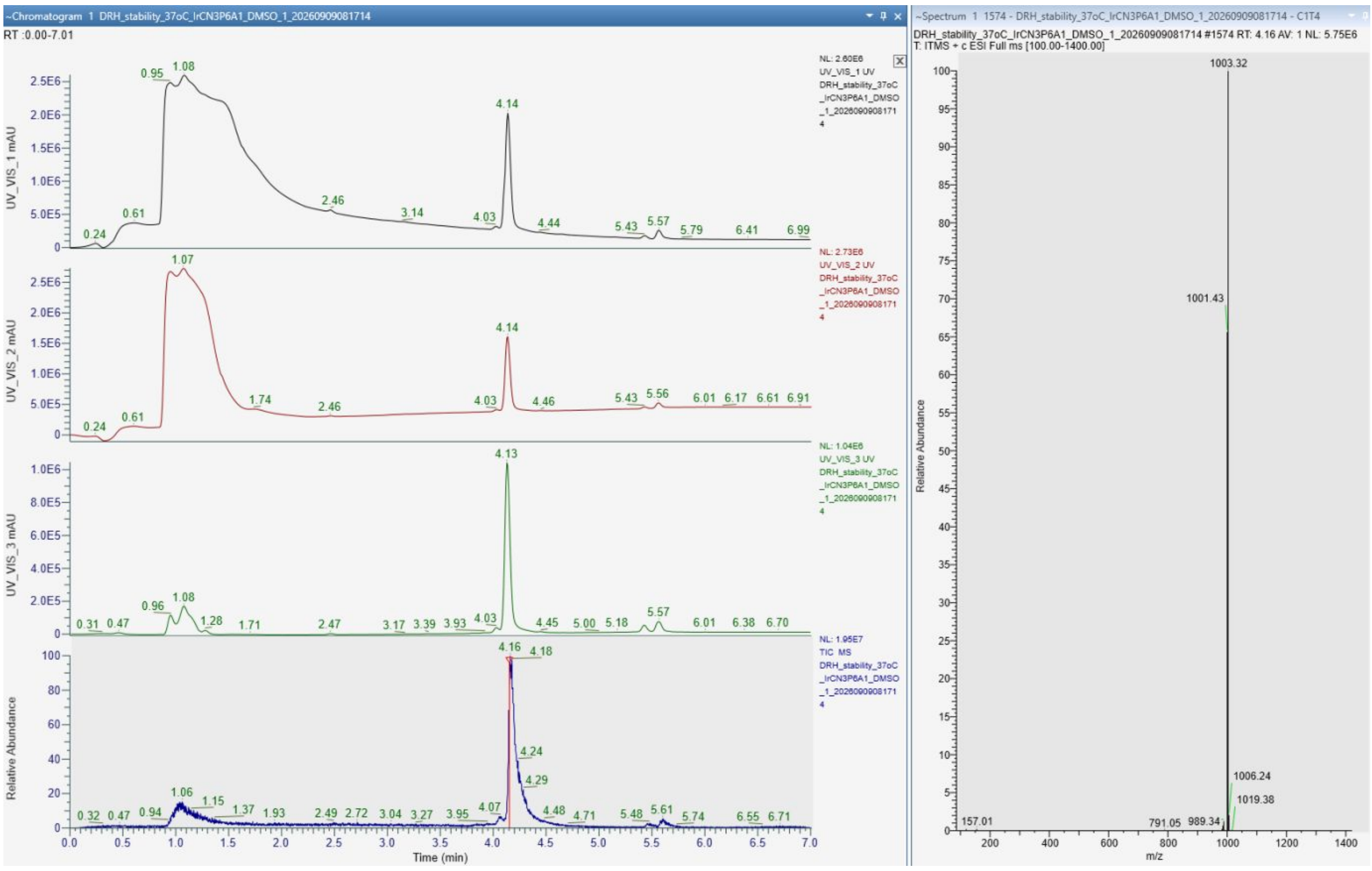

Figure S30: LC-MS trace (7 min method) of IrCN3(P6A1) in DMSO (100 μM) after 18 h at 37 °C

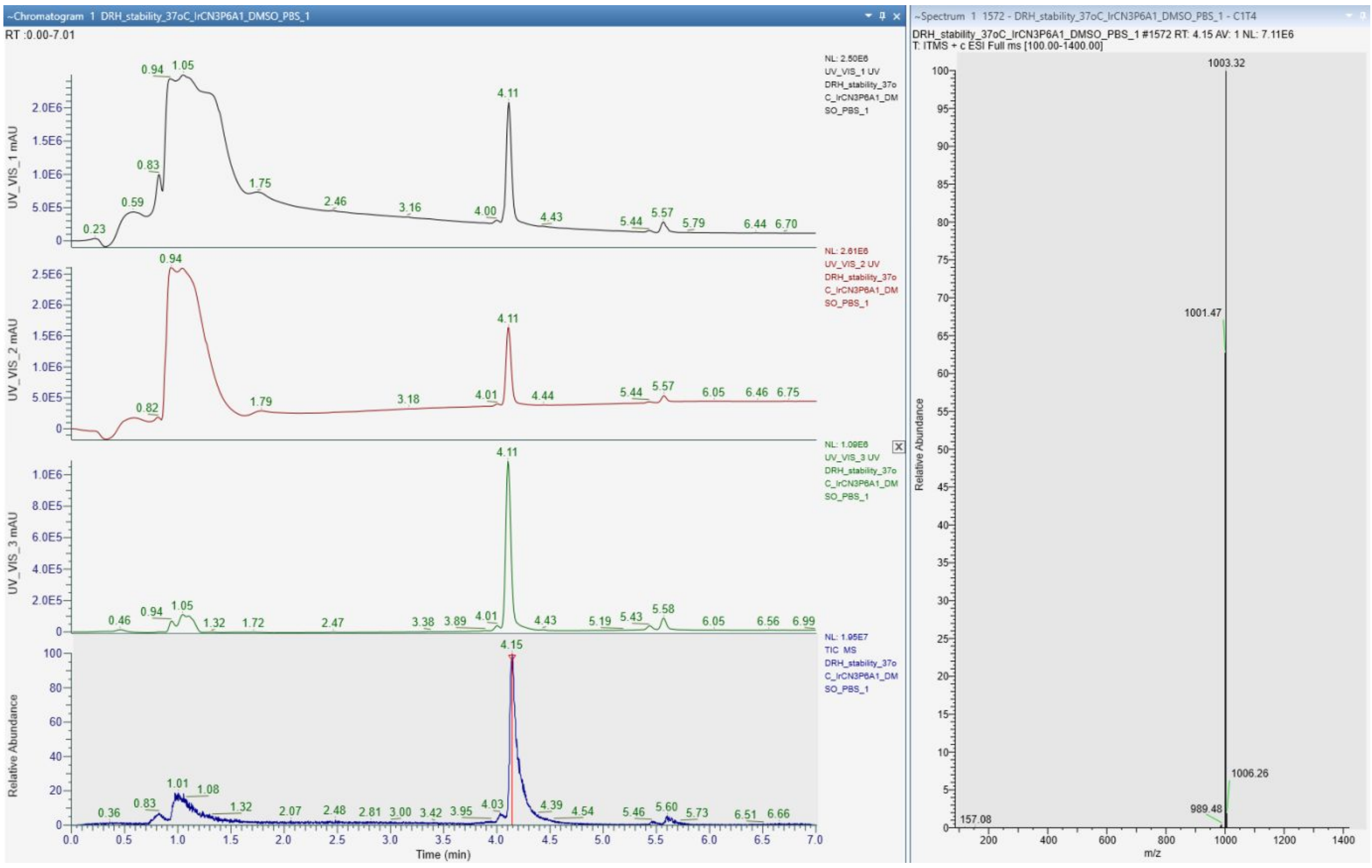

Figure S31: LC-MS trace (7 min method) of IrCN3(P6A1) in DMSO: PBS (1X) (100 μM) after 0 h at 37 °C

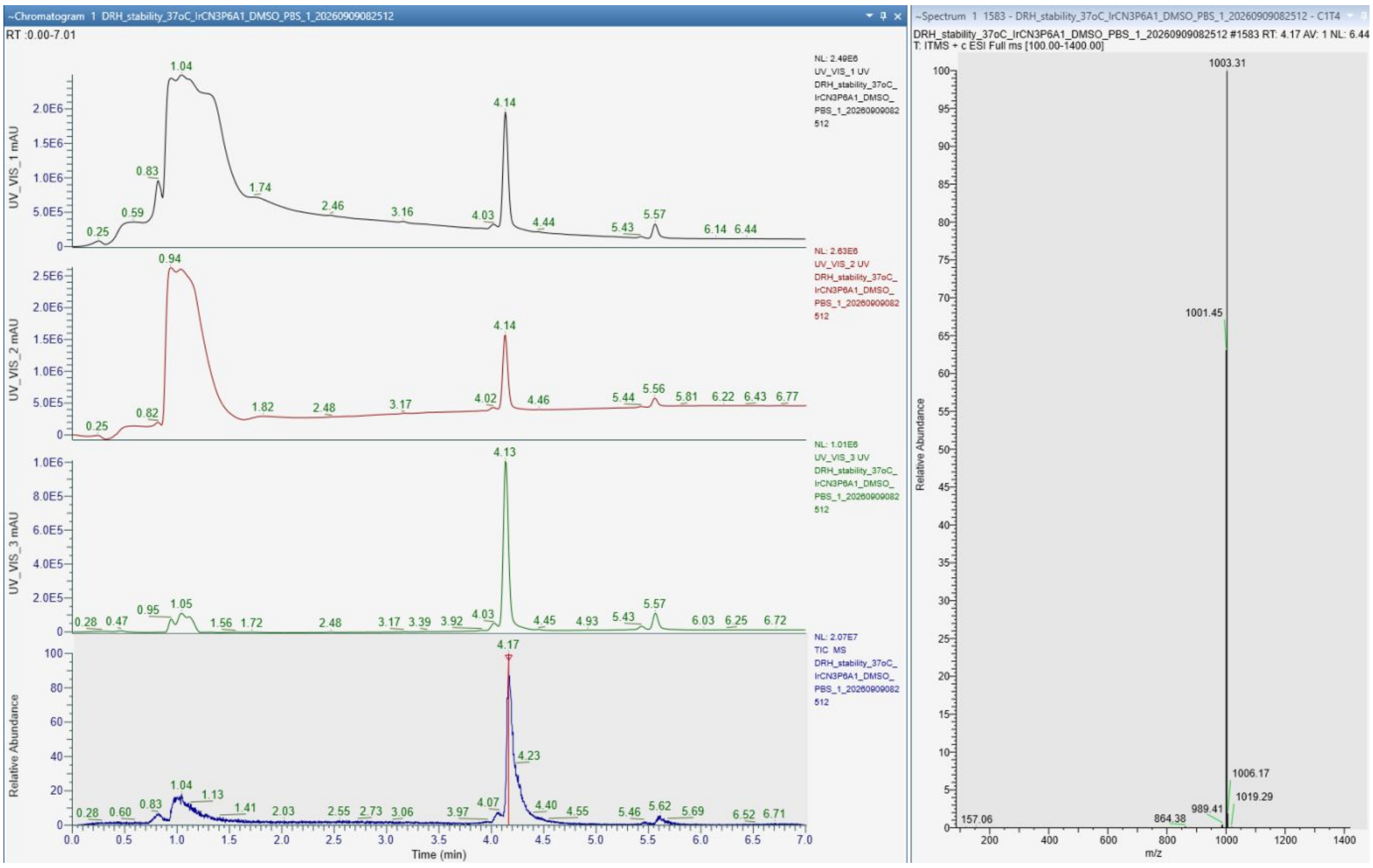

Figure S32: LC-MS trace (7 min method) of IrCN3(P6A1) in DMSO: PBS (1X) (100 µM) after 18 h at 37 °C

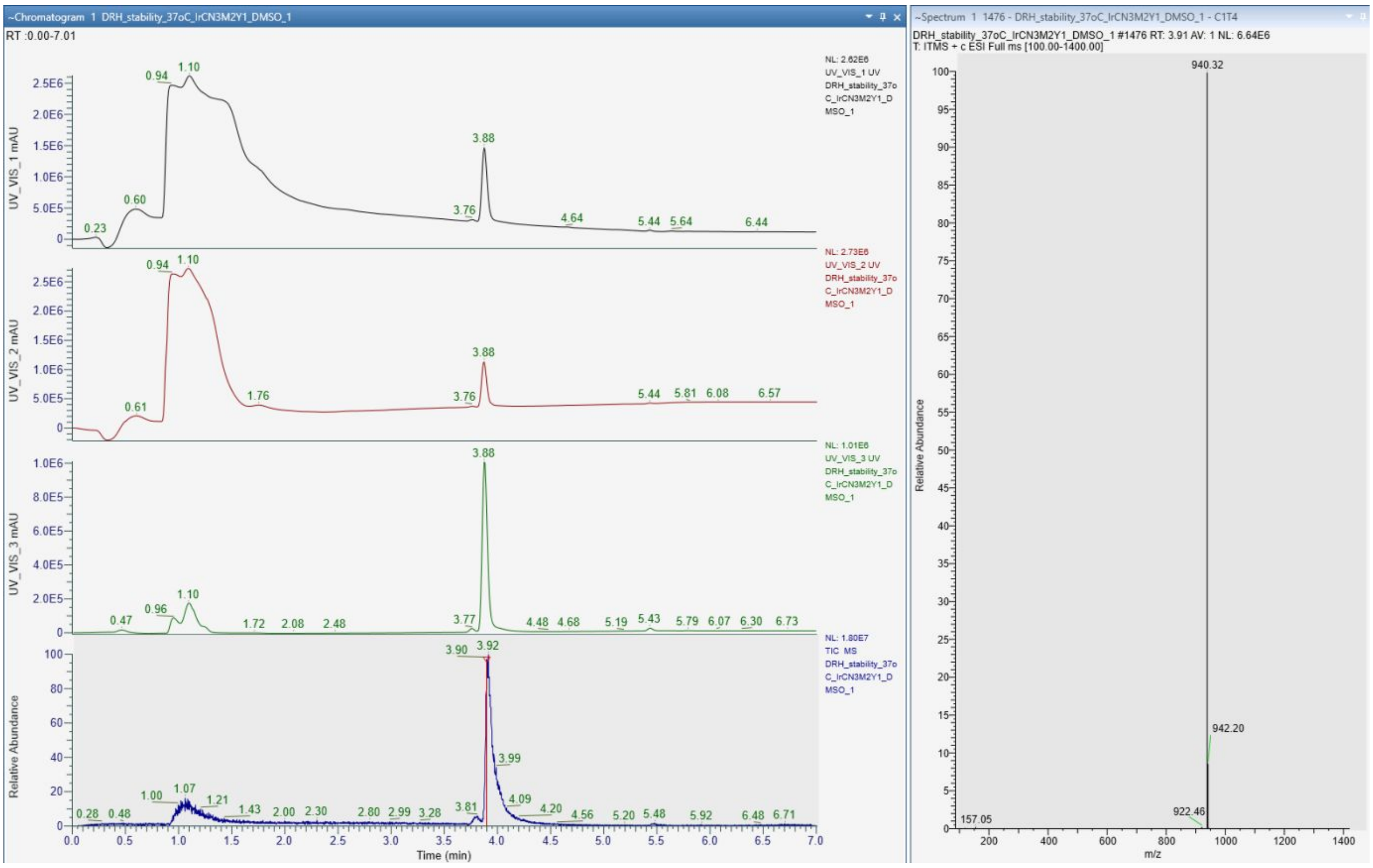

Figure S33: LC-MS trace (7 min method) of IrCN3(M2Y1) in DMSO (100 µM) after 0 h at 37 °C

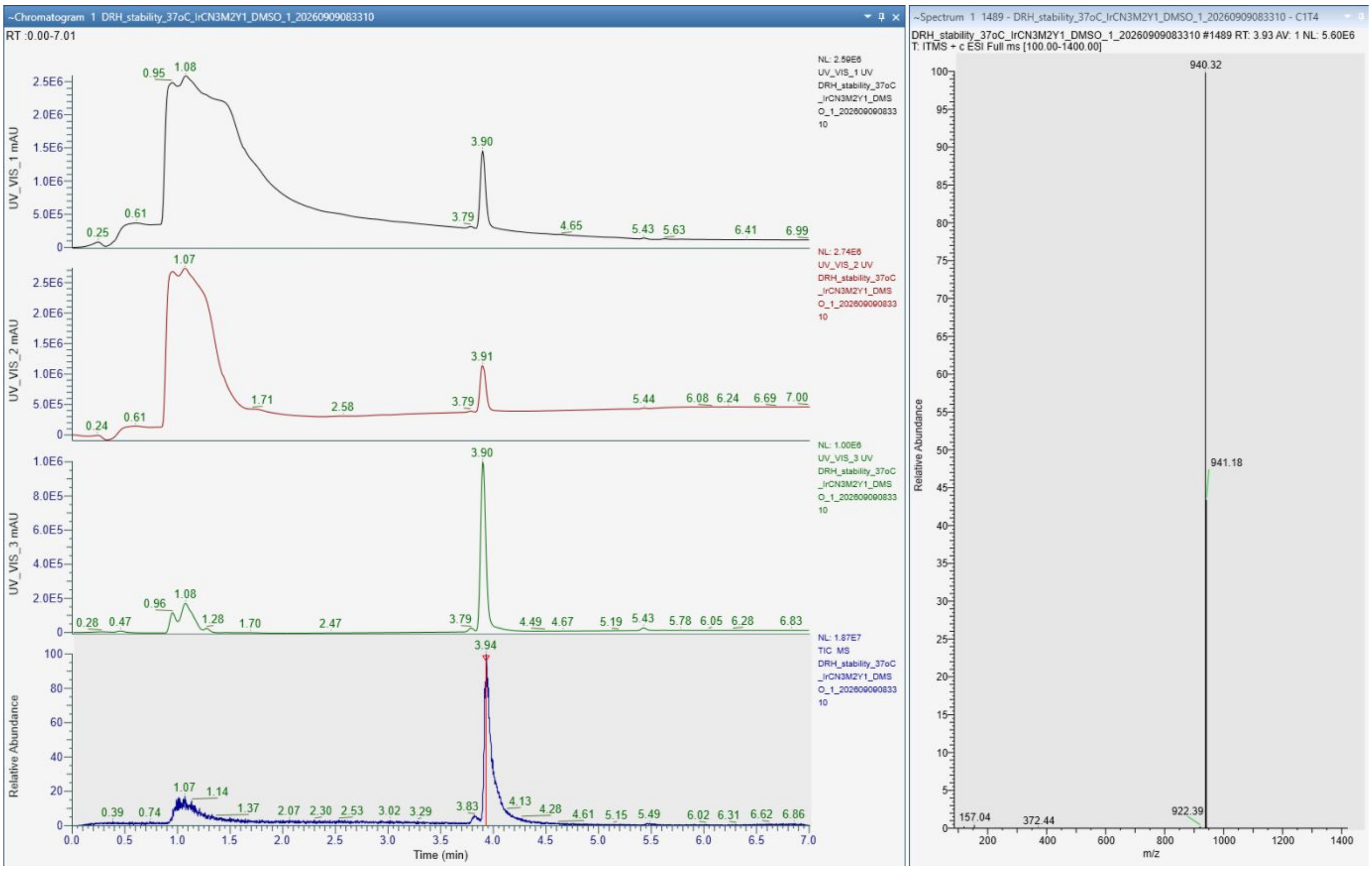

Figure S34: LC-MS trace (7 min method) of IrCN3(M2Y1) in DMSO (100 µM) after 18 h at 37 °C

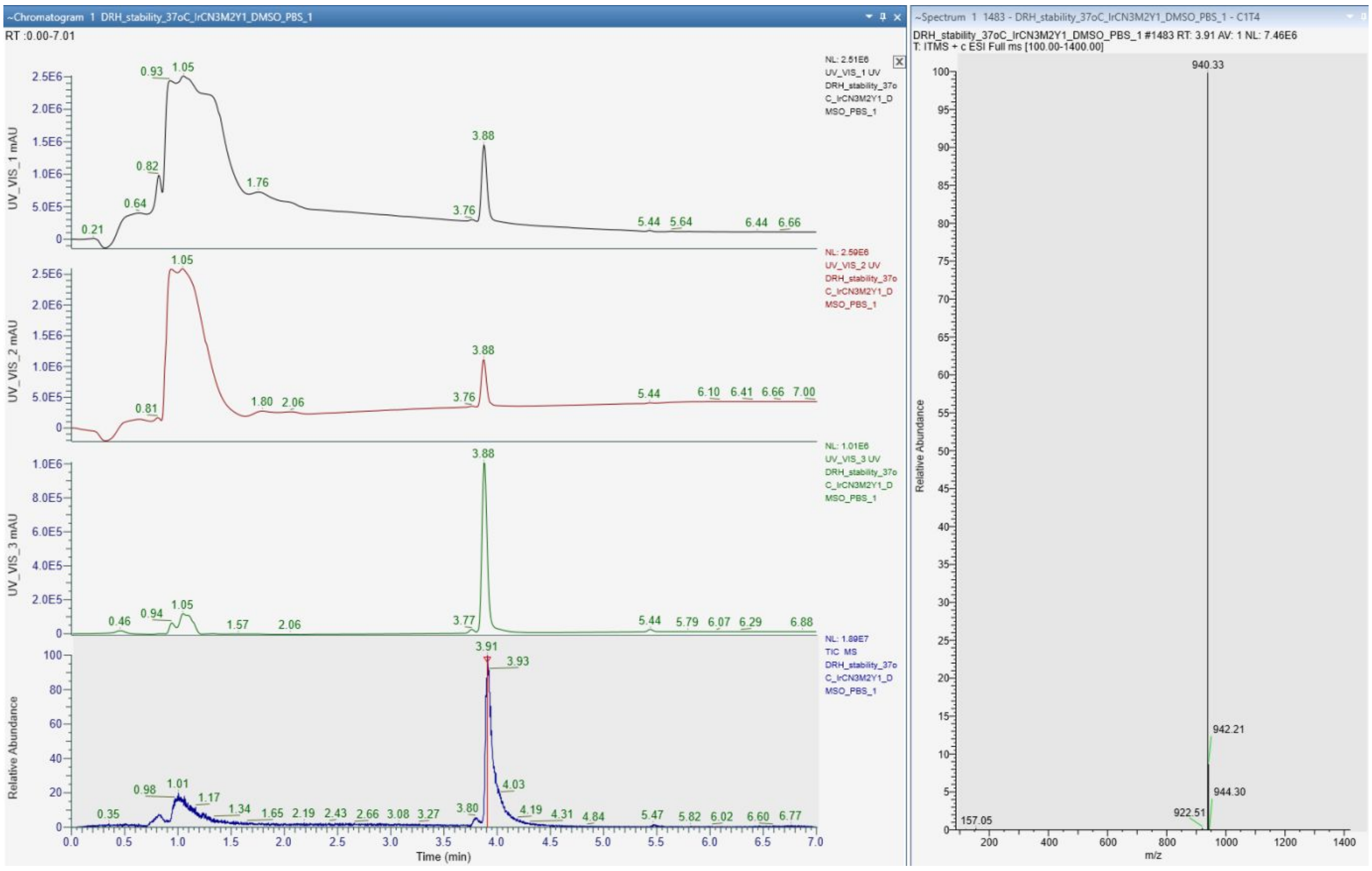

Figure S35: LC-MS trace (7 min method) of IrCN3(M2Y1) in DMSO: PBS (1X) (100 µM) after 0 h at 37 °C

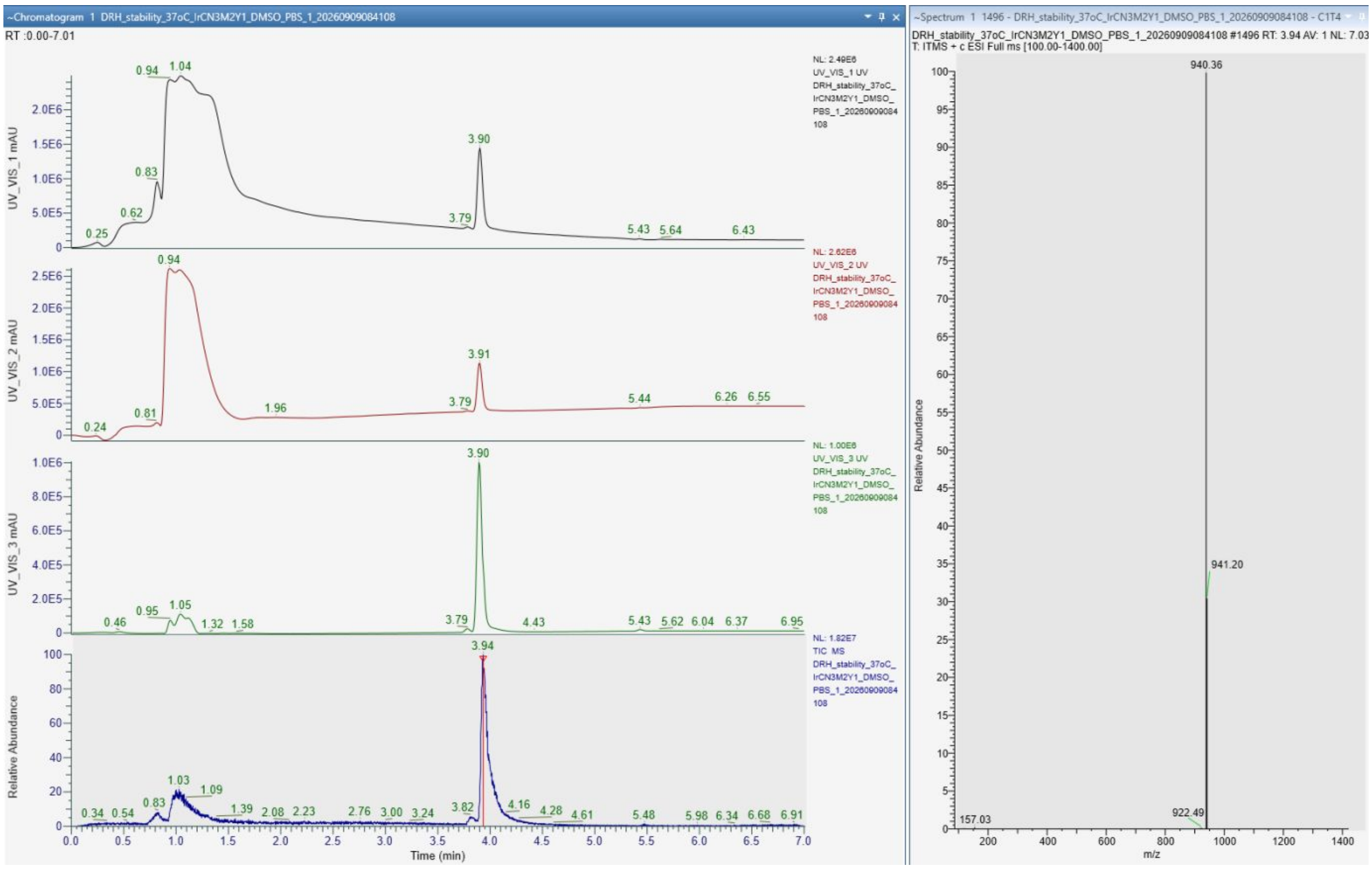

Figure S36: LC-MS trace (7 min method) of IrCN3(M2Y1) in DMSO: PBS (1X) (100 μM) after 18 h at 37 °C

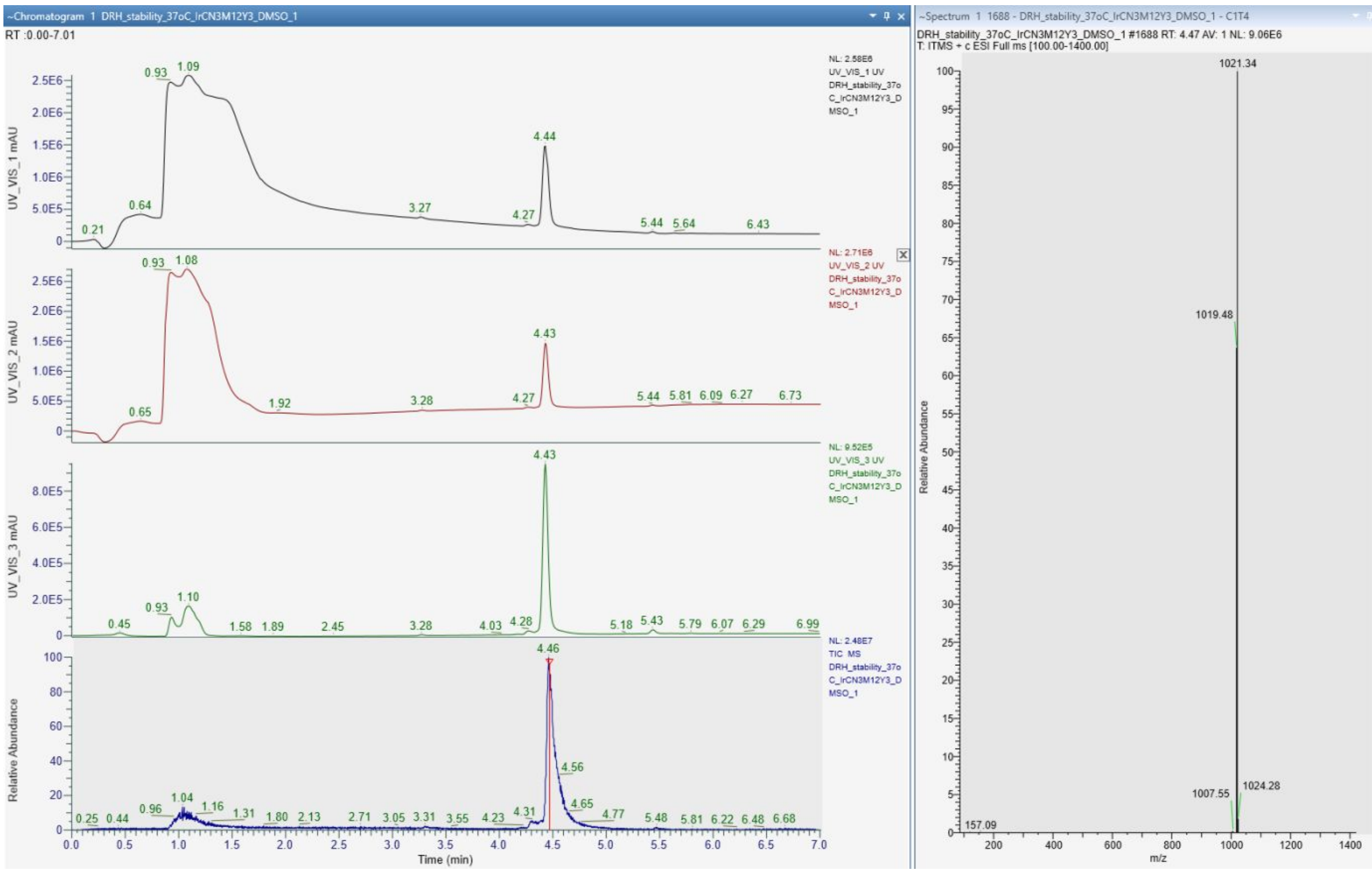

Figure S37: LC-MS trace (7 min method) of IrCN3(M12Y3) in DMSO (100 μM) after 0 h at 37 °C

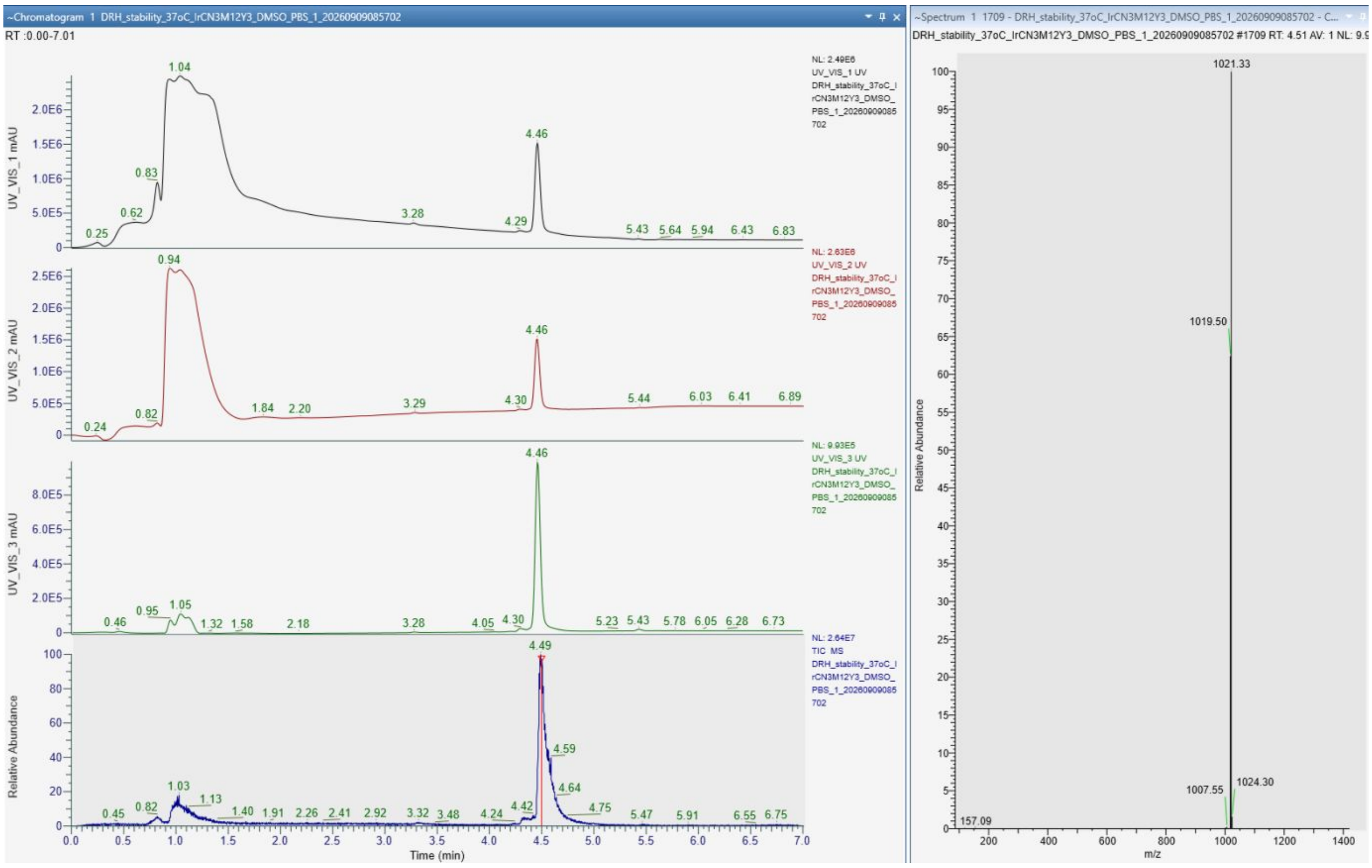

Figure S38: LC-MS trace (7 min method) of IrCN3(M12Y3) in DMSO (100 µM) after 18 h at 37 °C

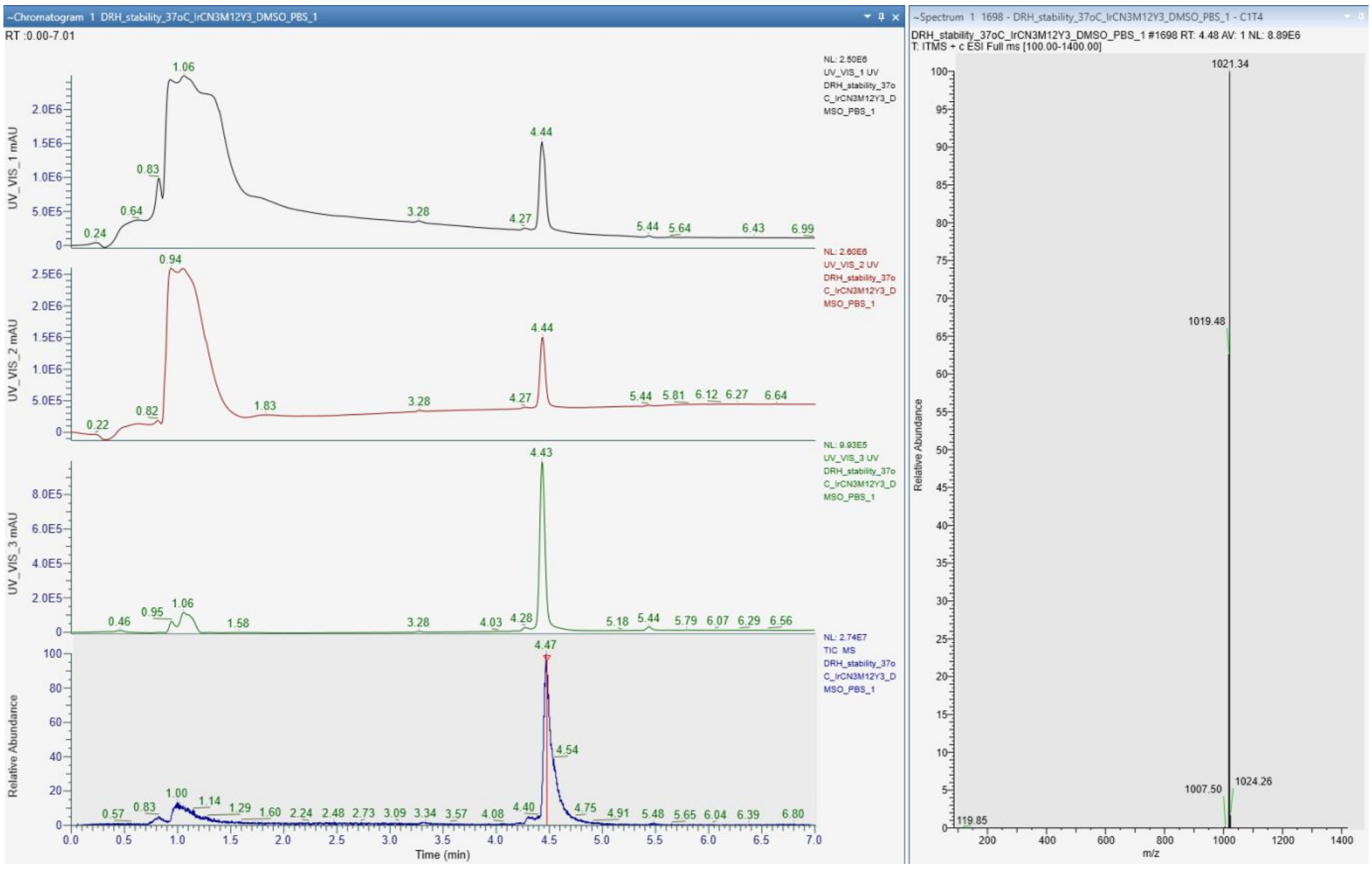

Figure S39: LC-MS trace (7 min method) of IrCN3(M12Y3) in DMSO: PBS (1X) (100 µM) after 0 h at 37 °C

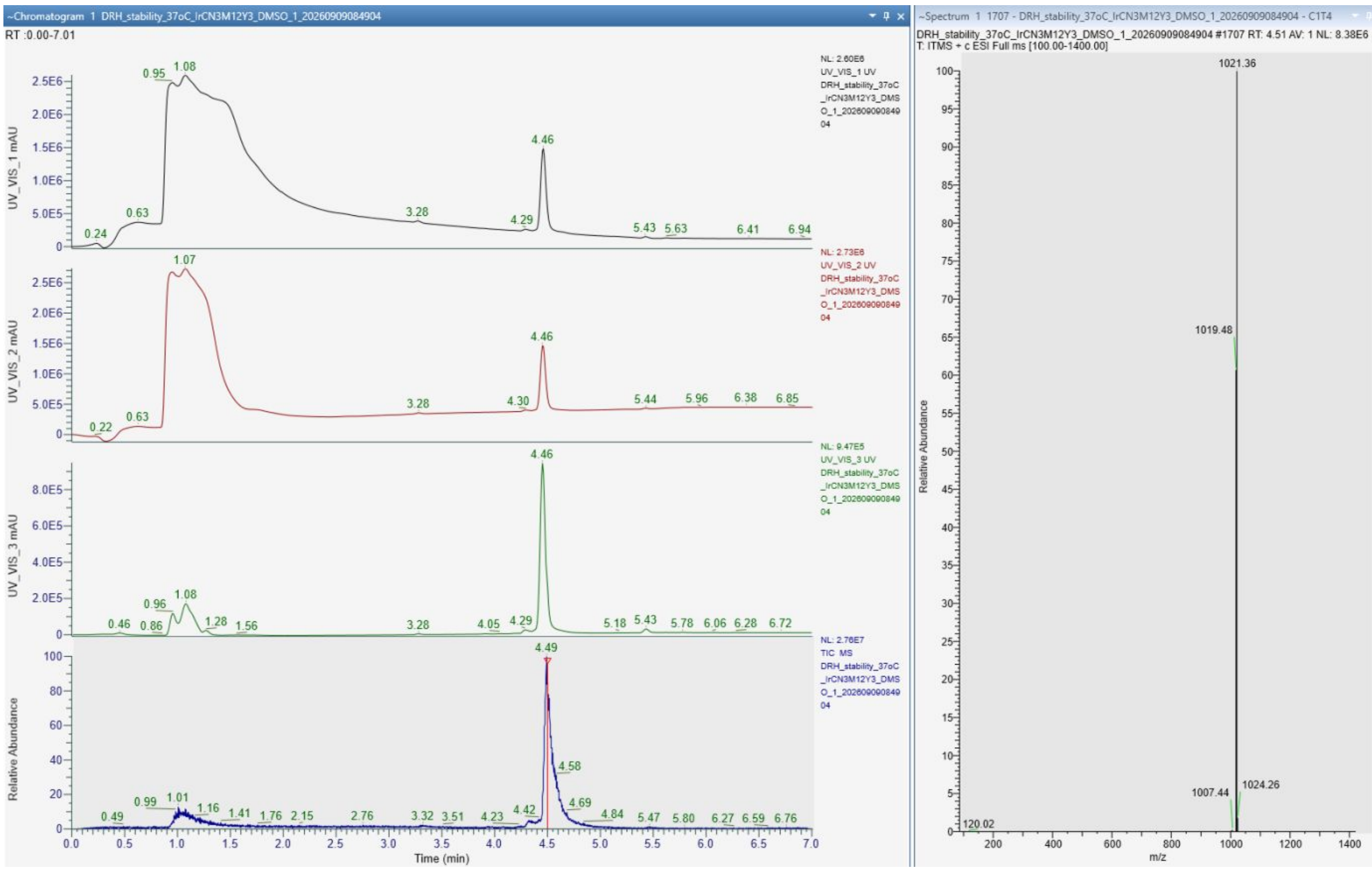

Figure S40: LC-MS trace (7 min method) of IrCN3(M12Y3) in DMSO: PBS (1X) (100 μM) after 18 h at 37 °C

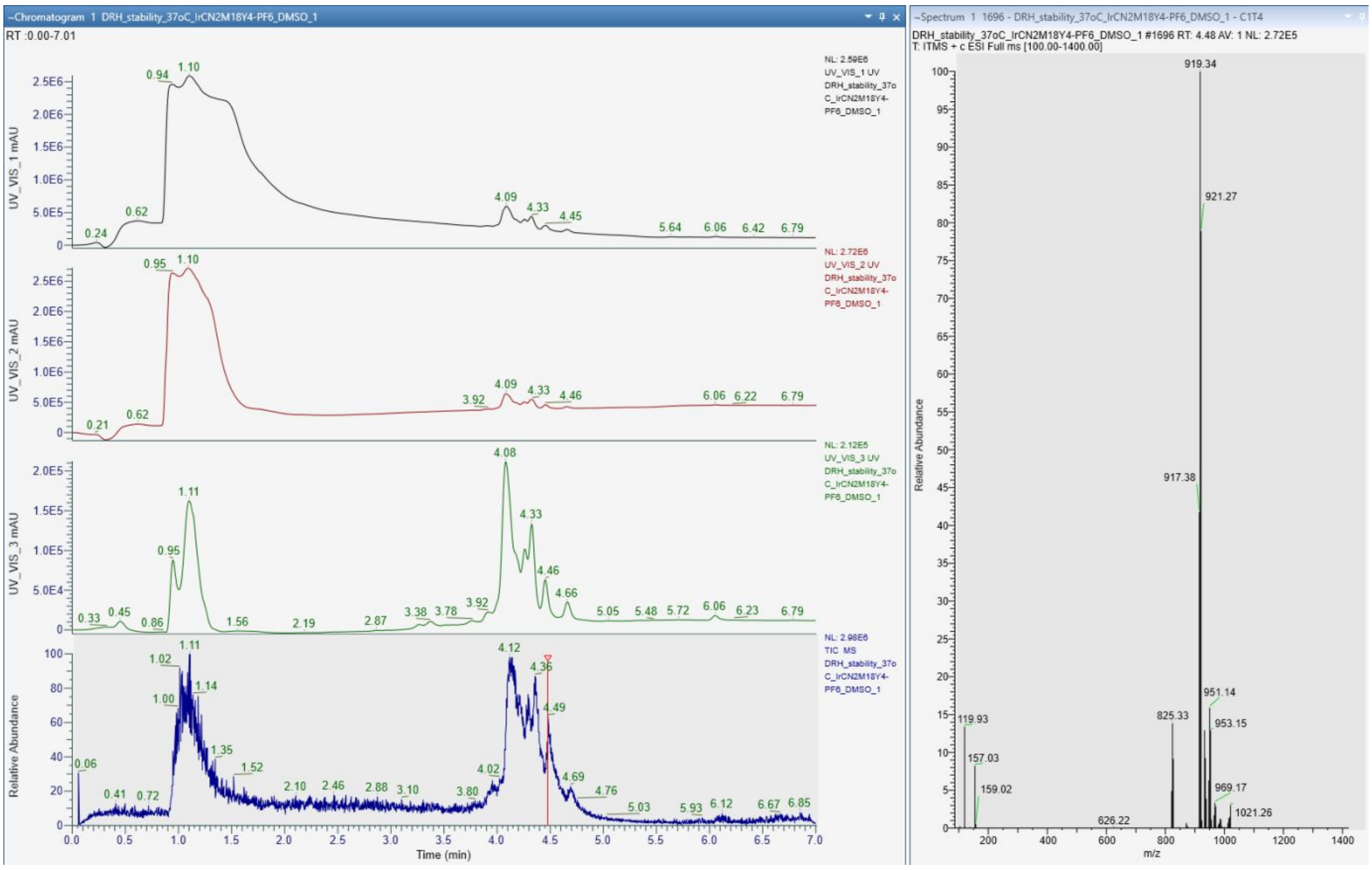

Figure S41: LC-MS trace (7 min method) of IrCN2(M18Y4)-PF_6 in DMSO (100 μM) after 0 h at 37 °C

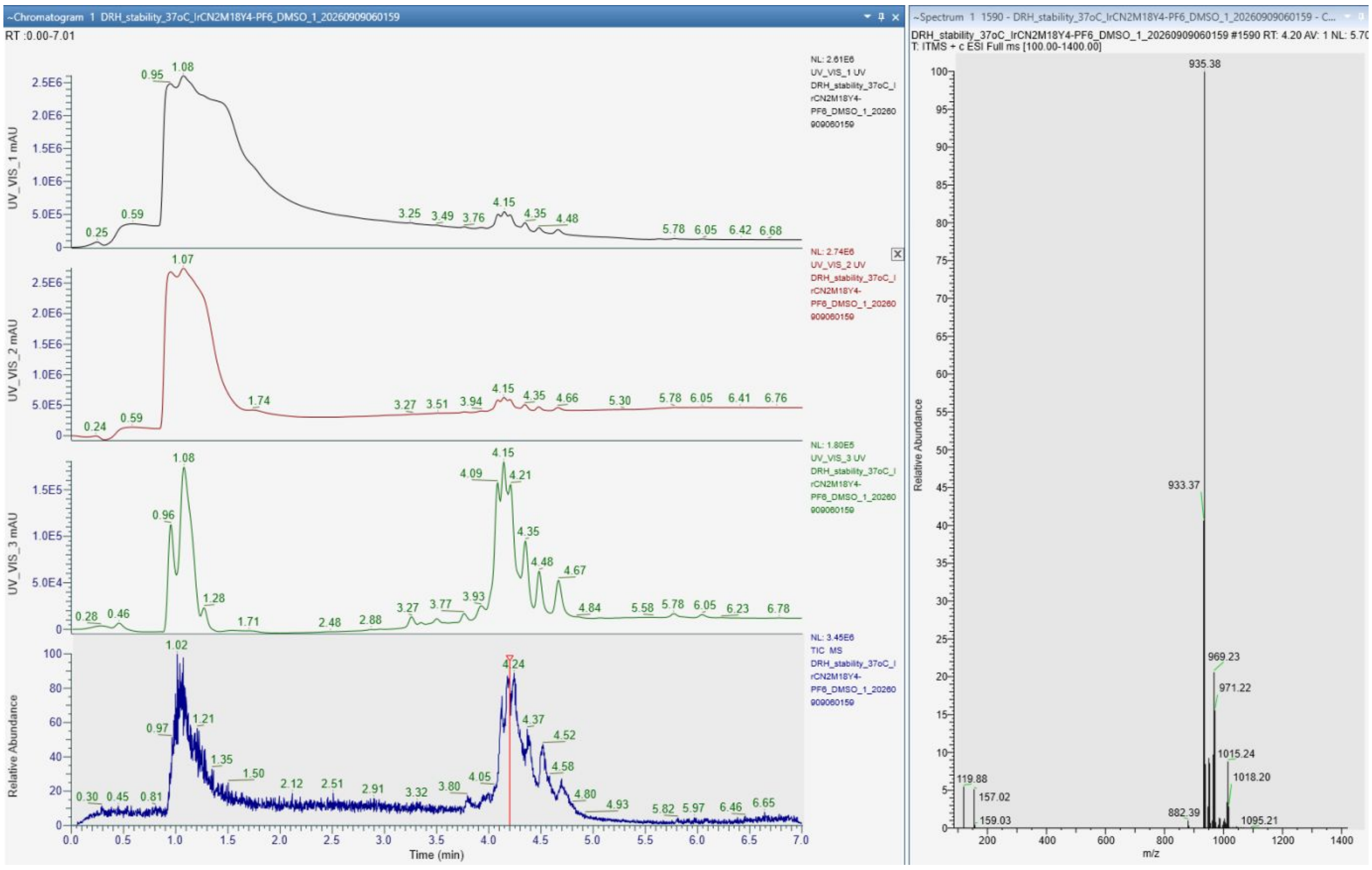

Figure S42: LC-MS trace (7 min method) of IrCN2(M18Y4)-PF$_6$ in DMSO (100 µM) after 18 h at 37 °C

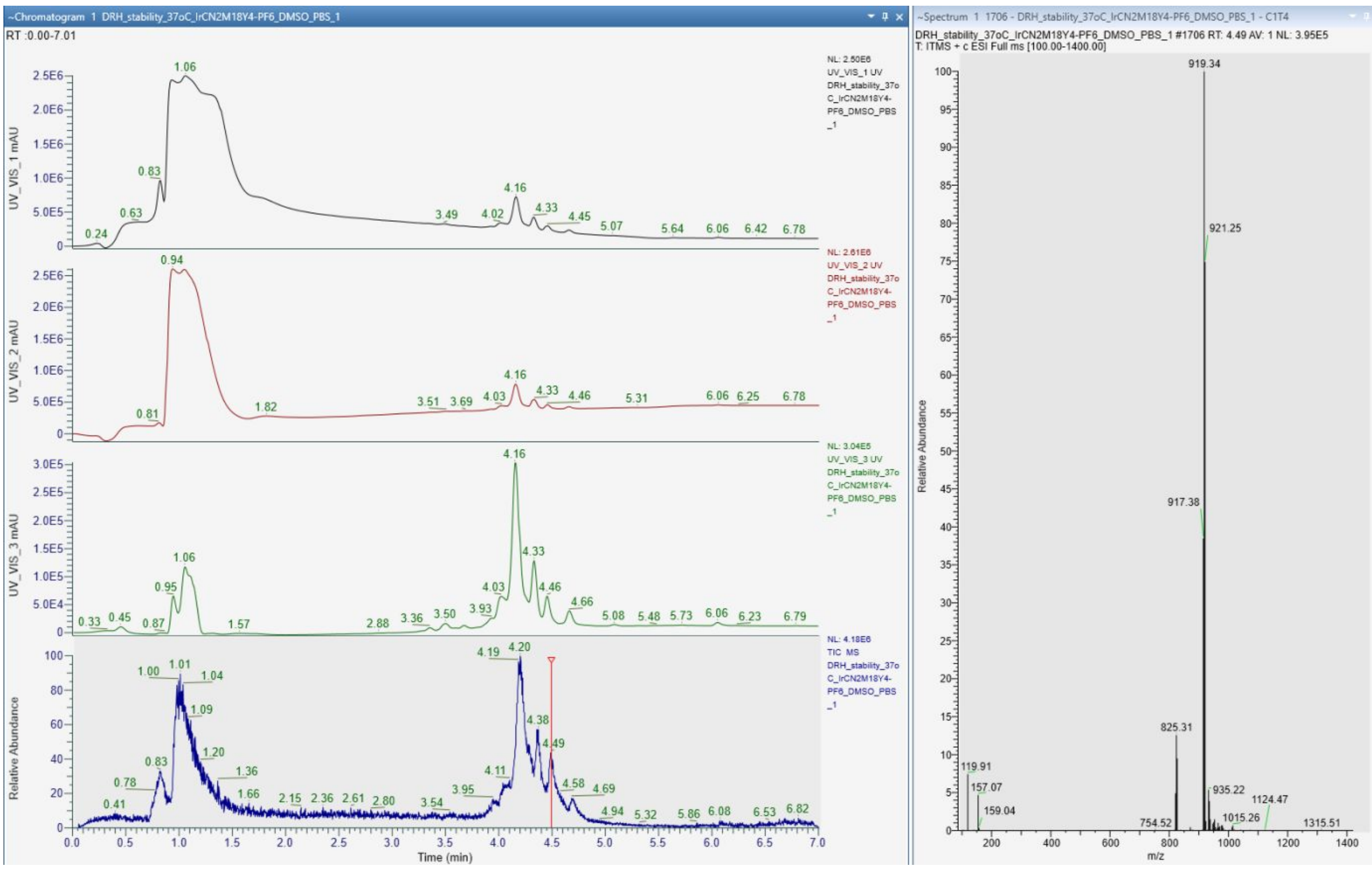

Figure S43: LC-MS trace (7 min method) of IrCN2(M18Y4)-PF$_6$ in DMSO: PBS (1X) (100 µM) after 0 h at 37 °C

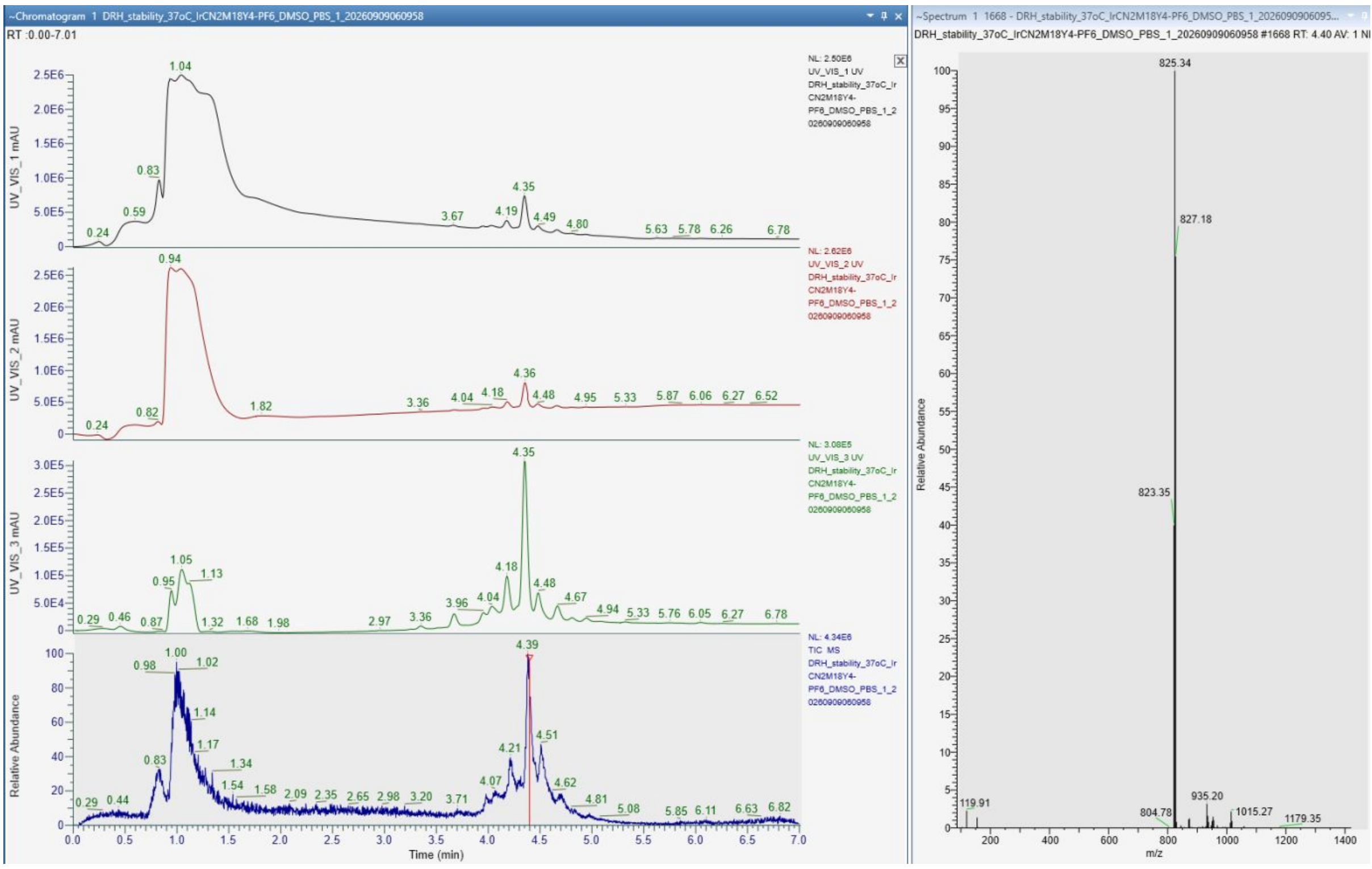

Figure S44: LC-MS trace (7 min method) of IrCN2(M18Y4)-PF_6 in DMSO: PBS (1X) (100 μM) after 18 h at 37 °C

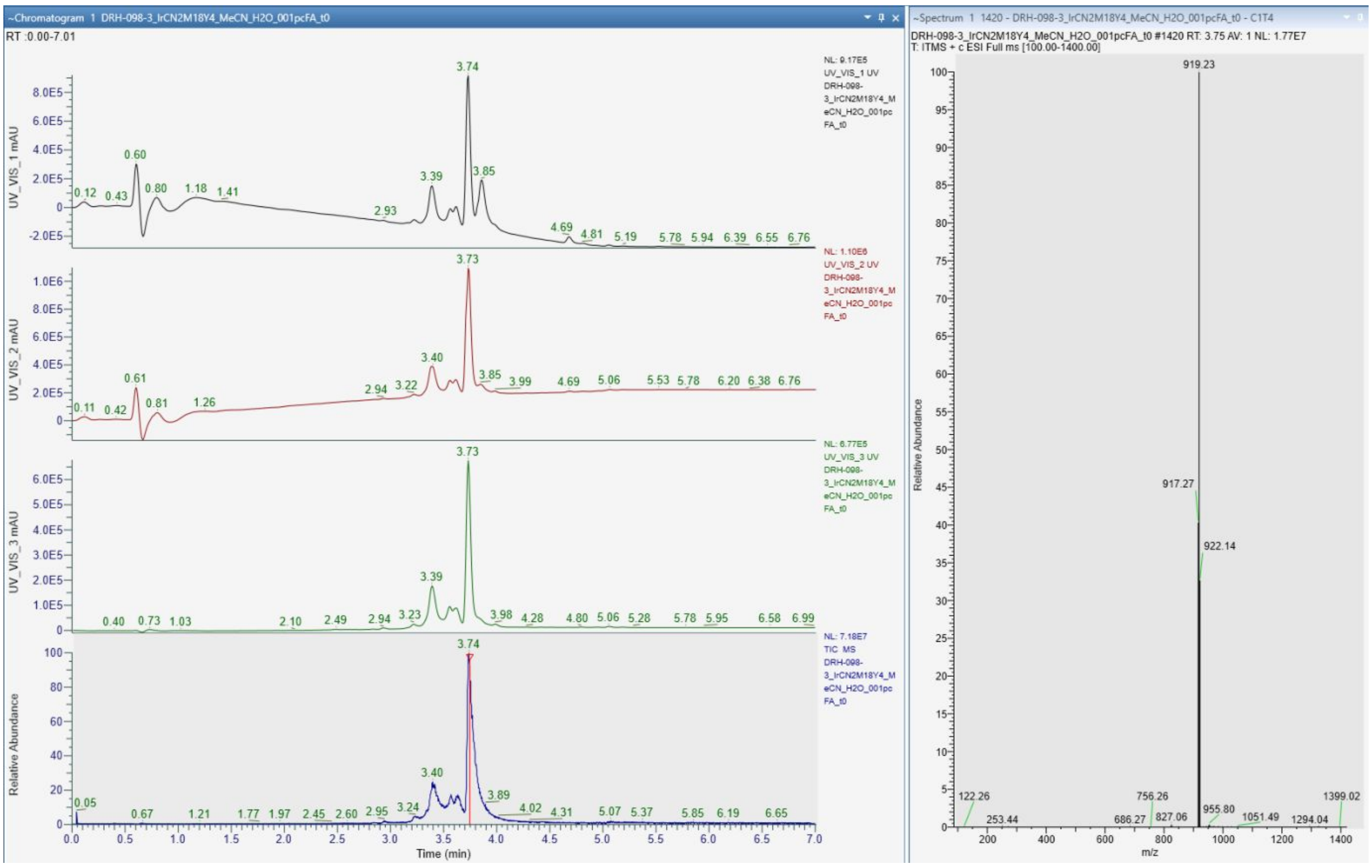

Figure S45: LC-MS trace (7 min method), stability of IrCN2(M18Y4) in MeCN:H_2O (60:40) with 0.01% formic acid. T = 0 h.

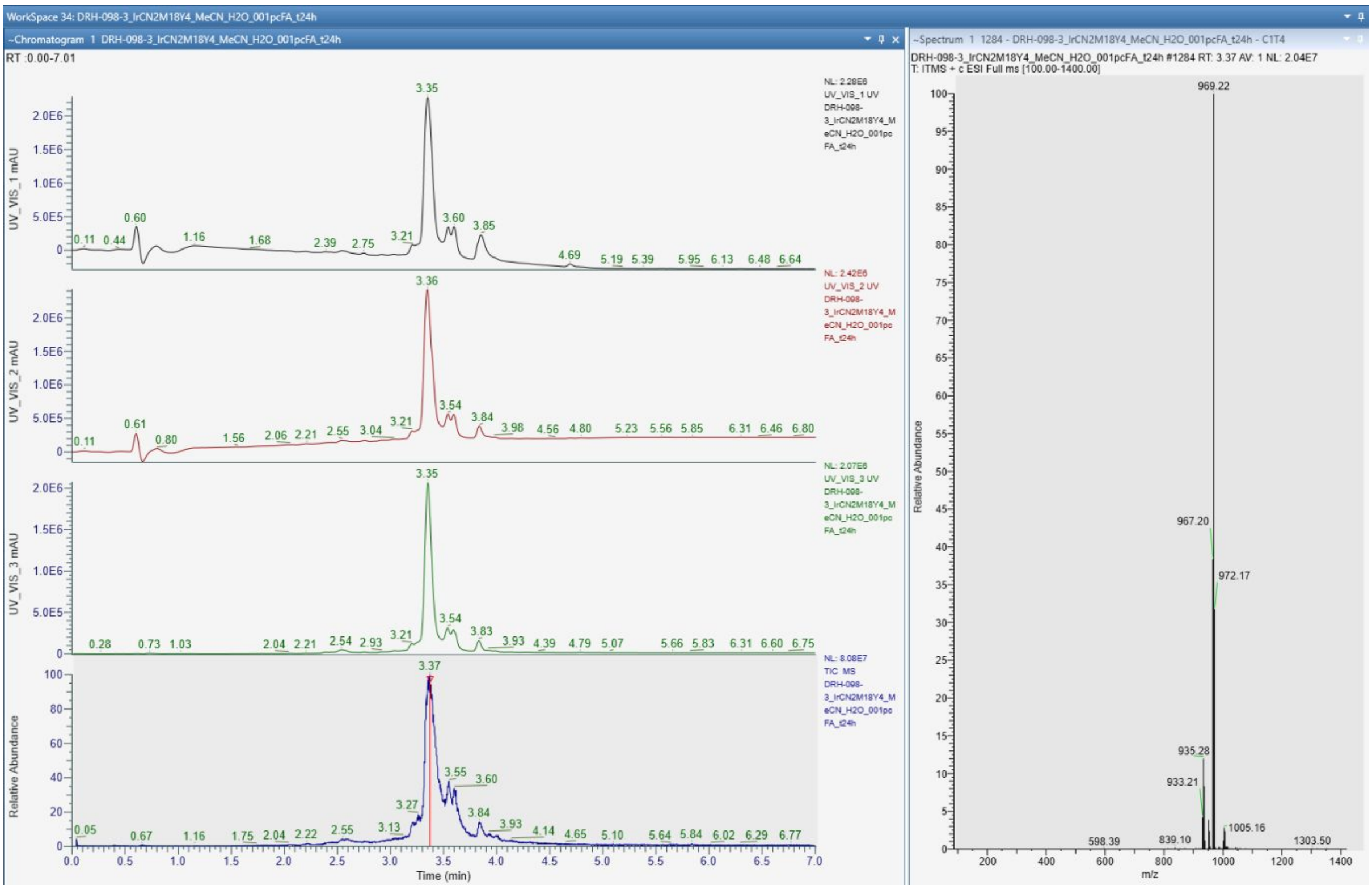

Figure S46: LC-MS trace (7 min method), stability of IrCN2(M18Y4) in MeCN:H_2O (60:40) with 0.01% formic acid. T = 24 h. Total degredation of IrCN2(M18Y4) over this time frame.

IrCN1P1A3

Stable in both cases.

IrCN1P8A1

Mostly stable, very slight degradation in DMSO (1% impurity). Slightly more pronounced degradation in PBS (5% impurity).

IrCN2P3A2

Significant degradation in both to a different product (3.91 min – IrCN2 dimer, confirmed by an authentic sample of IrCN2 dimer). Full change after o/n in DMSO. In PBS, slower, 45% impurity from degradation.

IrCN2P6A9

Stable in both cases.

IrCN3P6A1

Mostly stable, some small degradation (5.57 min - forming the IrCN3 dimer, confirmed by an authentic sample of IrCN3 dimer) over time in both cases (DMSO – 10% impurity, DMSO/PBS – 11% impurity).

IrCN3M2Y1

Stable in both cases.

IrCN3M12Y3

Stable in both cases.

IrCN2M18Y4-PF6

Very unstable in both cases. In DMSO, by the time the first sample had been run, significant degradation already observed. Degradation continued, funnelling to an unknown Ir containing species with a mass of 825.39. A different degradation profile and product was observed in DMSO:PBS. Alongside the acid sensitivity, this instability is postulated to be caused in part by hydrolysis of furan ring.

5. LCMS Processing

LCMS spectra were automatically processed using bespoke Python and R code. This processing was used to obtain purity data (as peak area% of the total spectrum), to characterize the compounds (using mass spec associated with the UV peak), to obtain retention times of the compounds as a pseudo logP measurement, and to generate the spectra in the supplementary data associated with this paper.

As a workflow, the LCMS data was obtained from the ThermoScientific LCMS as .raw files. These files were converted to .mzml files using the ThermoRawFileParser (version 1.4.4) integrated with Python.[4] .mzml files were then converted into .csv files using R code (using the RaMS library). A list of target masses for the complexes in the files being processed was prepared. Then, using custom Python code, the mass list was searched and filtered for the target mass, and the associated UV peak (using UV3, 254 nm) was found. The UV peak was integrated to determine the %peak area as a total area, and the UV trace and extracted ion chromatogram were plotted in a single graph (using normalized data). The peak was labeled, and its retention time extracted.

To allow for accurate integration and account for baseline drift, a baseline correction was used, where the UV3 trace of a blank sample (60% MeCN in H_2O) was subtracted from the UV trace. An example of the spectra generated is shown below.

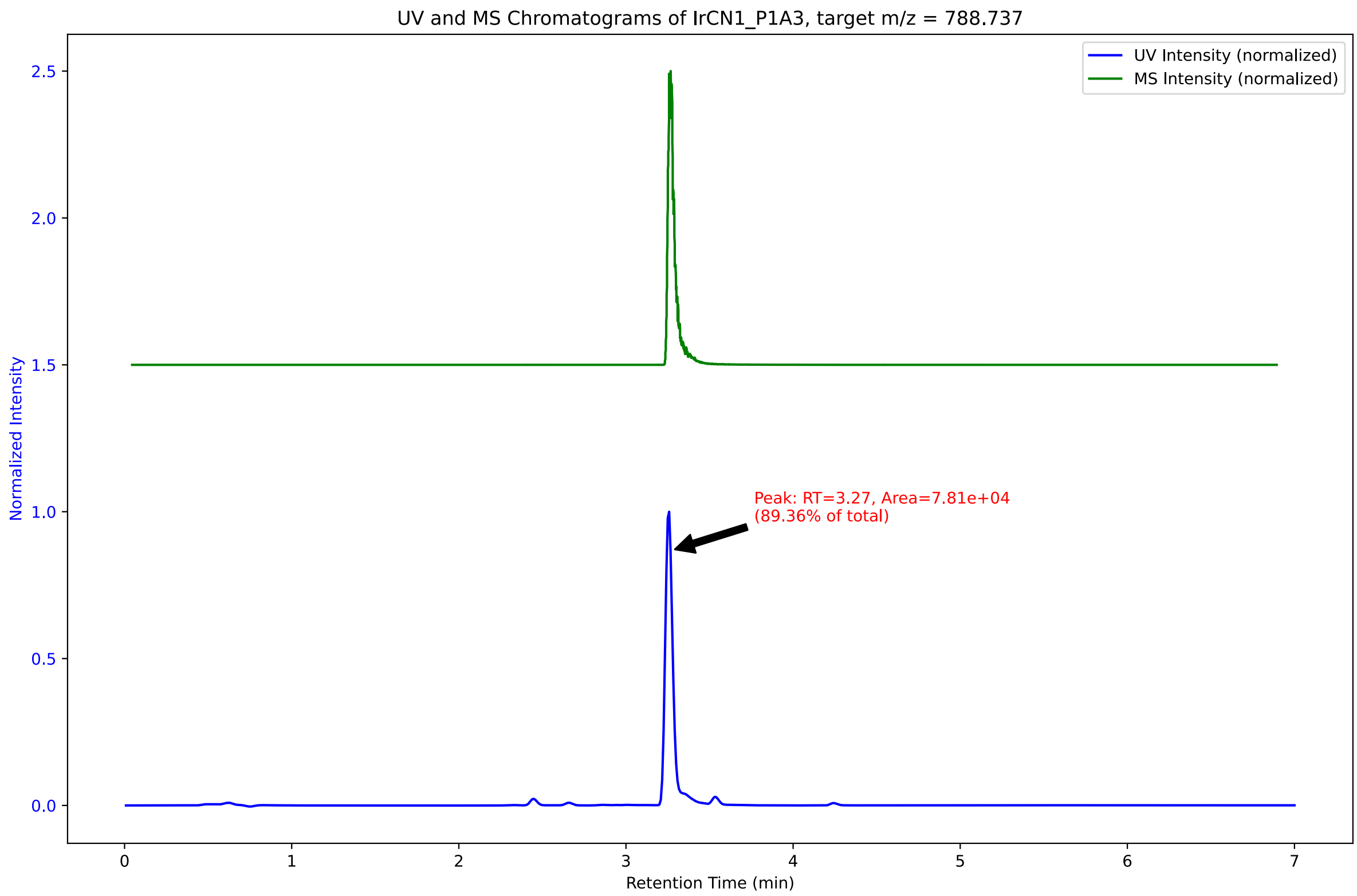

Figure S47: An example automatically processed LCMS spectrum for IrCN1(P1A3) (crude). The lower graph is the UV trace at 254 nm, and the upper trace is the extracted ion chromatograph for the target mass of the complex.

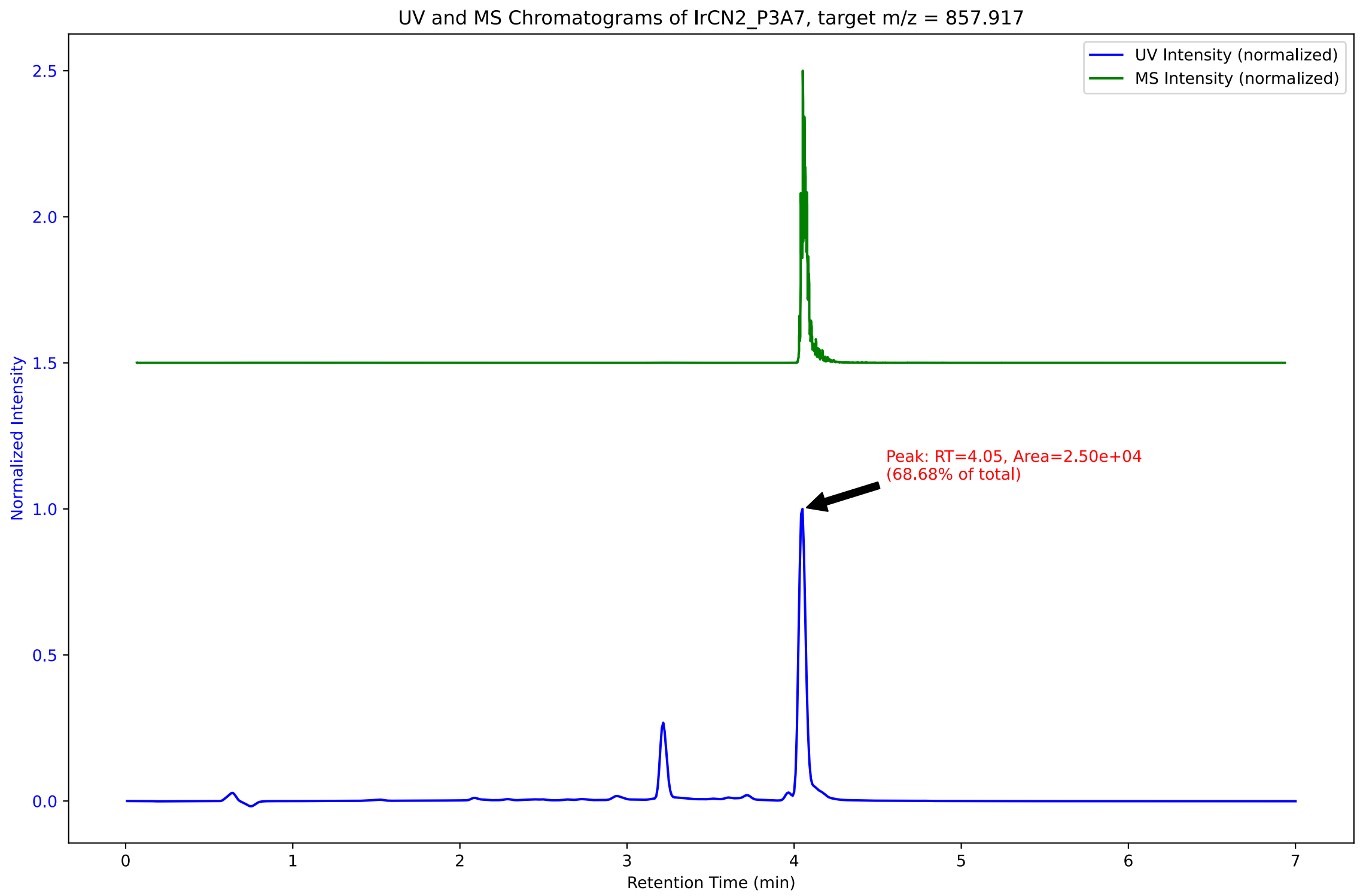

Figure S48: An example automatically processed LCMS spectrum for IrCN2(P3A7) (crude). The lower graph is the UV trace at 254 nm, and the upper trace is the extracted ion chromatograph for the target mass of the complex.

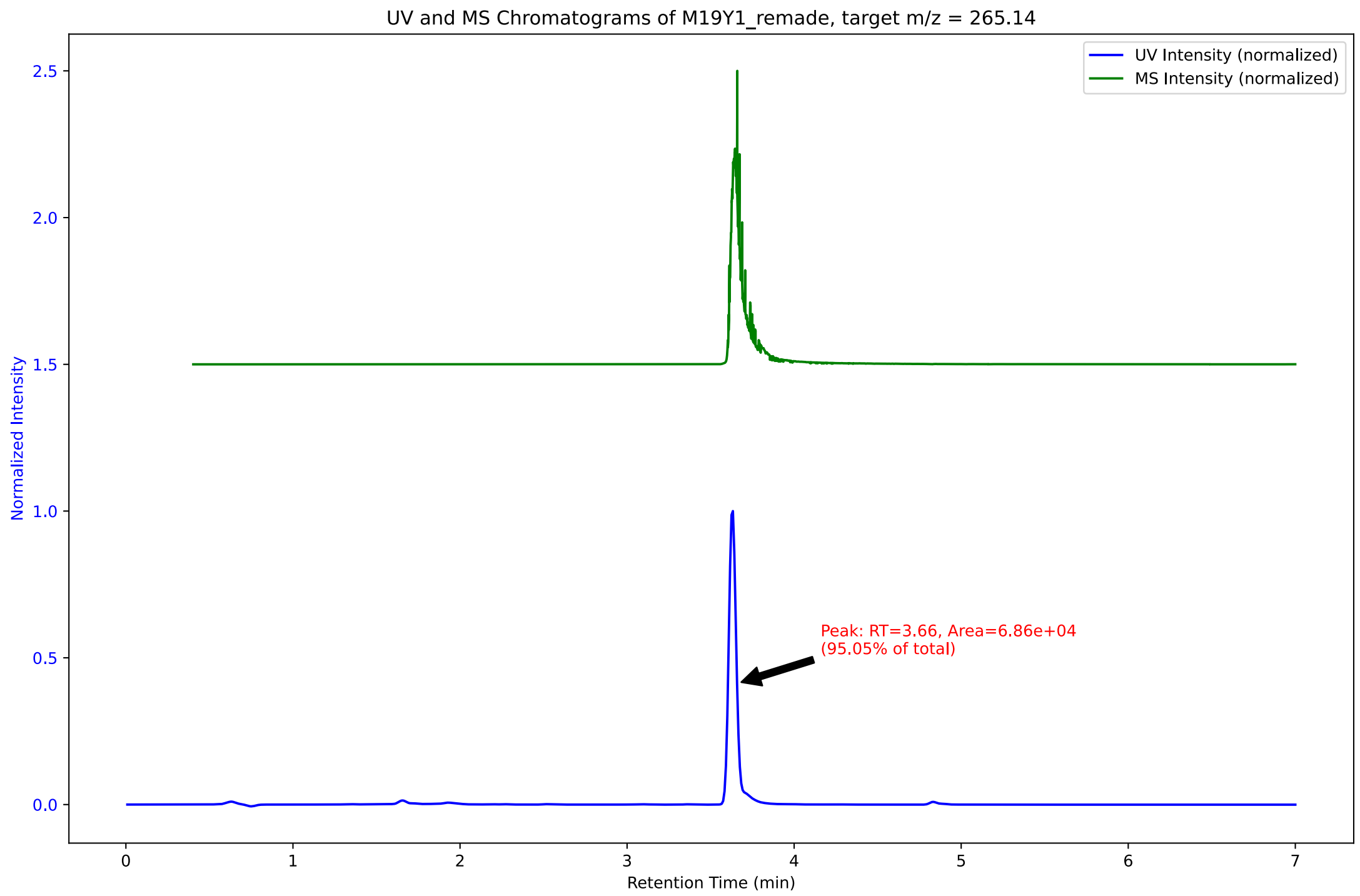

Figure S49: An example automatically processed LCMS spectrum for M19Y1 (crude). The lower graph is the UV trace at 254 nm, and the upper trace is the extracted ion chromatograph for the target mass of the complex.

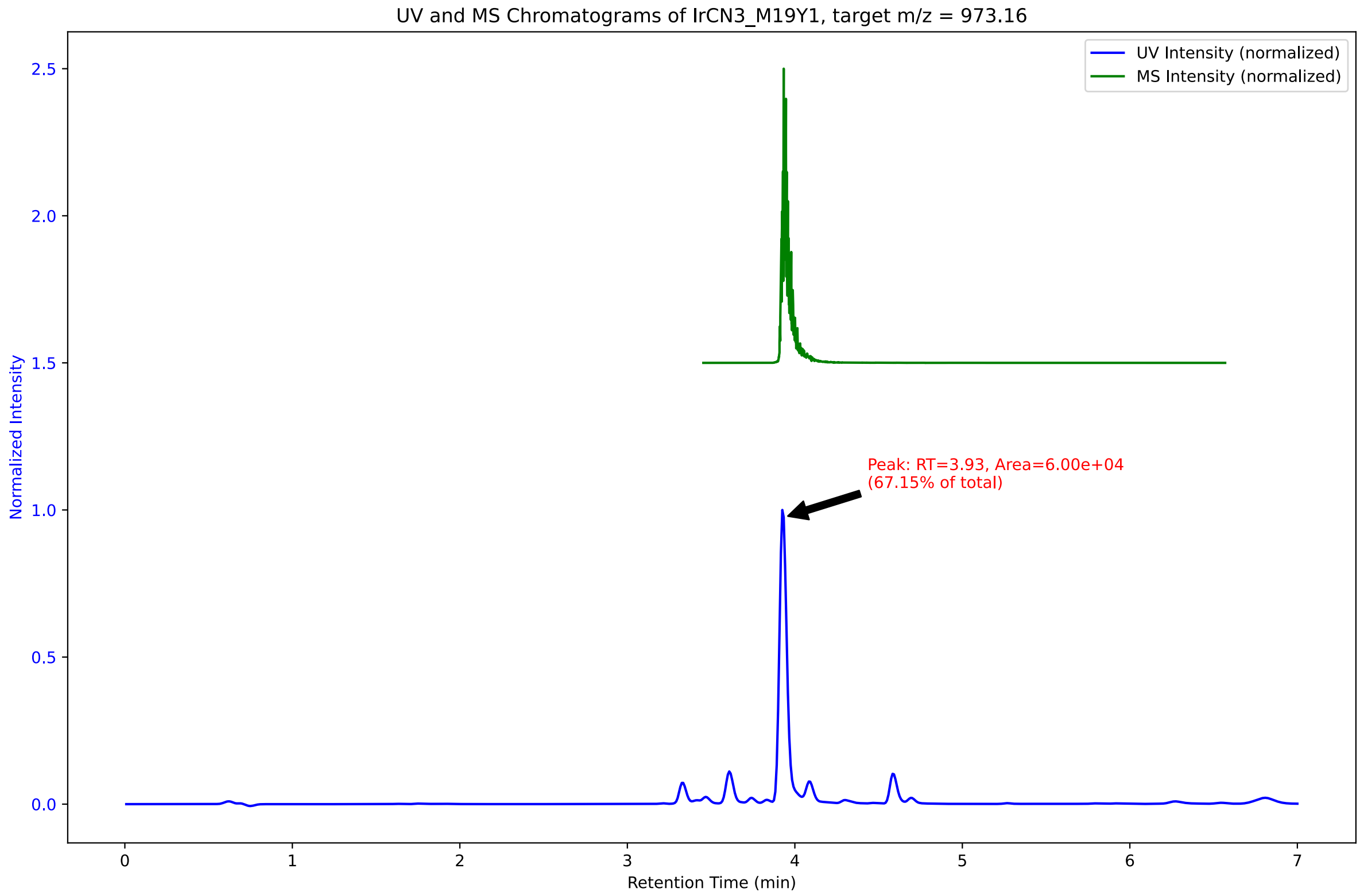

Figure S50: An example automatically processed LCMS spectrum for IrCN3(M19Y1) (crude). The lower graph is the UV trace at 254 nm, and the upper trace is the extracted ion chromatograph for the target mass of the complex.

5.1 LCMS Stability of Crude Reaction Mixtures over 1 year

To assess the stability of the IrCN complexes, a selection of compounds was re-analyzed by LC-MS after approximately 1 year of being stored in the solvent mixtures at 5 °C. The compounds were chosen to get a representative view of stability, and all were tested for MIC. Overall, the target complexes have either increased slightly in their conversion or stayed the same (For IrCN1/2-SB, improvement from -3 to +26% compared to the initial conversion). The main exception to this is the IrCN3-Schiff library, which contained a significant amount of yellow precipitate in each well. Each well was aspirated thoroughly before sampling. This iridium-containing precipitate (4.59 min, consistent across all IrCN3 samples), appears to be a common degradation product for IrCN3 containing samples. It appears based on the degradation of the IrCN3 target complexes (-14 – -39% change compared to the initial conversion) that the electron-poor IrCN3-Schiff library may be subject to Schiff-base hydrolysis over time, while degradation is not observed for the other libraries. This degradation is not so pronounced for the IrCN3-Click library (+8 - -7% change compared to the initial conversion), which does not have the same mechanism of hydrolysis. However, the IrCN2-Click library does show increases in a common iridium-containing impurity (3.24 min, consistent across samples, +13 - -22% change compared to the initial conversion to the target complex), showing certain compounds in this particular IrCN-Click library may slowly degrade in solution under prolonged storage conditions. Processed LC-MS spectra can be found in the associated collated LC-MS spectra data file.

Table S2: LC-MS data showing the change in peak area% (254 nm) for a selection of compounds. Peak area% >100 is due to small differences in the base line processing.

Compound	Original Peak area%	Peak area% after 1 year storage	Difference	Qualitative Comments
IrCN1(P1A2)	92.6	118.9	26.2	Same as before, no change
IrCN1(P2A11)	65.4	73.7	8.3	Similar to before, slightly purer
IrCN1(P4A5)	92.5	100.6	8.2	Similar to before, slightly purer
IrCN1(P6A9)	84.4	91.9	7.6	Similar to before, slightly purer
IrCN2(P1A1)	82.6	97.0	14.4	Similar to before, slightly purer
IrCN2(P2A5)	86.1	90.5	4.3	Similar to before, slightly purer
IrCN2(P5A9)	65.0	62.4	-2.6	Same as before, no change
IrCN2(P6A3)	82.2	83.3	1.0	Similar to before, slightly purer
IrCN3(P1A10)	87.6	72.9	-14.7	Moderate increase in impurities, still the main species. Increase in IrCN3 dimer
IrCN3(P4A1)	51.1	29.3	-21.8	Significant increase in in IrCN3 dimer
IrCN3(P6A7)	48.2	23.0	-25.2	Significant increase in impurities. IrCN3 Dimer main species
IrCN3(P7A2)	70.8	31.7	-39.1	Significant increase in in IrCN3 dimer
IrCN1(M2Y1)	85.4	94.0	8.6	No change
IrCN1(M8Y2)	91.5	88.8	-2.7	No change
IrCN1(M11Y3)	48.1	54.8	6.8	Slight change, but broadly similar
IrCN1(M20Y4)	68.5	70.9	2.4	No change
IrCN2(M9Y1)	86.4	88.7	2.3	No change
IrCN2(M7Y2)	78.2	90.8	12.6	no change
IrCN2(M12Y3)	51.0	38.1	-12.9	Significant increase in in IrCN2 dimer
IrCN2(M18Y4)	54.8	33.2	-21.7	Significant increase in in IrCN2 dimer
IrCN3(M13Y1)	64.4	72.7	8.3	Similar to before, slightly purer
IrCN3(M22Y2)	58.4	57.1	-1.3	Similar to before, slightly less pure
IrCN3(M14Y3)	43.7	40.8	-2.9	Moderate increase in IrCN3 dimer

IrCN3(M23Y4)	47.1	39.9	-7.2	Slight increase in IrCN3 dimer

6.Biological testing

The following Gram positive bacterial strains were used for testing:

Enterococcus faecalis CCUG 19916T

Enterococcus faecium CCUG 19434

Staphylococcus aureus CCUG 62707

Bacillus subtilis 168CA

Bacterial stock solutions were prepared as follows. Single bacterial colonies were grown on Tryptic Soy Broth (TSB) or Miller's LB Broth (LB, *B. subtilis*) agar. A single colony was removed and placed in the relevant growth medium (5 mL TSB in a 15 mL falcon tube, or 2 mL LB in a 50 mL falcon tube) and incubated overnight at 37 °C (200 rpm). 100 µL of this overnight culture was added to 5 mL of the same growth medium, and the day culture was incubated at 37 °C (200 rpm) until the OD600 was 0.4 – 0.9 (approx. 4 h). A sample of this culture was diluted with Mueller Hinton (MH) broth to OD600 = 0.022, at which point the bacterial cultures were added to plates for testing.

For all testing, samples were incubated with bacteria for 16 – 20 h at 37 °C, after which the absorbance at 600 nm was measured using a plate reader.

The antibiotic control was vancomycin (stock solution at 1 mg / mL in water, MIC beginning at 20 µg / mL) for all bacteria.

Unless stated otherwise, 2 biological repeats per sample were recorded, with each having at least 2 technical repeats. Polystyrene 96 well plates from TPP and CytoOne were used in all cases (sterile, DNA, DNase, RNase, pyrogen free). DMSO and other solvents were kept below 5% volume in the final solutions for biological testing.

6.1 Minimum Inhibitory Concentration (MIC)

Stock solutions of samples were diluted with DMSO so that 6 uL of stock solution can be added to 300 µL of MH growth media to give an initial concentration of 100 µM. These solutions were serially diluted (150 µL into 150 µL) 7 times (crude compounds) or 11 times (pure compounds) to give solutions of 150 µL. To these, 5 µL of the relevant bacterial strain was added in MH growth media. The plates were incubated for 16 – 20 h at 37 °C, after which the absorbance at 600 nm was measured using a plate reader.

Data for the median MICs of purified compounds are shown below (in the tables, a "N/A" means not active at any of the tested concentrations). For automatic processing of MIC values, if there is an equal number of values at 2 different concentrations, the lower was picked as the MIC. Photoirradiation was achieved using an array of 96 405 nm LEDs positioned 2 cm above the plate for 12 min after addition of bacteria.

Table S3: MIC for IrCN-Schiff crudes against *S. aureus* CCUG 62707 (MH growth media)

	S. aureus (biological repeat 1) MIC (µM)			*S. aureus* (biological repeat 2) MIC (µM)			
	Statistical repeat			Statistical repeat			
**Crude**	1	2	3	1	2	3	**MIC**
**IrCN1_P4A11**	3.125	3.125	3.125	6.25	3.125	3.125	**3.125**
**IrCN2_P3A6**	25	12.5	12.5	25	25	12.5	**12.5**
**IrCN3_P4A11**	12.5	12.5	12.5	12.5	12.5	12.5	**12.5**
**IrCN1_P4A11**	0.78	0.78	0.78	0.78	0.78	0.78	**0.78**
**IrCN2_P3A11**	100	100	100	100	100	N/A	**100**
**IrCN3_P6A11**	25	25	25	25	25	25	**25**
**IrCN1_P4A5**	0.78	0.78	0.78	0.78	0.78	1.56	**0.78**
**IrCN2_P3A11**	12.5	12.5	25	25	12.5	12.5	**12.5**
**IrCN3_P6A11**	6.25	12.5	12.5	6.25	6.25	6.25	**6.25**
**IrCN1_P4A11**	3.125	0.78	3.125	1.56	3.125	3.125	**3.125**
**IrCN2_P3A11**	100	50	50	50	50	50	**50**
**IrCN3_P6A11**	3.125	3.125	3.125	3.125	3.125	3.125	**3.125**
**IrCN1_P5A9**	50	50	50	50	50	50	**50**
**IrCN2_P5A11**	50	12.5	12.5	25	25	12.5	**25**
**IrCN3_P6A7**	12.5	25	12.5	12.5	12.5	12.5	**12.5**
**IrCN1_P6A11**	12.5	12.5	12.5	12.5	12.5	12.5	**12.5**
**IrCN2_P5A11**	100	50	N/A	50	25	25	**50**
**IrCN3_P6A8**	6.25	12.5	6.25	6.25	6.25	12.5	**6.25**
**IrCN1_P6A11**	25	6.25	25	12.5	25	25	**25**
**IrCN2_P6A9**	3.125	1.56	1.56	1.56	1.56	3.125	**1.56**
**IrCN3_P6A10**	3.125	6.25	3.125	6.25	3.125	12.5	**3.125**
**IrCN1_P7A11**	12.5	6.25	12.5	12.5	12.5	12.5	**12.5**
**IrCN2_P5A11**	25	25	25	25	25	25	**25**
**IrCN3_P7A11**	100	100	100	N/A	100	N/A	**100**
**IrCN1_P8A9**	1.56	1.56	1.56	1.56	1.56	3.125	**1.56**
**IrCN2_P6A11**	6.25	1.56	1.56	1.56	3.125	3.125	**3.125**
**IrCN3_P7A11**	50	100	50	100	50	100	**50**
**IrCN1_P8A11**	3.125	1.56	3.125	3.125	3.125	3.125	**3.125**
**IrCN2_P8A5**	25	25	25	25	25	25	**25**
**IrCN3_P7A11**	6.25	12.5	6.25	6.25	6.25	6.25	**6.25**
**IrCN1_P1A11**	12.5	12.5	12.5	12.5	12.5	12.5	**12.5**
**IrCN2_P1A11**	1.56	1.56	1.56	1.56	1.56	1.56	**1.56**
**IrCN3_P1A9**	25	25	25	25	25	25	**25**
**IrCN1_P1A11**	N/A	N/A	N/A	N/A	N/A	N/A	**N/A**
**IrCN2_P1A11**	1.56	3.125	3.125	3.125	3.125	1.56	**3.125**
**IrCN3_P1A11**	6.25	6.25	6.25	12.5	6.25	6.25	**6.25**
**IrCN1_P8A8**	6.25	1.56	1.56	1.56	1.56	1.56	**1.56**
**IrCN2_P8A10**	25	12.5	12.5	12.5	12.5	12.5	**12.5**

**IrCN3_P8A9**	12.5	12.5	12.5	12.5	6.25	12.5	**12.5**
**IrCN1_P1A11**	0.78	0.78	0.78	0.78	0.78	0.78	**0.78**
**IrCN1_P8A11**	12.5	12.5	12.5	12.5	12.5	12.5	**12.5**
**IrCN3_P1A11**	1.56	1.56	0.78	3.125	0.78	0.78	**1.56**
**IrCN1_P1A3**	1.56	1.56	1.56	1.56	1.56	1.56	**1.56**
**IrCN2_P1A11**	12.5	12.5	12.5	12.5	12.5	12.5	**12.5**
**IrCN2_P8A11**	3.125	3.125	3.125	3.125	3.125	3.125	**3.125**
**IrCN1_P1A5**	0.78	0.78	0.78	0.78	0.78	0.78	**0.78**
**IrCN2_P2A11**	3.125	6.25	3.125	6.25	6.25	3.125	**3.125**
**IrCN3_P1A8**	1.56	1.56	1.56	3.125	1.56	1.56	**1.56**
**IrCN1_P1A10**	1.56	1.56	1.56	3.125	1.56	1.56	**1.56**
**IrCN2_P2A5**	6.25	6.25	6.25	6.25	6.25	3.125	**6.25**
**IrCN3_P4A9**	12.5	12.5	12.5	25	12.5	12.5	**12.5**
**IrCN1_P1A11**	1.56	3.125	3.125	3.125	1.56	1.56	**1.56**
**IrCN2_P2A11**	50	25	50	50	50	25	**50**
**IrCN3_P4A11**	25	12.5	12.5	12.5	12.5	12.5	**12.5**
**IrCN1_P2A8**	1.56	3.125	3.125	3.125	3.125	3.125	**3.125**
**IrCN2_P3A9**	100	50	N/A	100	100	50	**100**
**IrCN3_P4A5**	50	50	50	50	50	50	**50**
**IrCN1_P4A11**	3.125	3.125	3.125	3.125	3.125	3.125	**3.125**
**IrCN2_P3A11**	6.25	12.5	12.5	12.5	6.25	6.25	**6.25**
**IrCN3_P5A11**	N/A	N/A	N/A	N/A	N/A	N/A	**N/A**

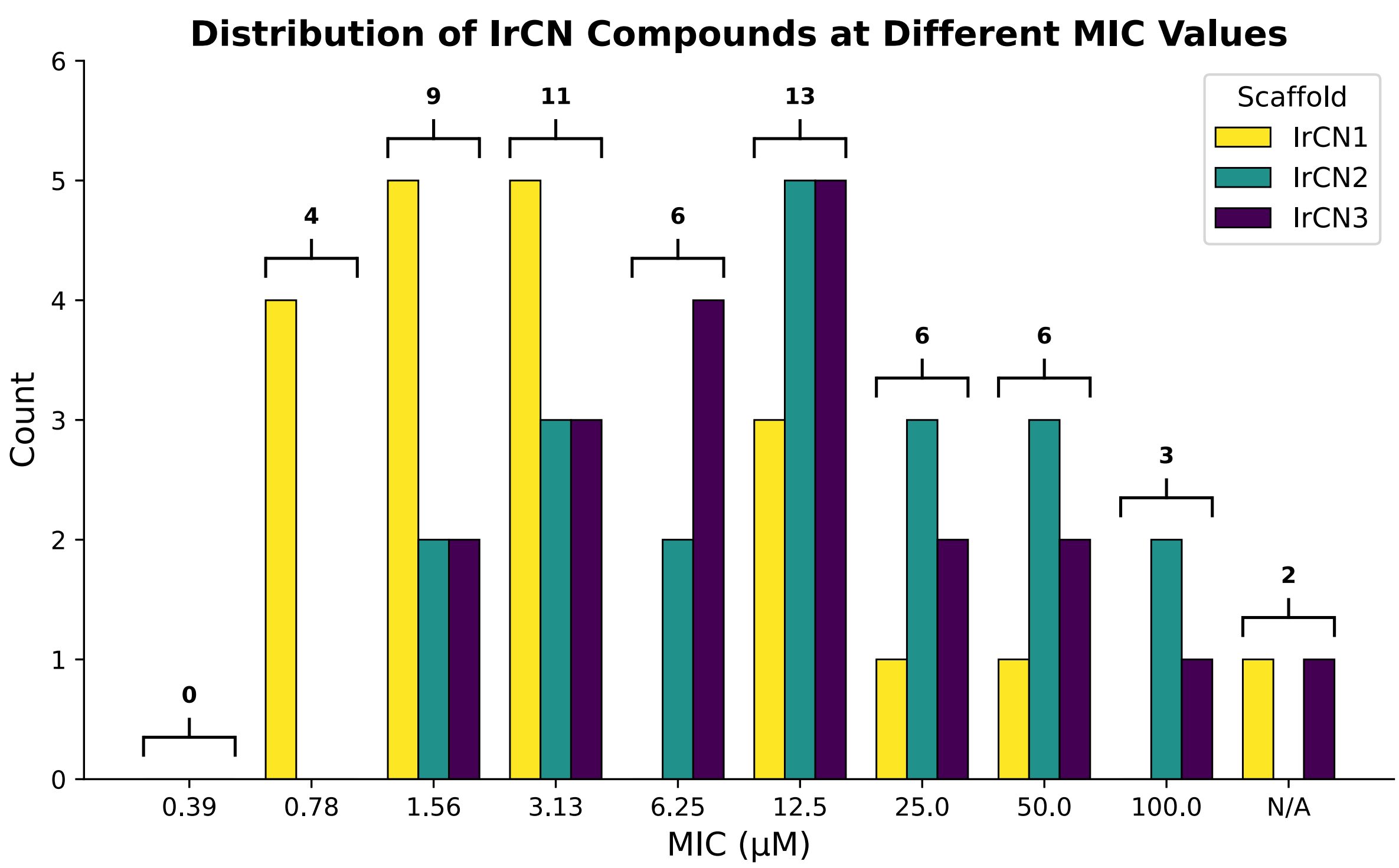

Figure S51: Distribution of MICs for IrCN-SB libraries against *S. aureus*, broken down by IrCN scaffold

Table S4: MIC for IrCN-Click crudes against *S. aureus* CCUG 62707 (MH growth media)

	***S. aureus* (biological repeat 1) MIC (µM)**		***S. aureus* (biological repeat 2) MIC (µM)**		
	**Statistical repeat**		**Statistical repeat**		
**Crude**	1	2	1	2	**MIC**
**IrCN1_M11Y3**	0.78	0.78	1.56	1.56	**0.78**
**IrCN2_M12Y3**	25	25	25	25	**25**
**IrCN3_M23Y2**	50	N/A	50	50	**50**
**IrCN1_M15Y3**	50	50	50	50	**50**
**IrCN2_M14Y3**	50	25	25	50	**25**
**IrCN3_M11Y3**	12.5	12.5	12.5	12.5	**12.5**
**IrCN1_M16Y3**	3.125	3.125	1.56	1.56	**1.56**
**IrCN2_M16Y3**	0.78	1.56	1.56	1.56	**1.56**
**IrCN3_M12Y3**	3.125	3.125	6.25	6.25	**3.125**
**IrCN1_M2Y4**	6.25	6.25	6.25	6.25	**6.25**
**IrCN2_M18Y3**	3.125	6.25	6.25	6.25	**6.25**
**IrCN3_M14Y3**	25	50	25	50	**25**
**IrCN1_M6Y4**	0.39	0.78	0.78	0.78	**0.78**
**IrCN2_M10Y4**	25	25	25	25	**25**
**IrCN3_M23Y3**	50	50	50	50	**50**
**IrCN1_M8Y4**	0.78	0.78	0.78	0.78	**0.78**
**IrCN2_M13Y4**	25	N/A	N/A	N/A	**N/A**
**IrCN3_M10Y4**	50	6.25	6.25	6.25	**6.25**
**IrCN1_M9Y4**	0.78	1.56	1.56	1.56	**1.56**
**IrCN2_M16Y4**	1.56	1.56	3.125	3.125	**1.56**
**IrCN3_M11Y4**	50	50	50	50	**50**
**IrCN1_M10Y4**	0.78	0.78	0.78	1.56	**0.78**
**IrCN2_M17Y4**	6.25	12.5	12.5	25	**12.5**
**IrCN3_M18Y4**	6.25	6.25	6.25	6.25	**6.25**
**IrCN1_M16Y4**	1.56	3.125	3.125	3.125	**3.125**
**IrCN2_M18Y4**	3.125	6.25	6.25	6.25	**6.25**
**IrCN3_M19Y4**	25	3.125	6.25	6.25	**6.25**
**IrCN1_M17Y4**	0.78	0.78	0.78	0.78	**0.78**
**IrCN2_M20Y4**	12.5	25	25	25	**25**

**IrCN3_M22Y4**	6.25	12.5	12.5	12.5	**12.5**
**IrCN1_M2Y1**	25	25	25	25	**25**
**IrCN2_M9Y1**	50	12.5	25	25	**25**
**IrCN3_M2Y1**	3.125	3.125	6.25	3.125	**3.125**
**IrCN1_M7Y1**	1.56	1.56	1.56	1.56	**1.56**
**IrCN2_M11Y1**	6.25	6.25	6.25	12.5	**6.25**
**IrCN3_M11Y1**	6.25	6.25	6.25	6.25	**6.25**
**IrCN1_M20Y4**	0.78	0.78	0.78	0.78	**0.78**
**IrCN2_M23Y4**	25	25	50	50	**25**
**IrCN3_M23Y4**	6.25	50	50	50	**50**
**IrCN1_M8Y1**	0.39	0.39	0.39	0.39	**0.39**
**IrCN2_M14Y1**	50	25	25	25	**25**
**IrCN3_M12Y1**	3.125	3.125	3.125	1.56	**3.125**
**IrCN1_M20Y1**	0.78	0.78	0.78	0.78	**0.78**
**IrCN2_M16Y1**	6.25	6.25	6.25	6.25	**6.25**
**IrCN3_M13Y1**	6.25	6.25	6.25	6.25	**6.25**
**IrCN1_M2Y2**	12.5	12.5	12.5	12.5	**12.5**
**IrCN2_M18Y1**	3.125	3.125	3.125	6.25	**3.125**
**IrCN3_M23Y1**	25	50	50	25	**25**
**IrCN1_M8Y2**	0.39	0.39	0.39	0.39	**0.39**
**IrCN2_M2Y2**	1.56	1.56	1.56	1.56	**1.56**
**IrCN3_M10Y2**	12.5	12.5	12.5	6.25	**12.5**
**IrCN1_M16Y2**	0.78	0.78	0.78	0.78	**0.78**
**IrCN2_M7Y2**	1.56	1.56	1.56	1.56	**1.56**
**IrCN3_M14Y2**	50	50	50	50	**50**
**IrCN1_M17Y2**	0.39	0.39	0.39	0.39	**0.39**
**IrCN2_M23Y2**	N/A	N/A	N/A	N/A	**N/A**
**IrCN3_M20Y2**	12.5	12.5	12.5	12.5	**12.5**
**IrCN1_M2Y3**	12.5	12.5	12.5	12.5	**12.5**
**IrCN2_M11Y3**	12.5	12.5	12.5	12.5	**12.5**
**IrCN3_M22Y2**	25	50	12.5	25	**25**

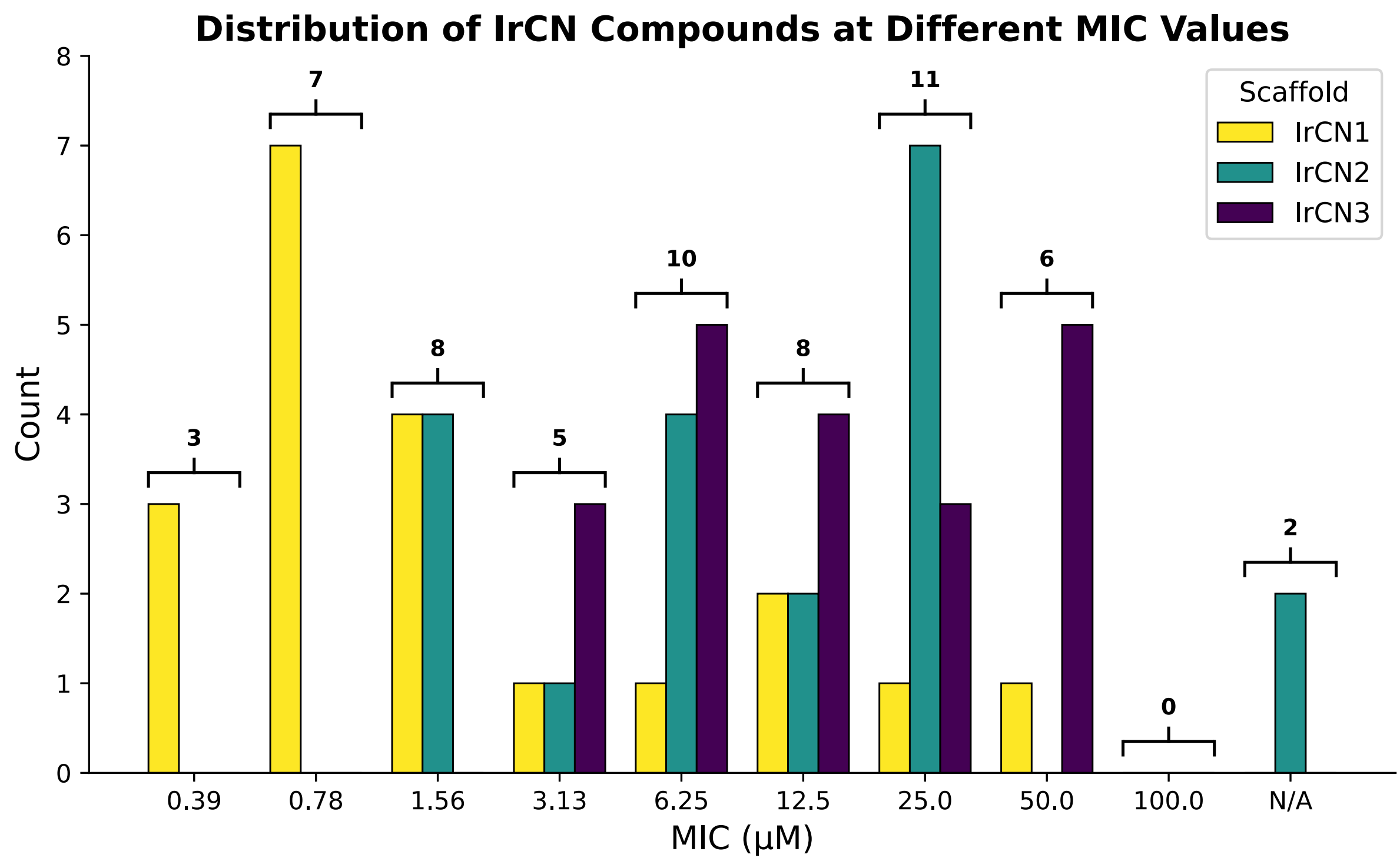

Figure S52: Distribution of MICs for IrCN-Click libraries against *S. aureus*, broken down by IrCN scaffold

Table S5: MIC for purified compounds against *S. aureus* CCUG 62707 (MH growth media)

	S. aureus **(biological repeat 1) MIC (µM)**			***S. aureus*** **(biological repeat 2) MIC (µM)**			
	**Statistical repeat**			**Statistical repeat**			
**Compound**	1	2	3	1	2	3	**MIC**
**IrCN1(P1A3)**	3.13	1.56	1.56	1.56	1.56	1.56	1.56
**IrCN1(P8A1)**	3.13	3.13	3.13	3.13	3.13	3.13	3.13
**IrCN2(P3A2)**	3.13	3.13	3.13	6.25	3.13	3.13	3.13
**IrCN2(P6A9)**	3.13	1.56	1.56	1.56	3.13	1.56	1.56
**IrCN3(P6A1)**	1.56	3.13	3.13	3.13	3.13	3.13	3.13
**IrCN3(M2Y1)**	3.13	3.13	6.25	3.13	3.13	3.13	3.13
**IrCN3(M12Y3)**	3.13	3.13	3.13	3.13	3.13	1.56	3.13
**IrCN2(M18Y4)-PF_6**	12.5	12.5	12.5	25	25	25	12.5
**VCM**	1.73	1.73	1.73	1.73	1.73	1.73	1.73

Table S6: MIC for purified compounds against *S. aureus* CCUG 62707 with 12 min of photoirradiation at 405 nm (MH growth media)

	S. aureus **(biological repeat 1) MIC (μM)**			***S. aureus*** **(biological repeat 2) MIC (μM)**			
	**Statistical repeat**			**Statistical repeat**			
**Compound**	1	2	3	1	2	3	**MIC**
**IrCN1(P1A3)**	0.78	0.78	0.78	0.78	0.78	0.78	0.78
**IrCN1(P8A1)**	1.56	3.13	1.56	1.56	1.56	1.56	1.56
**IrCN2(P3A2)**	1.56	1.56	1.56	1.56	1.56	3.13	3.13
**IrCN2(P6A9)**	1.56	1.56	0.78	1.56	1.56	0.78	1,56
**IrCN3(P6A1)**	1.56	3.13	1.56	1.56	1.56	1.56	1.56
**IrCN3(M2Y1)**	3.13	6.25	3.13	1.56	3.13	3.13	3.13
**IrCN3(M12Y3)**	1.56	1.56	1.56	1.56	3.13	1.56	3.13
**IrCN2(M18Y4)-PF_6**	3.13	3.13	3.13	6.25	6.25	6.25	3.13
**VCM**	1.73	1.73	1.73	1.73	1.73	1.73	1.73

Table S7: MIC for purified compounds against *E. faecium CCUG 19434* (TSB growth media)

	E. faecium **(biological repeat 1) MIC (μM)**			***E. faecium*** **(biological repeat 2) MIC (μM)**			
	**Statistical repeat**			**Statistical repeat**			
**Compound**	1	2	3	1	2	3	**MIC**
**IrCN1(P1A3)**	3.13	3.13	6.25	3.13	6.25	6.25	3.13
**IrCN1(P8A1)**	6.25	6.25	6.25	6.25	6.25	6.25	6.25
**IrCN2(P3A2)**	6.25	6.25	6.25	6.25	6.25	6.25	6.25
**IrCN2(P6A9)**	3.13	3.13	3.13	3.13	6.25	3.13	3.13
**IrCN3(P6A1)**	6.25	6.25	6.25	6.25	6.25	6.25	6.25
**IrCN3(M2Y1)**	25	25	25	25	25	25	25

**IrCN3(M12Y3)**	3.13	3.13	3.13	3.13	3.13	3.13	3.13
**VCM**	1.73	1.73	1.73	1.73	1.73	1.73	1.73

Table S8: MIC for purified compounds against *E. faecalis CCUG 19916T* (MH growth media)

	***E. faecalis* (biological repeat 1) MIC (µM)**			***E. faecalis* (biological repeat 2) MIC (µM)**			
	**Statistical repeat**			**Statistical repeat**			
**Compound**	1	2	3	1	2	3	**MIC**
**IrCN1(P1A3)**	1.56	1.56	1.56	1.56	3.13	3.13	1.56
**IrCN1(P8A1)**	3.13	3.13	6.25	6.25	6.25	3.13	3.13
**IrCN2(P3A2)**	12.5	6.25	6.25	12.5	12.5	6.25	6.
**IrCN2(P6A9)**	1.56	1.56	1.56	1.56	3.13	3.13	1.56
**IrCN3(P6A1)**	3.13	3.13	3.13	3.13	3.13	3.13	3.13
**IrCN3(M2Y1)**	12.5	12.5	12.5	12.5	12.5	12.5	12.5
**IrCN3(M12Y3)**	3.13	3.13	3.13	6.25	3.13	3.13	3.13
**VCM**	0.86	0.86	0.86	0.86	0.86	0.43	0.86

Table S9: MIC for purified compounds against *B. subtilis 168CA* (MH growth media)

	***B. subtilis* (biological repeat 1) MIC (µM)**			***B. subtilis* (biological repeat 2) MIC (µM)**			
	**Statistical repeat**			**Statistical repeat**			
**Compound**	1	2	3	1	2	3	**MIC**
**IrCN1(P1A3)**	1.56	1.56	1.56	1.56	1.56	1.56	1.56
**IrCN1(P8A1)**	6.25	6.25	3.13	3.13	6.25	3.13	3.13
**IrCN2(P3A2)**	3.13	3.13	3.13	3.13	3.13	3.13	3.13
**IrCN2(P6A9)**	3.13	3.13	3.13	3.13	6.25	3.13	3.13
**IrCN3(P6A1)**	3.13	3.13	3.13	3.13	3.13	3.13	3.13

**IrCN3(M2Y1)**	3.13	3.13	3.13	3.13	6.25	3.13	3.13
**IrCN3(M12Y3)**	6.25	3.13	3.13	3.13	3.13	3.13	3.13
**VCM**	0.43	0.86	0.43	0.43	0.86	0.86	0.43

6.2 Toxicity Testing

Cell culture

The human embryonic kidney (HEK) 293T cell line was cultured in Dulbecco's Modified Eagle Medium high glucose (DMEM) (Sigma Aldrich, USA) supplemented with 10% heat-inactivated fetal bovine serum (FBS), 100 µg/mL streptomycin and 100 Units/mL penicillin, and 2 mM L-glutamine. The cells were maintained in a 5% CO_2 environment, at 37 °C and the culture media were replaced with fresh media every 72 hours.

Determination of the cytotoxic concentration 50% (CC_{50}) – MTT assay

HEK-293T cells were seeded at 10000 cells per well and incubated overnight to allow adhesion. 5 mM stock solutions of pure compounds were prepared in DMSO. In a 1.5 mL 96 well dilution plate, starting concentrations of 150 µM and 200 µM of each compound were made in growth media (1 mL). 500 uL of these were serial diluted into 500 uL of media 11 times. 500 uL of media was then added to each well to make 1 mL of each concentration (starting at 75 µM and 100 µM). 100 uL of these solutions were added into wells containing the pre-counted cells and incubated for 24 h. To quantify cell viability, the cells were treated with the 3-(4,5-dimethylthiazol-2-yl)-2,5 diphenyltetrazolium bromide (MTT) salt (Apollo Scientific, UK) according to the procedure described by Mosmann.[5] The absorbance at 595 nm was measured using a TECAN well plate reader instrument M1000. For each compound, 4 biological repeats in triplicate were done for this study (12 data points) from which the CC_{50} was determined using custom Python scripts. The fit of the line was fixed between 0% and 100% viability, and this value is reported as the CC_{50}.

Table S10: Calculated CC_{50} values for pure compounds with the associated standard deviation (SD)

	**Toxicity (µM)**	
**Compound**	**CC_{50}**	**SD**
IrCN1(P1A3)	3.30	0.11
IrCN1(P8A1)	3.61	0.10
IrCN2(P6A9)	7.75	0.20
IrCN3(P6A1)	13.81	0.39

IrCN3(M2Y1)	13.83	0.33
IrCN3(M12Y3)	2.72	0.10

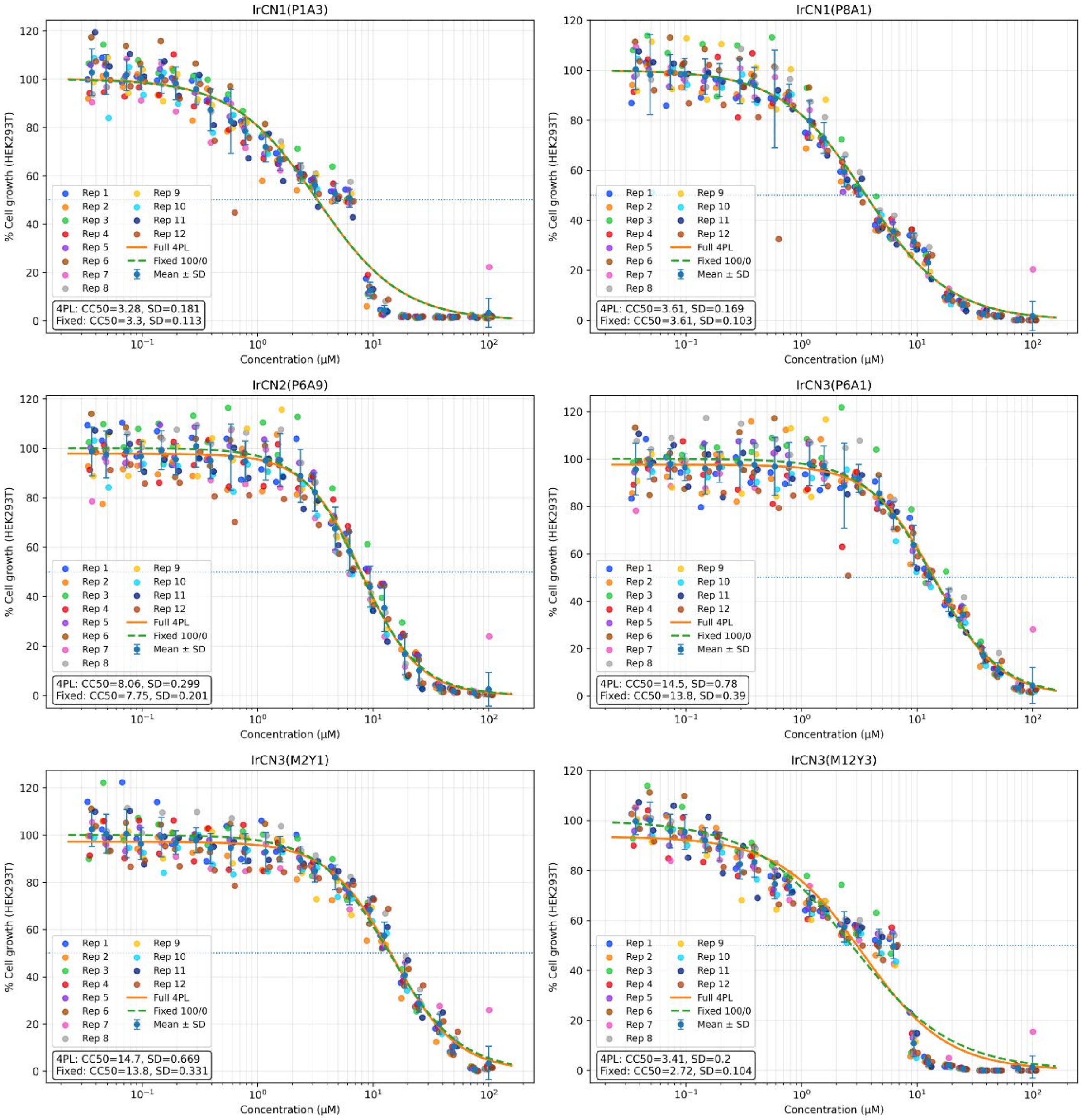

Figure S53: CC_{50} dose response plots for purified compounds (HEK293T cells)

7. HRMS data of pure compounds

York - Chemistry - Mass Spectrometry Service Report

MGD-013-IrCN1All_af120912md

Analysis Information

Analysis Filename	af120912md_P1-A-8_01_59372.d	Acquisition Date	24/07/2025 12:32:26
Method	ESI_low mass_2c1s.m	Instrument	compact
Submission Name	af120912md	ESI	Positive

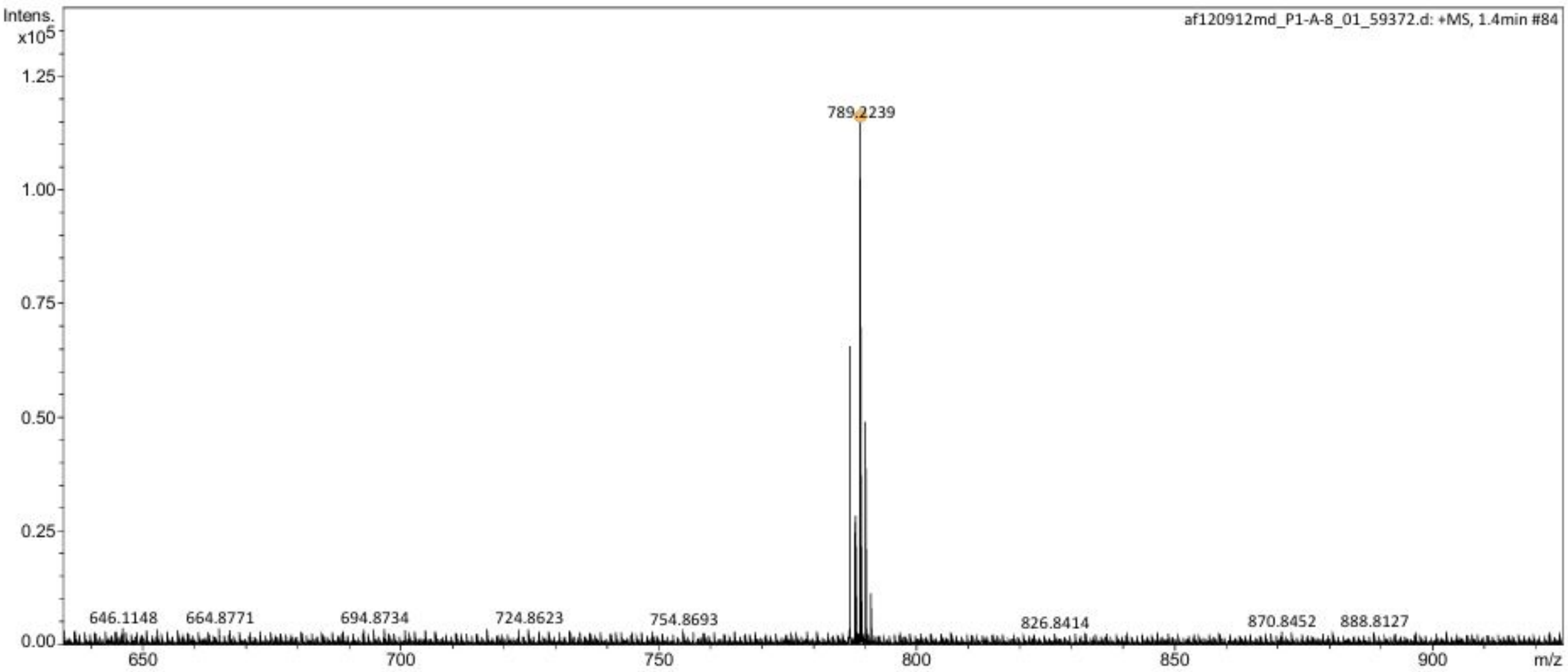

Meas. m/z	#	Ion Formula	m/z	err [ppm]	err [mDa]	mSigma	Mean err [ppm]
789.2239	1	C41H32IrN4O	789.2200	-4.7	-3.7	10.0	-4.5

Figure S54: HRMS (ESI positive mode) of IrCN1(P1A3)

York - Chemistry - Mass Spectrometry Service Report

MGD-013-IrCN1P6_af120913md

Analysis Information

Analysis Filename	af120913md_P1-A-9_01_59373.d	Acquisition Date	24/07/2025 12:35:23
Method	ESI_low mass_2c1s.m	Instrument	compact
Submission Name	af120913md	ESI	Positive

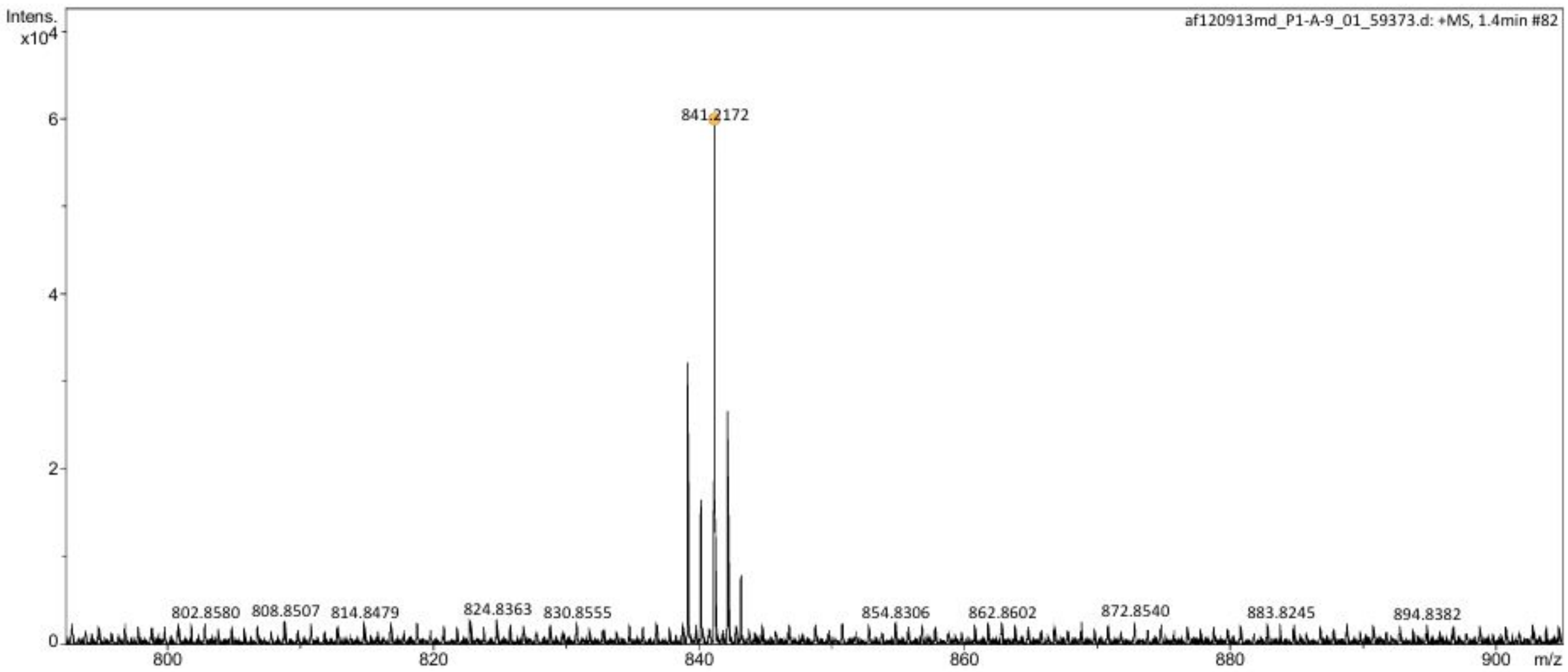

Meas. m/z	#	Ion Formula	m/z	err [ppm]	err [mDa]	mSigma	Mean err [ppm]
841.2172	1	C44H32IrN4O2	841.2149	-2.4	-2.0	13.0	-1.7

Figure S55: HRMS (ESI positive mode) of IrCN1(P8A1)

York - Chemistry - Mass Spectrometry Service Report

MGD-014-IrCN2D7_af120914md

Analysis Information

Analysis Filename	af120914md_P1-B-1_01_59374.d	Acquisition Date	24/07/2025 12:38:22
Method	ESI_low mass_2c1s.m	Instrument	compact
Submission Name	af120914md	ESI	Positive

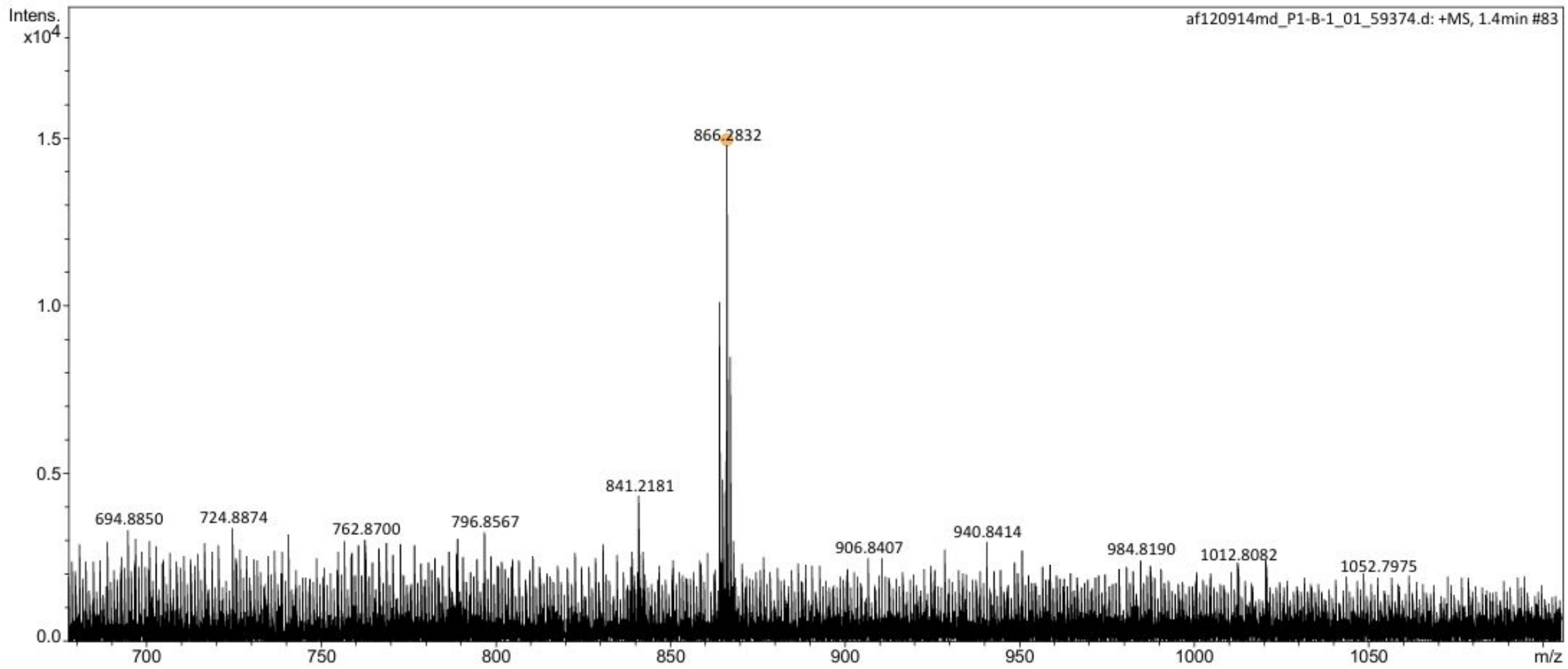

Meas. m/z	#	Ion Formula	m/z	err [ppm]	err [mDa]	mSigma	Mean err [ppm]
866.2832	1	C47H39IrN5	866.2829	0.0	0.0	71.2	-2.7

Figure S56: HRMS (ESI positive mode) of IrCN2(P3A2)

York - Chemistry - Mass Spectrometry Service Report

MGD-014-IrCN2J3_af120915md

Analysis Information

Analysis Filename	af120915md_P1-B-2_01_59375.d	Acquisition Date	24/07/2025 12:41:21
Method	ESI_low mass_2c1s.m	Instrument	compact
Submission Name	af120915md	ESI	Positive

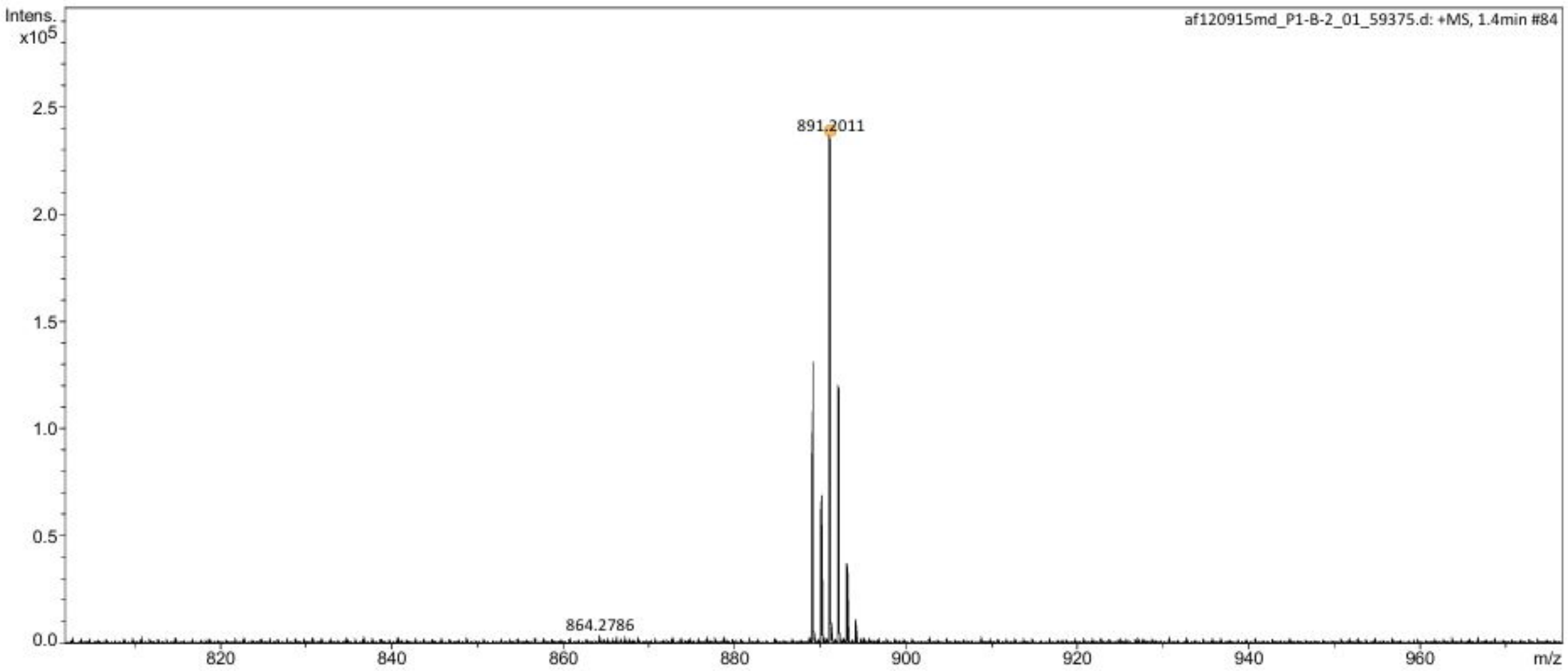

Meas. m/z	#	Ion Formula	m/z	err [ppm]	err [mDa]	mSigma	Mean err [ppm]
891.2011	1	C44H34IrN4O3S	891.1975	-3.9	-3.5	16.2	-4.1

Figure S57: HRMS (ESI positive mode) of IrCN2(P6A9)

York - Chemistry - Mass Spectrometry Service Report

MGD-014-IrCN3J6_af120916md

Analysis Information

Analysis Filename	af120916md_P1-B-3_01_59376.d	Acquisition Date	24/07/2025 12:44:21
Method	ESI_low mass_2c1s.m	Instrument	compact
Submission Name	af120916md	ESI	Positive

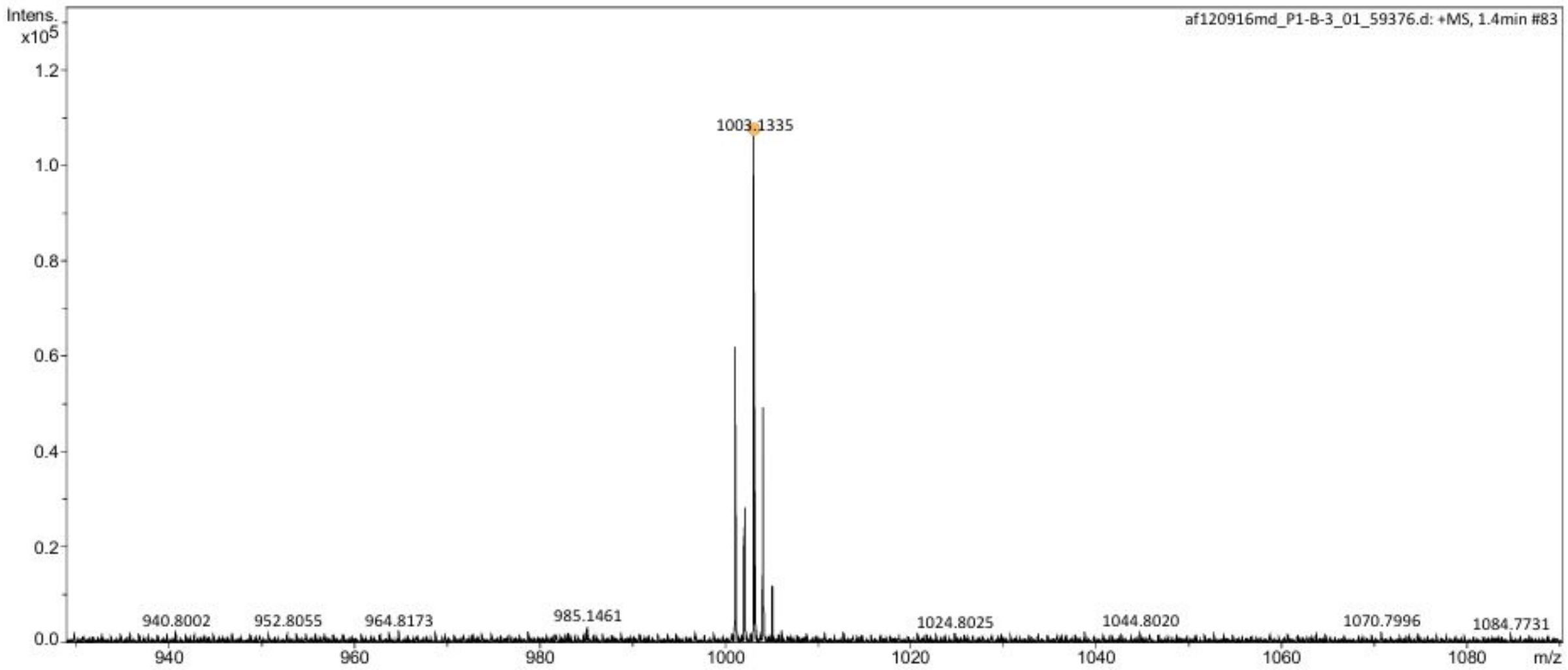

Meas. m/z	#	Ion Formula	m/z	err [ppm]	err [mDa]	mSigma	Mean err [ppm]
1003.1335	1	C41H24F10IrN4O3	1003.1312	-2.0	-2.0	14.1	-1.5

Figure S58: HRMS (ESI positive mode) of IrCN3(P6A1)

York - Chemistry - Mass Spectrometry Service Report

DRH-097-1_af121970dh

Analysis Information

Analysis Filename	af121970dh_P1-C-2_01_60499.d	Acquisition Date	06/10/2025 15:15:27
Method	ESI_low mass_2c1s.m	Instrument	compact
Submission Name	af121970dh	ESI	Positive

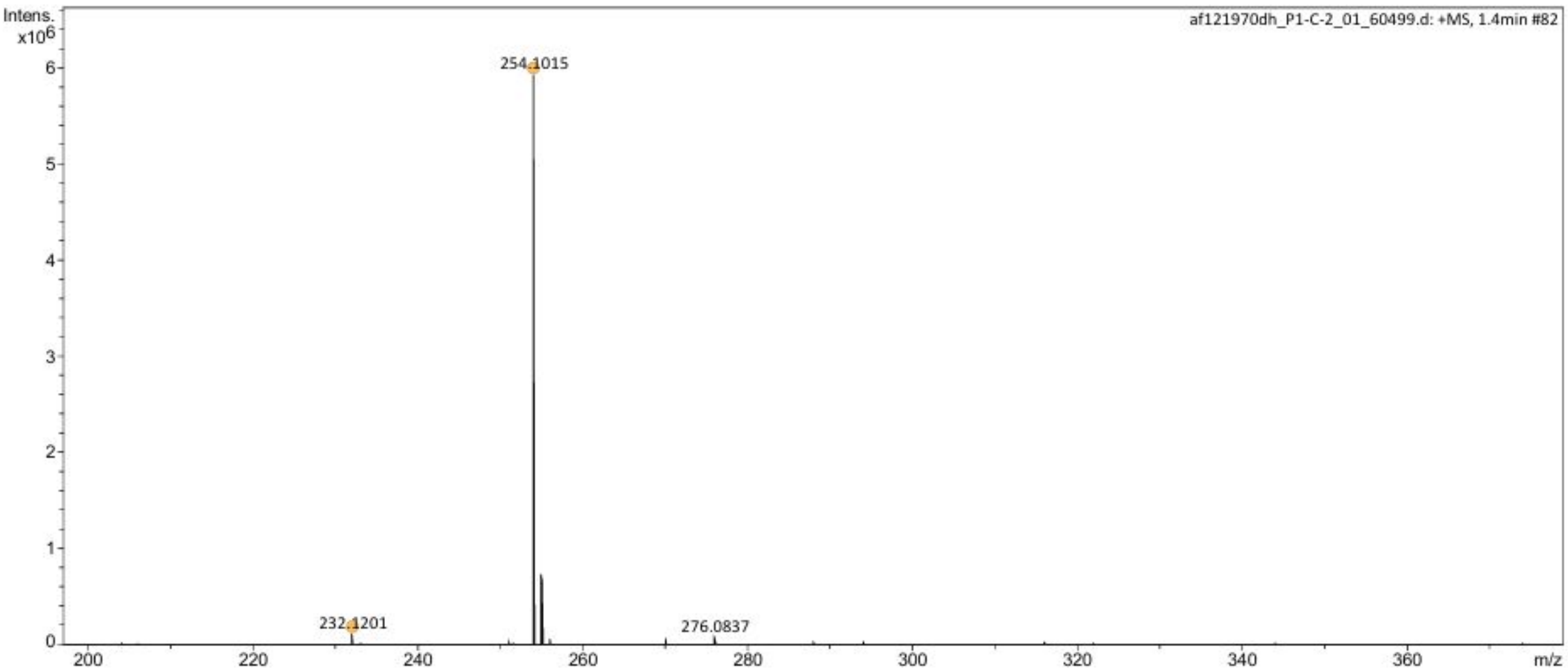

Meas. m/z	#	Ion Formula	m/z	err [ppm]	err [mDa]	mSigma	Mean err [ppm]
232.1201	1	C11H14N5O	232.1193	-3.7	-0.8	9.8	-2.1
254.1015	1	C11H13N5NaO	254.1012	-1.1	-0.3	9.0	-2.0

Figure S59: HRMS (ESI positive mode) of M2Y1

York - Chemistry - Mass Spectrometry Service Report

DRH-097-2_af122202dh

Analysis Information

Analysis Filename	af122202dh_P1-D-5_01_60767.d	Acquisition Date	17/10/2025 12:46:41
Method	ESI_low mass_2c1s.m	Instrument	compact
Submission Name	af122202dh	ESI	Positive

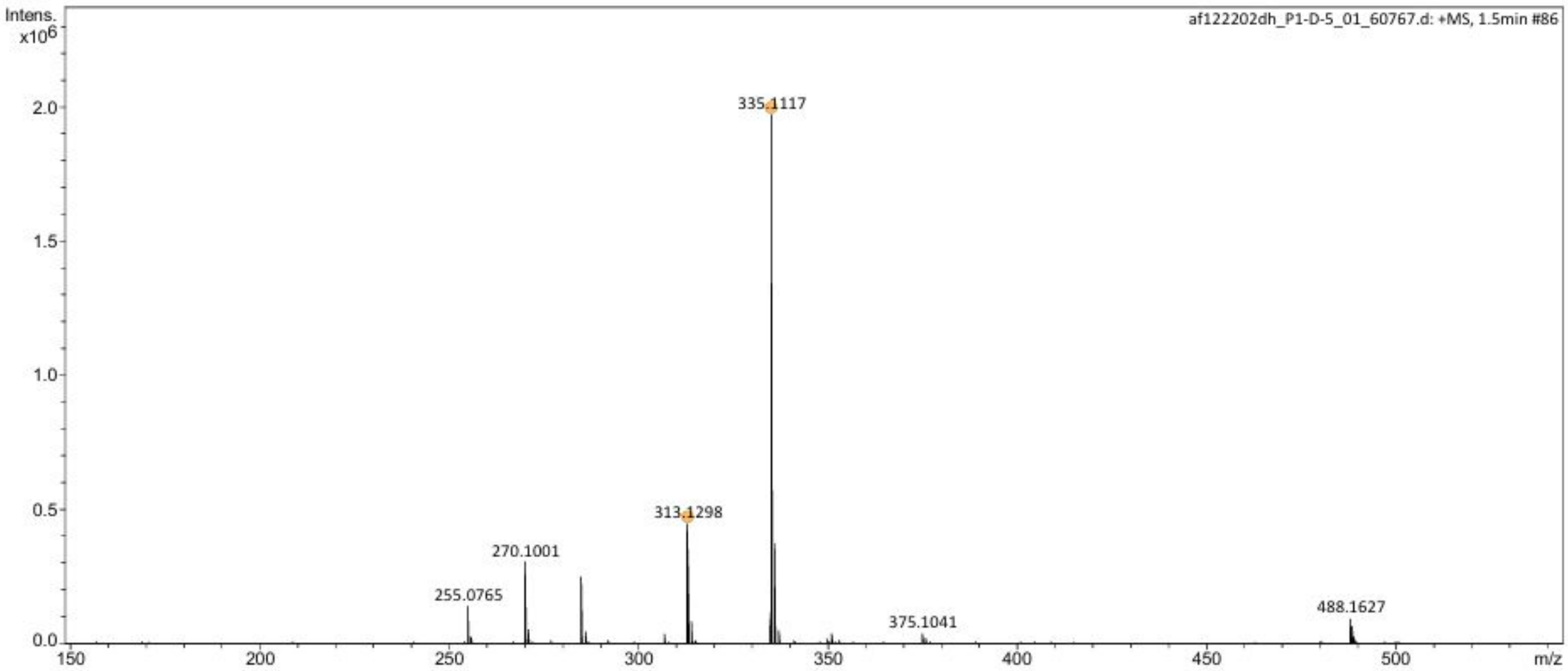

Meas. m/z	#	Ion Formula	m/z	err [ppm]	err [mDa]	mSigma	Mean err [ppm]
313.1298	1	C16H17N4O3	313.1295	-0.8	-0.3	1.0	-1.4
335.1117	1	C16H16N4NaO3	335.1115	-0.6	-0.2	1.0	-1.2

Figure S60: HRMS (ESI positive mode) of M12Y3

York - Chemistry - Mass Spectrometry Service Report

DRH-097-3_af122203dh

Analysis Information

Analysis Filename	af122203dh_P1-D-6_01_60768.d	Acquisition Date	17/10/2025 12:49:39
Method	ESI_low mass_2c1s.m	Instrument	compact
Submission Name	af122203dh	ESI	Positive

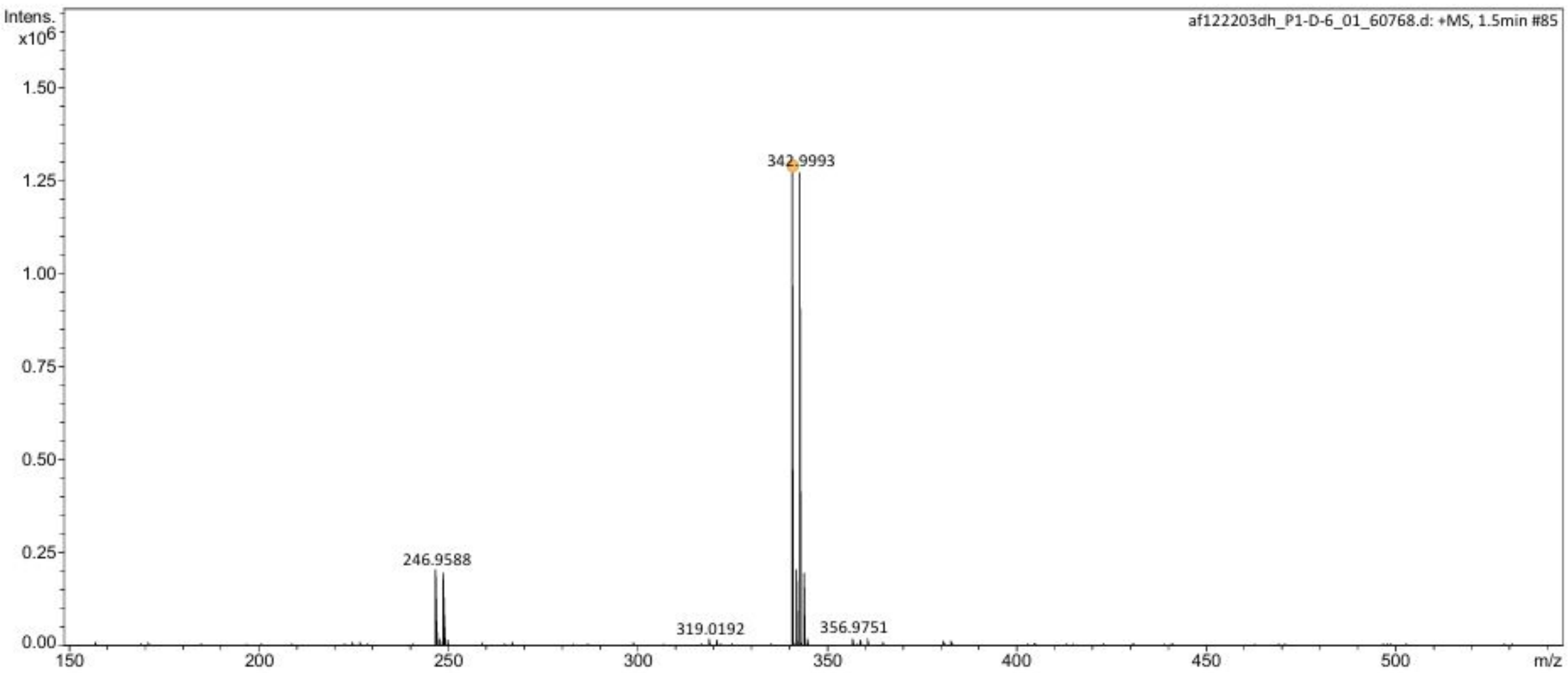

Meas. m/z	#	Ion Formula	m/z	err [ppm]	err [mDa]	mSigma	Mean err [ppm]
341.0009	1	C13H11BrN4NaO	341.0008	-0.2	-0.1	6.5	-1.0

Figure S61: HRMS (ESI positive mode) of M18Y4

York - Chemistry - Mass Spectrometry Service Report

DRH-098-1_af122198dh

Analysis Information

Analysis Filename	af122198dh_P1-D-1_01_60763.d	Acquisition Date	17/10/2025 11:03:53
Method	ESI_low mass_2c1s.m	Instrument	compact
Submission Name	af122198dh	ESI	Positive

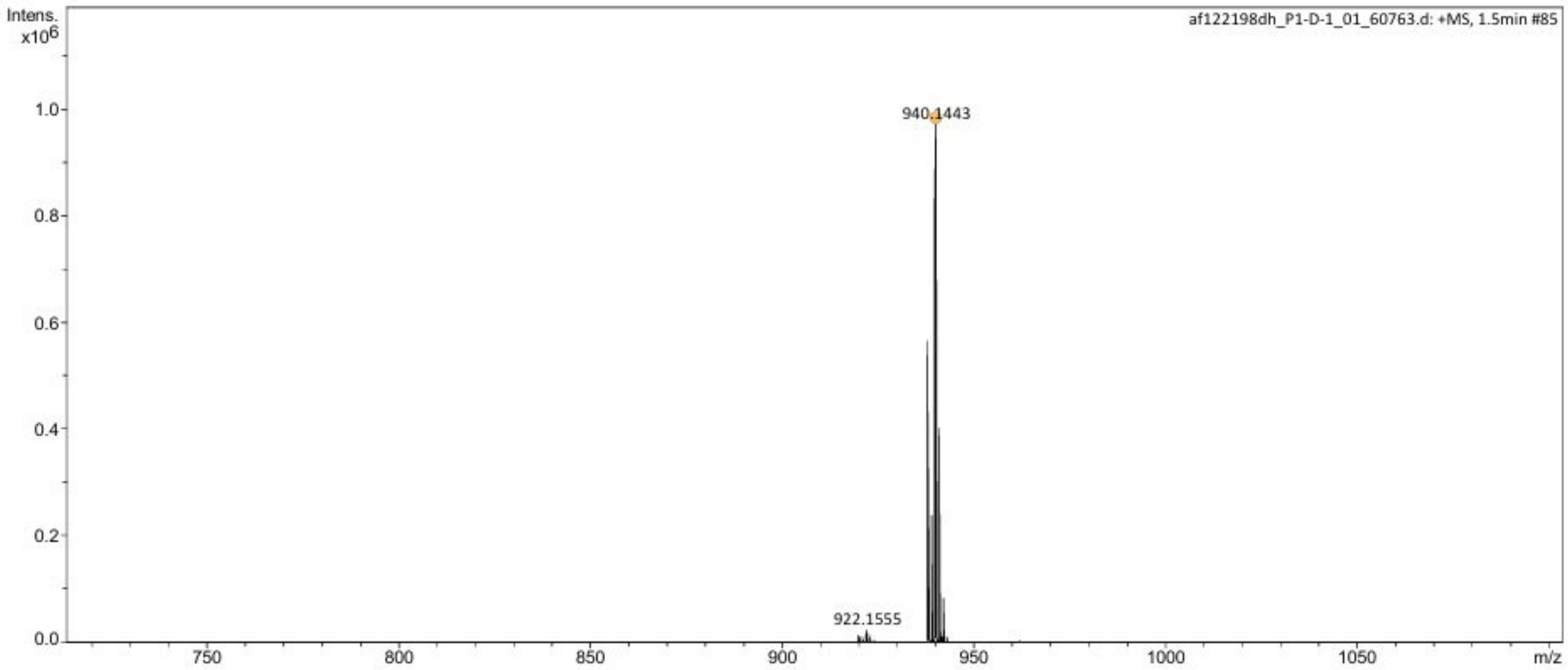

Meas. m/z	#	Ion Formula	m/z	err [ppm]	err [mDa]	mSigma	Mean err [ppm]
940.1443	1	C35H23F10IrN7O	940.1428	-1.4	-1.3	12.4	-1.2

Figure S62: HRMS (ESI positive mode) of IrCN3(M2Y1)

York - Chemistry - Mass Spectrometry Service Report

DRH-098-2_af122199dh

Analysis Information

Analysis Filename	af122199dh_P1-D-2_01_60764.d	Acquisition Date	17/10/2025 11:06:52
Method	ESI_low mass_2c1s.m	Instrument	compact
Submission Name	af122199dh	ESI	Positive

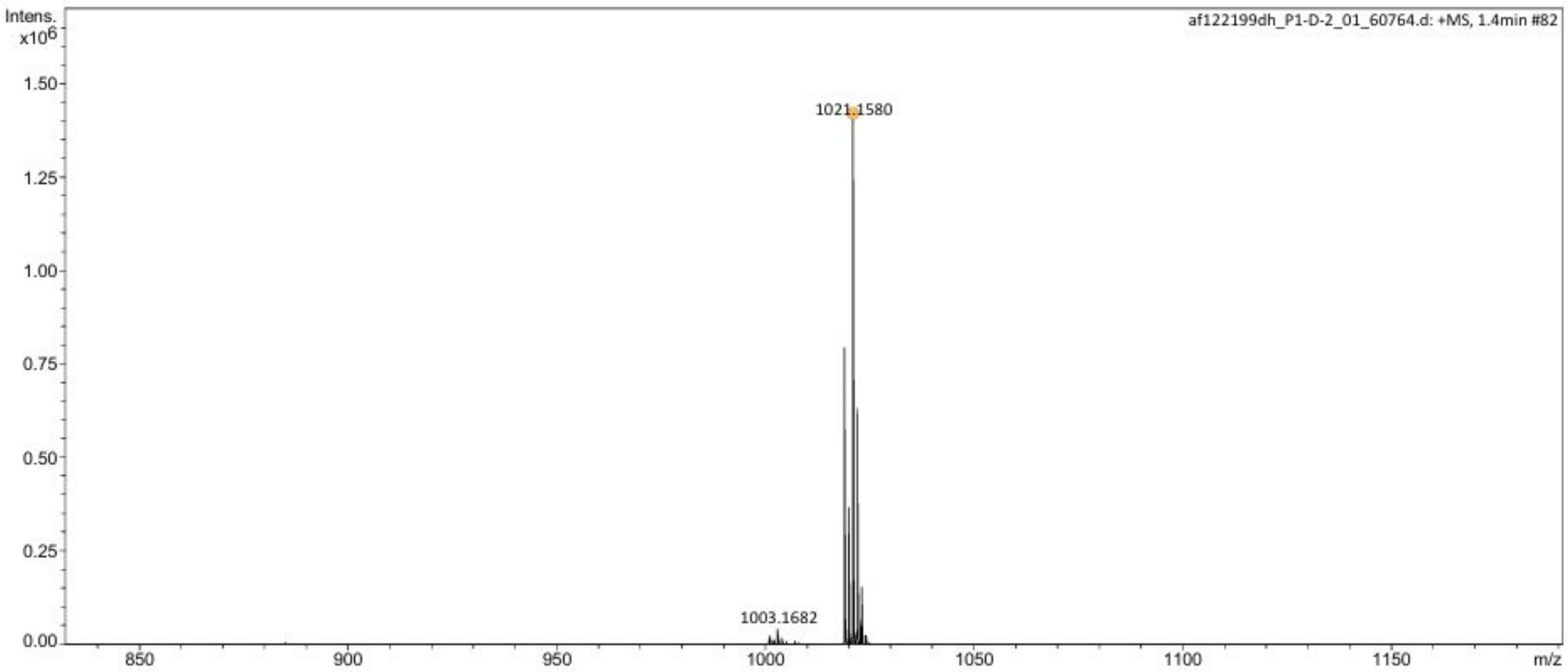

Meas. m/z	#	Ion Formula	m/z	err [ppm]	err [mDa]	mSigma	Mean err [ppm]
1021.1580	1	C40H26F10IrN6O3	1021.1530	-4.6	-4.7	6.2	-5.0

Figure S63: HRMS (ESI positive mode) of IrCN3(M12Y3)

York - Chemistry - Mass Spectrometry Service Report

DRH-098-3_af122185dh

Analysis Information

Analysis Filename	af122185dh_P1-B-6_01_60747.d	Acquisition Date	16/10/2025 14:26:44
Method	ESI_low mass_2c1s.m	Instrument	compact
Submission Name	af122185dh	ESI	Positive

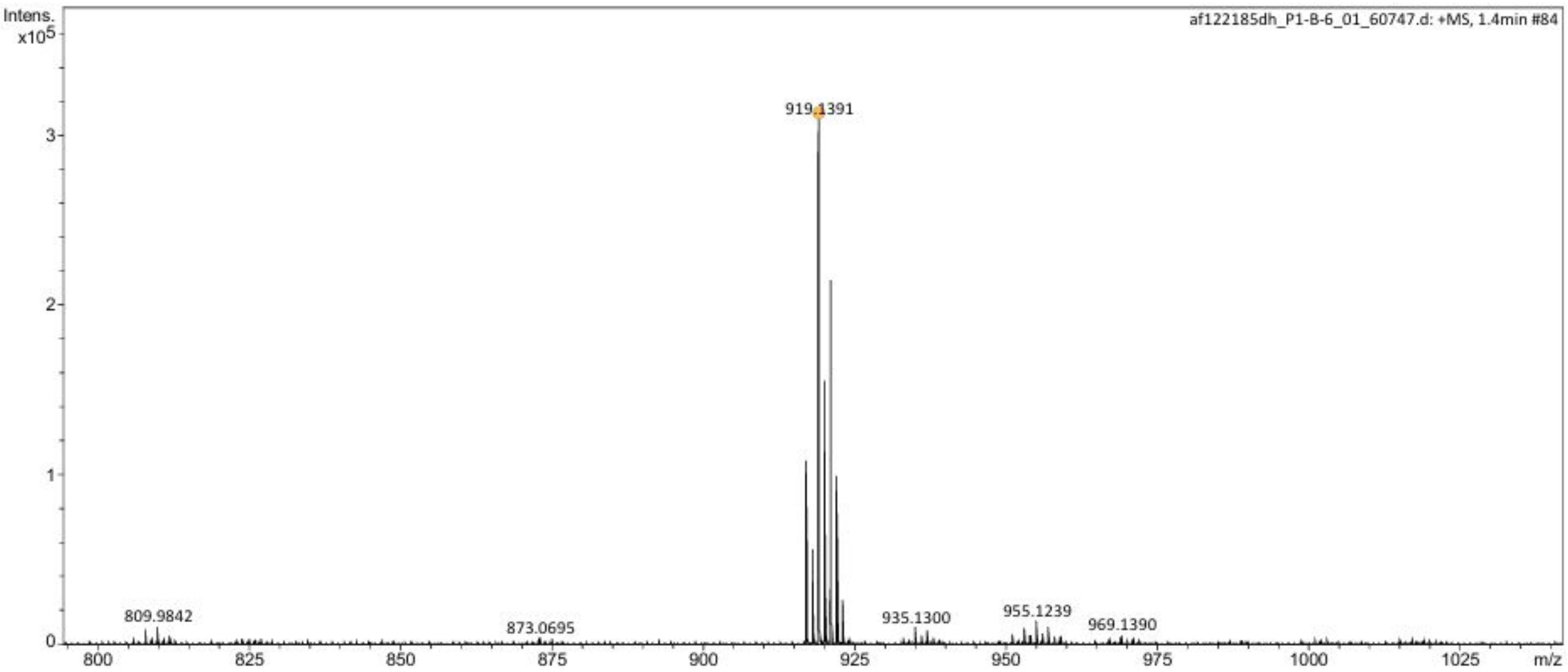

Meas. m/z	#	Ion Formula	m/z	err [ppm]	err [mDa]	mSigma	Mean err [ppm]
919.1391	1	C43H31BrIrN6O	919.1366	-4.1	-3.8	13.8	-4.5

Figure S64: HRMS (ESI positive mode) of IrCN2(M18Y4)

8. LCMS data of pure compounds

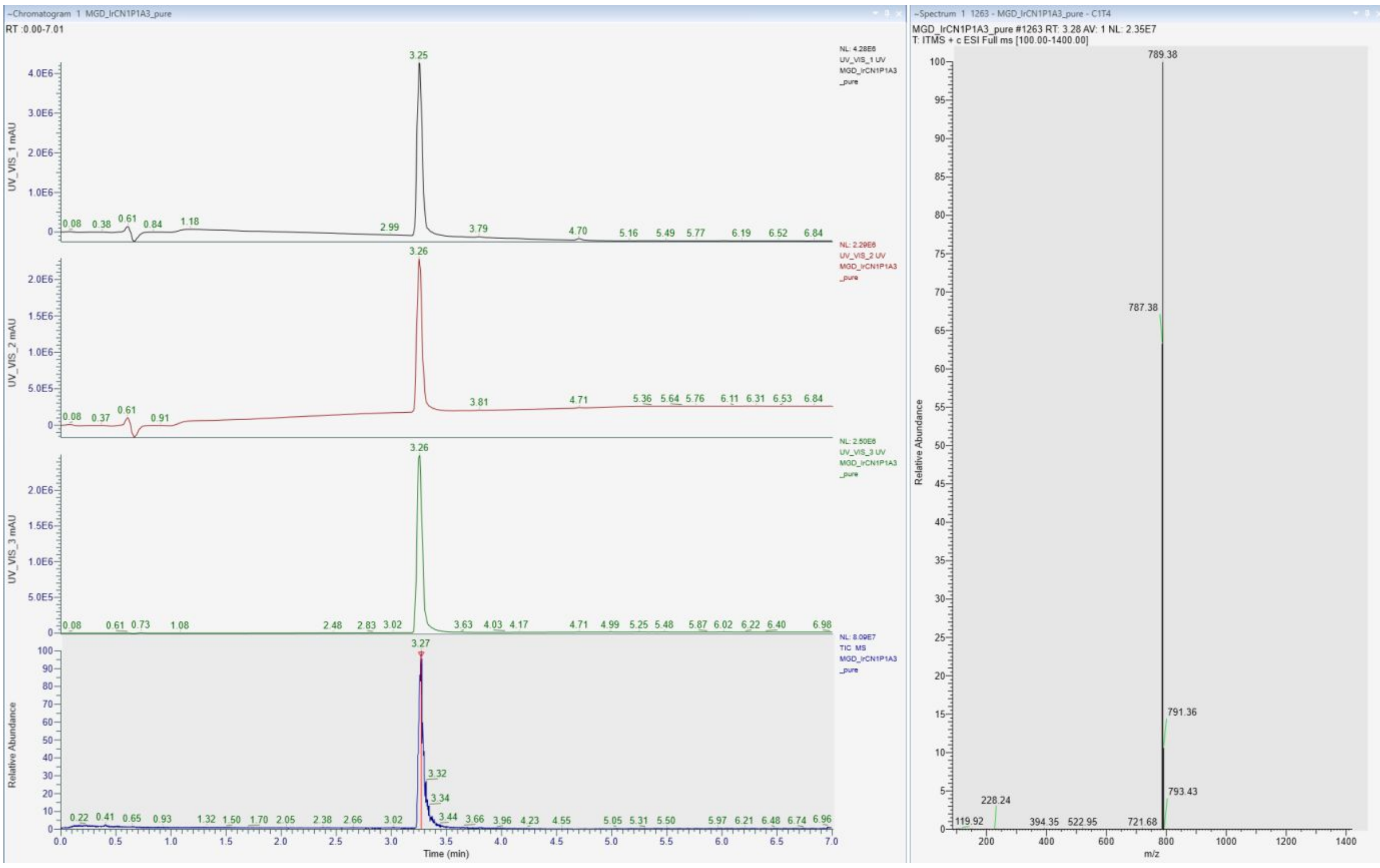

Figure S65: LCMS trace (7 min method) of IrCN1(P1A3)

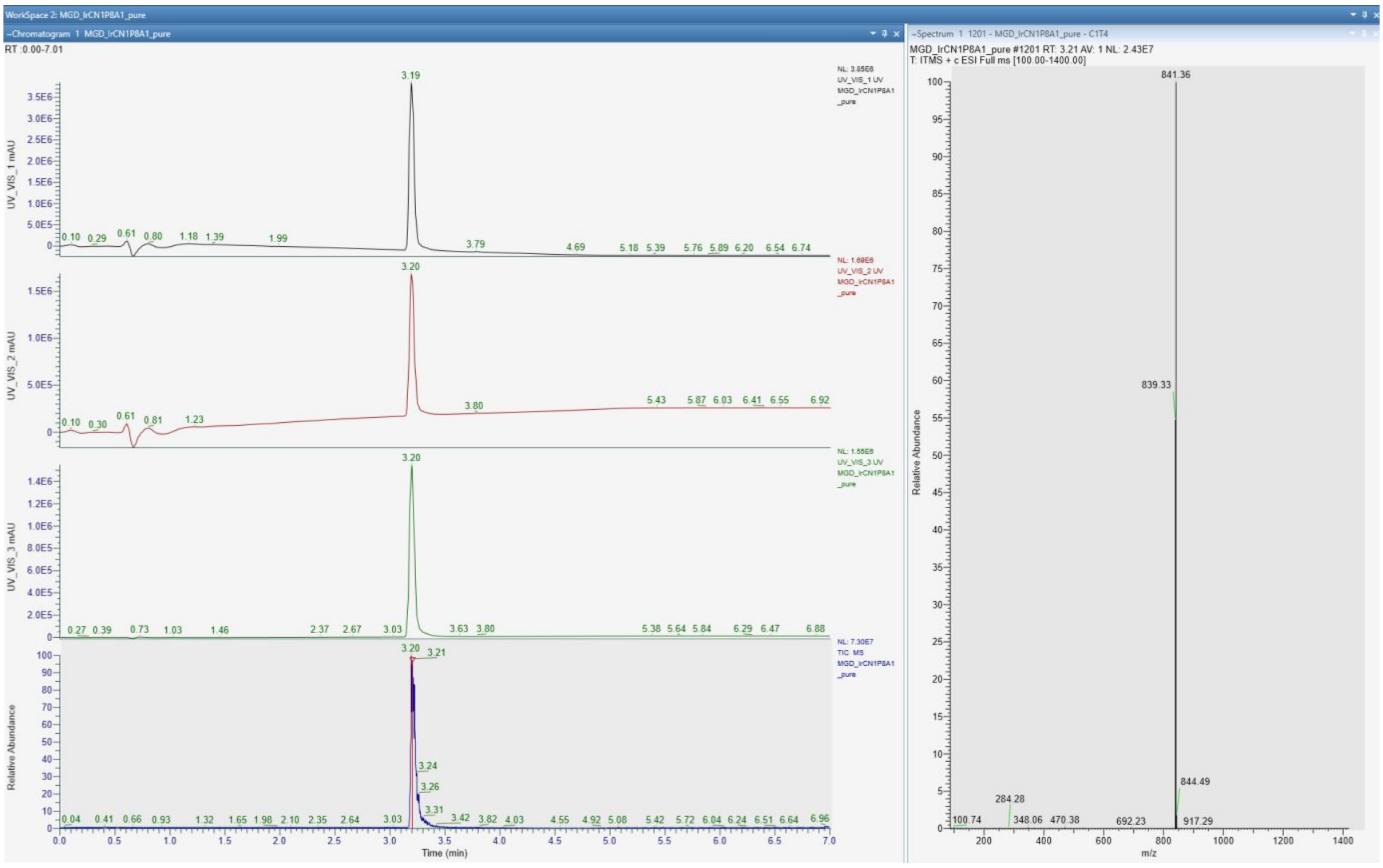

Figure S66: LCMS trace (7 min method) of IrCN1(P8A1)

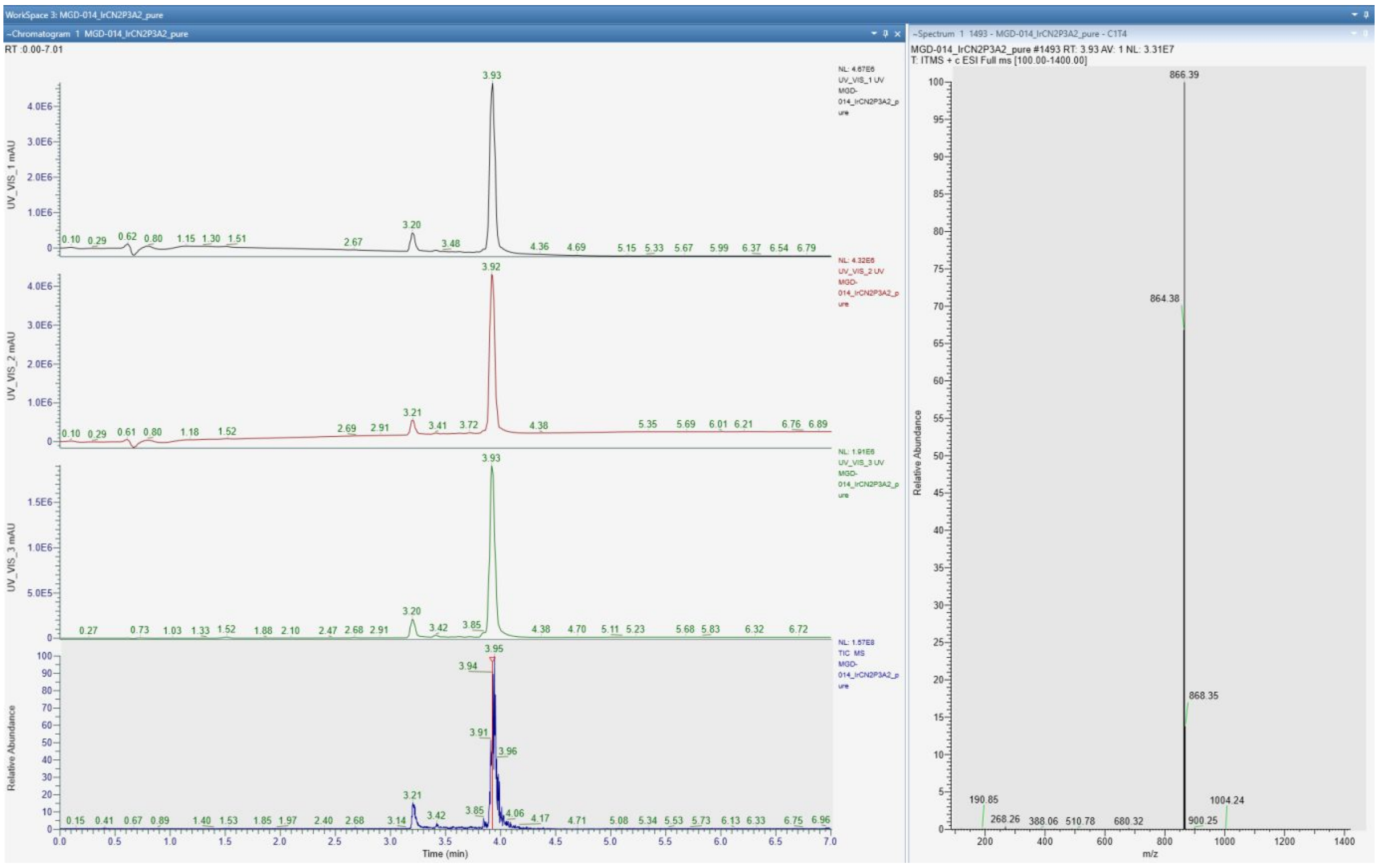

Figure S67: LCMS trace (7 min method) of IrCN2(P3A2)

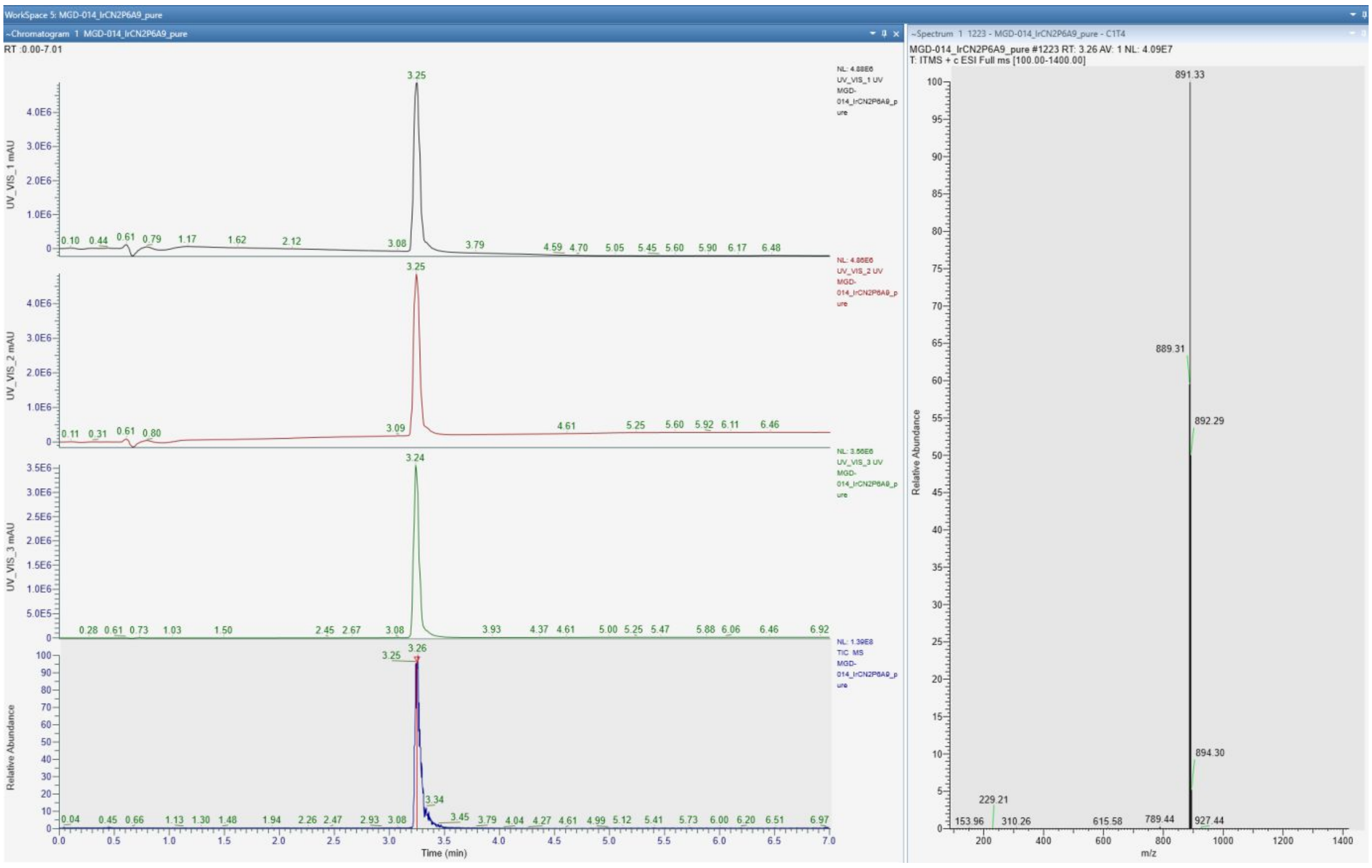

Figure S68: LCMS trace (7 min method) of IrCN2(P6A9)

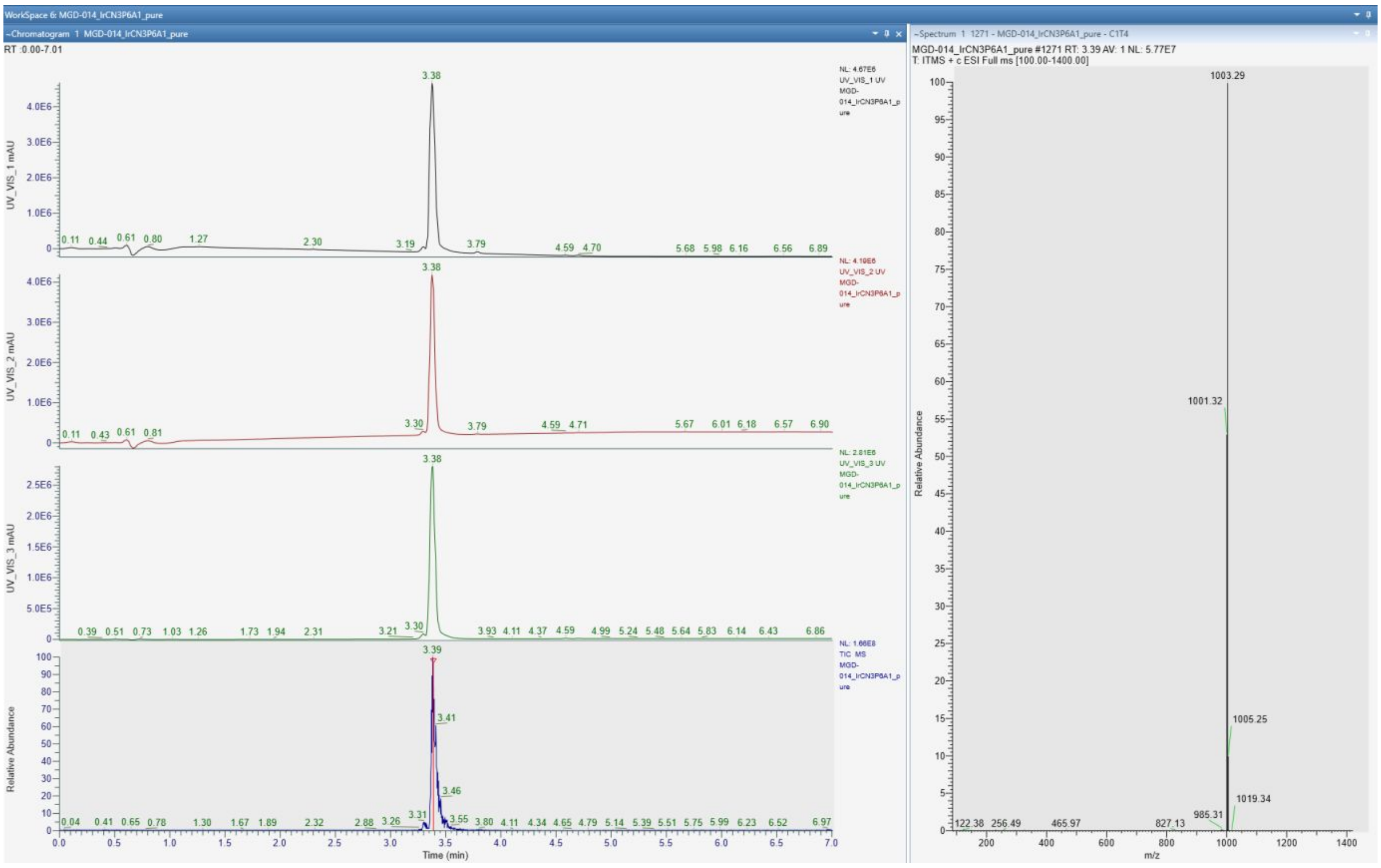

Figure S69: LCMS trace (7 min method) of IrCN3(P6A1)

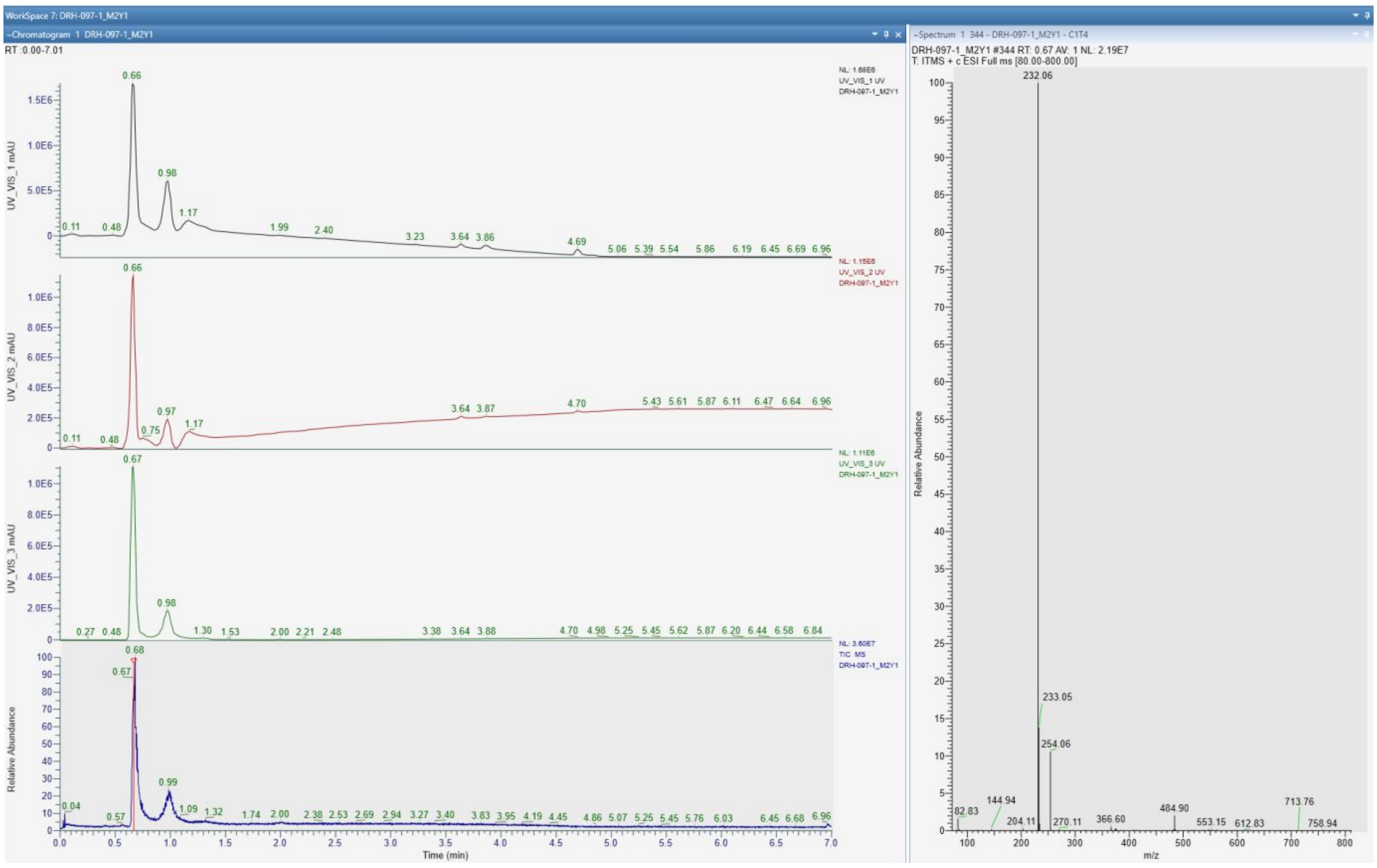

Figure S70: LCMS trace (7 min method) of M2Y1

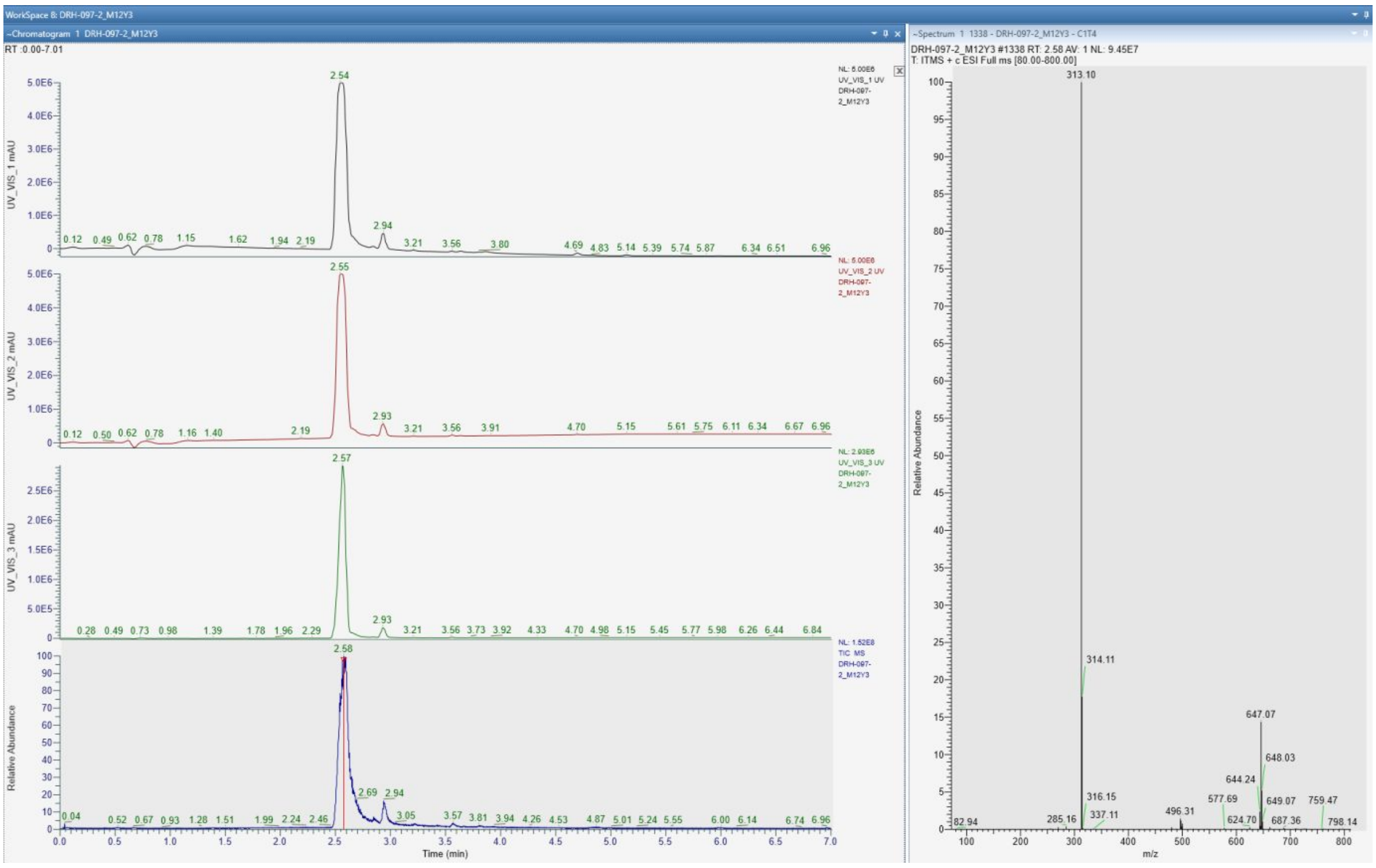

Figure S71: LCMS trace (7 min method) of M12Y3

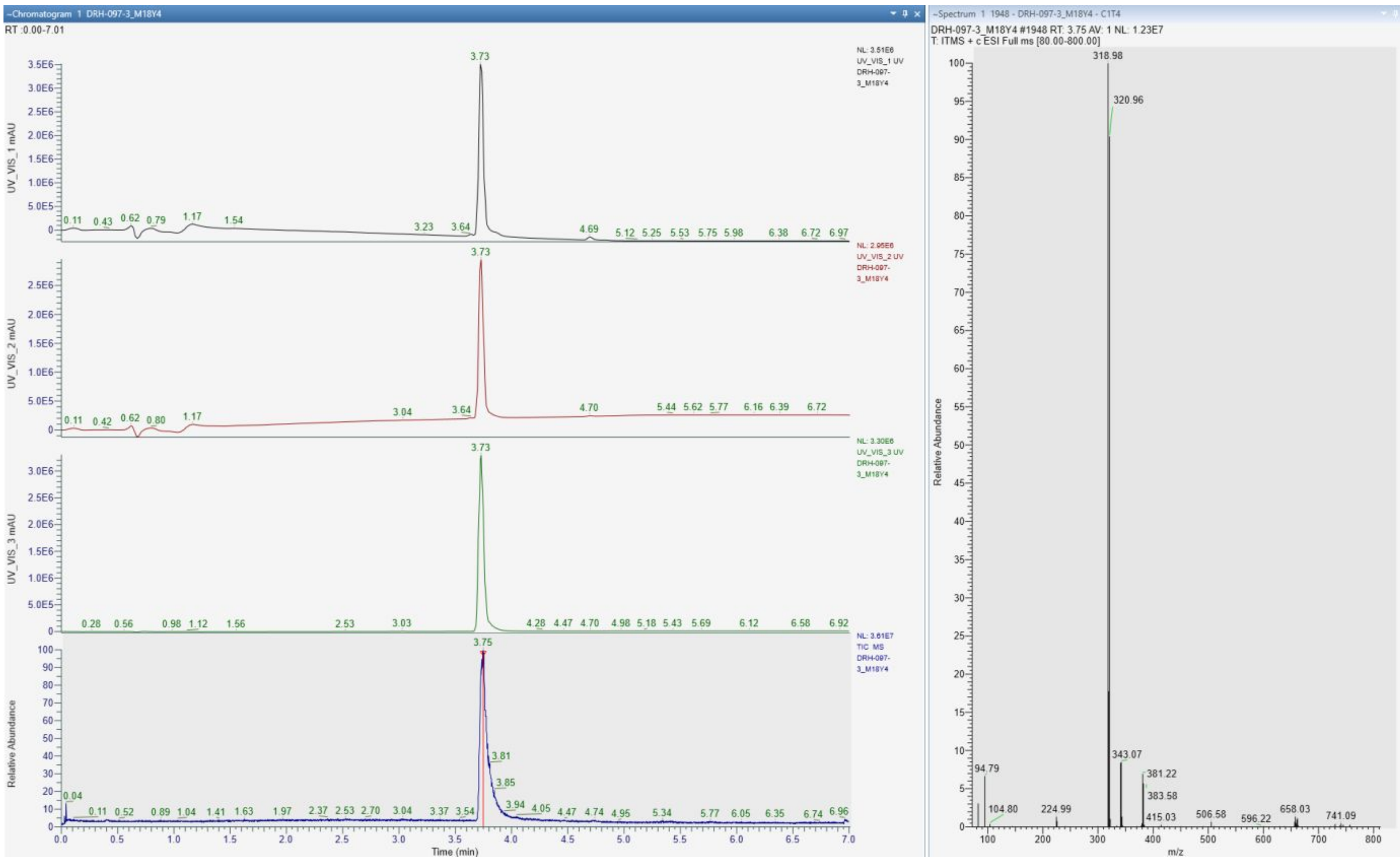

Figure S72: LCMS trace (7 min method) of M18Y4

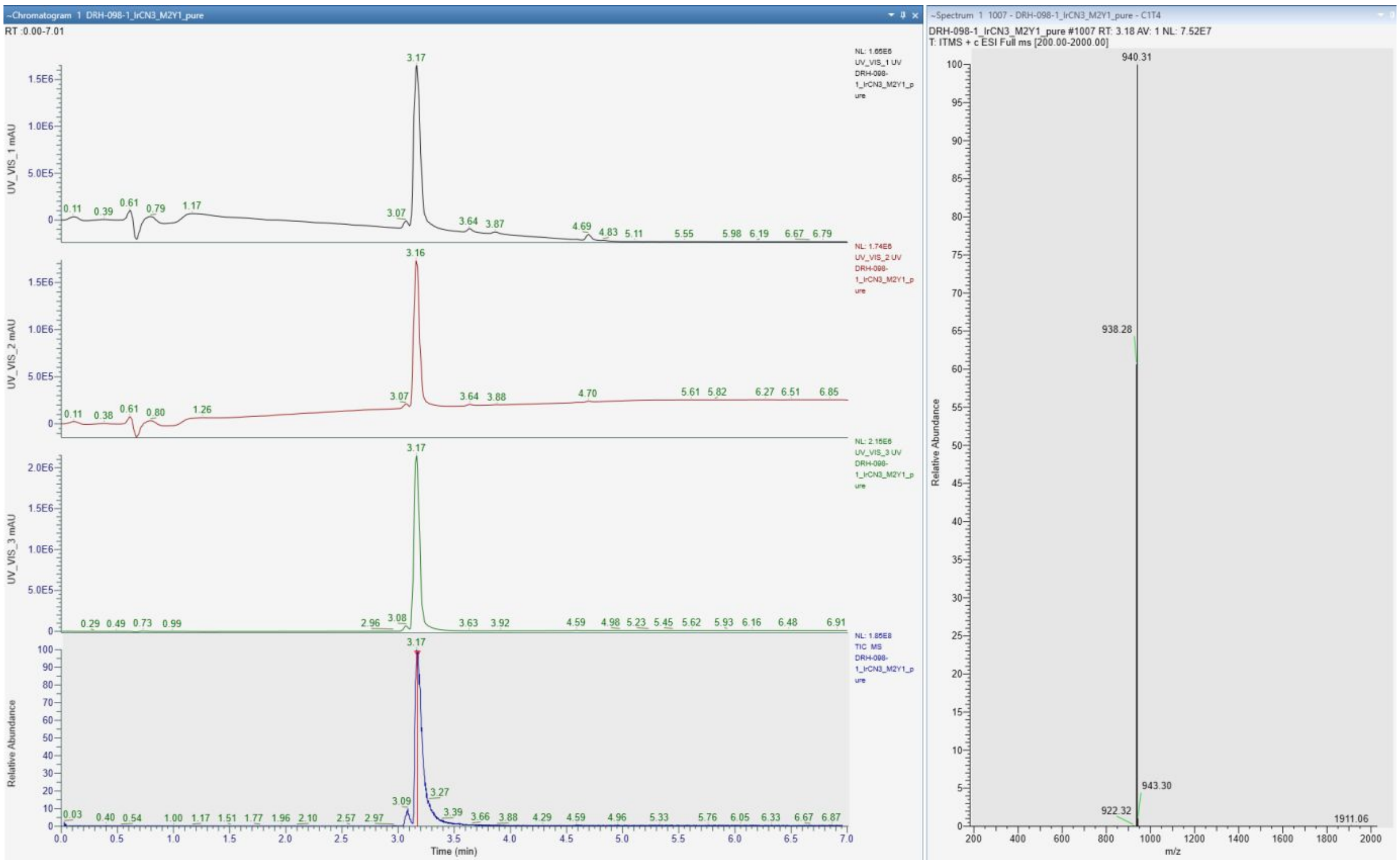

Figure S73: LCMS trace (7 min method) of IrCN3(M2Y1)

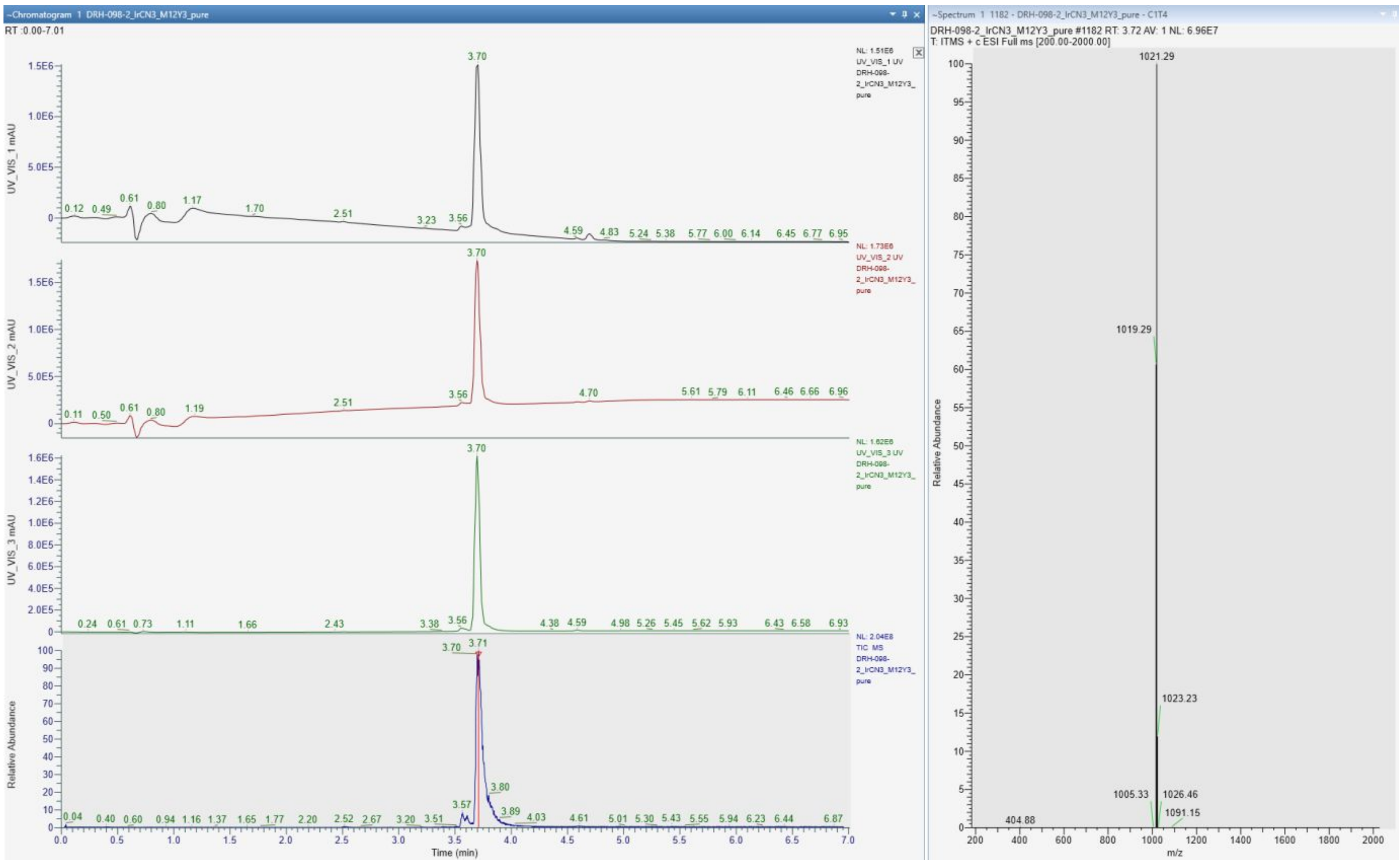

Figure S74: LCMS trace (7 min method) of IrCN3(M12Y3)

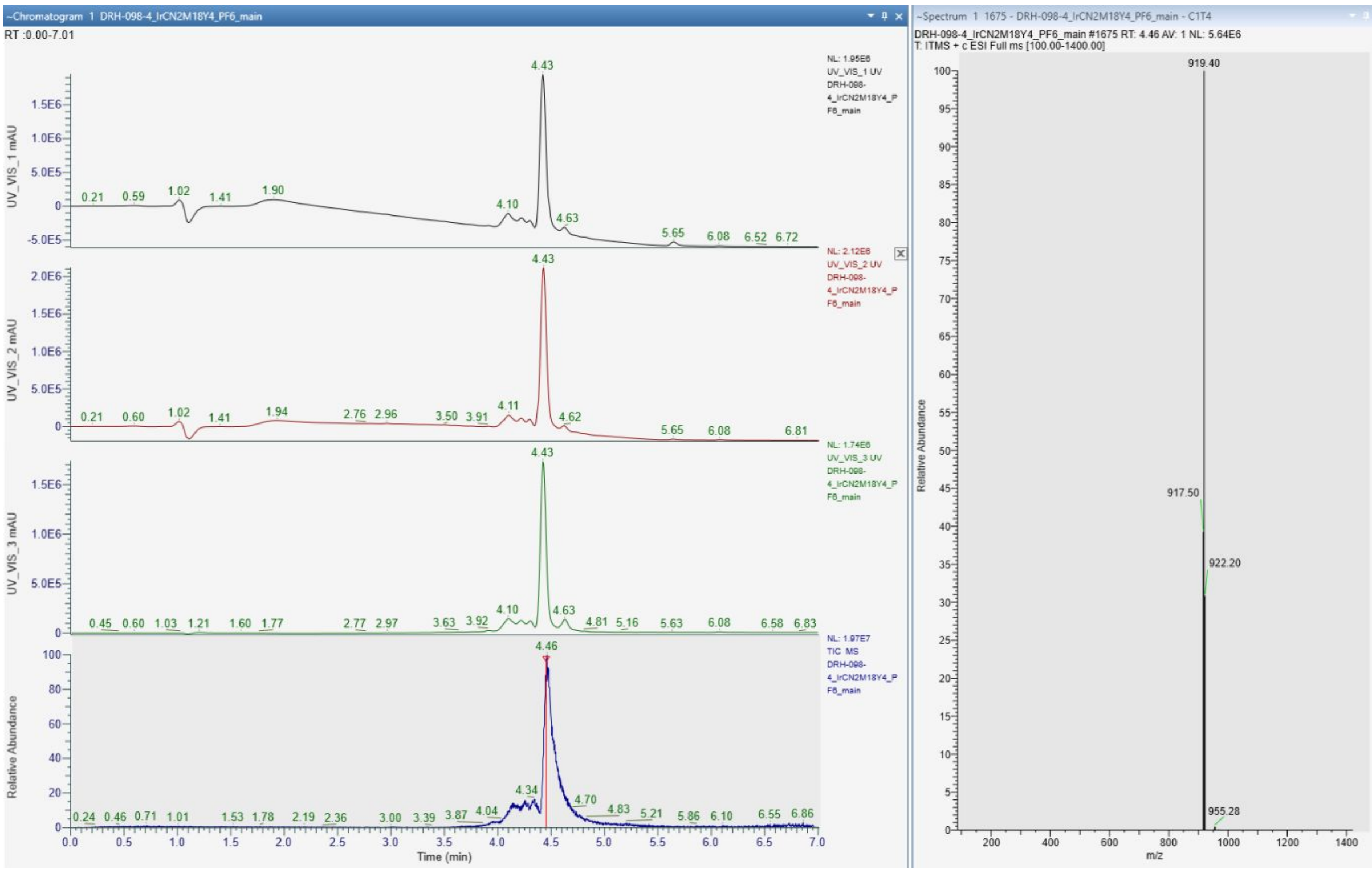

Figure S75: LCMS trace (7 min method) of IrCN2(M18Y4)-PF_6

9. X-Ray Crystallography

Diffraction data were collected at 110 K on an Oxford Diffraction XtaLAB Synergy HyPix-Arc 100 diffractometer with Cu-K_{α} radiation (λ = 1.54184 Å). The crystal was cooled with an Oxford Instruments Cryostream 1000. Diffractometer control, data collection, initial unit cell determination, frame integration and unit-cell refinement was carried out with “CrysAlisPro”.[6] Face-indexed absorption corrections were applied using spherical harmonics, implemented in SCALE3 ABSPACK scaling algorithm.[7] OLEX2[8] was used for overall structure solution and refinement . Within OLEX2, the algorithm used for structure solution was “ShelXT dual-space”.[9] Refinement was carried out by full-matrix least-squares used the SHELXL-97[9] algorithm within OLEX2. All non-hydrogen atoms were refined anisotropically. Crystalmaker® software was used to visualize the structures as well as generating the figures presented herein.

af25004: IrCN3 dimer CCDC 2561583

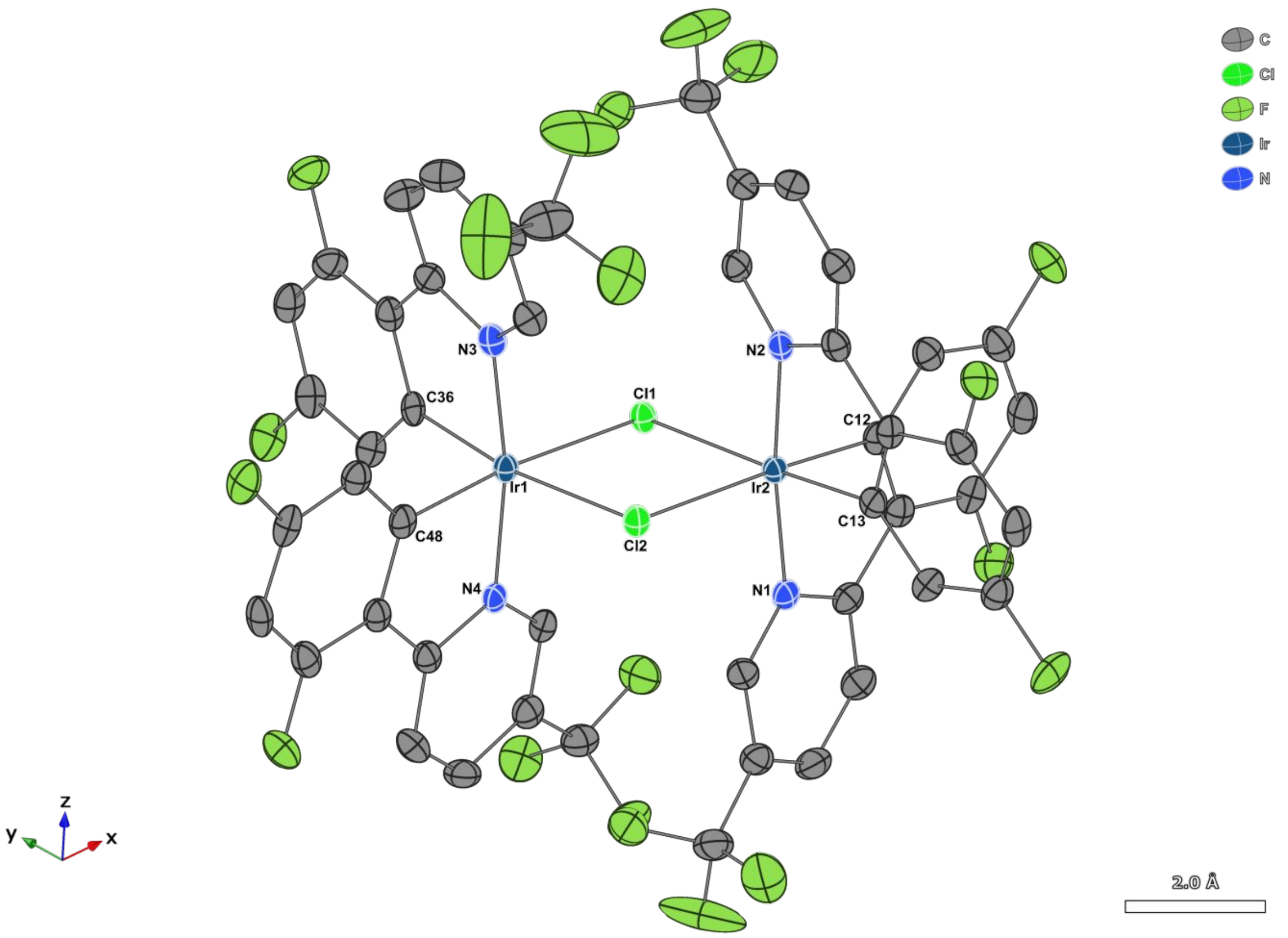

Figure S76: Structure, obtained by X-ray diffraction, of a single crystal of IrCN3 dimer (thermal ellipsoids are set at 50% probability). A DCM of crystallisation is not shown for clarity Selected interatomic lengths /Å: Ir1-N3 = 2.034(3); Ir1-N4 = 2.045(3); Ir2-N1 = 2.026(3); Ir2-N2 = 2.037(3); Ir1-C36 = 1.984(3); Ir1-C48 = 1.992(3); Ir2-C12 = 1.991(3); Ir2-C13 = 1.983(3); Ir1-Cl1 = 2.5037(7); Ir1-Cl2 = 2.5257(7); Ir2-Cl1 = 2.5210(7); Ir2-Cl2 = 2.5215(7). Selected interatomic angles /°: Cl1-Ir1-Cl2 = 81.29(2); Cl1-Ir2-Cl2 = 81.03(2); C48-Ir1-N3 = 91.45(12); C36-Ir-N4 = 93.90(11); C13-Ir2-N2 = 80.81(12); C12-Ir2-N1 = 80.57(12); C36-Ir1-C48 = 91.41(12); C13-Ir2-C12 = 87.93(12); Ir1-Cl1-Ir2 = 99.14(2); Ir2-Cl2-Ir1 = 98.54(2).

Table S11: X-Ray Diffraction Data for IrCN3 dimer

Identification code	af25004
Empirical formula	$C_{49}H_{22}Cl_4F_{20}Ir_2N_4$
Formula weight	1572.90
Temperature/K	110.00(3)
Crystal system	monoclinic
Space group	$P2_1/n$
a/Å	10.77457(4)
b/Å	22.34957(13)
c/Å	20.30294(9)
α/°	90
β/°	90.7744(4)
γ/°	90
Volume/Å^3	4888.64(4)
Z	4
ρ_{calc}g/cm^3	2.137
μ/mm^{-1}	13.487
F(000)	2984.0
Crystal size/mm^3	0.189 x 0.06 x 0.055
Radiation	Cu Kα (λ = 1.54184)
2Θ range for data collection/°	5.882 to 136.49
Index ranges	-12 ≤ h ≤ 8, -26 ≤ k ≤ 26, -24 ≤ l ≤ 24
Reflections collected	45563
Independent reflections	8932 [R_{int} = 0.0346, R_{sigma} = 0.0251]
Data/restraints/parameters	8932/0/712
Goodness-of-fit on F^2	1.053
Final R indexes [I>=2σ (I)]	R_1 = 0.0224, wR_2 = 0.0573
Final R indexes [all data]	R_1 = 0.0237, wR_2 = 0.0582
Largest diff. peak/hole / e Å^{-3}	1.03/-0.62

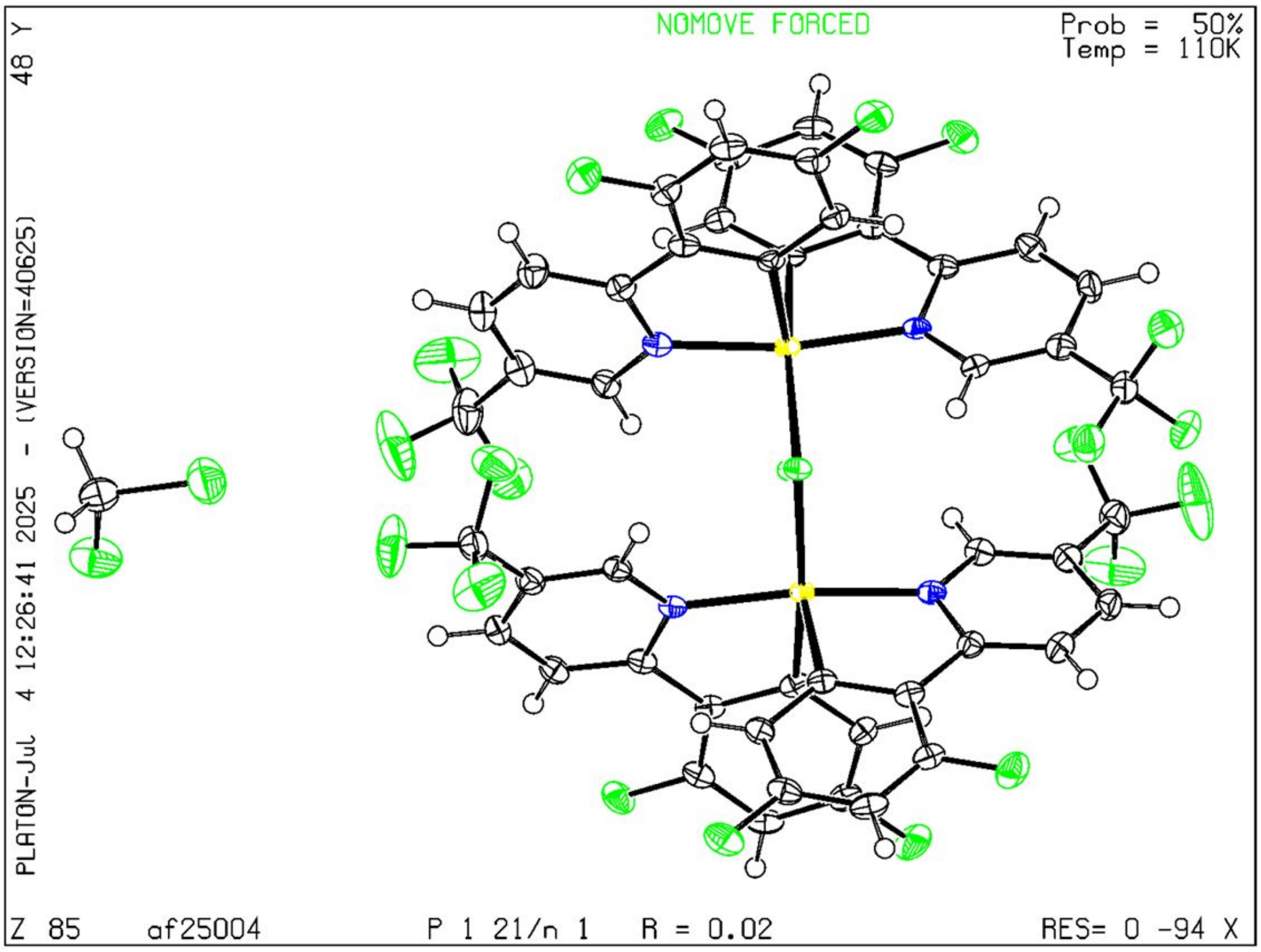

Figure S77: ORTEP representation of IrCN3 dimer (thermal ellipsoids are set at 50% probability). CCDC deposition number 2561583

af25011a: IrCN3(M1Y2) CCDC 2561584

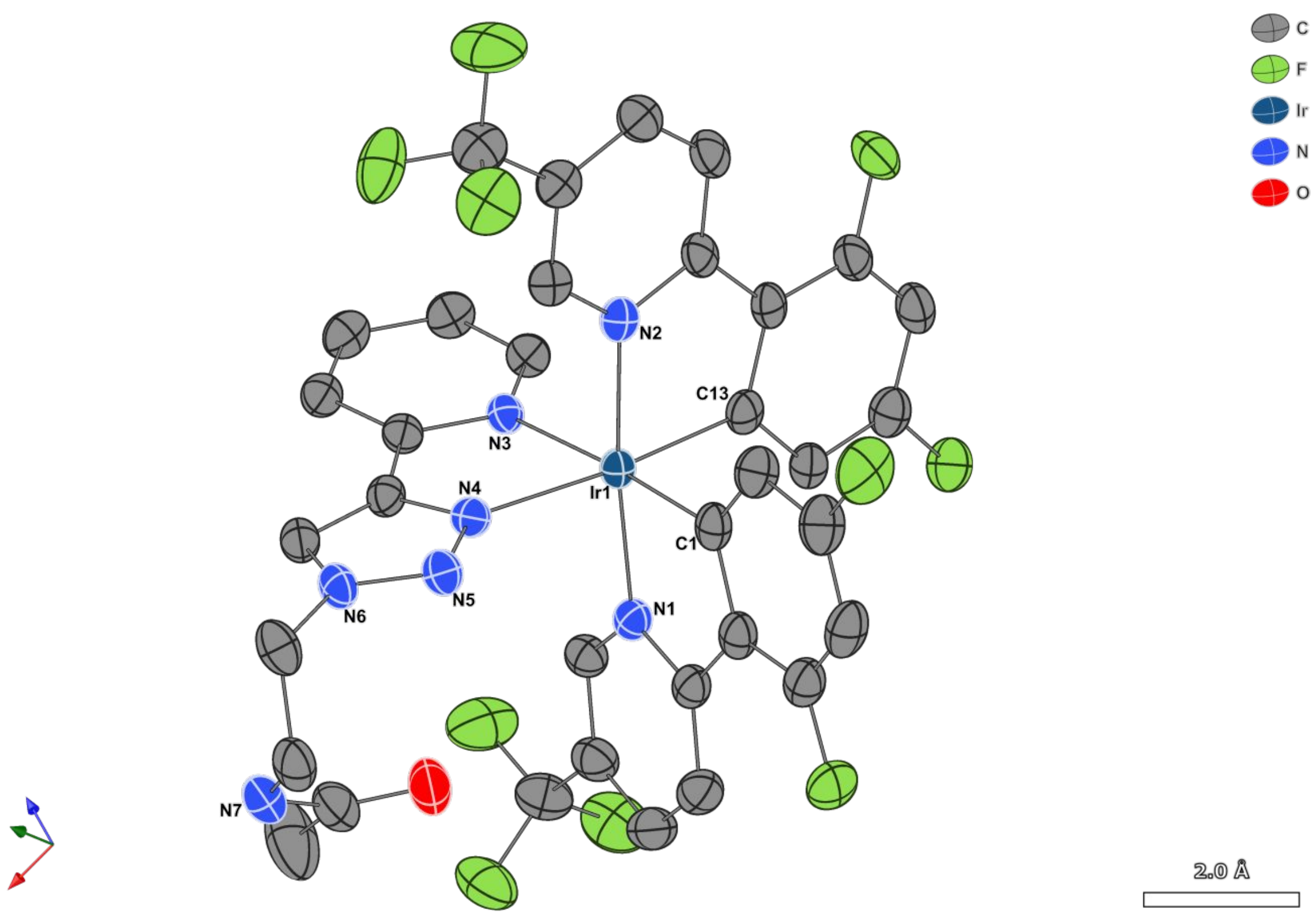

Figure S78: Structure, obtained by X-ray diffraction, of a single crystal of IrCN3(M1Y2) (thermal ellipsoids are set at 50% probability). The Cl^- counterion and benzene solvent of crystallization are not shown for clarity. Selected interatomic lengths /Å: Ir1-N1 = 2.051(2; Ir1-N2 = 2.045(2); Ir1-N3 = 2.169(2); Ir1-N4 = 2.127(2); Ir1-C1 = 2.003(3); Ir1-C13 = 2.001(3). Selected interatomic angles /°: N3-Ir1-N4 = 76.77(8); N1-Ir1-C1 = 80.74(10); N2-Ir1-C13 = 80.41(10); N1-Ir1-N2 = 173.74(8).

Refinement special details

Modelled using a solvent mask with a predicted volume of 46 cubic angstroms per asymmetric unit containing 8 electrons. This is most likely to be a partially occupied water molecule at 60% occupancy.

Table S12: X-Ray Diffraction Data for IrCN3(M1Y2)

**Identification code**	**af25011a**
**Empirical formula**	$C_{50}H_{37}ClF_{10}IrN_7O$
**Formula weight**	1169.51
**Temperature/K**	109.97(15)
**Crystal system**	orthorhombic
**Space group**	Pbcn
**a/Å**	26.4870(2)
**b/Å**	19.9519(2)
**c/Å**	18.41250(10)
**α/°**	90
**β/°**	90
**γ/°**	90

**Volume/Å^3**	9730.38(13)
**Z**	8
**ρ_{calc}g/cm^3**	1.597
**μ/mm^{-1}**	6.549
**F(000)**	4624.0
**Crystal size/mm^3**	0.138 × 0.071 × 0.069
**Radiation**	Cu Kα (λ = 1.54184)
**2Θ range for data collection/°**	5.546 to 136.478
**Index ranges**	-27 ≤ h ≤ 31, -24 ≤ k ≤ 24, -22 ≤ l ≤ 21
**Reflections collected**	48289
**Independent reflections**	8893 [R_{int} = 0.0288, R_{sigma} = 0.0213]
**Data/restraints/parameters**	8893/0/633
**Goodness-of-fit on F^2**	1.019
**Final R indexes [I>=2σ (I)]**	R_1 = 0.0238, wR_2 = 0.0585
**Final R indexes [all data]**	R_1 = 0.0296, wR_2 = 0.0627
**Largest diff. peak/hole / e Å^{-3}**	1.16/-0.48

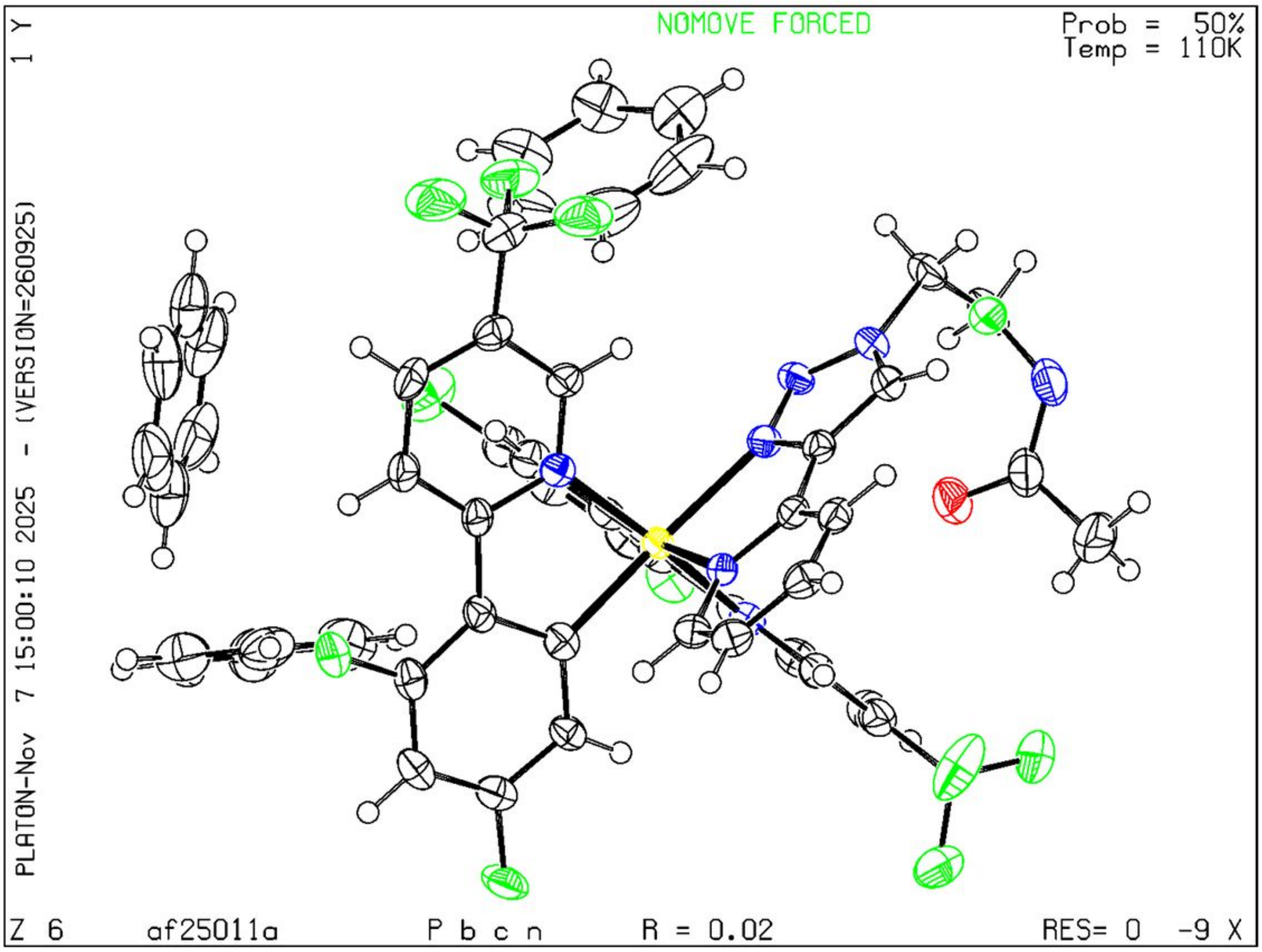

Figure S79: ORTEP representation of IrCN3(M2Y1) (thermal ellipsoids are set at 50% probability). CCDC deposition number 2561584

af26016: IrCN2(P6A9) CCDC 2561585

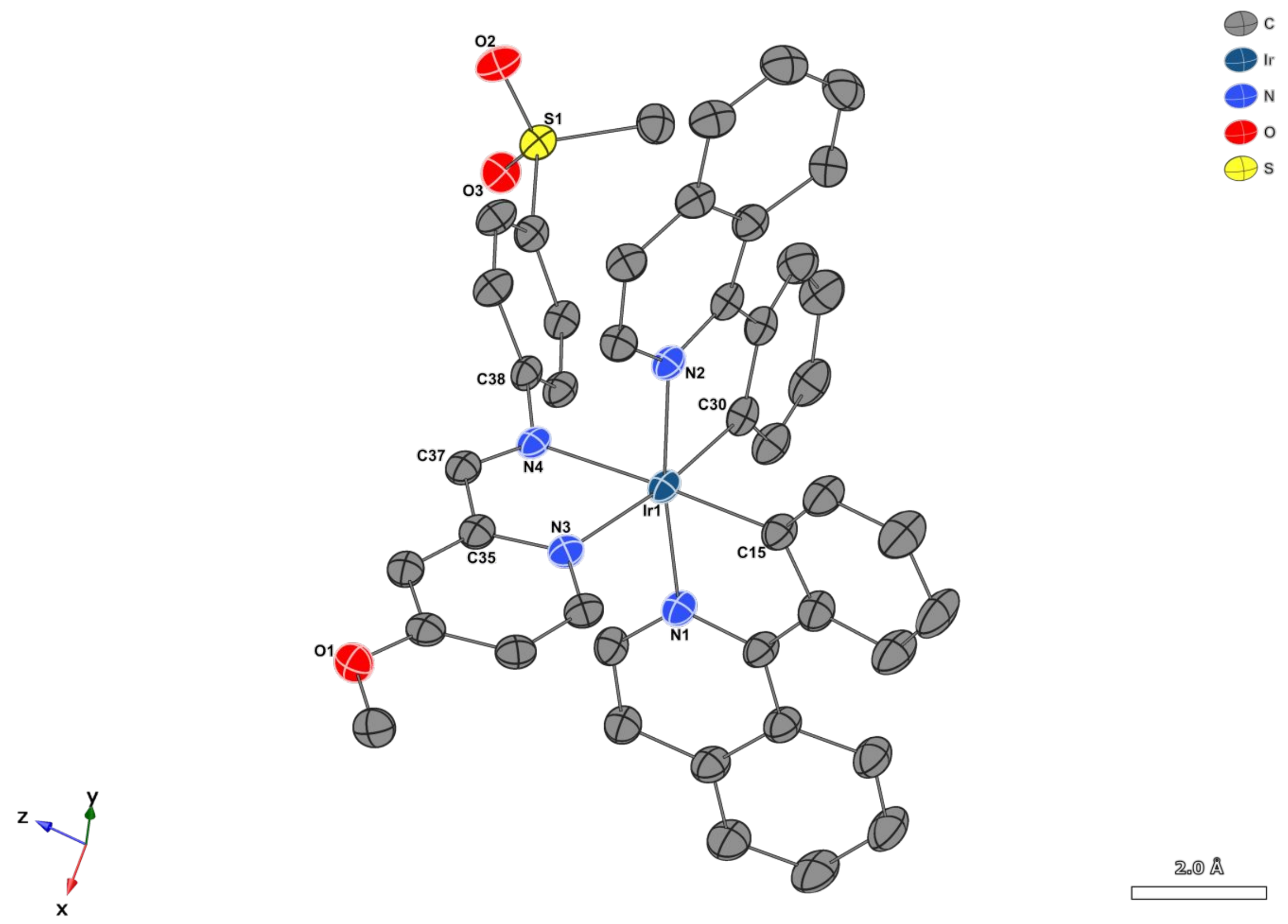

Figure S80: Structure, obtained by X-ray diffraction, of a single crystal of IrCN2(P6A9) (thermal ellipsoids are set at 50% probability). The PF_6^- counterion is not shown for clarity. Selected interatomic lengths /Å: Ir1-N1 2.044(2); Ir1-N2 = 2.048(2); Ir1-N3 = 2.152(2); Ir1-N4 = 2.155(2); Ir1-C15 = 2.003(3); Ir1-C30 = 2.014(3). Selected interatomic angles /°: N3-Ir1-N4 = 75.17(9); N1-Ir1-C15 = 79.19(11); N2-Ir1-C30 = 79.75(11); N1-Ir1-N2 = 171.96(9).

Refinement special details

A solvent mask was calculated and 58 electrons were found in a volume of 257\%A^3^ in 1 void per unit cell. This is consistent with the presence of 0.7[C4H10O] per asymmetric unit which accounts for 59 electrons per unit cell. The PF_6^- anion was restrained in two positions with 0.7 and 0.3 occupancy. A high amount of residual density (2.60) is located around the Ir atom. This is due to Fourier ripples from the high electron density in the heavy Ir atom.

Table S13: X-Ray Diffraction Data for IrCN2(P6A9)

**Identification code**	**af26016**
**Empirical formula**	$C_{46.8}H_{41}F_6IrN_4O_{3.7}PS$
**Formula weight**	1087.86
**Temperature/K**	110.0(4)
**Crystal system**	triclinic
**Space group**	P-1
**a/Å**	11.45040(10)
**b/Å**	11.92730(10)

**c/Å**	16.8441(2)
**α/°**	94.0540(10)
**β/°**	102.1290(10)
**γ/°**	106.8430(10)
**Volume/Å^3**	2131.22(4)
**Z**	2
**ρ_{calc}g/cm^3**	1.695
**μ/mm^{-1}**	7.535
**F(000)**	1083.0
**Crystal size/mm^3**	0.13 × 0.07 × 0.05
**Radiation**	Cu Kα (λ = 1.54184)
**2Θ range for data collection/°**	5.42 to 136.494
**Index ranges**	-13 ≤ h ≤ 13, -14 ≤ k ≤ 14, -20 ≤ l ≤ 20
**Reflections collected**	39531
**Independent reflections**	7802 [R_{int} = 0.0388, R_{sigma} = 0.0285]
**Data/restraints/parameters**	7802/151/606
**Goodness-of-fit on F^2**	1.044
**Final R indexes [I>=2σ (I)]**	R_1 = 0.0269, wR_2 = 0.0686
**Final R indexes [all data]**	R_1 = 0.0278, wR_2 = 0.0692
**Largest diff. peak/hole / e Å^{-3}**	2.74/-0.93

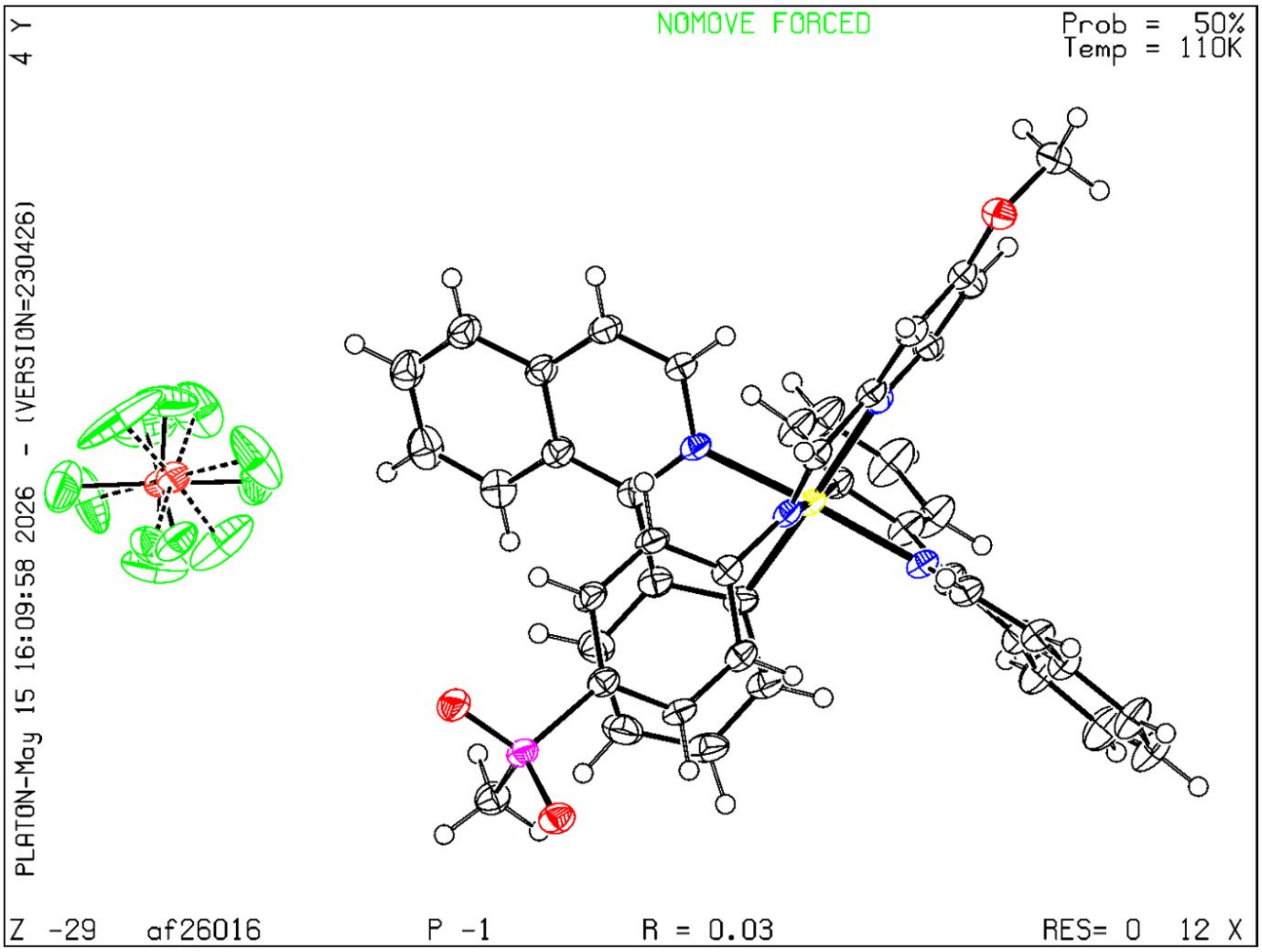

Figure S81: ORTEP representation of IrCN2(P6A9) (thermal ellipsoids are set at 50% probability). CCDC deposition number 2561585

10. NMR Spectral data for Organic and Inorganic Compounds

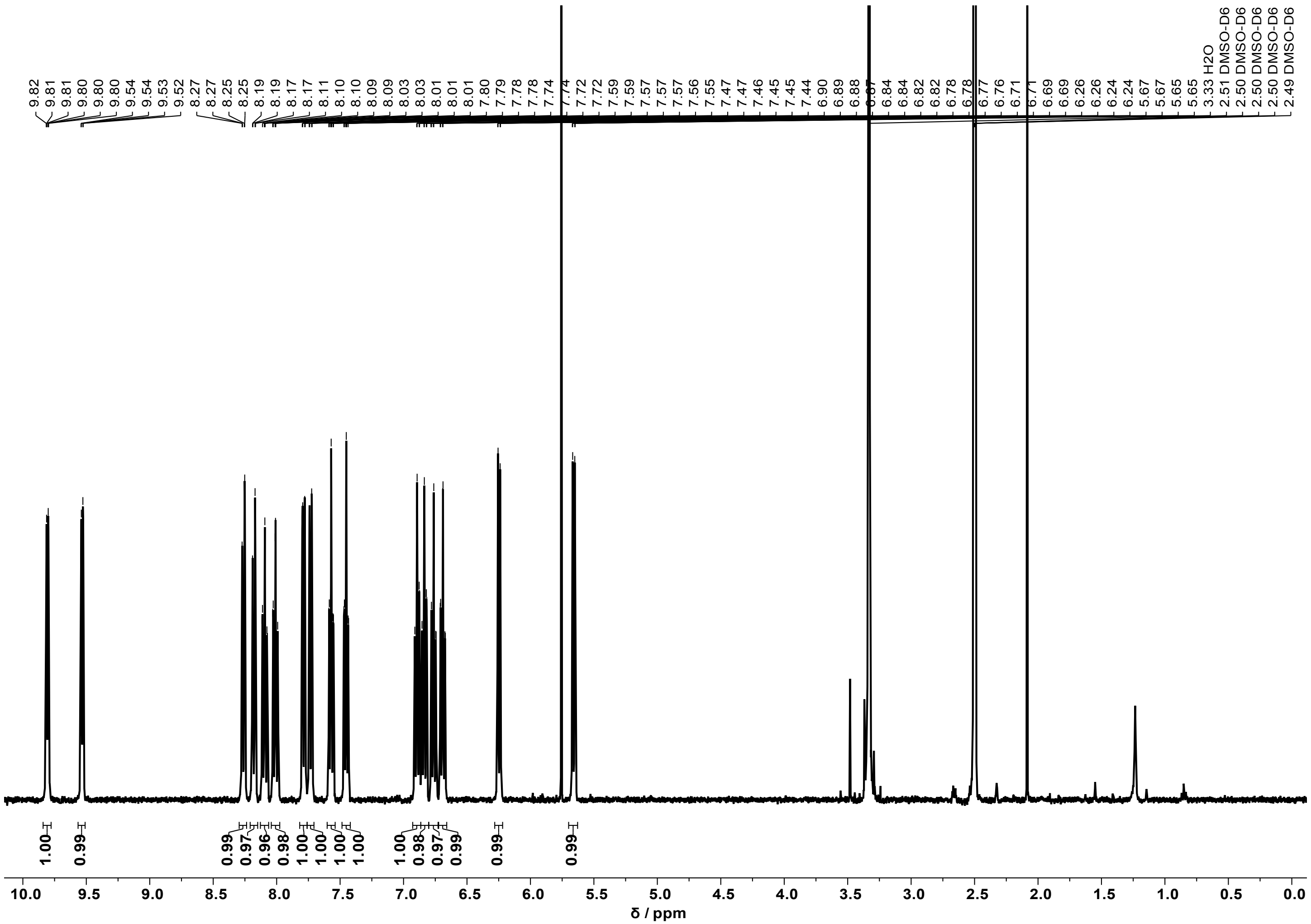

Figure S82: ^{1}H NMR spectrum (400 MHz, DMSO-d_6) of IrCN1 dimer

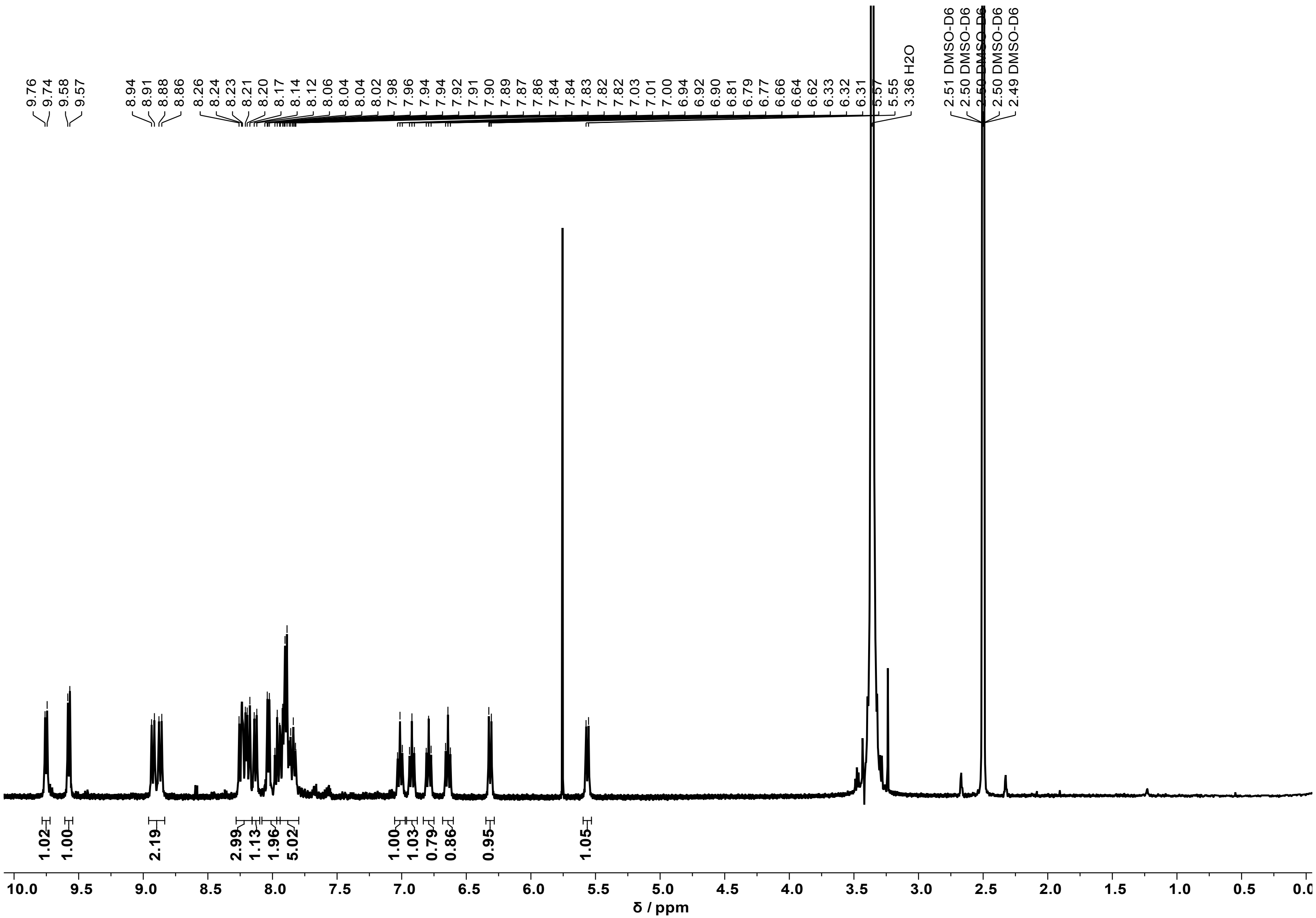

Figure S83: ^{1}H NMR spectrum (400 MHz, DMSO-d_6) of IrCN2 dimer

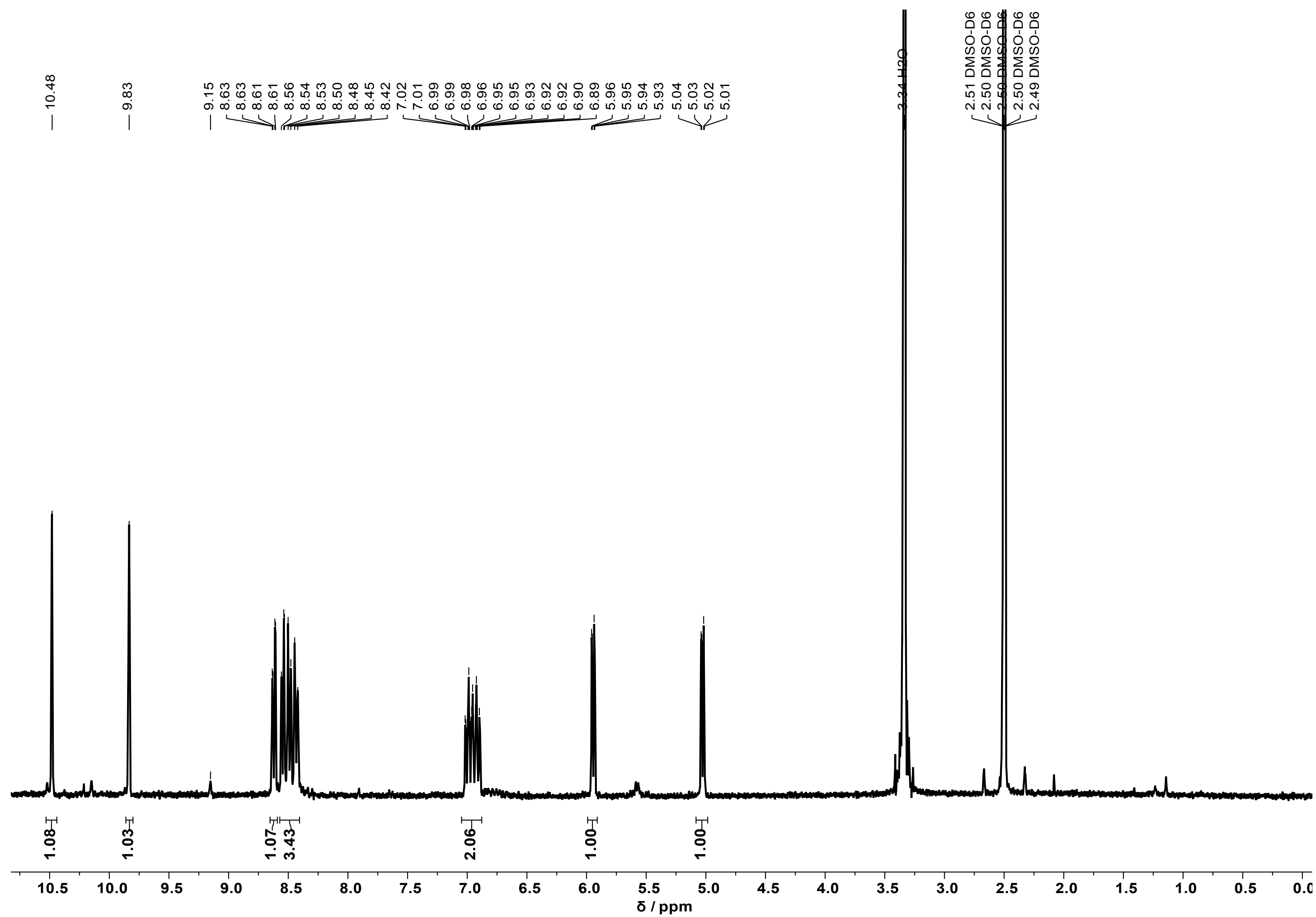

Figure S84: ^{1}H NMR spectrum (400 MHz, DMSO-d_6) of IrCN3 dimer

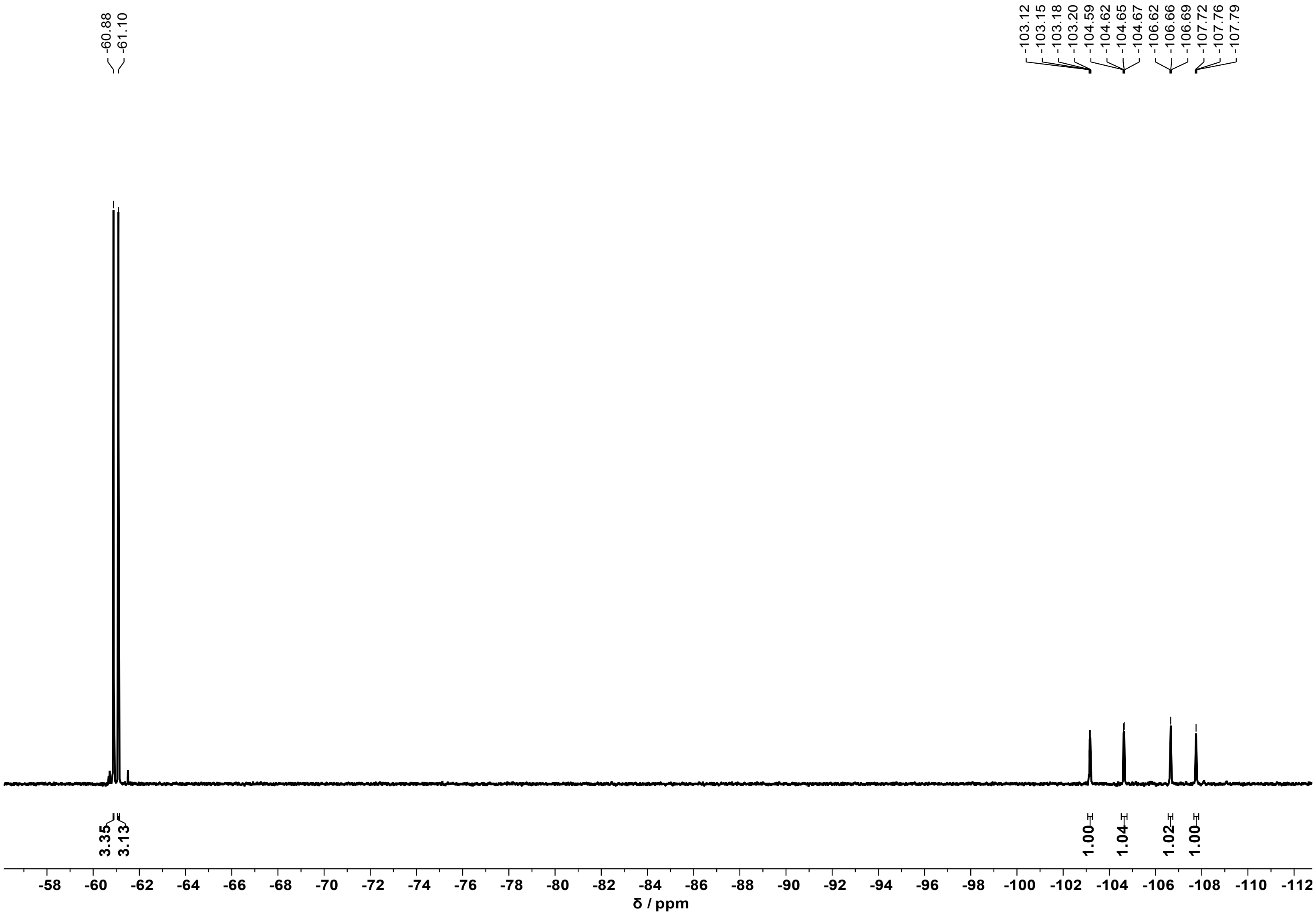

Figure S85: ^{19}F NMR spectrum (376 MHz, DMSO-d_6) of IrCN3 dimer

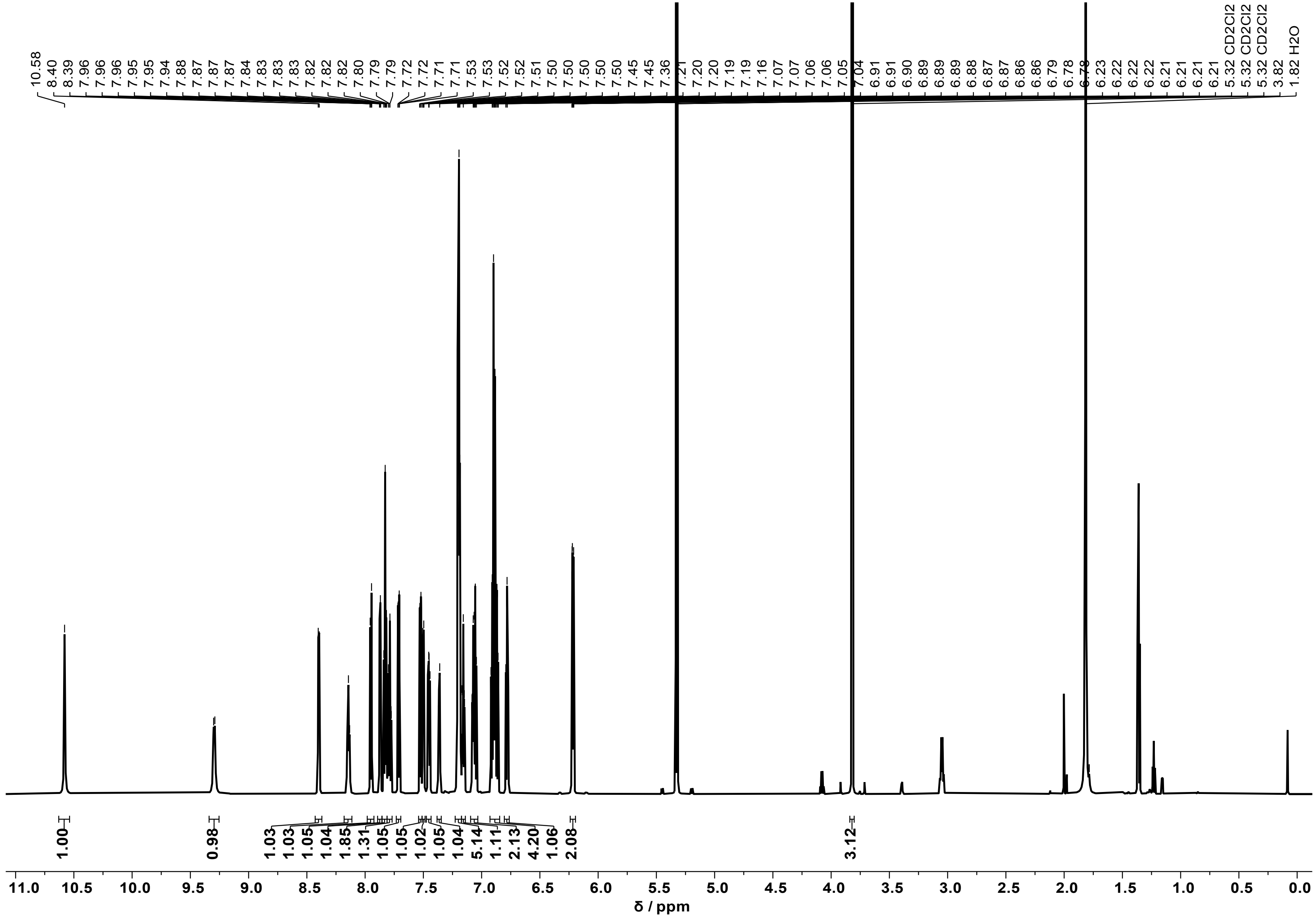

Figure S86: ^{1}H NMR spectrum (700 MHz, DCM-d_2) of IrCN1(P1A3)

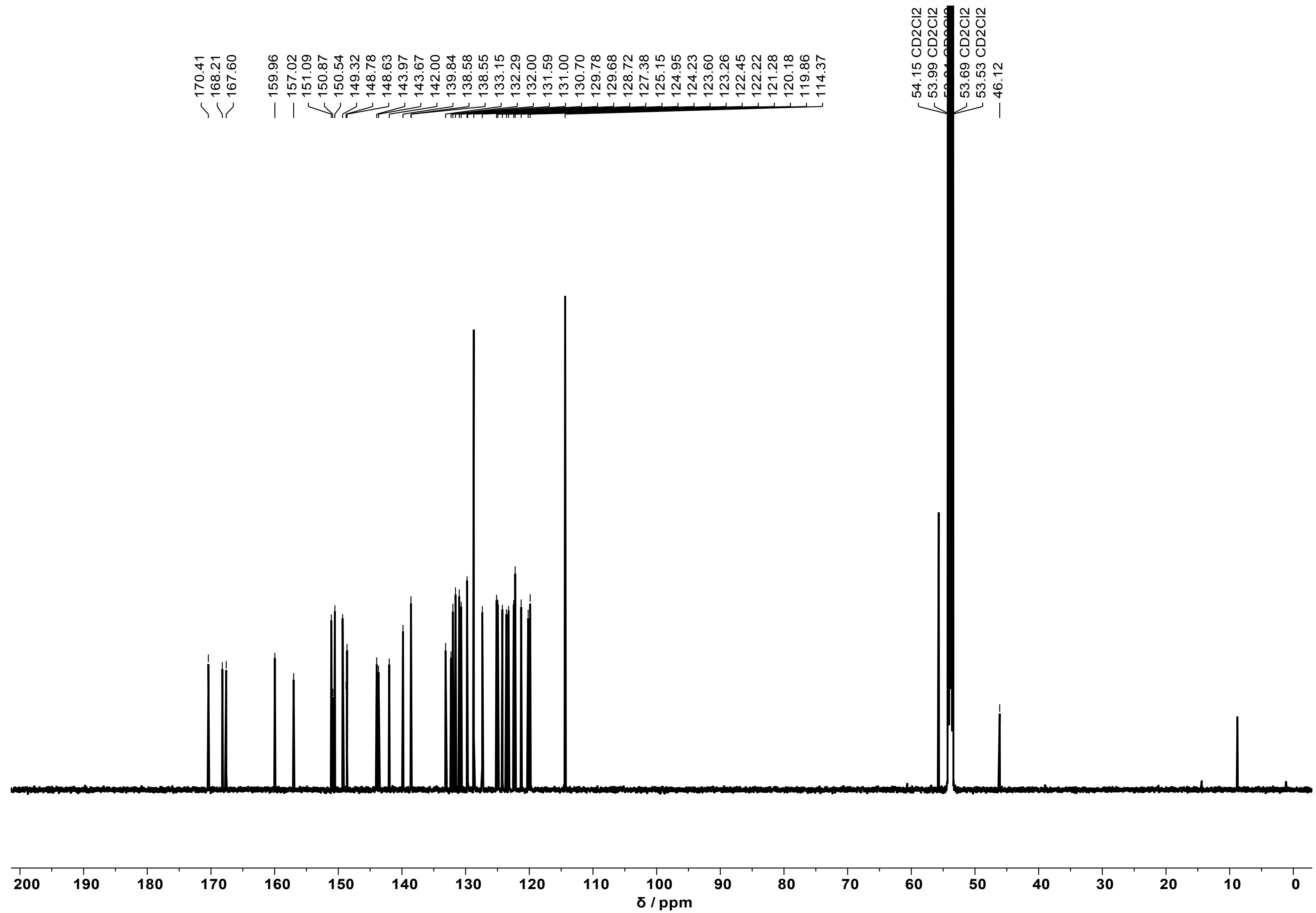

Figure S87: ^{13}C NMR spectrum (176 MHz, DCM-d_2) of IrCN1(P1A3)

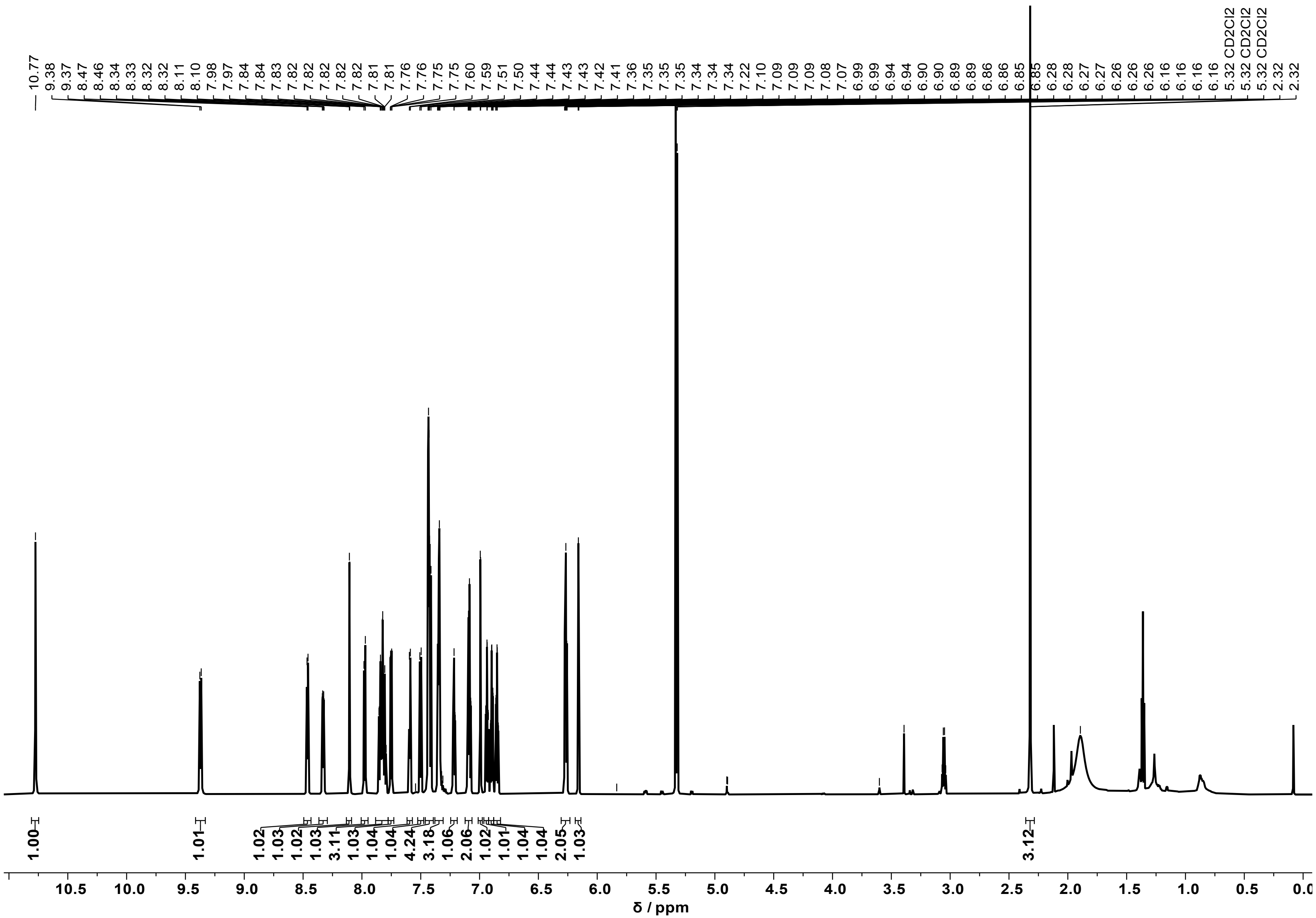

Figure S88: ^{1}H NMR spectrum (700 MHz, DCM-d_2) of IrCN1(P8A1)

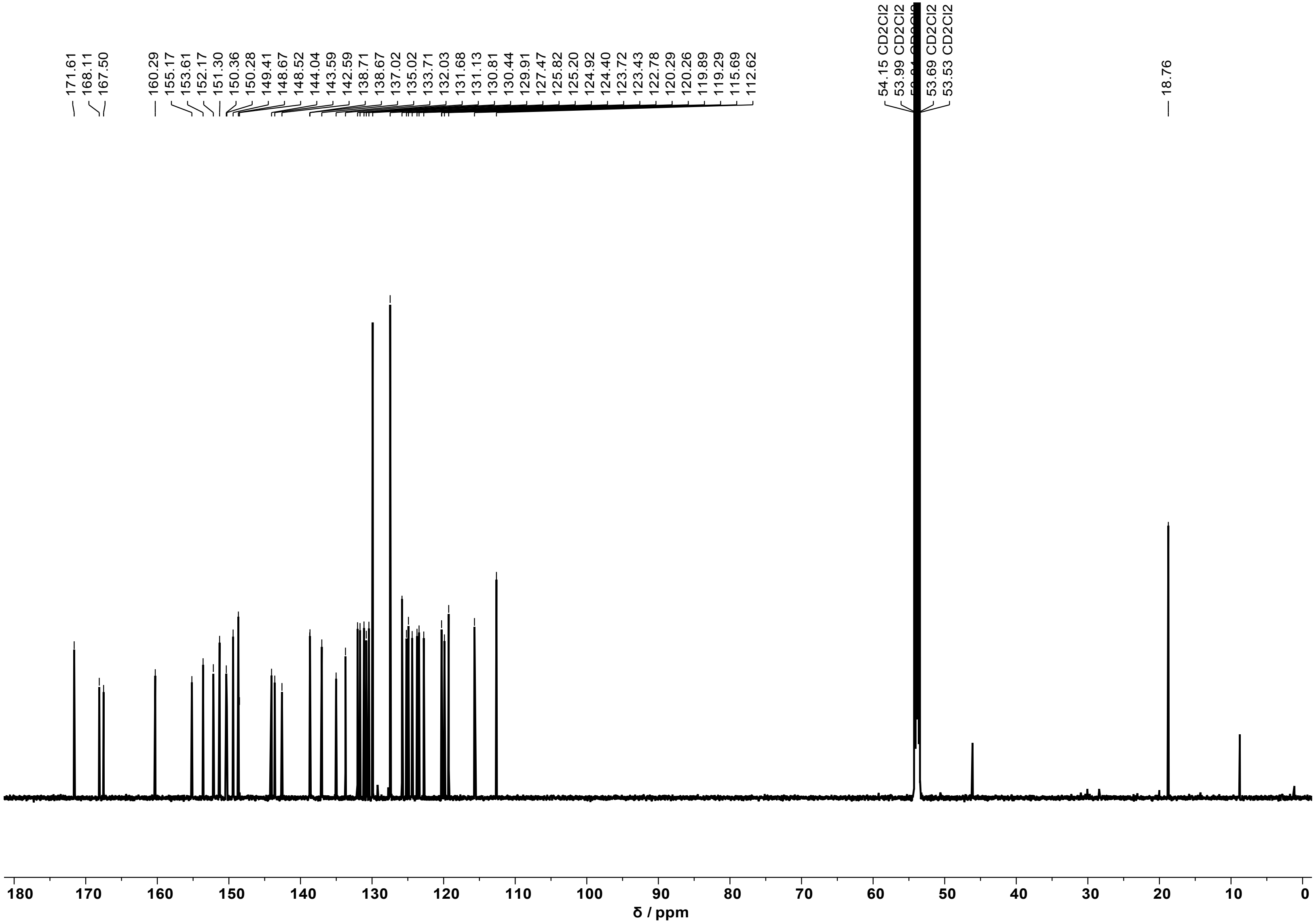

Figure S89: ^{13}**C NMR spectrum (176 MHz, DCM-d_2) of IrCN1(P8A1)**

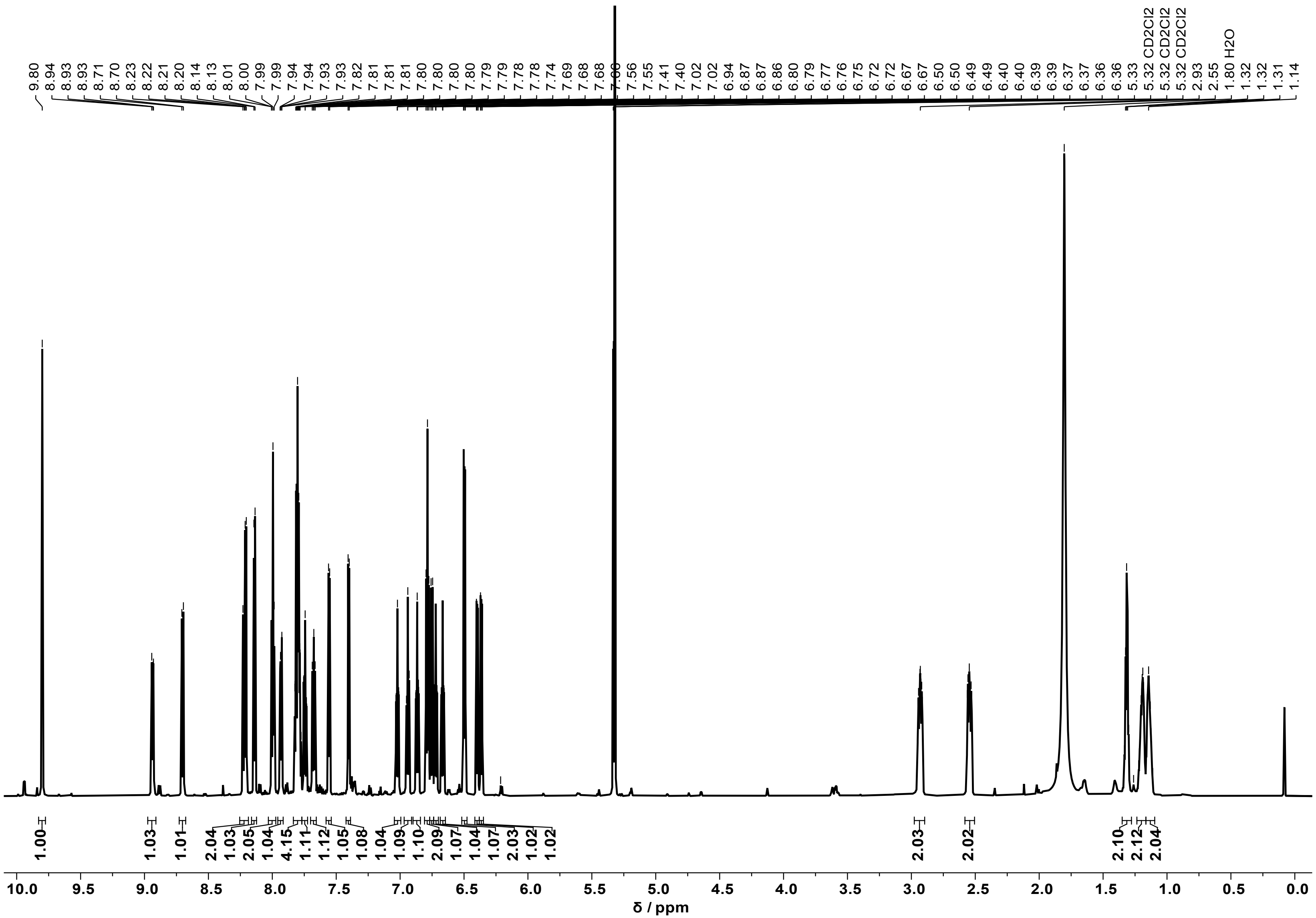

Figure S90: ^{1}H NMR spectrum (700 MHz, DCM-d_2) of IrCN2(P3A2)

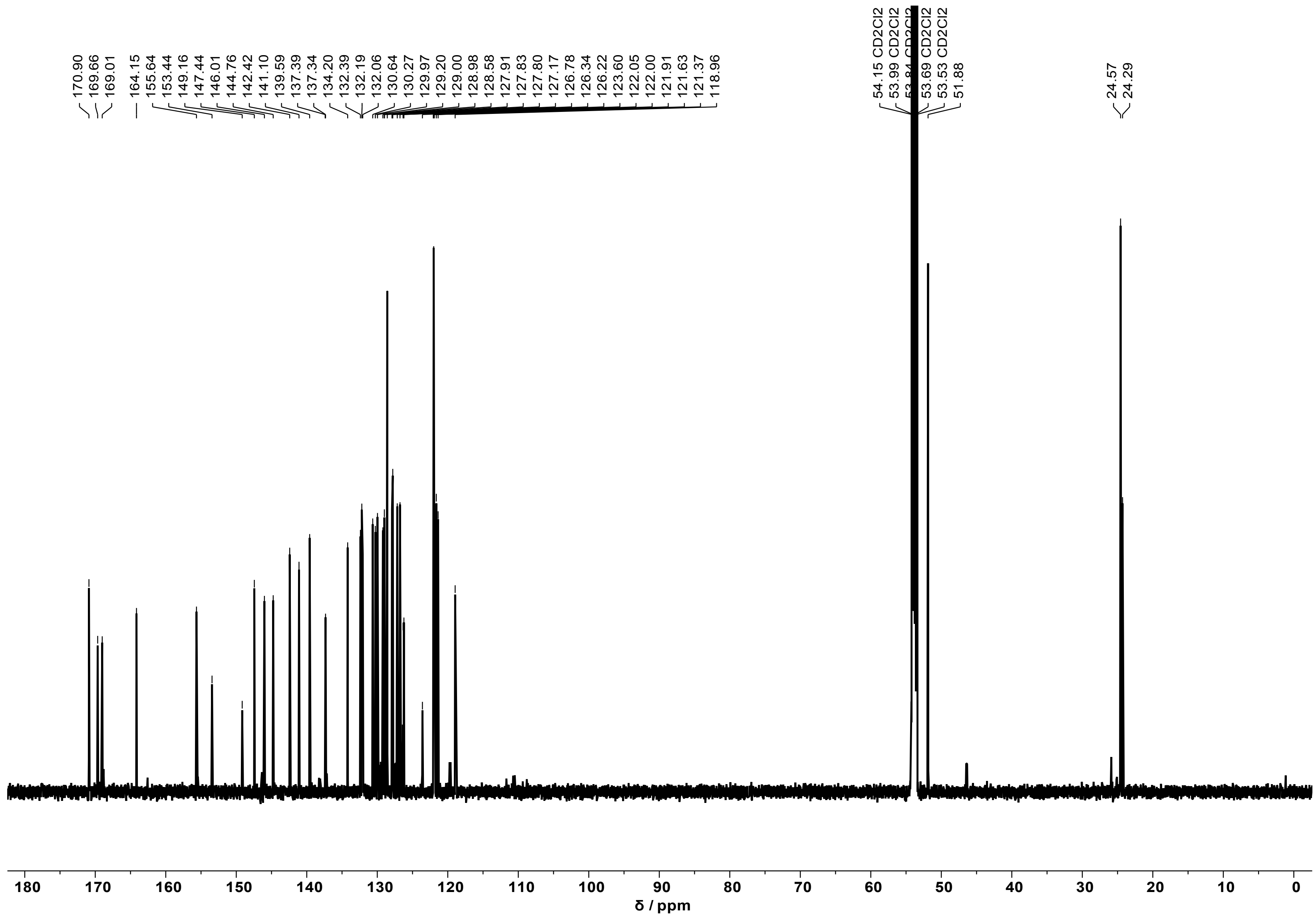

Figure S91: ^{13}C **NMR spectrum (176 MHz, DCM-d_2) of IrCN2(P3A2)**

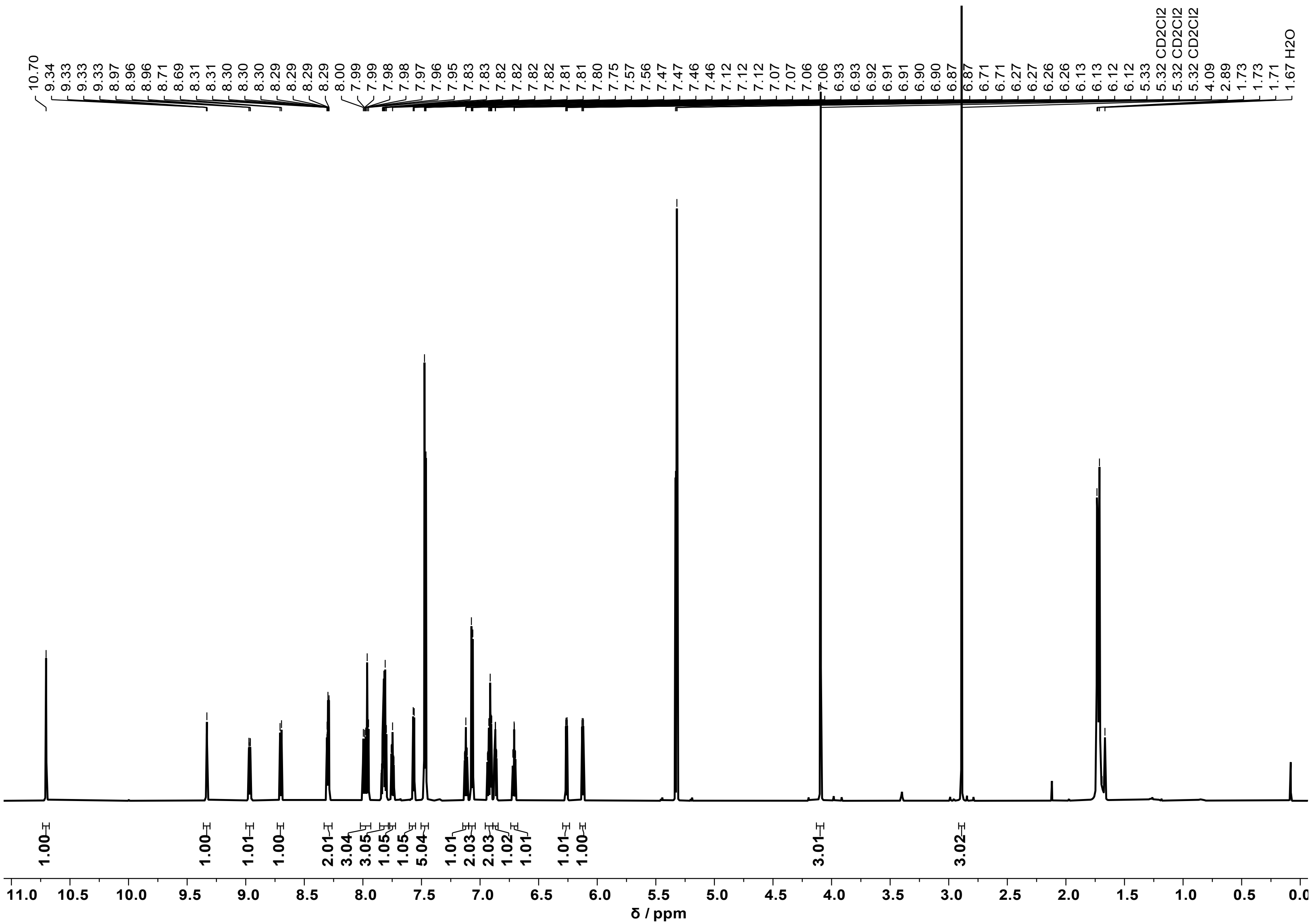

Figure S92: ^{1}H NMR spectrum (700 MHz, DCM-d_2) of IrCN2(P6A9)

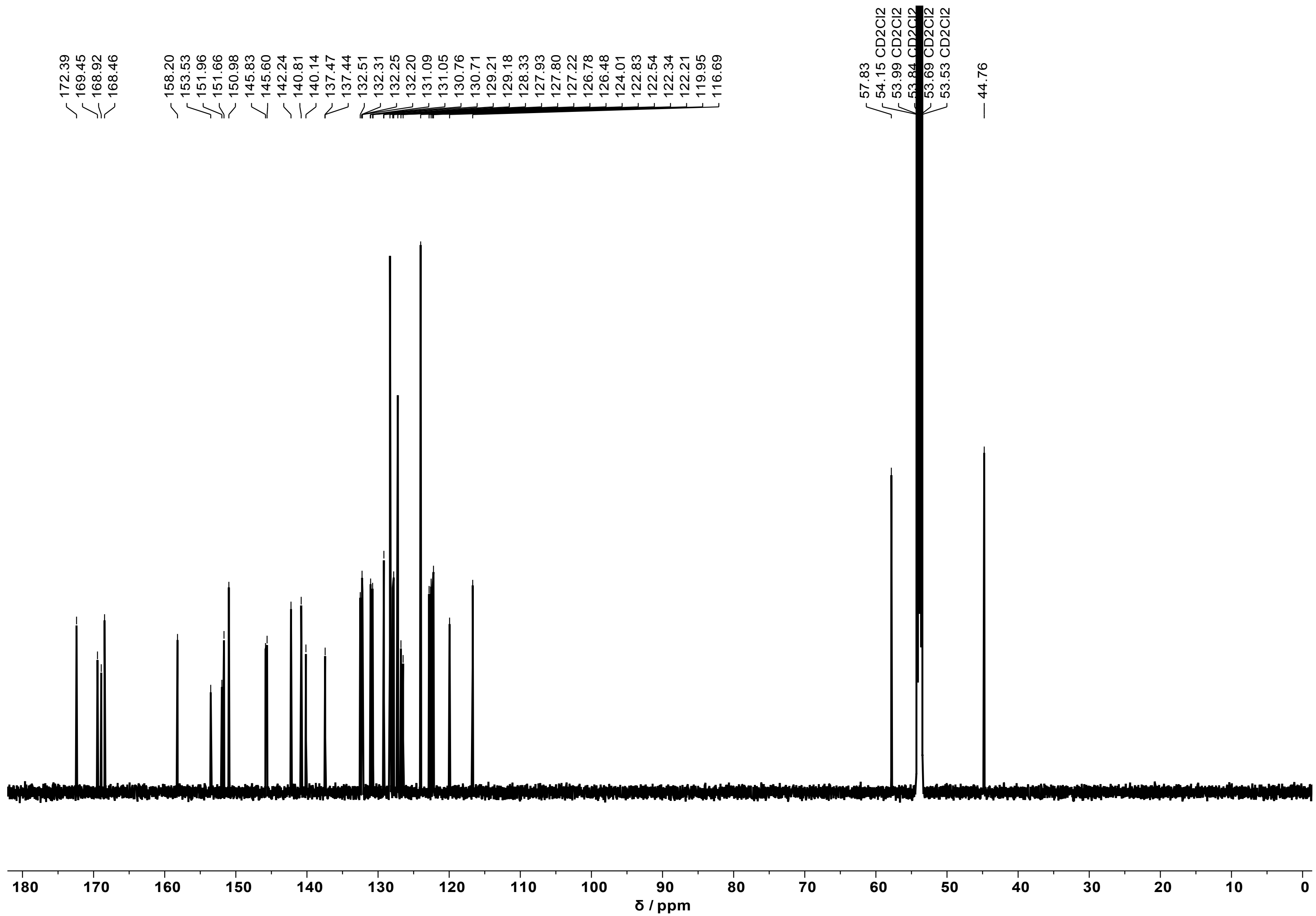

Figure S93: ^{13}C NMR spectrum (176 MHz, DCM-d_2) of IrCN2(P6A9)

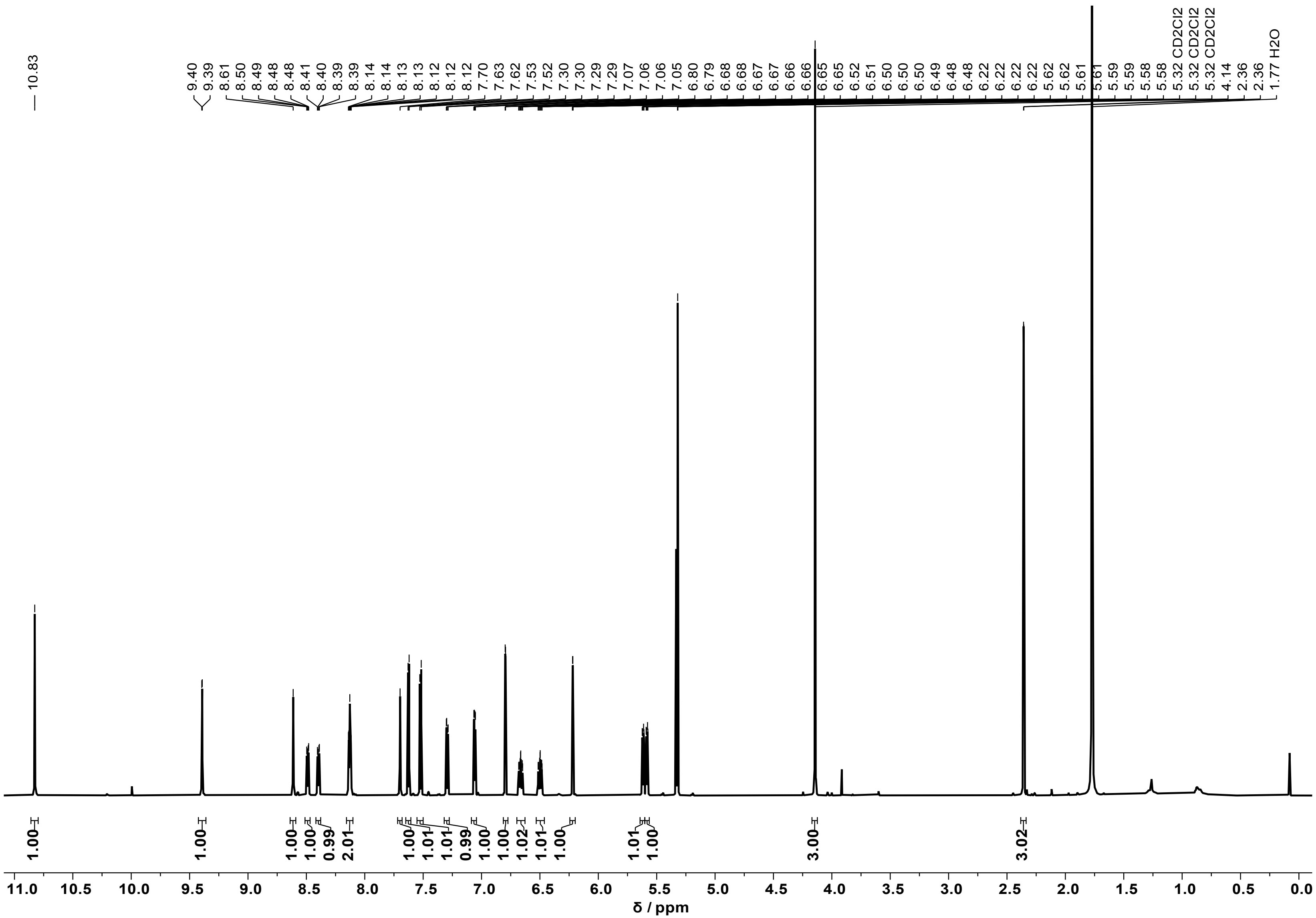

Figure S94: ^{1}H NMR spectrum (700 MHz, DCM-d_2) of IrCN3(P6A1)

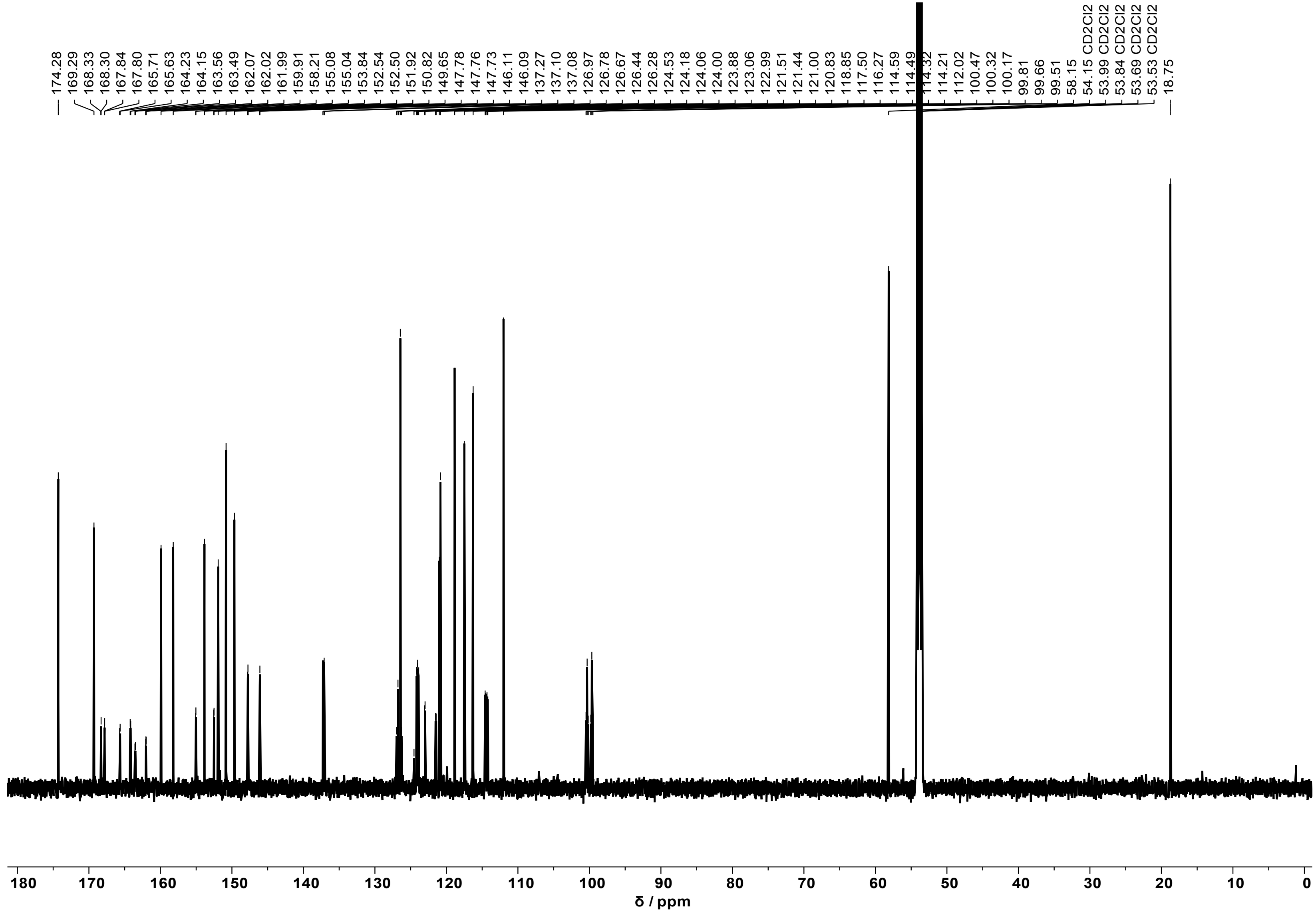

Figure S95: ^{13}C **NMR spectrum (176 MHz, DCM-d_2) of IrCN3(P6A1)**

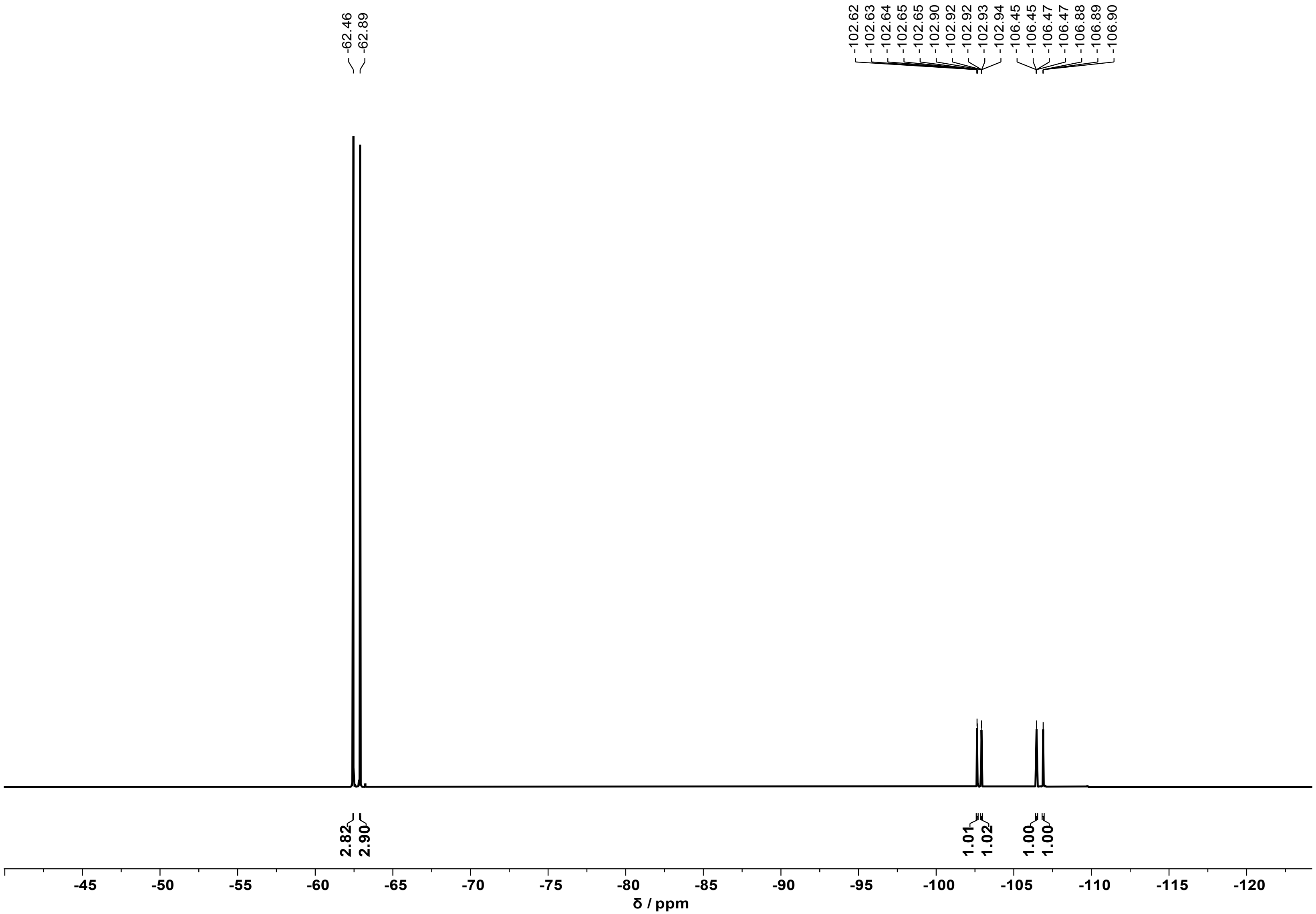

Figure S96: ^{19}F NMR spectrum (659 MHz, DCM-d_2) of IrCN3(P6A1)

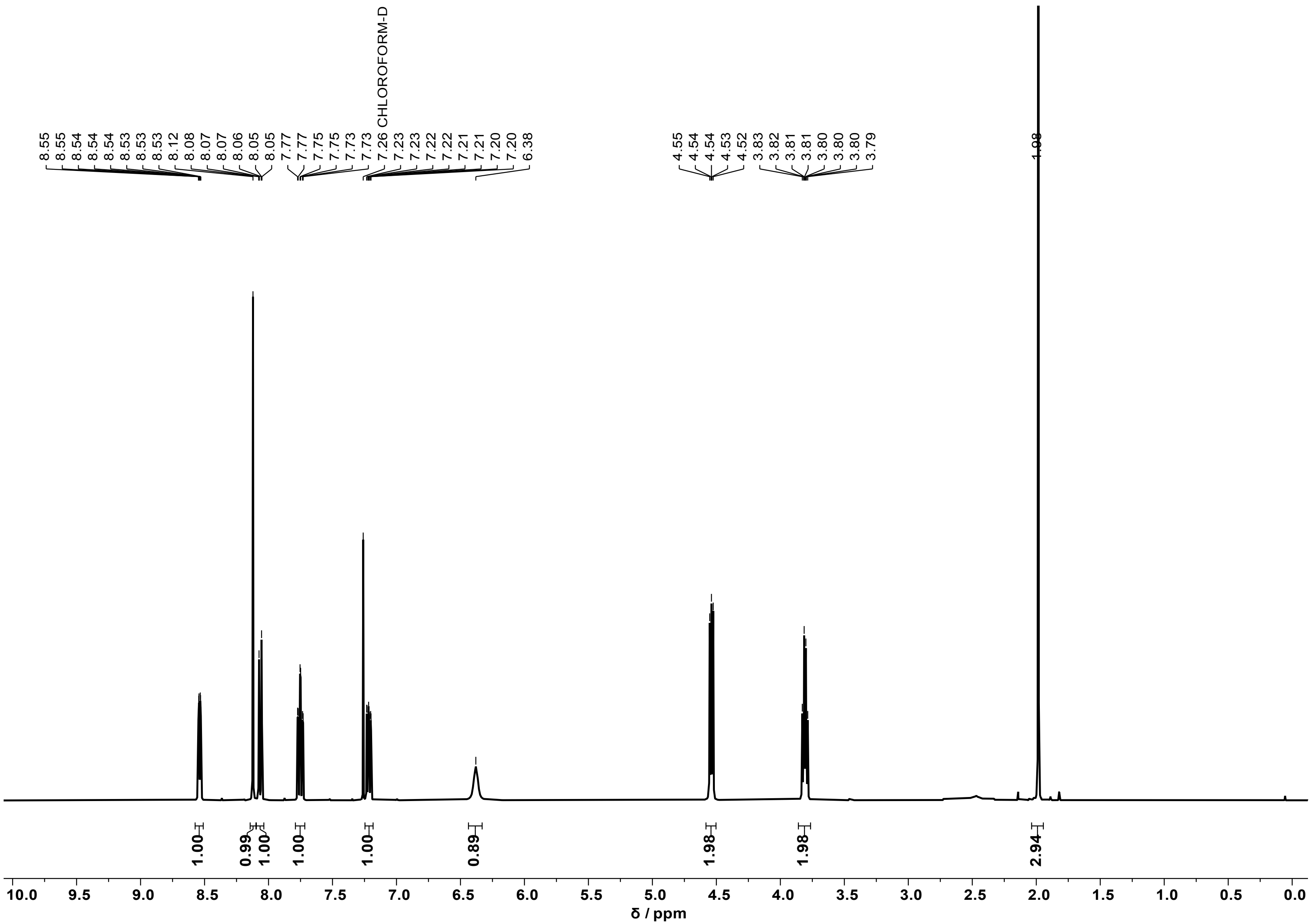

Figure S97: ¹H NMR spectrum (400 MHz, C*D*Cl$_3$) of M2Y1

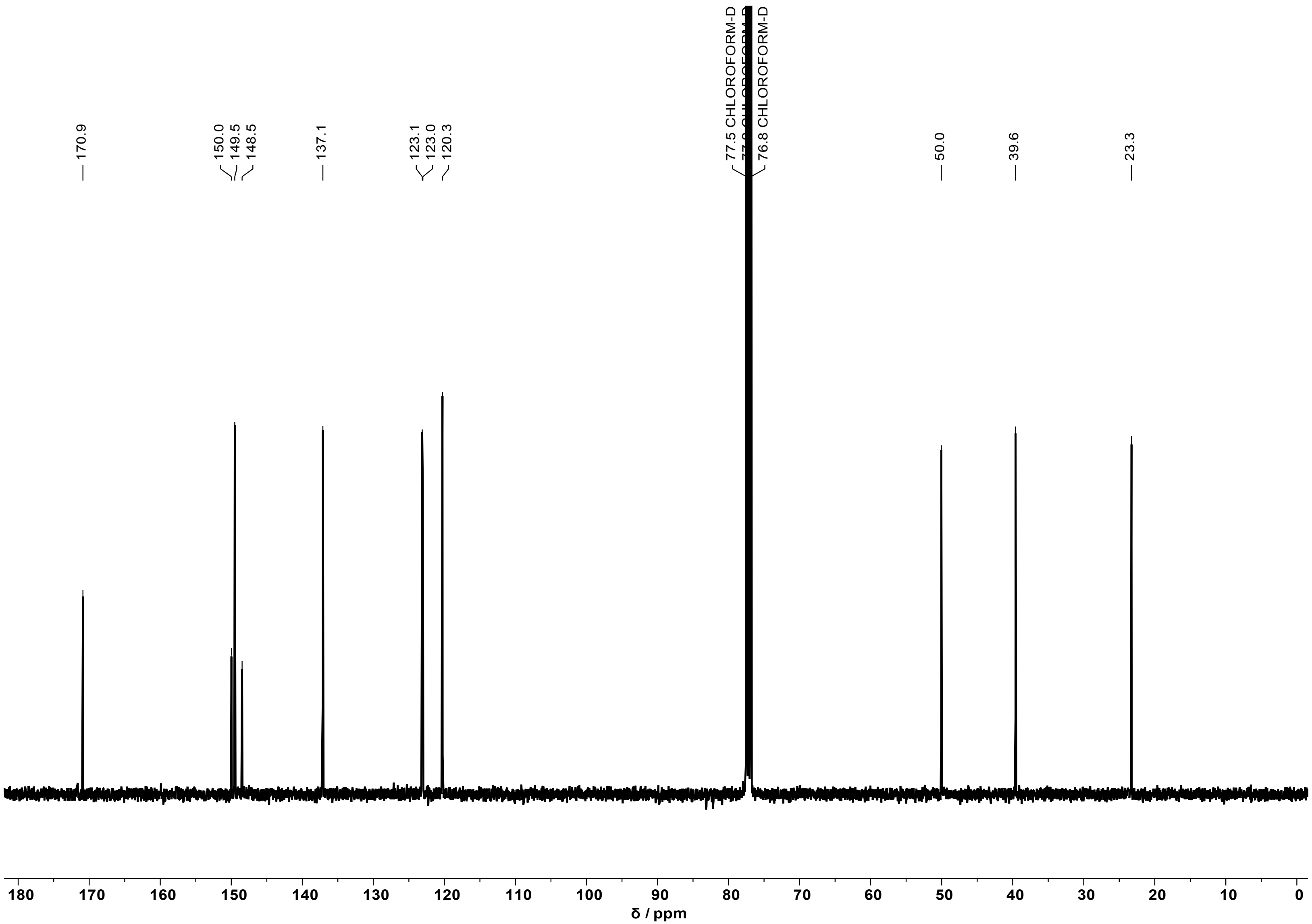

Figure S98: ^{13}C NMR spectrum (101 MHz, C*D*Cl$_3$) of M2Y1

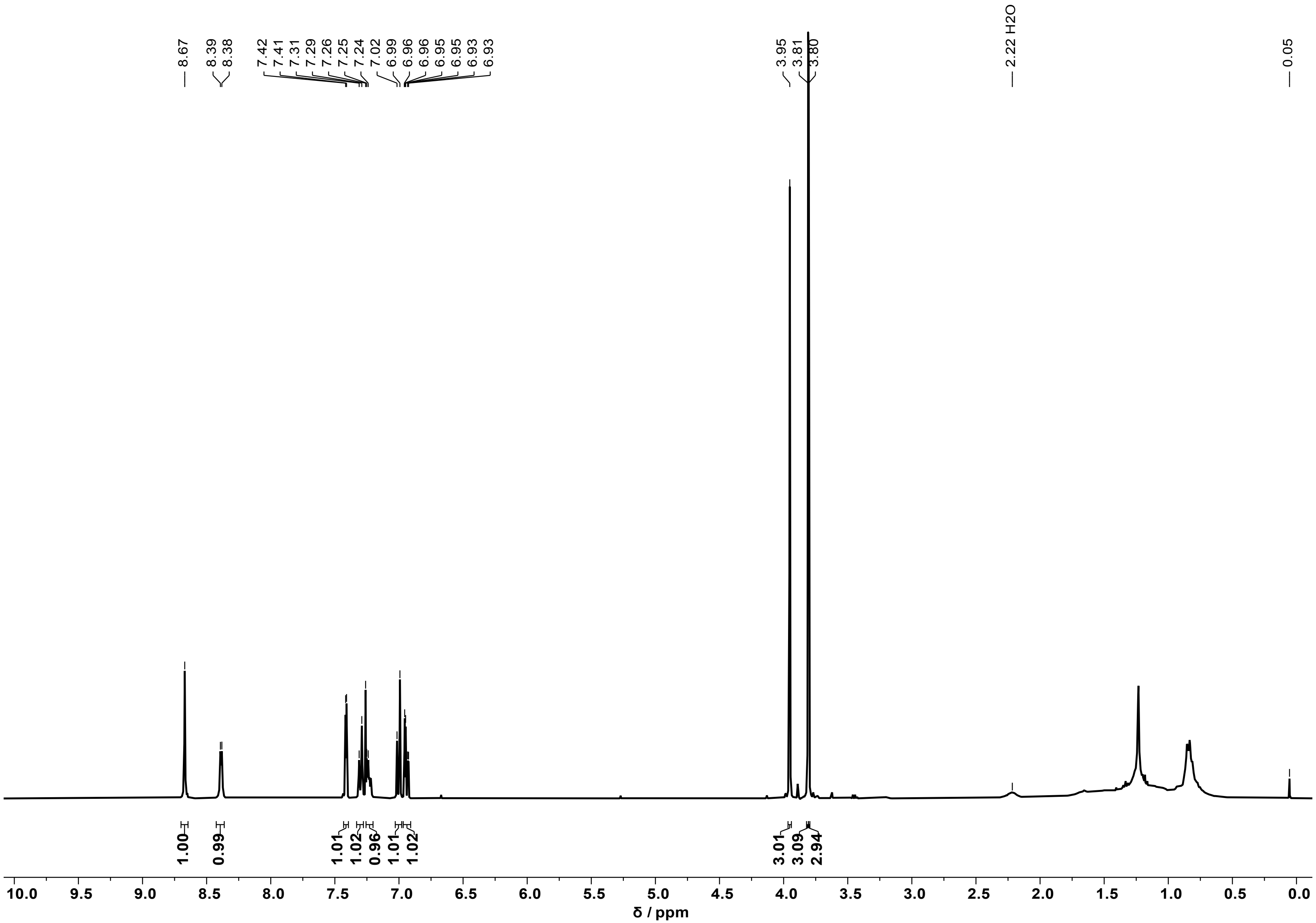

Figure S99: ^{1}H NMR spectrum (400 MHz, C*D*Cl$_3$) of M12Y3

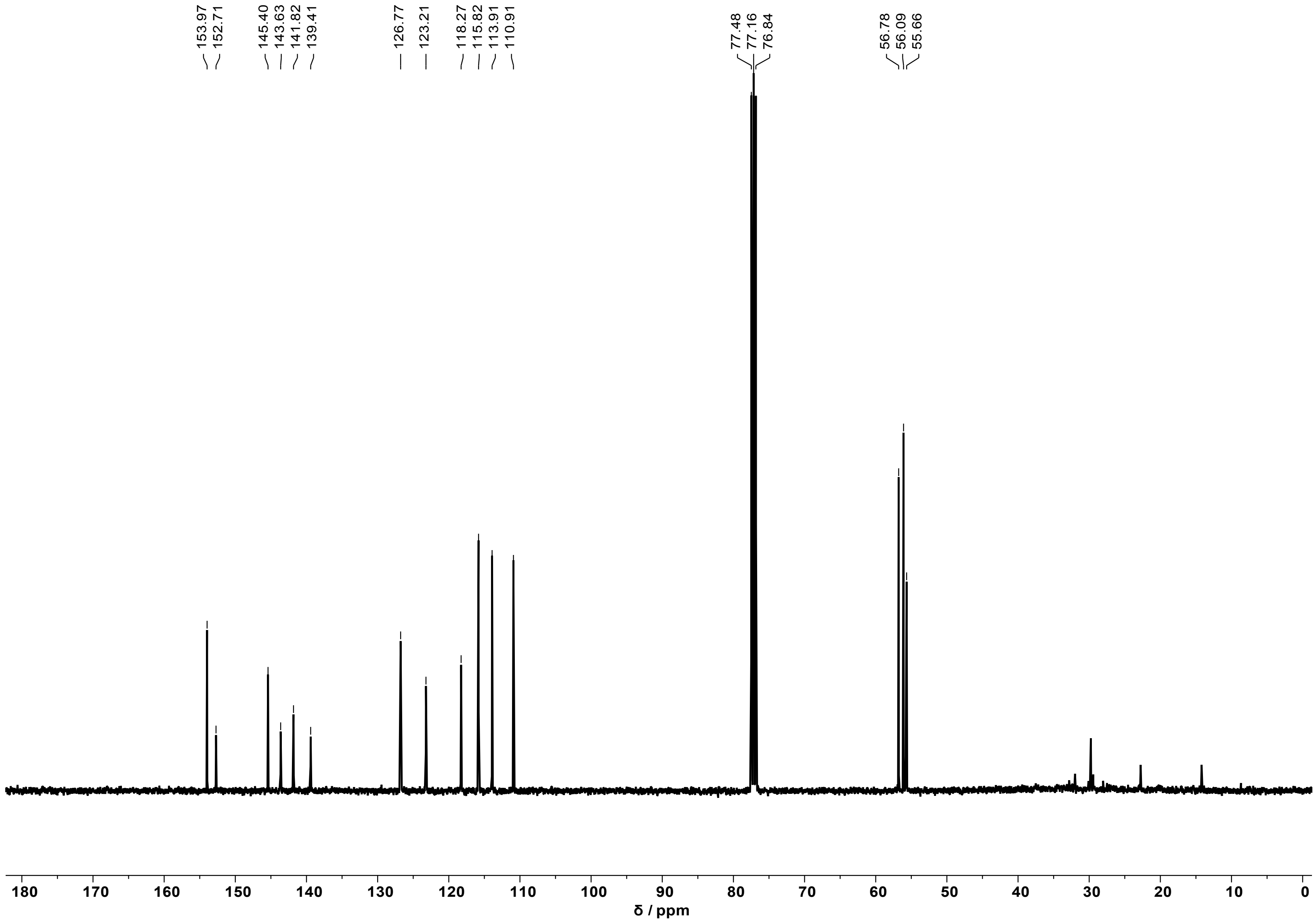

Figure S100: ^{13}C NMR spectrum (101 MHz, C*D*Cl$_3$) of M12Y3

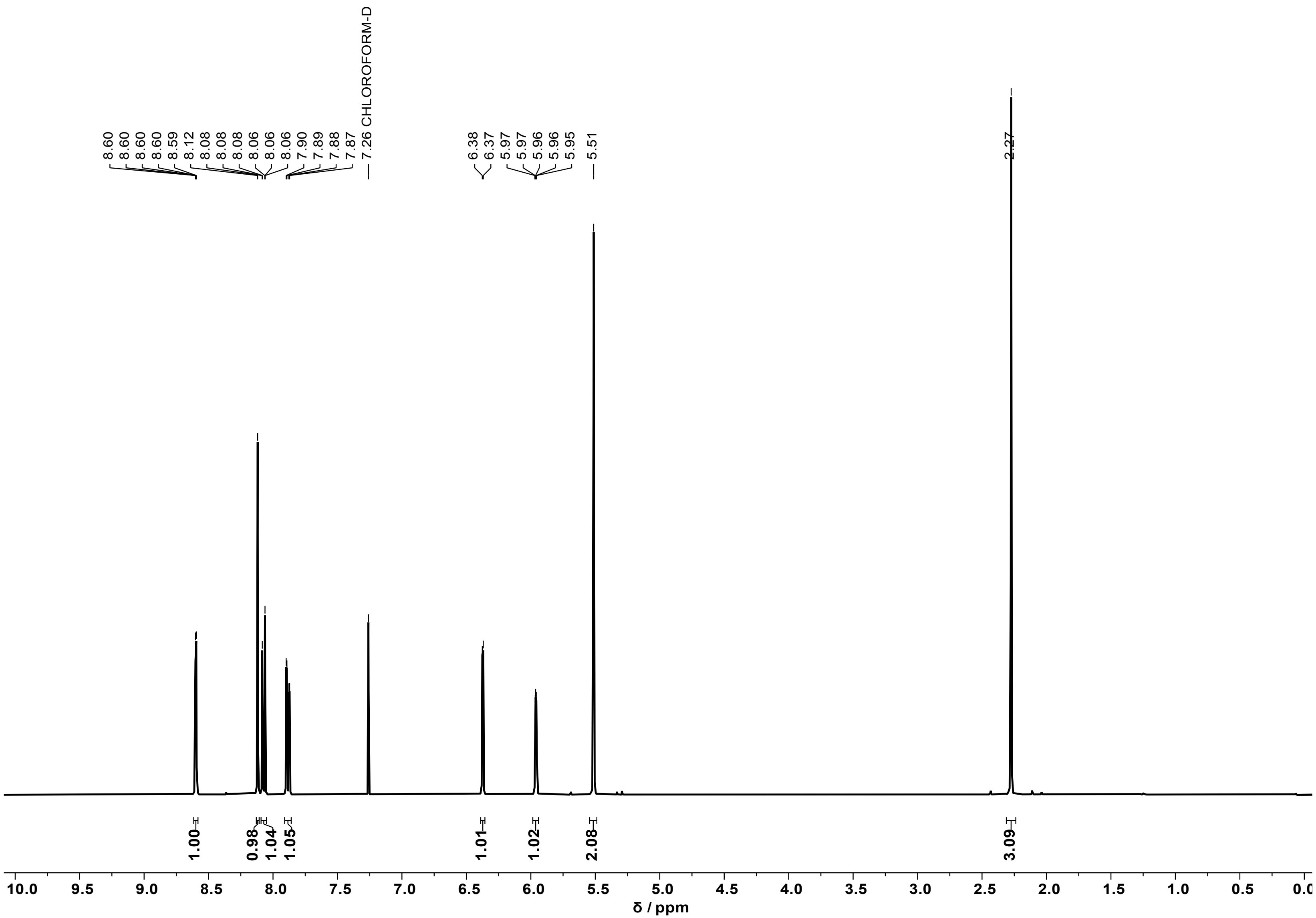

Figure S101: ^{1}H NMR spectrum (400 MHz, C*D*Cl$_3$) of M18Y4

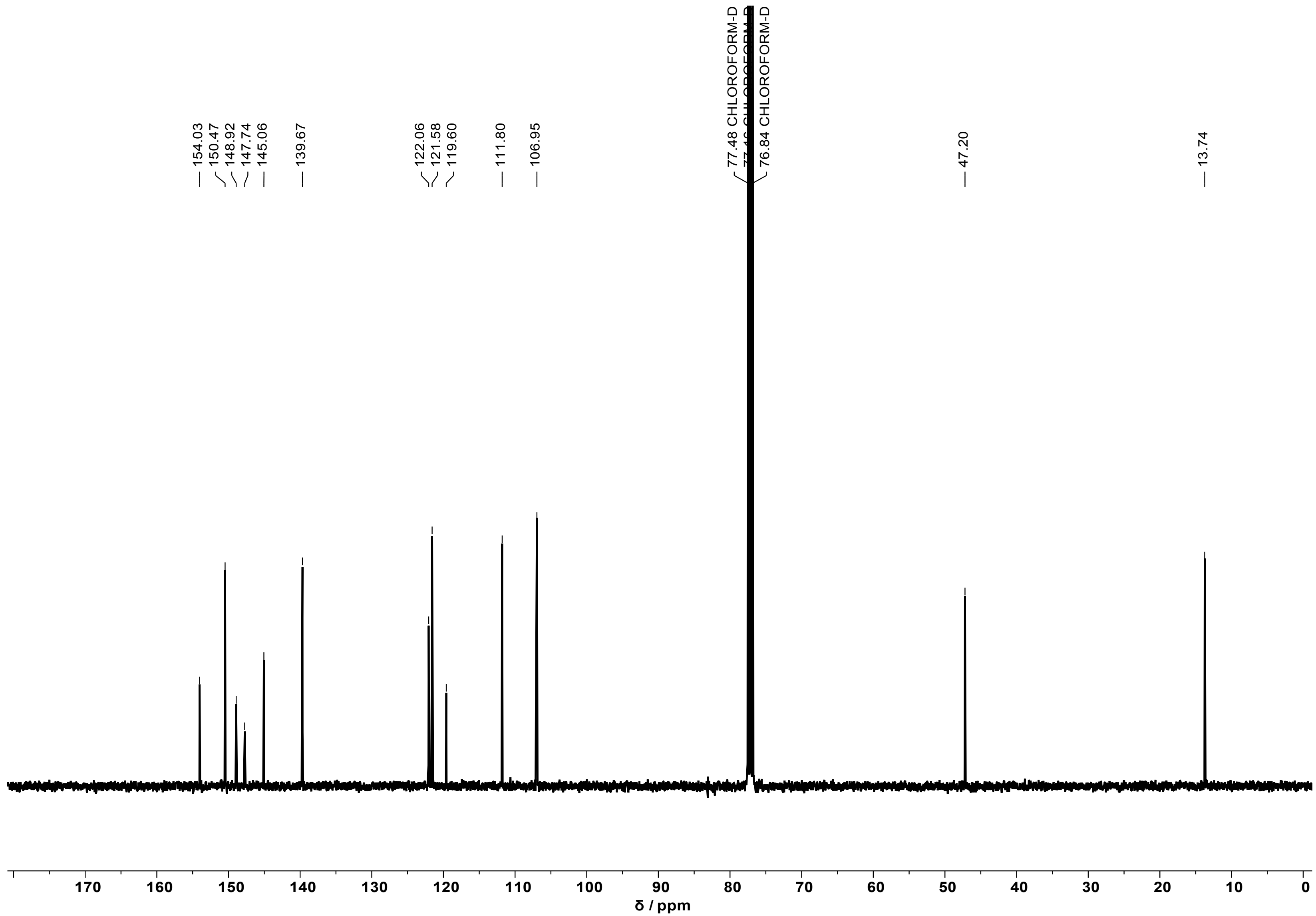

Figure S102: ^{13}C NMR spectrum (101 MHz, C*D*Cl_3) of M18Y4

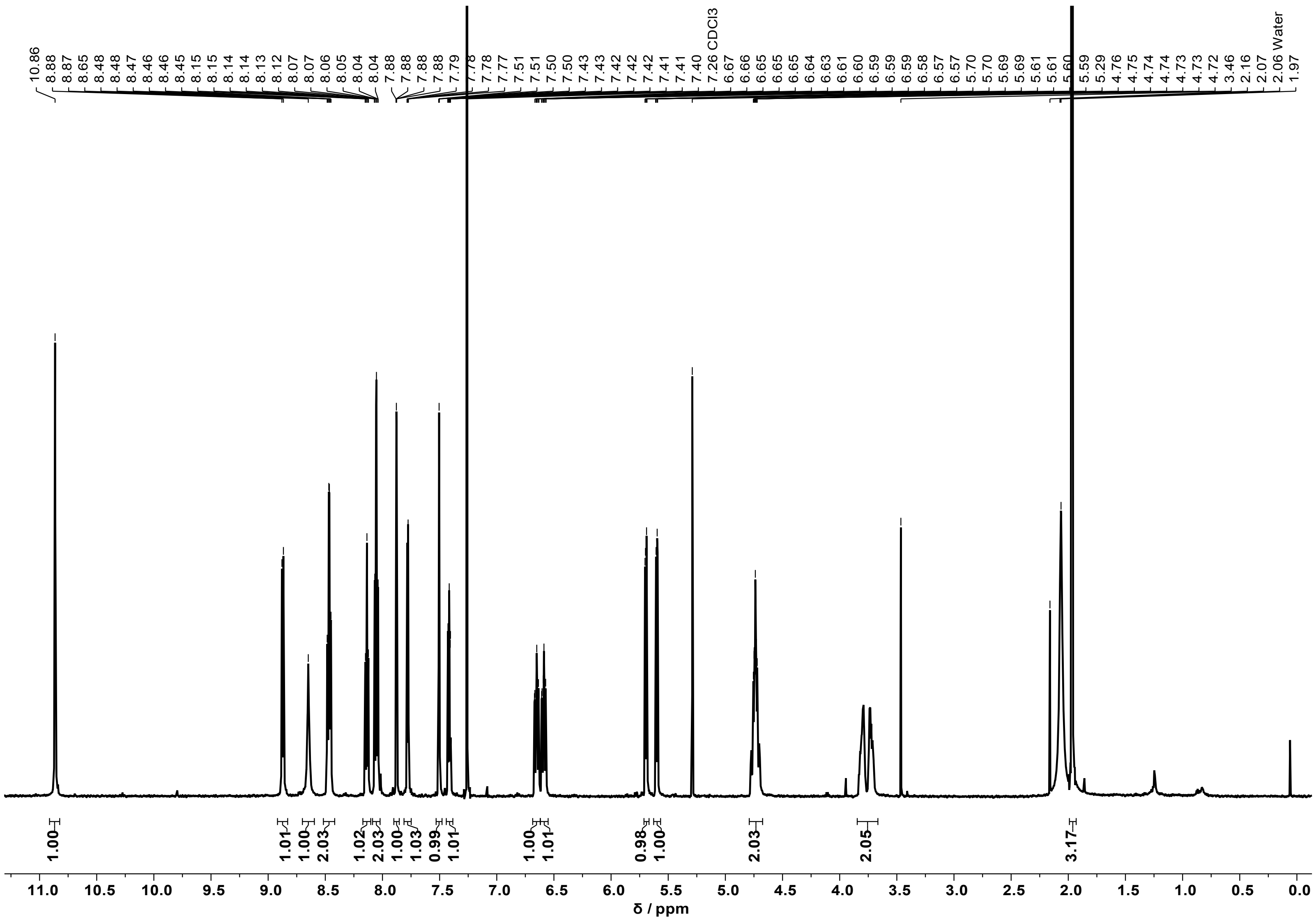

Figure S103: ^{1}H NMR spectrum (600 MHz, C*D*Cl$_3$) of IrCN3(M2Y1)

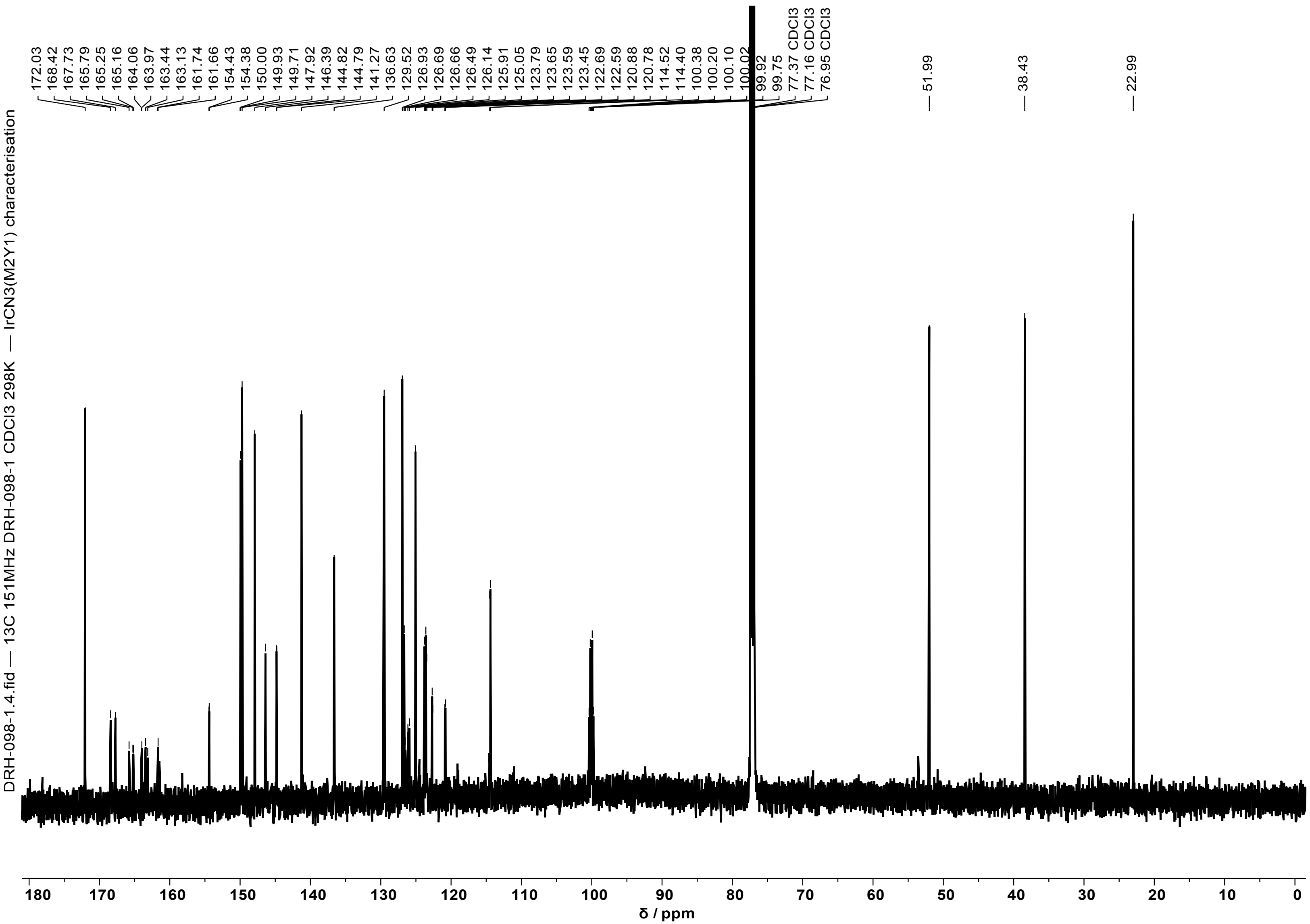

Figure S104: ^{13}C NMR spectrum (151 MHz, $CDCl_3$) of IrCN3(M2Y1)

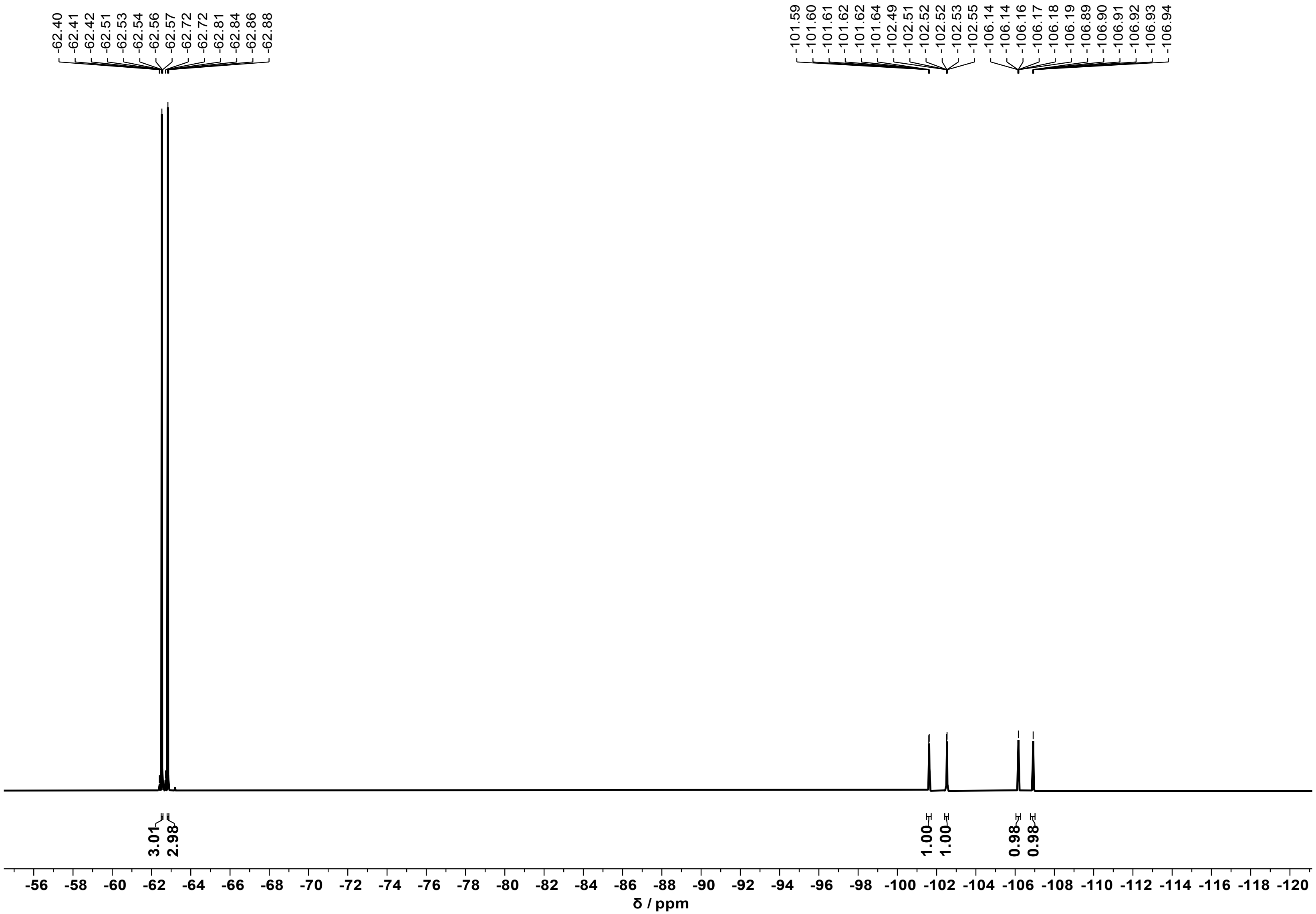

Figure S105: ^{19}F NMR spectrum (565 MHz, C*D*Cl$_3$) of IrCN3(M2Y1)

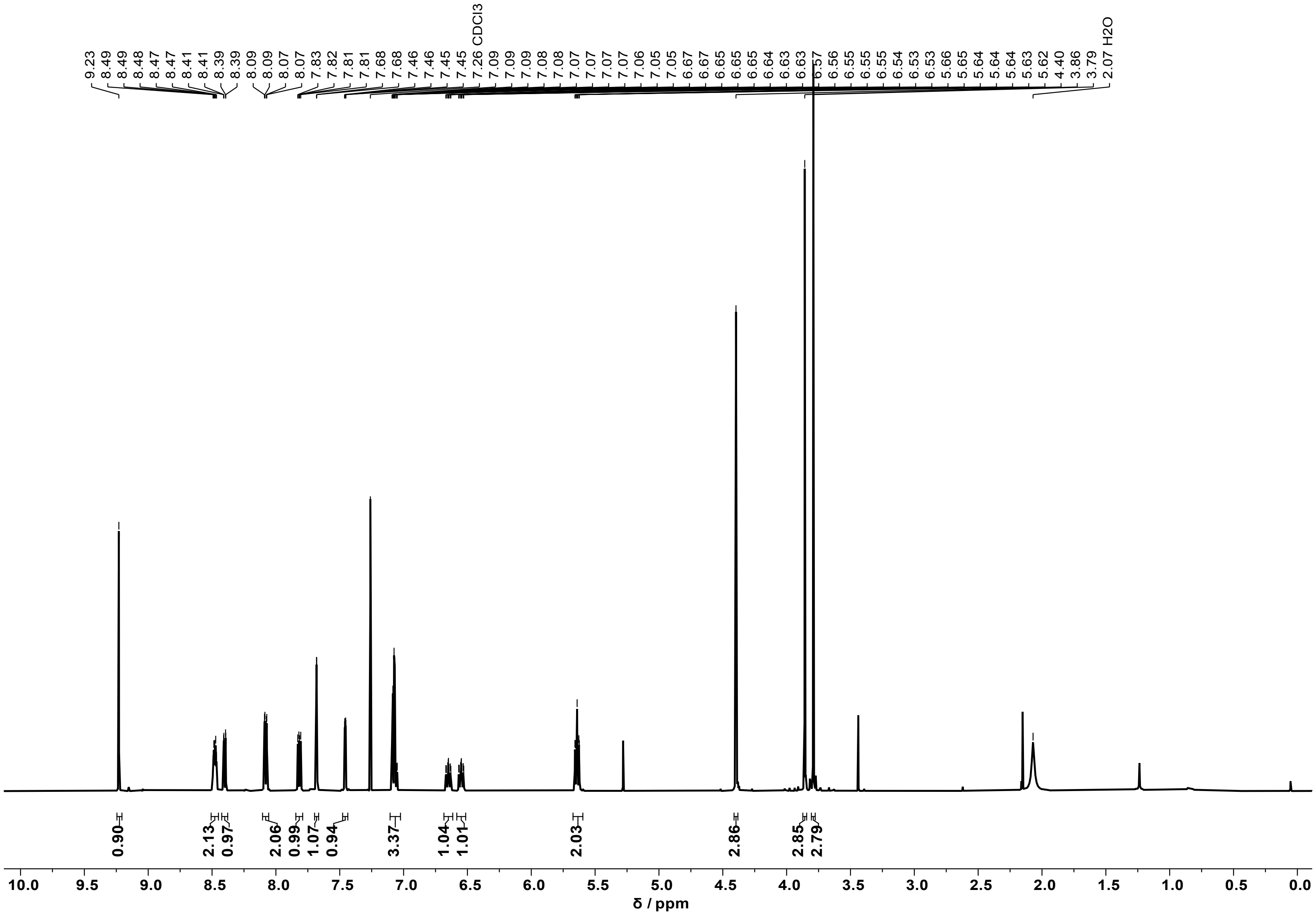

Figure S106: ^{1}H NMR spectrum (600 MHz, C*D*Cl$_3$) of IrCN3(M12Y3)

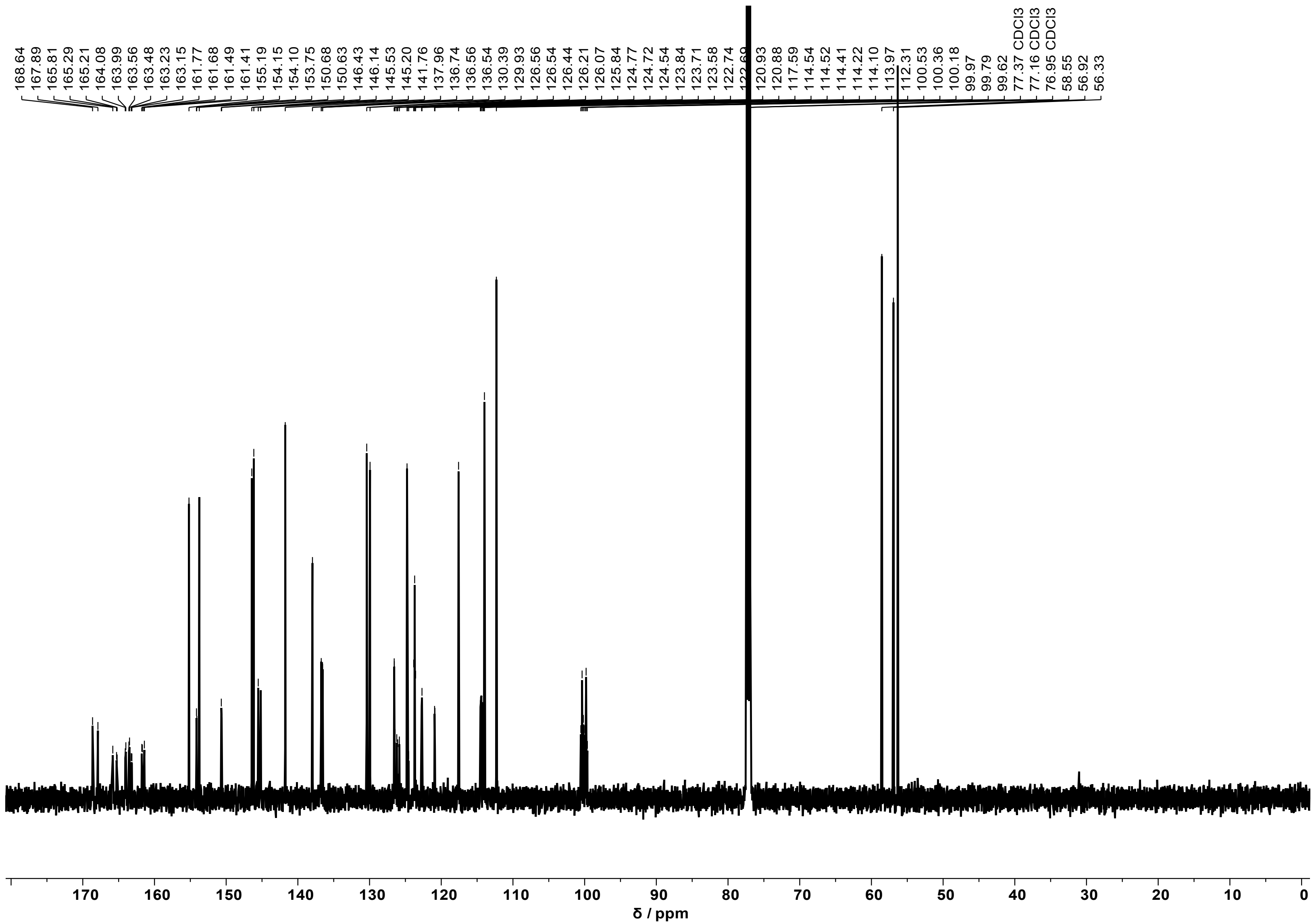

Figure S107: ^{13}C NMR spectrum (151 MHz, C*D*Cl$_3$) of IrCN3(M12Y3)

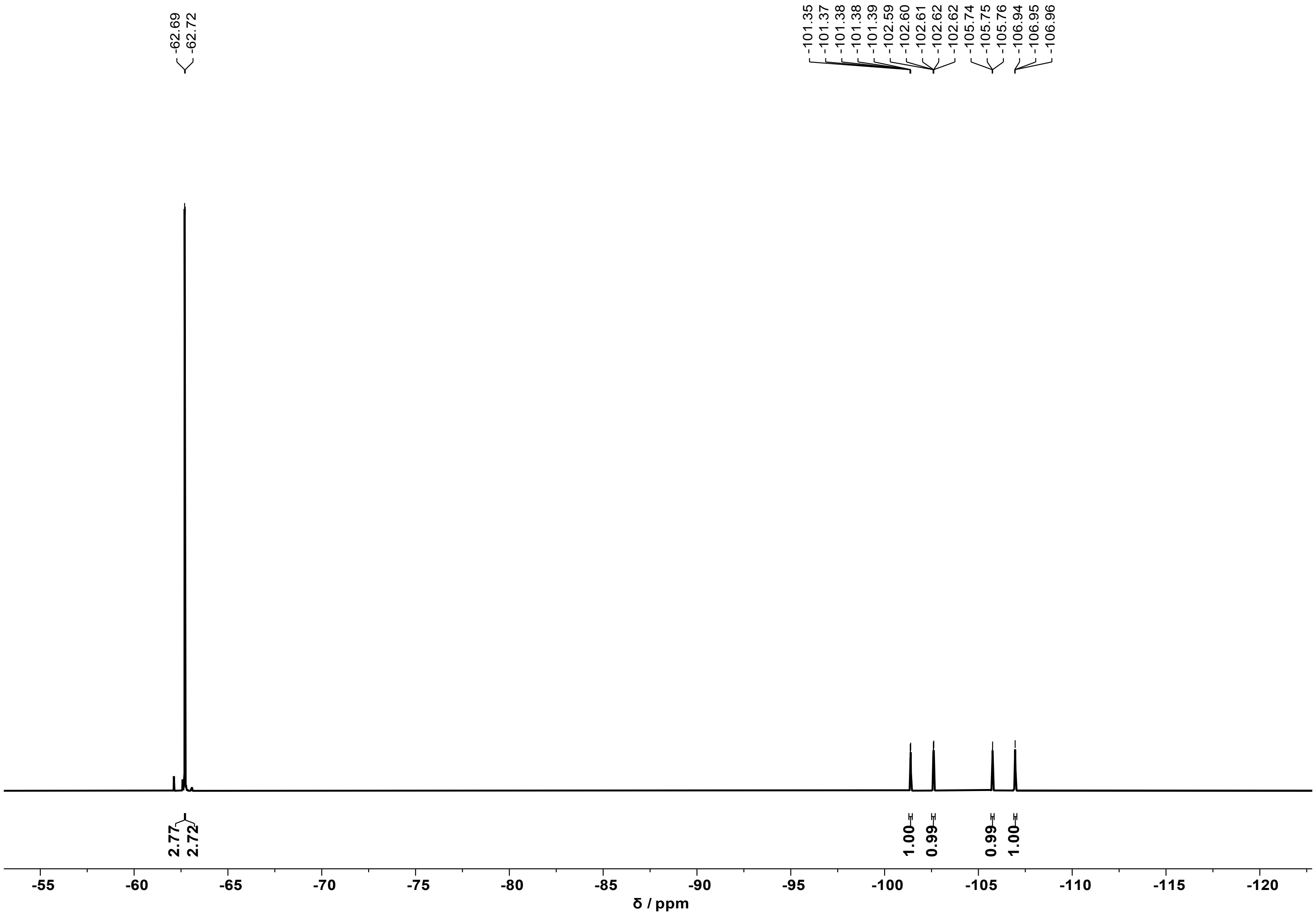

Figure S108: ^{19}F NMR spectrum (565 MHz, C*D*Cl$_3$) of IrCN3(M12Y3)

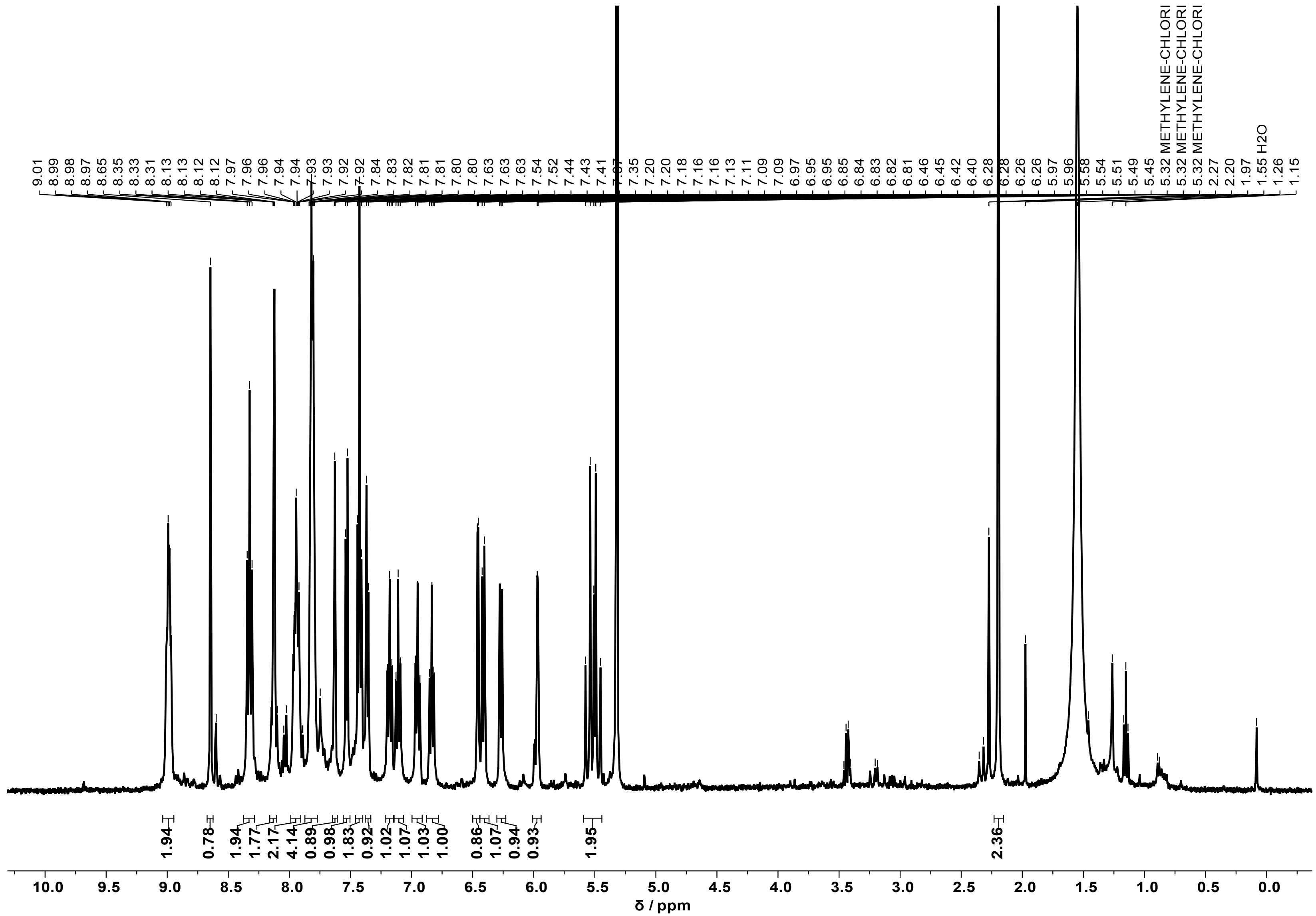

Figure S109: ^{1}H NMR spectrum (400 MHz, CD_2Cl_2) of IrCN2(M18Y4)-PF_6

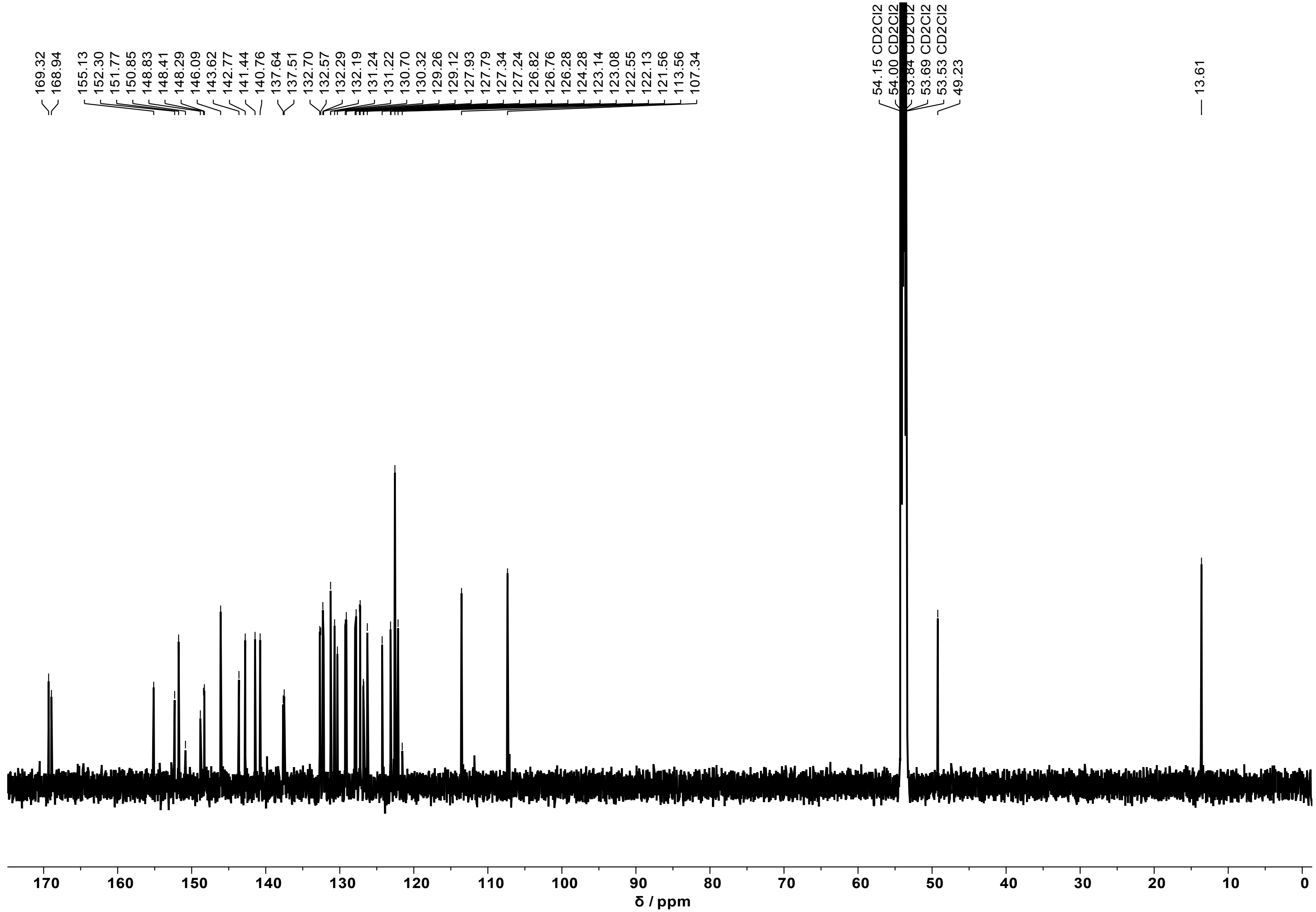

Figure S110: ^{13}C NMR spectrum (151 MHz, CD_2Cl_2) of IrCN2(M18Y4)-PF$_6$

11. References

(1) Kench, T.; Rahardjo, A.; Terrones, G. G.; Bellamkonda, A.; Maher, T. E.; Storch, M.; Kulik, H. J.; Vilar, R. A Semi-Automated, High-Throughput Approach for the Synthesis and Identification of Highly Photo-Cytotoxic Iridium Complexes. *Angew. Chem. Int. Ed.* **2024**, *63* (18), e202401808. https://doi.org/10.1002/anie.202401808.

(2) Husbands, D. R.; Özsan, Ç.; Welsh, A.; Gammons, R. J.; Frei, A. High-Throughput Triazole-Based Combinatorial Click Chemistry for the Synthesis and Identification of Functional Metal Complexes. *Nat Commun* **2025**, *16* (1), 11195. https://doi.org/10.1038/s41467-025-67341-z.

(3) Meng, G.; Guo, T.; Ma, T.; Zhang, J.; Shen, Y.; Sharpless, K. B.; Dong, J. Modular Click Chemistry Libraries for Functional Screens Using a Diazotizing Reagent. *Nature* **2019**, *574* (7776), 86–89. https://doi.org/10.1038/s41586-019-1589-1.

(4) Hulstaert, N.; Shofstahl, J.; Sachsenberg, T.; Walzer, M.; Barsnes, H.; Martens, L.; Perez-Riverol, Y. ThermoRawFileParser: Modular, Scalable, and Cross-Platform RAW File Conversion. *J. Proteome. Res.* **2020**, *19* (1), 537–542. https://doi.org/10.1021/acs.jproteome.9b00328.

(5) Mosmann, T. Rapid Colorimetric Assay for Cellular Growth and Survival: Application to Proliferation and Cytotoxicity Assays. *J. Immunol. Methods* **1983**, *65* (1), 55–63. https://doi.org/10.1016/0022-1759(83)90303-4.

(6) CrysAlisPro. CrysAlisPro, Oxford Diffraction Ltd. Version 1.171.34.41, Oxford Diffraction Ltd. Version 1.171.34.41.

(7) Empirical absorption correction using spherical harmonics implemented in SCALE3 ABSPACK scaling algorithm within CrysAlisPro software. Scale3. Empirical absorption correction using spherical harmonics, implemented in SCALE3 ABSPACK scaling algorithm within CrysAlisPro software, p Oxford Diffraction Ltd. Version 1.171.34.40.

(8) Dolomanov, O. V.; Bourhis, L. J.; Gildea, R. J.; Howard, J. A. K.; Puschmann, H. OLEX2: A Complete Structure Solution, Refinement and Analysis Program. *J. Appl. Crystallogr.* **2009**, *42* (2), 339–341. https://doi.org/10.1107/S0021889808042726.

(9) Sheldrick, G. M. SHELXT - Integrated Space-Group and Crystal-Structure Determination. *Acta Cryst. A* **2015**, *71* (1), 3–8. https://doi.org/10.1107/S2053273314026370.
